# Stapling of β‑Glucans Increases Antibody Binding

**DOI:** 10.1021/jacs.5c12690

**Published:** 2025-10-07

**Authors:** Jiří Ledvinka, Richard Kullmann, Emelie E. Reuber, Thomas Weikl, Manuel G. Ricardo, Peter H. Seeberger

**Affiliations:** † Department of Biomolecular Systems, Max-Planck-Institute of Colloids and Interfaces, Am Mühlenberg 1, 14476 Potsdam, Germany; ‡ Institute of Chemistry and Biochemistry, Freie Universität Berlin, Arnimallee 22, 14195 Berlin, Germany

## Abstract

Higher-order structures are essential for the function of biological macromolecules. Tuning the conformational space of peptides by stapling improves their pharmacological properties. The three-dimensional (3D) structures of glycans are much less well understood than those of peptides and oligonucleotides, and willful modulation of oligosaccharide structures to improve binding to proteins has not been described to date. Herein, we describe stapling of β-(1,3)-glucans to tune their conformation, aiming to mimick the naturally occurring triple helix. The stapled glycans are prepared by automated glycan assembly, followed by linker construction and ring-closure assisted by solid-phase peptide synthesis. Thereby, staples of different lengths, polarities, and topologies can be readily introduced. Molecular dynamics simulations served to evaluate the effect of stapling on the conformational space. Glycan microarray experiments revealed that stapled glycans bound significantly more tightly to monoclonal mouse and rabbit antibodies than did linear glycans. Controlling the conformational space of short oligosaccharides creates opportunities for synthetic glycans in drug and vaccine development.

## Graphical abstract

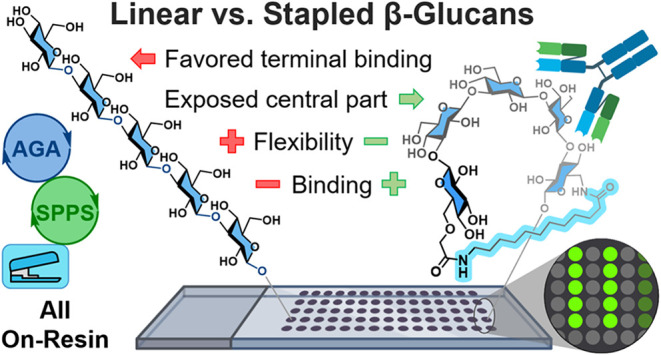

## Introduction

Glycans play many crucial roles in nature, as they serve as construction materials, for energy storage and are present on the surface of nearly all living cells, where they mediate cell–cell recognition and regulate the immune system.^1^ The polysaccharides present on the cell surface of pathogens are built of oligosaccharide repeating units (RUs). The unique three-dimensional structure of successive RUs enables the recognition of individual pathogens by the corresponding biological receptors and antibodies.^2^

Synthetic biomolecules have been key to understanding receptor binding. Defined oligosaccharides mimicking the RU structure and function have been prepared enzymatically and/or chemically.^2,3^ Short oligosaccharides can exhibit different degrees of conformational flexibility depending mainly on the glycosidic linkage and therefore may not resemble the higher-order structures of the parent polysaccharides (Figure 1A).^4–6^

**Figure 1:**
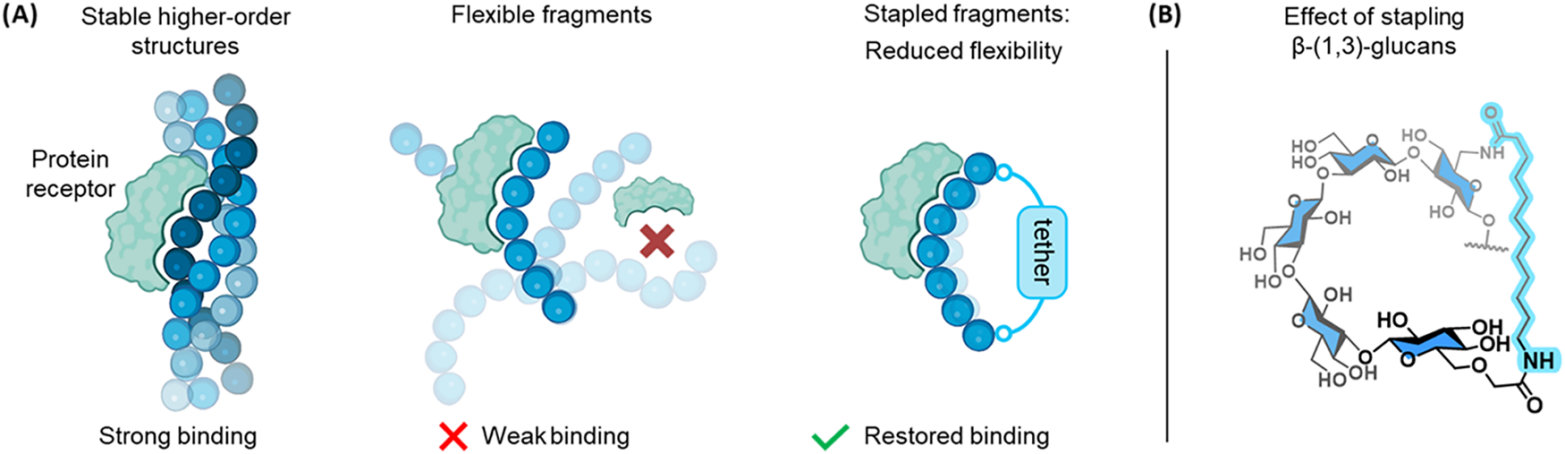
Stapling a flexible oligosaccharide fragment can decrease its conformational freedom, as is the case for the parent polysaccharide. (A) Polysaccharides naturally occurring on the cell walls of many pathogens reach significant lengths and consist of many repeating units. These polysaccharides can form higher-order structures with well-defined conformations. In contrast, short oligosaccharide fragments do not adopt stable higher-order structures and can therefore exist in many conformations, leading to low affinities toward receptors. The conformational freedom can be reduced by stapling the glycan to favor the geometry recognized by the receptors. (B) β-(1,3)-Glucan stapling replaces C6-hydroxyl groups with amines and carboxylic acid groups that can be subsequently connected by virtually any peptide-like linker in order to form the key macrocycle.

Tuning the conformational space of short oligosaccharides can modify their properties.^7,8^ The 3D structure of peptides^9^ and short oligonucleotides has been altered in many different ways.^10–13^ A widely used strategy is stapling, which stabilizes α-helical peptides by chemically connecting two amino acid (AA) side chains, improving their receptor binding and increasing membrane permeation.^14–18^ Stapled peptides bind better to their receptors when the recognized conformation is locked.^18^ Flexible ligands need to undergo conformational changes until they fit in the recognition domain, resulting in a loss of entropy associated with the ligand–receptor complex formation.^19,20^ The conformational space of stapled molecules is comparatively smaller, leading to a decrease in entropy loss.

β-(1,3)-Glucans are present on the cell surface of fungi, serving as pathogen-associated molecular patterns (PAMPs) for a variety of infections such as those with *Candida*.^21^ This particular fungal infection can be fatal and is becoming a global health threat.^22^ β-(1,3)-Glucans are recognized by anti-β-glucan antibodies (Abs) that are indicative of an infection and contribute to an early diagnosis.^23^

In order to better understand the immunological implications of β-(1,3)-glucans, we designed a general stapling strategy for β-(1,3)-glucans, paying particular attention to the consequences for antibody binding.

β-Glucans occur naturally in the cell walls of plants and fungi.^24,25^ With single chains reaching over 100 units in length, they are known to adopt a triple-helix confirmation.^24^ In these helices, primary C6 hydroxyl groups point out of the helix and are ideal for the introduction of handles for stapling (Figure 1B).

The scope of peptide stapling techniques has expanded from Grubbs metathesis,^20,26^ through the reaction of two cysteines^27^ to peptide macrolactamization.^28^ These peptide stapling strategies can rely on easily accessible modified or completely synthetic amino acids, but can also take advantage of the variety of functional groups present in the side chains of natural amino acids.

The diversity of functional groups in glycans, on the other hand, is minimal, with simple monosaccharides containing just primary and secondary alcohol groups. Therefore, when designing glycan stapling strategies, it is necessary to functionalize carbohydrate building blocks (BBs) for stapling following the glycan backbone construction by automated glycan assembly (AGA).^7^ The handles installed on the BB need to withstand acidic glycosylation conditions, basic conditions for temporary protecting group cleavage, and upon stapling form a macrocycle stable enough to survive methanolysis of esters and hydrogenolysis of benzyl ethers, the harsh reaction conditions used to remove the two types of permanent protecting groups.

Inspired by peptide macrolactamization,^28^ we designed two glucose BBs: one with a protected amine group in place of C6-OH and one with a protected carboxylic acid attached to C6-OH (Figure 2). The C6 position was chosen for the introduction of modifications, as it points out of the glucan turn. Thereby, after the glycan backbone is assembled by AGA, the peptide chain can be grown from the amine group by solid-phase peptide synthesis (SPPS) prior to glycan stapling by macrolactamization. This strategy can use the pool of amino acids, allowing for wide modularity of the linker, including its length, polarity, and even branching. Using glycan array analysis, we proved that stapled glycans bind significantly more strongly to anti-glucan antibodies than to linear, unmodified oligosaccharides.

## Results and Discussion

### AGA of β-Glucan Backbone

The β-glucan backbone was constructed by automated glycan assembly on a 15 μmol scale using a home-built synthesizer.^29^ Merrifield resin functionalized with a photocleavable linker bearing 5-aminopentanol **1** was used to provide conjugation-ready glycans (Figure 2).^30^ When the free reducing end of the final glycan was desired, photocleavable linker **2** equipped with a 1,4-bis­(hydroxymethyl)­benzene spacer was used instead.^6^ Oligosaccharide backbone construction relied on repetitive cycles of acidic wash, glycosylation, and deprotection. All three thioglycoside BBs were activated using *N*-iodosuccinimide (NIS) and trifluoromethanesulfonic acid (TfOH), and glycosylation required a strictly controlled temperature profile −20 °C (10 min) → 0 °C (30 min). The design of glucose BBs relied on the strategy of two permanent protecting groups: benzoyl (Bz) esters on the C2-hydroxyl ensure β-selective glycosylation and benzyl (Bn) ethers on all unfunctionalized hydroxyl groups increase the reactivity of the BB. The C3-hydroxyl group was temporarily protected as a fluorenylmethoxycarbonate (Fmoc) group in anticipation of chain extension. Based on the AGA protecting group pattern, orthogonal protecting groups for the amine and carboxylic acid groups had to be selected. Allyl ester proved to be a suitable protecting group for the carboxylic acid that can be revealed using Pd­(PPh_3_)_4_ and PhSiH_3_. This BB was introduced last during AGA, such that the alkene does not interfere with the thioglycoside activation conditions (NIS, TfOH). Once the desired glycan is completed, Fmoc-carbamate (stable to triethylamine) is cleanly cleaved using piperidine. Fmoc-carbonate and Fmoc-carbamate are an ideal set of orthogonal temporary protecting groups for AGA, followed by SPPS. The linker AAs can then be installed using standard peptide coupling conditions based on azabenzotriazole tetramethyluronium hexafluorophosphate (HATU) and *N*-methylmorpholine (NMM).

**Figure 2:**
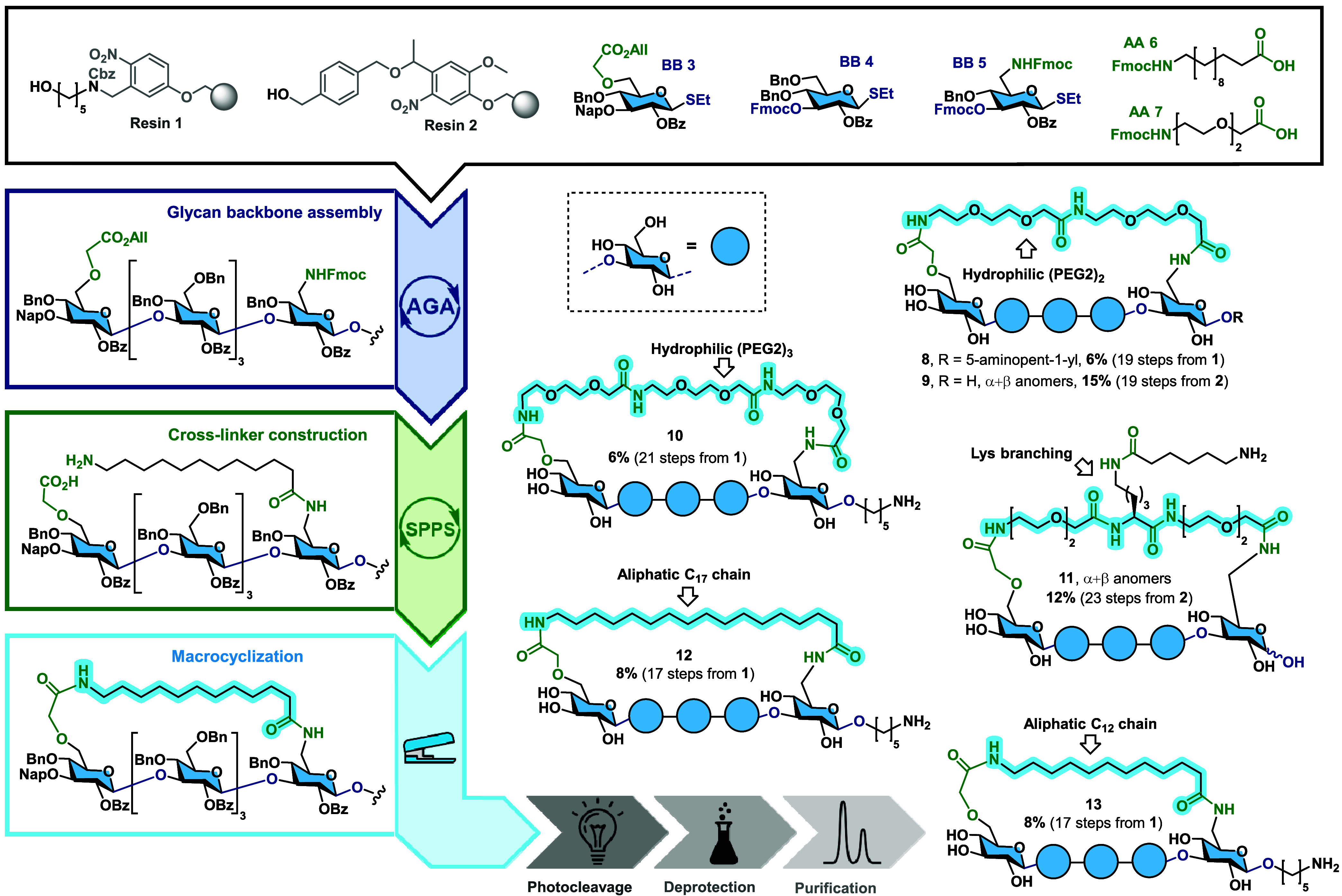
Synthesis of stapled β-glucans by AGA, SPPS, and macrolactamization. Schematic representation of the process: starting from three glucose BBs, the glycan is first assembled on resin **1** or **2** by AGA. The staple is then grown from the amine on the first glucose unit by SPPS. The terminal amine of the linker and carboxylic group of the last glucose unit are used to cyclize the molecule by macrolactamization, affording stapled glycans after deprotection and one final purification.

### On-Resin Macrolactamization

Once AGA had been established, macrocyclization was studied using the 12-aminododecanoic acid linker. Starting from resin **1** with a loading of 0.45 mmol/g used traditionally for AGA, the desired glycan was first assembled and the linker was attached. Following Fmoc and allyl group removal, the resin was thoroughly washed and dried *in vacuo* to remove any interfering amine. The cyclization was achieved using (7-azabenzotriazol-1-yloxy)­tripyrrolidinophosphonium hexafluorophosphate (PyAOP) and NMM in DMF, and the stapled glycan was observed by MALDI-TOF after photocleavage of a few resin beads. However, upon further deprotections, no product was isolated, possibly due to oligomerization side reactions, which compete with macrocyclization. In solution chemistry, high-dilution conditions or template effects favor macrocyclization over polymerization.^31^ During solid-phase synthesis, high dilution can be achieved using a low-loading resin. To increase the yield of glucan macrocyclization, a series of resins was prepared with loading ranging from 0.09 to 0.25 mmol/g by reacting Merrifield resin with limited amounts of the photocleavable linker and capping the remaining chloromethyl groups by reaction with cesium acetate (see the Supporting Information). The loading was determined by glycosylation with a mannose BB (see SI), followed by a spectrophotometric quantification of the Fmoc group.^32^ The isolated yields of the protected glycans stapled with a 12-carbon linker from the individual resins were determined.

With the 0.25 and 0.16 mmol/g loadings, no uncyclized side products, dimer, or trimer were observed, and the protected stapled glycan was obtained in 9 and 16% yield (over 15 steps starting from resin **1**), respectively. The lower resin loading required double glycosylation cycles to suppress deletion sequences, and loading lower than 0.16 mmol/g gave lower yields of the stapled glycan, probably due to difficulties related to mixing and mass transfer within the higher amount of resin. Therefore, we selected the two most promising resin loadings (0.25 and 0.16 mmol/g) to further explore different linker types and lengths.

Long-chain AAs of different polarities and lengths were selected as macrocyclization cross-linkers (Figure 2 and SI). Allyloxycarbonyl-protected 17-aminoheptadecanoic acid (prepared in one step from octadecanedioc acid) was used as the staple to provide higher flexibility (17 vs 12 carbon chain). The aliphatic hydrocarbon staples connecting the hydrophilic glycan render the final molecules amphiphilic. Aliphatic hydrocarbon staples have improved the pharmacological properties of peptides.^33^ In order to improve water solubility and avoid hydrophobic interactions with protein receptors, a hydrophilic poly­(ethylene glycol) (PEG) linker was used for glycan stapling. The glycans were stapled with two or three units of Fmoc-protected 8-amino-3,6-dioxaoctanoic acid as an SPPS-compatible PEG mimetic to provide glycans with lower or higher flexibility. Other supports such as resin **2** equipped with 1,4-bis­(hydroxymethyl)­benzene spacer and a loading of 0.25 mmol/g afforded, after deprotection, the desired stapled glucan **9** with the PEG linker and the free reducing end in 15% yield (over 19 steps starting from resin **2**).

The modularity of the stapling approach was explored by introducing l-lysine in the middle of the staple. The resulting stapled glycan can be conjugated while still preferentially displaying the central part of the molecule. During SPPS, l-lysine was introduced with a monomethoxytrityl-protected (Mmt) ε-amino group. The linear linker was first completed, then Mmt was cleaved and *N*-benzyloxycarbonyl-6-aminocaproic acid was attached to serve as a substitute for 5-aminopentanol used traditionally on the reducing end. Following stapling and deprotection, the stapled glycan was obtained in 12% yield.

In order to determine whether the properties of the stapled glycans arise from stapling alone or from the nature of the linker used for stapling, a collection of unstapled glycans containing the same linkers was prepared by executing the same synthetic sequence but omitting the macrocyclization step (Figure 3). The PEG-AA side chain of **15** was capped with acetic anhydride to prevent the terminal amine from competing with the 5-aminopentanol linker during conjugation.

**Figure 3:**
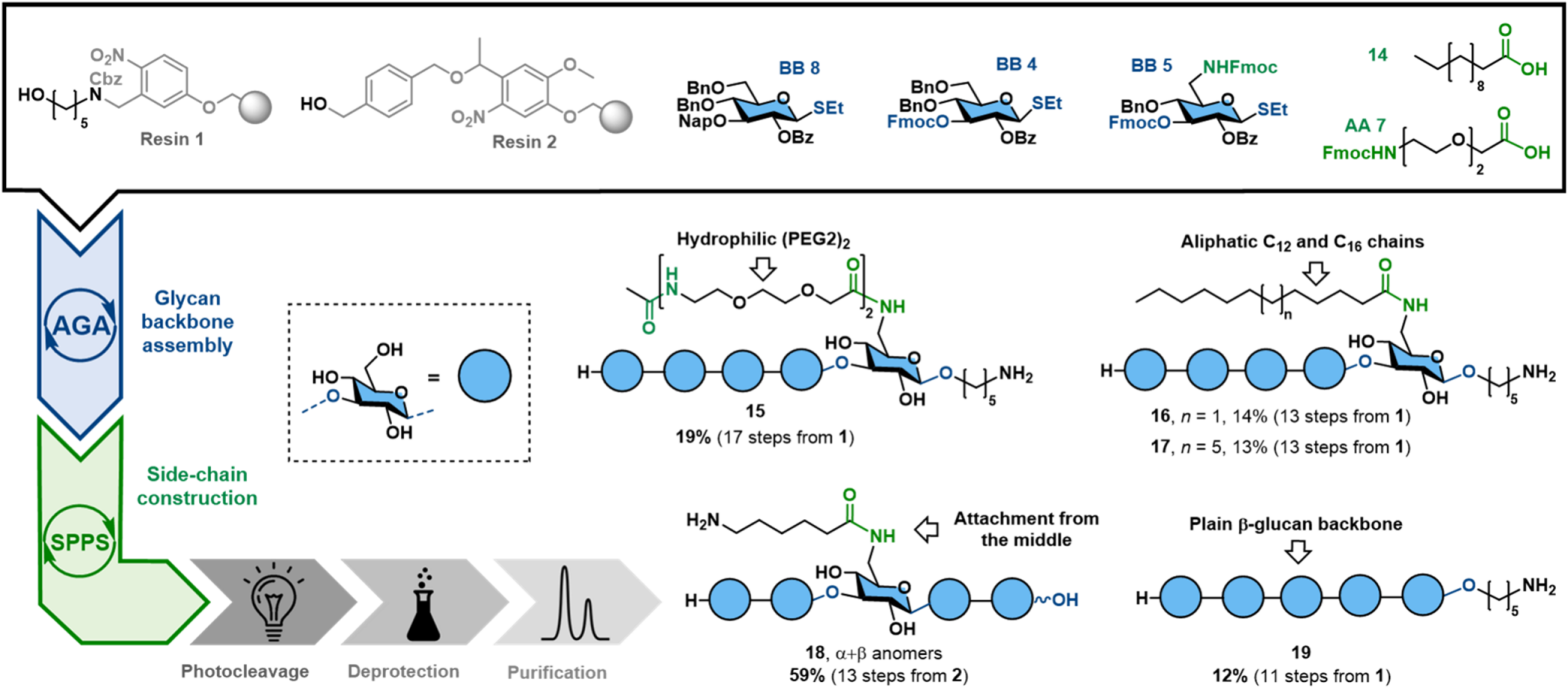
β-Glucans synthesized by AGA and SPPS were constructed like the stapled structures, but omitting the macrolactamization.

### Off-Resin Modifications

After the complete assembly of the glycans, the benzoyl groups were removed by on-resin methanolysis for branched glycans or hydrazinolysis in the case of linear β-glucan, followed by photocleavage in a flow setup. The cleaved and ester-free glycans were then subjected to hydrogenolysis to provide the crude target glycans. As expected, the non-glycan moieties introduced as cross-linkers remained intact under these conditions. A single purification afforded the stapled glycans in 6–15% yields.

### Molecular Dynamics (MD) Simulations of Stapled and Linear β-Glucans

Atomistic MD simulations of both linear and stapled β-(1,3)-glucans were performed to study the effect of stapling on the glycan conformation. In these simulations, the glycan part is modeled with the force field GLYCAM06_OSMOr14_ ^34,35^ and the side chains with the general AMBER force field (GAFF)^36^ (see SI for further details). Equilibrium end-to-end distances of the two terminal C6-hydroxyl groups of the glycans determined from simulation trajectories with a total length of 25 μs for each system provided insights concerning the compactness. The unmodified β-glucan pentamer mainly adopts extended conformations, leading to a maximum end-to-end distance probability at about 17 Å (dashed gray lines in Figure 4), around 4 Å above the value of the triple-helical curdlan. The presence of unstapled aliphatic or PEG side chains did not significantly influence the distribution when compared to the unmodified glucan. Analyses of snapshots indicate that the side chains point away from the glycan and do not interact with it (left snapshots in Figure 4). The C6-hydroxyl in β-glucan points out of the potential helix such that these compounds do not form inclusion complexes unlike amylose.^37^ On the other hand, stapling has a pronounced effect on the end-to-end distance distribution. All stapled structures develop second maxima on the curve at around 10 Å when the glycans adopt a curved conformation (right snapshots in Figure 4), while higher end-to-end distances decrease in population. The simulations demonstrate the potential of stapling for enriching compact, curved glucan conformations.

**Figure 4:**
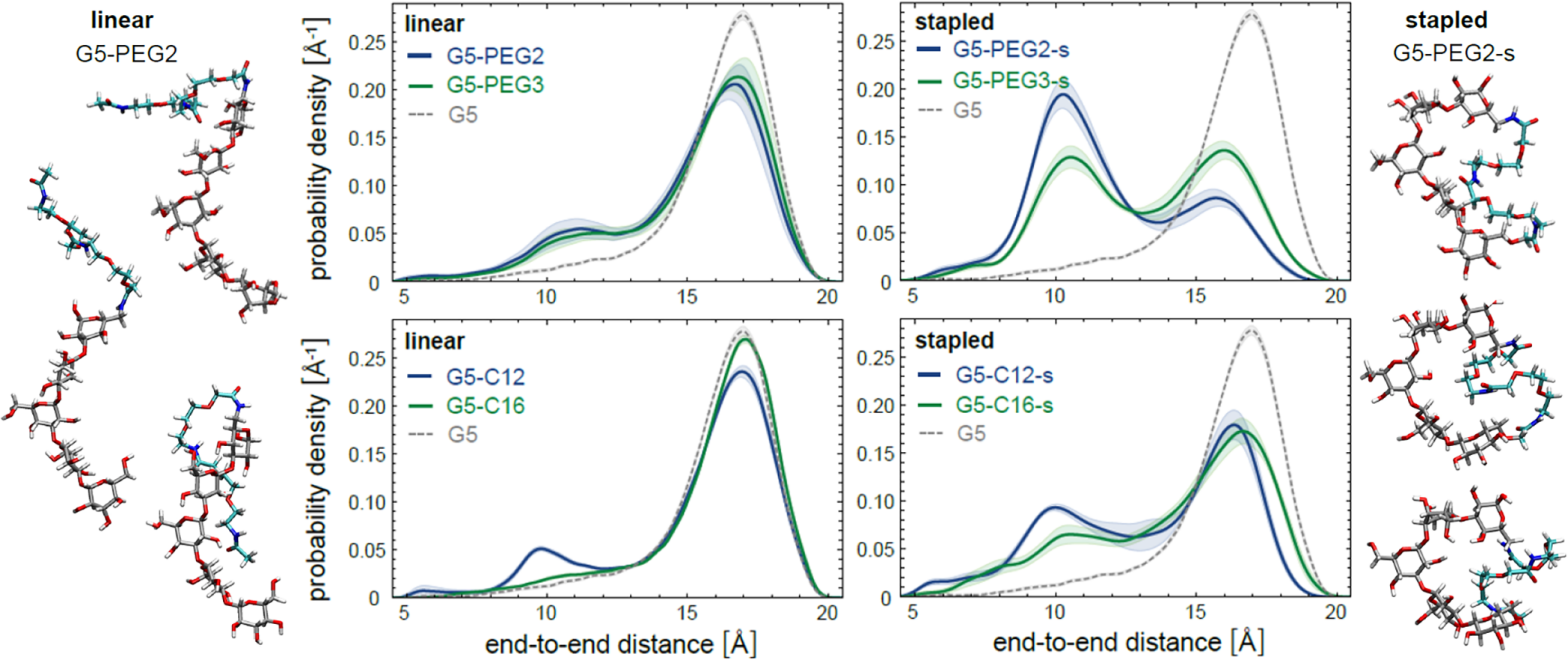
MD simulations of linear and stapled β-glucan oligosaccharides. Left and right: Snapshots of linear and stapled glucans with a (PEG2)­2 side chain. Center: Probability density distributions of end-to-end distance for opened and stapled β-glucans bearing a 12-carbon (C12), 16-carbon (C16), or two (PEG2) and three (PEG3) 8-amino-3,6-dioxaoctanoic acid units side chain. The end-to-end distance distribution of an unmodified β-glucan pentamer (G5) is included for comparison. Shaded regions indicate standard errors of the mean determined from distributions for five independent data sets of trajectories with a length of five microseconds.

### Antibody Binding

Short synthetic β-glucan oligosaccharides are poorly bound by anti-β-glucan antibodies. Likely, binding is length-dependent, as has been observed for binding of Dectin-1 to β-glucan oligosaccharides.^38^

Stapling peptides in the recognized conformation improves the affinity toward their receptors. In order to investigate whether the same is true for the interaction of β-(1,3)-glucans with anti-β-glucan antibodies, we screened the interaction of two β-glucan antibodies, a rabbit monoclonal (IgG1, 8201) and mouse monoclonal β-glucan Ab (IgG2b, 2G8), using glycan microarray experiments.

Synthetic β-glucans were covalently coupled to *N*-hydroxysuccinimide (NHS) ester-coated glass slides.^39^ These slides were then incubated with anti-β-glucan Ab. Binding was analyzed by incubating the slides with fluorescently labeled secondary antibodies, followed by fluorescence measurements. Stapled structures were significantly stronger bound by both antibodies when compared to the corresponding unstapled glycans (Figure 5). Interestingly, glucans stapled with aliphatic linkers in general and particularly **13** bind more strongly than the PEG-stapled counterparts to the mouse antibody. On the contrary, the rabbit antibody strongly prefers PEG-stapled glycans, underscoring that the length and nature of the staple need to be carefully studied when designing stapled glycans. Furthermore, glycan stapled with PEG-linker and attached to the glass slide from the middle of the staple (**11**) bound best to mouse Ab among the PEG-stapled glycans and the strongest to rabbit Ab. Glycan presentation plays an important role in Ab binding and should be investigated in more detail. Our findings highlight the importance of glycan conformation for protein recognition and demonstrate that glycan stapling is a powerful tool to tune glycan conformational space.

**Figure 5:**
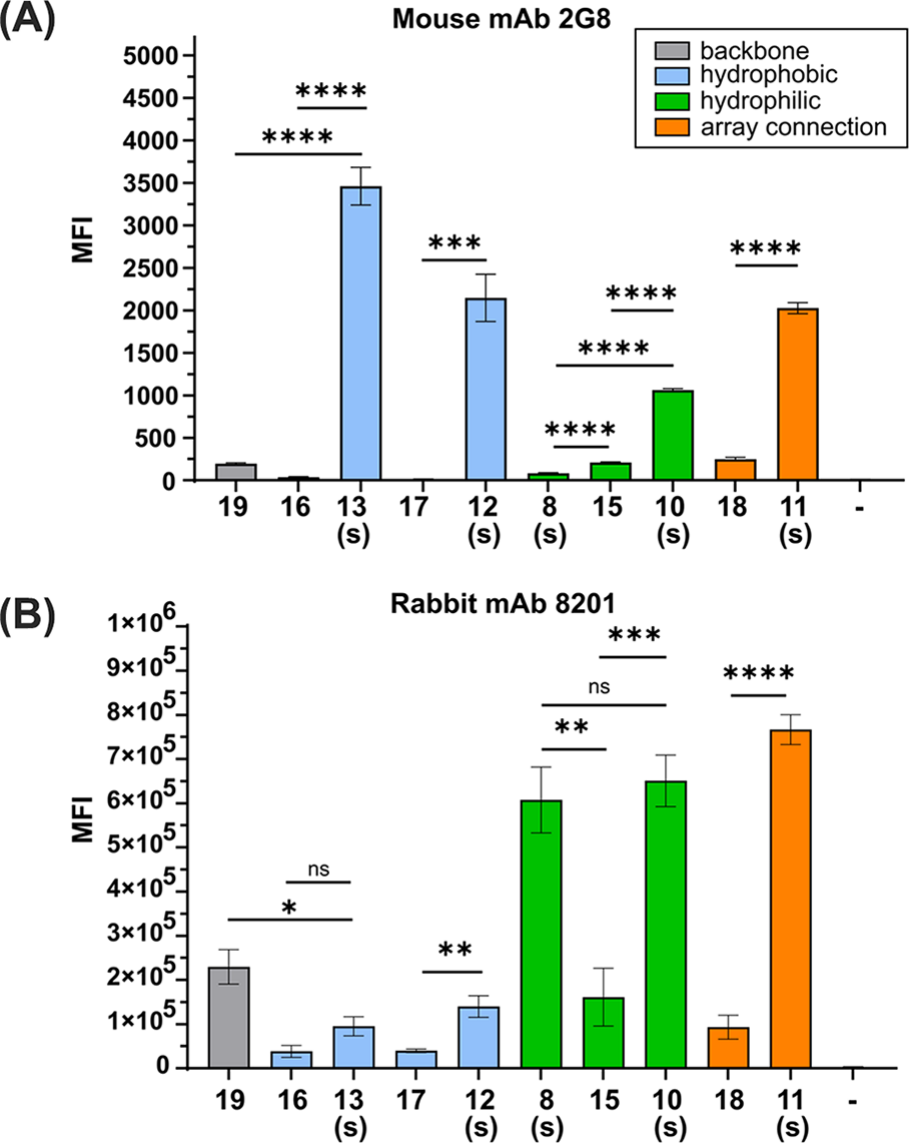
Glycan microarray analysis of antibody binding of synthetic β-glucan oligosaccharides. (−) Printing buffer. The letter “s” denotes stapled structures; for full structures, see Figures 2 and 3. (A) Mean fluorescence intensity of monoclonal antibody 2G8 binding to synthetic glycans. (B) Mean fluorescence intensity of monoclonal antibody 8201 binding to synthetic glycans. An antibody concentration of 10 μg/mL was used. Values represent mean ± SD. Differences were tested for significance using an unpaired *t*-test with Welch̀s correction with (****) *p* < 0.0001, (***) *p* < 0.001, (**) *p* < 0.01, (*) *p* < 0.05, and ns = not significant.

## Conclusions

We present a stapling strategy that enables access to glycans with a broad scope of staples, allowing for the use of hydrophobic aliphatic staples, PEG staples, or even lysine-branched staples for further modification. Glycan stapling changes the glycan conformational space as illustrated by MD simulations. Glycan array analysis revealed that conformational constraints can be used to significantly improve antibody binding. Glycan stapling underscores the importance of conformational studies in the glycosciences and its potential for the development of glycan-based vaccines.

## Supplementary Material



## Notes

Experimental section: all synthetic procedures, MD simulation details, glycan microarray printing and analysis, and characterization data including HPLC, HR-MS, and NMR (PDF)

## Abbreviations

AA: amino acid
Ab: antibody
AGA: automated glycan assembly
BB: building block
Cbz: benzyloxycarbonyl
HATU: azabenzotriazole tetramethyluronium hexafluorophosphate
NHS: *N*-hydroxysuccinimide
NIS: *N*-Iodosuccinimide
NMM: *N*-methylmorpholine
MD: molecular dynamics
Mmt: monomethoxytrityl
PEG: poly­(ethylene glycol)
RU: repeating unit
SPPS: solid-phase peptide synthesis
TfOH: trifluoromethanesulfonic acid
