# Supplementary material for: Stapling of β‑Glucans Increases Antibody Binding
Source: J Am Chem Soc. 2025 Oct 7;147(41):37634–40. doi: 10.1021/jacs.5c12690 (PMC12532290; doi:10.1021/jacs.5c12690)
Supplement: Supplementary file 1 [file ja5c12690_si_001.pdf]

SUPPORTING INFORMATION

Stapling of β-Glucans Increases Antibody Binding

Jiří Ledvinka,[⊥,§] Richard Kullmann,[⊥] Emelie E. Reuber,[⊥,§] Thomas Weikl,[⊥]
Manuel G. Ricardo,[⊥] Peter H. Seeberger*[,⊥,§]

Contents

1. General information 2
2. Abbreviations 2
3. Building block synthesis 3
4. Resin functionalization 27
5. Automated Glycan Assembly 28
   5.1. Preparation of reagent solutions 28
   5.2. Modules for Automated Solid-Phase Synthesis 28
   5.3. Post-AGA manipulations 31
   5.4. Post-solid-phase manipulations 34
   5.5. Oligosaccharide synthesis 36
      Synthesis and analytical data of **19** 36
      Synthesis and analytical data of **16** 41
      Synthesis and analytical data of **17** 45
      Synthesis and analytical data of **15** 50
      Synthesis and analytical data of **13** 54
      Synthesis and analytical data of **12** 59
      Synthesis and analytical data of **8** 64
      Synthesis and analytical data of **10** 69
      Synthesis and analytical data of **9** 74
      Synthesis and analytical data of **18** 78
      Synthesis and analytical data of **11** 83
6. Molecular dynamics simulations 88
   6.1. System setup 88
   6.2. Generation and analysis of simulation data 88
7. Glycan array analysis 89

1. General information

All chemicals used were reagent grade and used as supplied unless otherwise noted. The automated glycan syntheses were performed on home-built synthesizer developed at the Max Planck Institute of Colloids and Interfaces.[1] Analytical thin-layer chromatography (TLC) was performed on Merck silica gel 60 F254 plates (0.25 mm). Compounds were visualized by UV irradiation or dipping the plate in a staining solution (sugar stain: 10% H_2SO_4 in EtOH; CAM: 48 g/L ammonium molybdate, 60 g/L ceric ammonium molybdate in 6% H_2SO_4 aqueous solution). Flash column chromatography was carried out by using the forced flow of the indicated solvent on Fluka Kieselgel 60 M (0.04 – 0.063 mm). Analysis and purification by normal and reverse-phase HPLC were performed by using an Agilent 1200 series. Products were lyophilized using a Christ Alpha 2-4 LD plus freeze dryer. 1H, 13C, and HSQC NMR spectra were recorded on a Varian 400-MR (400 MHz), Varian 600-MR (600 MHz), or Bruker Biospin AVANCE700 (700 MHz) spectrometer. Spectra were recorded by using the solvent residual peak chemical shift as the internal standard ($CDCl_3$: 7.26 ppm ^{1}H, 77.0 ppm ^{13}C; D_2O: 4.79 ppm ^{1}H; CD_3OD: 3.31 ppm ^{1}H, 49.0 ppm ^{13}C or in D_2O/CD_3CN = 3/2 (*V*/*V*) using D_2O as a reference). High-resolution mass spectra were acquired using a 6210 ESI-TOF mass spectrometer (Agilent) and a MALDI-TOF autoflexTM (Bruker).

2. Abbreviations

AA, amino acid; AGA, automated glycan assembly; All, allyl; BB, building block; Bn, benzyl; Bz, benzoyl; Cbz, benzyloxycarbonyl; DCM, dichloromethane; DDQ, 2,3-dichloro-5,6-dicyano-1,4-benzoquinone; Et, ethyl; Ph, phenyl; DIPEA, *N*,*N*-diisopropylethylamine; DMAP, 4-(dimethylamino)pyridine; DMF, *N*,*N*-dimethylformamide; Nap, 2-naphthylmethyl; Fmoc, fluorenylmethoxycarbonyl; SPPS, solid-phase peptide synthesis; THF, tetrahydrofuran; SM, starting material; Ts, tosyl.

[1] Pardo-Vargas, A.; Delbianco, M.; Seeberger, P.H. *Curr. Opin. Chem. Biol.* **2018**, *46*, 48−55.

3. Building block synthesis

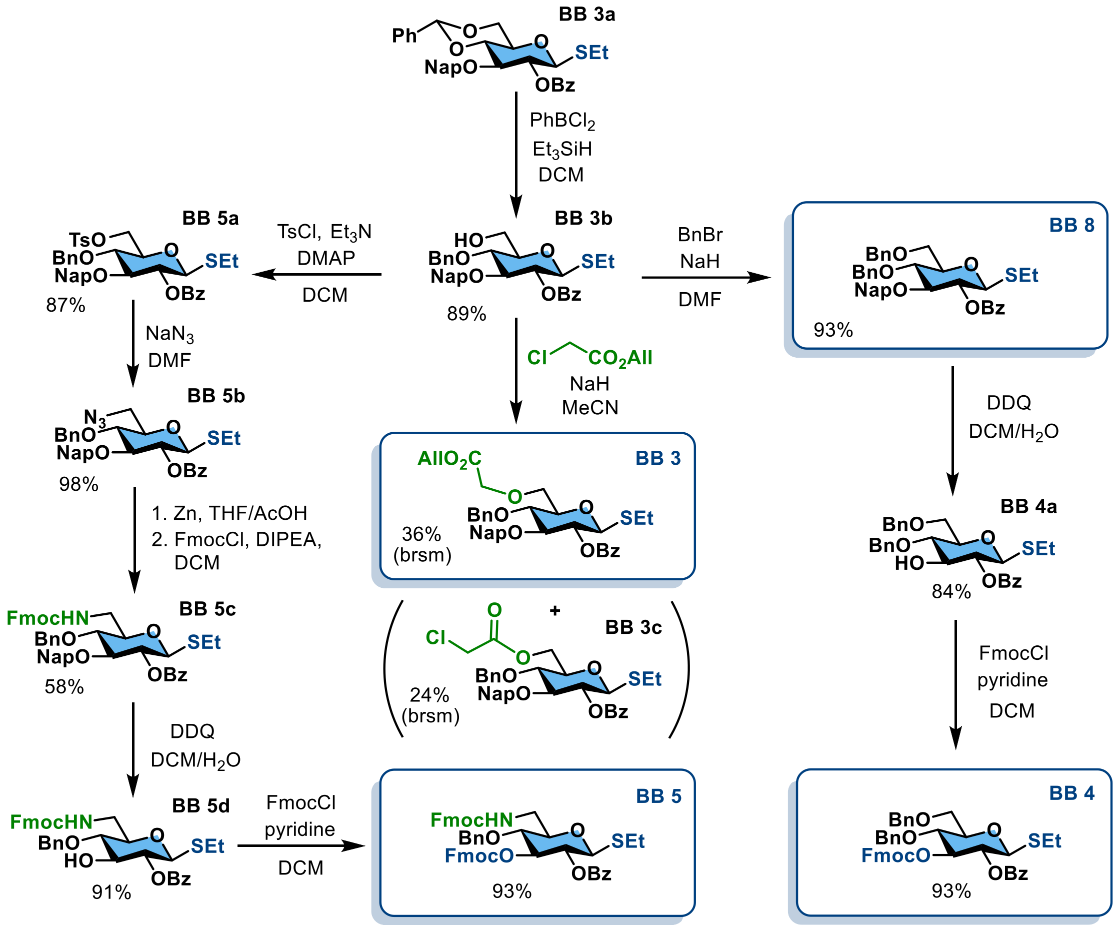

3.1. Ethyl 2-*O*-benzoyl-3-*O*-(2-naphthyl)methyl-4-*O*-benzyl-1-thio-β-D-glucopyranoside

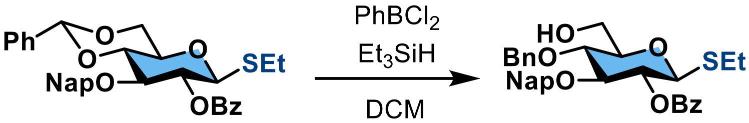

Anhydrous **BB 3a** (20.0 g, 35.9 mmol, 1.0 equiv.) and freshly dried powdered molecular sieves (4Å) were covered with anhydrous DCM (300 mL). The mixture was stirred for 30 minutes at r.t. and was then cooled to -78 °C. Triethylsilane (11.4 mL, 71.8 mmol, 2.0 equiv.) was added followed by addition of dichlorophenylborane (9.4 mL, 71.8 mmol, 2.0 equiv.). After 15 minutes of stirring at -78 °C, TLC (EA/hex = 1/3) indicated full conversion of the SM. The still cool reaction mixture was quenched with MeOH (20 mL), then triethylamine was added till neutral pH (ca. 25 mL). The mixture was warmed to r.t. and filtered through cellite. The filtrate was washed with sat. $NaHCO_3$ (150 mL) and brine (150 mL), dried with sodium sulfate, filtered and concentrated in vacuo. Flash chromatography of the crude in 0 to 15% EtOAc in hex/DCM = 1/1 afforded the product (17.8 g, 89% yield) as a white foamy solid. R*f* (hex/EA 3:1) = 0.32.

^{1}H NMR (400 MHz, $CDCl_3$) δ 7.98 – 7.90 (m, 2H), 7.72 – 7.64 (m, 1H), 7.63 – 7.58 (m, 1H), 7.57 – 7.49 (m, 3H), 7.44 – 7.28 (m, 9H), 7.23 (dd, *J* = 8.4, 1.7 Hz, 1H), 5.30 (dd, *J* = 10.0, 9.1 Hz, 1H), 4.92 (dd, *J* = 11.2, 9.0 Hz, 2H), 4.83 (d, *J* = 11.3 Hz, 1H), 4.71 (d, *J* = 10.9 Hz, 1H), 4.57 (d, *J* = 10.0 Hz, 1H), 3.98 – 3.87 (m, 2H), 3.80 – 3.71 (m, 2H), 3.50 (ddd, *J* = 9.7, 4.6, 2.6 Hz, 1H), 2.70 (qd, *J* = 7.5, 2.8 Hz, 2H), 1.22 (t, *J* = 7.4 Hz, 3H). ^{13}C NMR (101 MHz, $CDCl_3$) δ 165.41, 137.85, 135.28, 133.32, 133.18, 133.00, 129.88, 129.76, 128.70, 128.47, 128.29, 128.26, 128.19, 127.97, 127.76, 126.97, 126.15, 126.08, 125.94, 84.06, 83.87, 79.82, 77.84, 75.48, 75.36, 72.41, 62.15, 24.29, 15.02.

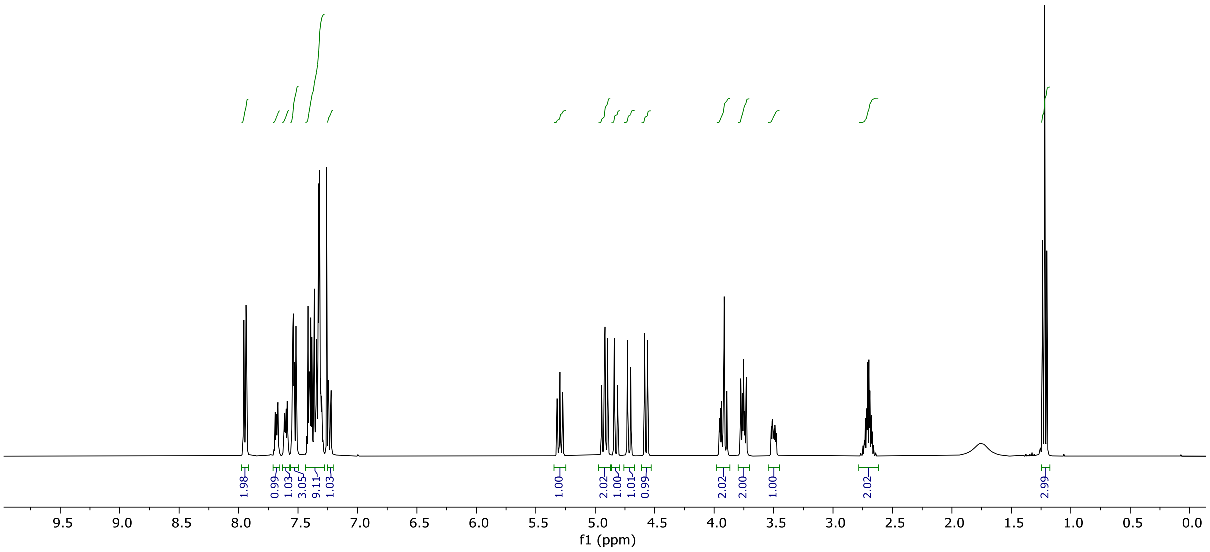

Figure S1. ^{1}H NMR (400 MHz, $CDCl_3$) spectrum of **3b**.

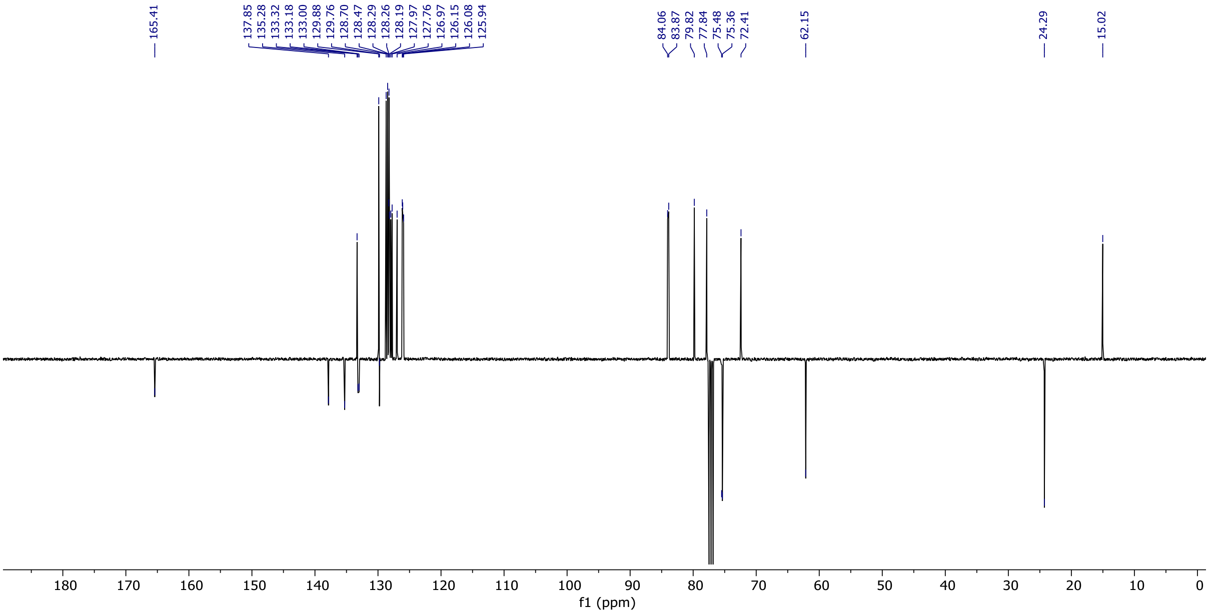

Figure S2. ^{13}C APT NMR (101 MHz, $CDCl_3$) spectrum of **3b**.

3.2. Ethyl 2-*O*-benzoyl-3-*O*-(2-naphthyl)methyl-4-*O*-benzyl-6-*O*-toluensulfonyl-1-thio-β-D-glucopyranoside

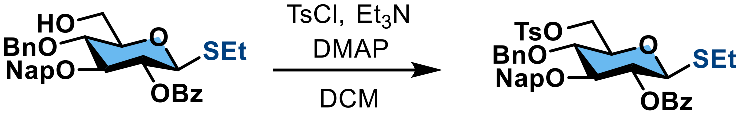

Compound **3b** (6.00 g, 10.7 mmol, 1.0 equiv.) was dissolved in anhydrous DCM (120 mL) under Ar. TsCl (8.16 g, 42.8 mmol, 4.0 equiv.) and DMAP (0.264 g, 2.14 mmol, 0.2 equiv.) were added followed by addition of triethylamine (3.0 mL, 21.4 mmol, 2.0 equiv.). The reaction mixture was stirred for five hours at r.t. After this time TLC indicated complete conversion (trace of SM). The reaction mixture was diluted with DCM, extracted with 10% aqueous citric acid (100 mL) and brine (100 mL). The organic layer was then dried with sodium sulfate, filtered and concentrated *in vacuo*. The crude was purified by column chromatography (0 to 5% EtOAc in hex/DCM = 2/1) giving product **5a** (5.54 g, 73% yield) as a white foamy solid. R*f* (hex/EA 3:1) = 0.55. ^{1}H NMR (400 MHz, $CDCl_3$) δ 7.96 – 7.89 (m, 2H), 7.83 – 7.75 (m, 2H), 7.72 – 7.64 (m, 1H), 7.63 – 7.57 (m, 1H), 7.56 – 7.48 (m, 3H), 7.44 – 7.28 (m, 9H), 7.25 – 7.19 (m, 3H), 5.25 (dd, J = 10.0, 9.1 Hz, 1H), 4.88 (t, J = 10.6 Hz, 2H), 4.80 (d, J = 11.3 Hz, 1H), 4.58 (d, J = 10.8 Hz, 1H), 4.48 (d, J = 10.0 Hz, 1H), 4.29 (dd, J = 10.6, 1.5 Hz, 1H), 4.16 (dd, J = 10.6, 4.5 Hz, 1H), 3.90 – 3.83 (m, 1H), 3.68 – 3.56 (m, 2H), 2.72 – 2.53 (m, 2H), 2.43 (s, 3H), 1.18 (t, J = 7.4 Hz, 3H). ^{13}C NMR (101 MHz, $CDCl_3$) δ 165.32, 145.10, 137.43, 135.07, 133.36, 133.17, 133.02, 132.76, 130.00, 129.86, 129.69, 129.33, 128.70, 128.48, 128.32, 128.25, 128.19, 127.97, 127.76, 126.97, 126.55, 126.12, 126.07, 126.00, 84.10, 83.57, 77.07, 75.52, 75.30, 72.08, 68.52, 24.00, 21.82, 15.05.

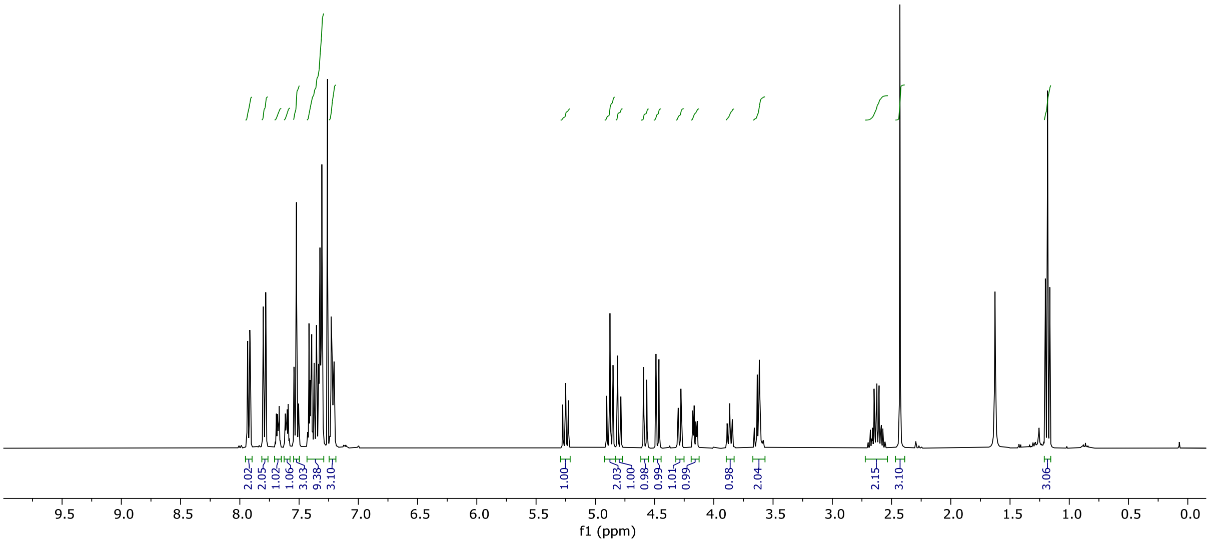

Figure S3. ^{1}H NMR (400 MHz, $CDCl_3$) spectrum of **5a**.

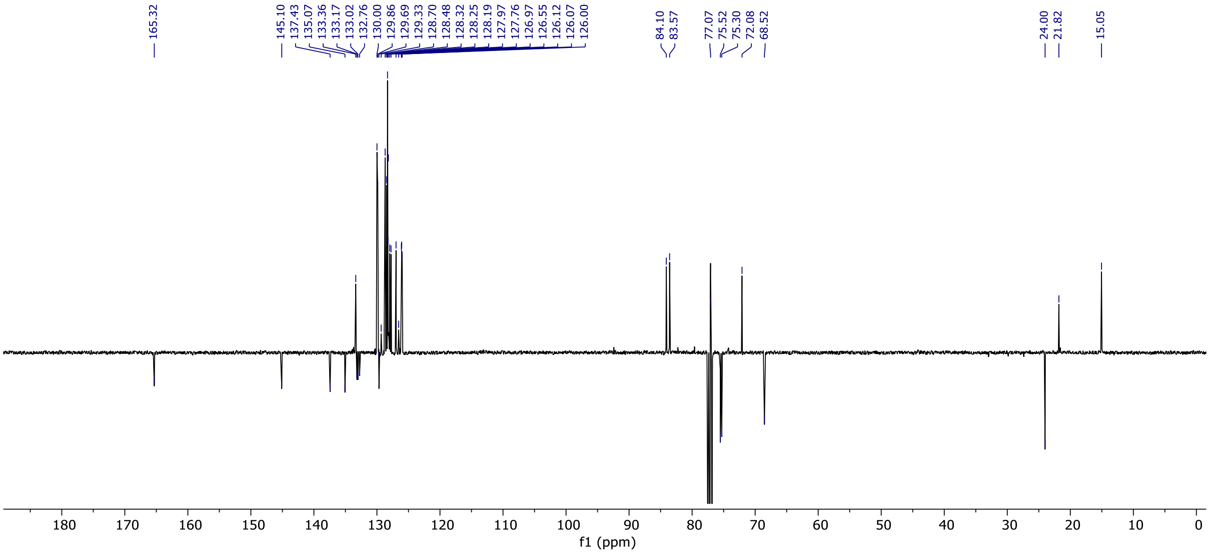

Figure S4. ^{13}C APT NMR (101 MHz, $CDCl_3$) spectrum of **5a**.

3.3. Ethyl 2-*O*-benzoyl-3-*O*-(2-naphthyl)methyl-4-*O*-benzyl-6-deoxy-6-azido-1-thio-β-D-glucopyranoside

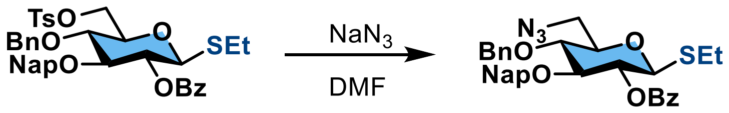

Compound **5a** (7.62 g, 10.7 mmol, 1.0 equiv.) was dissolved in anhydrous DMF (100 mL), sodium azide (2.71 g, 42.8 mmol, 4.0 equiv.) was added and the reaction mixture was stirred for 16 hours at 80 °C. The DMF was then removed at reduced pressure and the residue was partitioned between EtOAc (50 mL) and H_2O (50 mL). The organic layer was then washed with brine (50 mL), dried with sodium sulfate, filtered concentrated *in vacuo* yielding product **5b** (6.09 g, 98%) as an off-white solid. R*f* (hex/DCM/EA = 6/2/1) = 0.68. ^{1}H NMR (400 MHz, $CDCl_3$) δ 7.98 – 7.92 (m, 2H), 7.73 – 7.65 (m, 1H), 7.64 – 7.58 (m, 1H), 7.57 – 7.49 (m, 3H), 7.45 – 7.27 (m, 9H), 7.24 (dd, *J* = 8.5, 1.7 Hz, 1H), 5.34 (dd, *J* = 10.1, 9.1 Hz, 1H), 4.92 (dd, *J* = 11.1, 2.8 Hz, 2H), 4.82 (d, *J* = 11.3 Hz, 1H), 4.61 (dd, *J* = 35.3, 10.5 Hz, 2H), 3.90 (t, *J* = 8.9 Hz, 1H), 3.68 (dd, *J* = 9.6, 8.7 Hz, 1H), 3.63 – 3.51 (m, 2H), 3.36 (dd, *J* = 13.2, 5.7 Hz, 1H), 2.74 (qq, *J* = 12.0, 7.4 Hz, 2H), 1.23 (t, *J* = 7.5 Hz, 3H). ^{13}C NMR (101 MHz, $CDCl_3$) δ 165.36, 137.63, 135.14, 133.35, 133.18, 133.02, 129.89, 129.73, 128.72, 128.49, 128.33, 128.27, 128.22, 127.97, 127.77, 127.00, 126.12, 125.99, 84.13, 83.35, 79.03, 78.42, 75.55, 75.43, 72.22, 51.33, 23.54, 14.79.

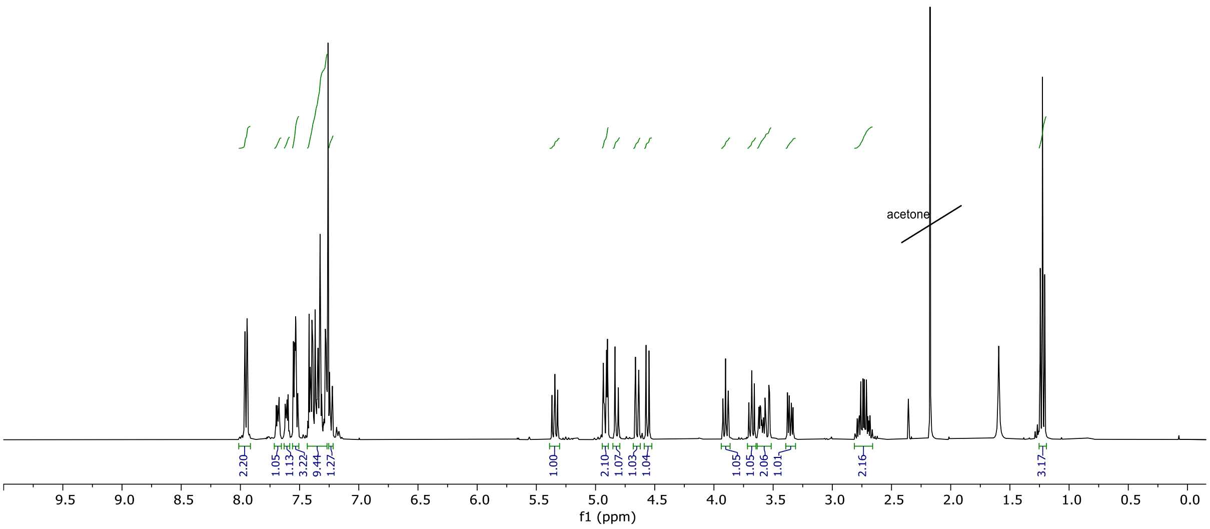

Figure S5. ^{1}H NMR (400 MHz, $CDCl_3$) spectrum of **5b**.

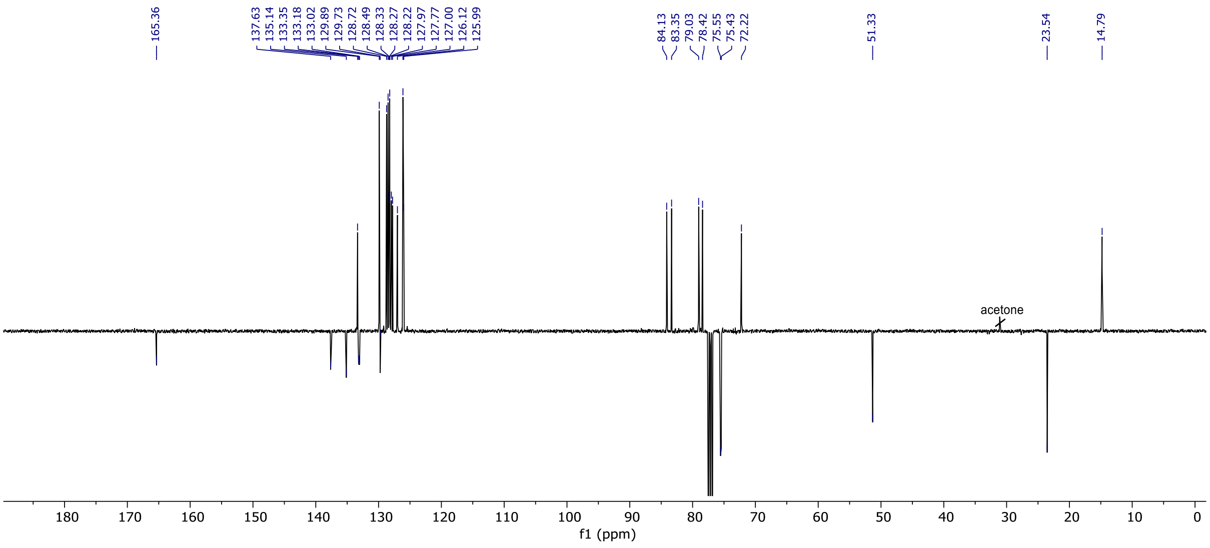

Figure S6. ^{13}C APT NMR (101 MHz, $CDCl_3$) spectrum of **5b**.

3.4. Ethyl 2-*O*-benzoyl-3-*O*-(2-naphthyl)methyl-4-*O*-benzyl-6-deoxy-6-*N*-(9-fluorenylmethoxycarbamido)-1-thio-β-D-glucopyranoside

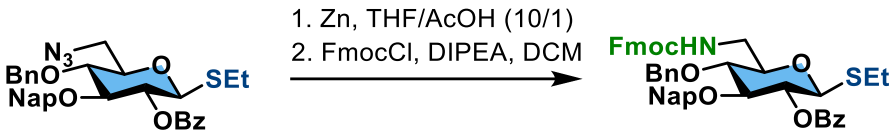

Compound **5b** (7.68 g, 13.2 mmol, 1.0 equiv.) was dissolved in dissolved in anhydrous THF (100 mL), then zinc dust (8.6 g, 132 mmol, 10 equiv.) was added under vigorous stirring and finally, AcOH (10 mL, 175 mmol, 13 equiv.) was added dropwise. After two hours of vigorous stirring, complete conversion of SM was observed by TLC/MS. The reaction mixture was filtered through cellite pad, which was then washed with DCM. The filtrate was then

evaporated to dryness *in vacuo*. The residue was dissolved in anhydrous DCM (100 mL) and DIPEA (14 mL, till basic pH) was added. After cooling to 0 °C, FmocCl (5.11 g, 19.7 mmol) was added. The reaction mixture was stirred for 30 minutes at 0 °C and then 30 minutes at r.t. The reaction mixture was then washed with 10% aqueous citric acid (2×100 mL) and brine (100 mL). The organic layer was then dried with sodium sulfate, filtered and concentrated *in vacuo*. The crude was purified by column chromatography (10% EtOAc in hex/DCM = 1/2) giving product **5c** (5.97 g, 58% yield) as a white solid. R*f* (hex/DCM/EA = 6/2/1) = 0.35. ^{1}H NMR (400 MHz, $CDCl_3$) δ 7.96 (dd, *J* = 8.3, 1.3 Hz, 2H), 7.81 – 7.74 (m, 2H), 7.73 – 7.65 (m, 1H), 7.65 – 7.58 (m, 3H), 7.57 – 7.50 (m, 3H), 7.45 – 7.28 (m, 8H), 7.26 – 7.22 (m, 1H), 5.29 (t, *J* = 9.6 Hz, 1H), 5.11 (t, *J* = 5.9 Hz, 1H), 4.93 (d, *J* = 11.2 Hz, 1H), 4.88 – 4.80 (m, 2H), 4.70 (d, *J* = 10.5 Hz, 1H), 4.54 (d, *J* = 10.0 Hz, 1H), 4.49 – 4.37 (m, 2H), 4.23 (t, *J* = 6.8 Hz, 1H), 3.90 (t, *J* = 8.4 Hz, 1H), 3.69 – 3.59 (m, 1H), 3.58 – 3.47 (m, 3H), 2.69 (q, *J* = 7.4 Hz, 2H), 1.23 (t, *J* = 7.5 Hz, 3H). ^{13}C NMR (101 MHz, $CDCl_3$) δ 165.42, 156.49, 144.03, 141.46, 137.75, 135.19, 133.36, 133.20, 133.02, 129.88, 129.74, 128.70, 128.58, 128.50, 128.32, 128.21, 127.98, 127.91, 127.84, 127.77, 127.24, 127.21, 126.98, 126.12, 125.98, 125.18, 125.15, 120.15, 84.21, 83.86, 78.41, 78.19, 75.59, 75.41, 72.34, 66.87, 47.38, 41.63, 24.38, 15.08.

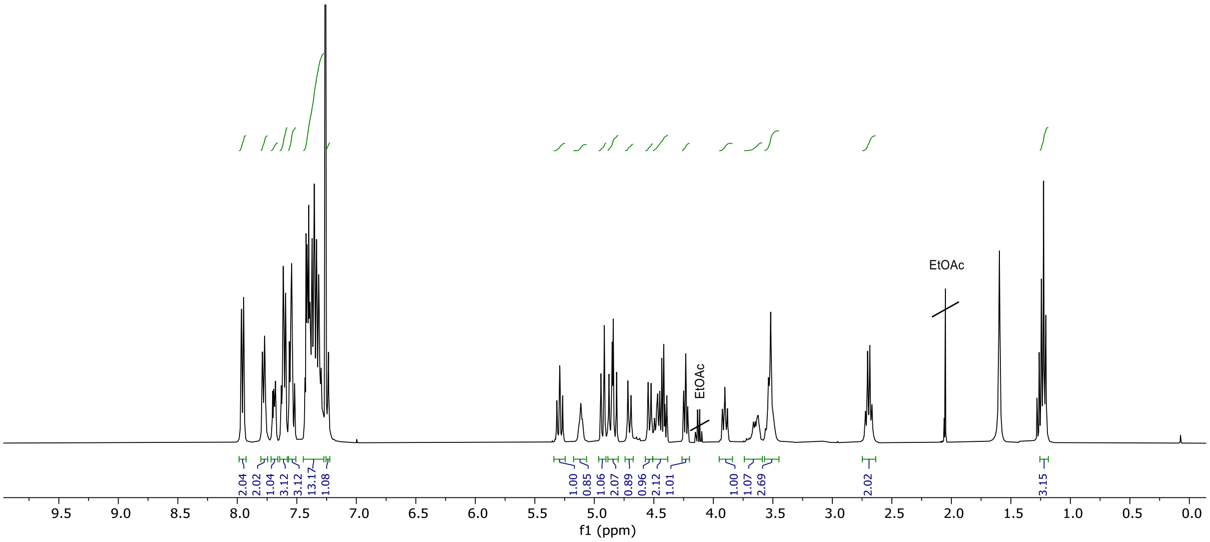

Figure S7. ^{1}H NMR (400 MHz, $CDCl_3$) spectrum of **5c**.

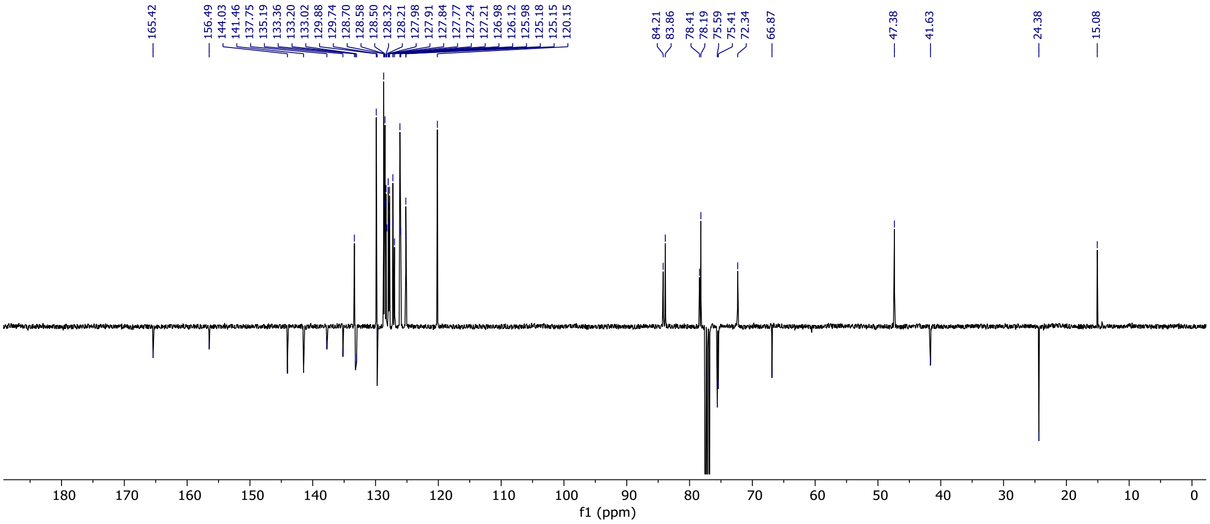

Figure S8. ^{13}C APT NMR (101 MHz, $CDCl_3$) spectrum of **5c**.

3.5. Ethyl 2-*O*-benzoyl-4-*O*-benzyl-6-deoxy-6-*N*-(9-fluorenylmethoxycarbamido)-1-thio-β-D-glucopyranoside

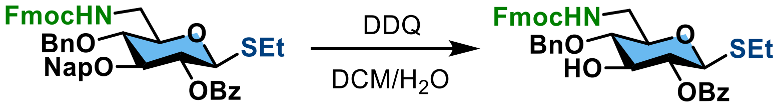

Compound **5c** (4.02 g, 5.16 mmol, 1.0 equiv.) was dissolved in DCM (50 mL) and water (5 mL) was added. DDQ (2.34 g, 10.3 mmol, 2.0 equiv.) was then added and the reaction mixture was stirred for one hour, upon which the mixture was diluted with DCM (50 mL) and more DDQ (0.30 g, 1.3 mmol, 0.3 equiv.) was added. After one hour, TLC indicated complete conversion of SM and the reaction mixture was quenched with sat. aqueous $NaHCO_3$ (100 mL) and sat. aqueous $Na_2S_2O_3$ (100 mL). The organic layer was separated, washed with sat. aqueous $NaHCO_3$ (100 mL), dried with sodium sulfate, filtered and concentrated *in vacuo*. The crude was purified by column chromatography (2 to 10% EA in hex/DCM = 1/1) giving product **5d** (2.99 g, 91% yield) as a white solid. R*f* (hex/DCM/EA = 2/2/1) = 0.60. ^1H NMR (400 MHz, $CDCl_3$) δ 8.11 – 8.04 (m, 2H), 7.82 – 7.75 (m, 2H), 7.64 – 7.55 (m, 3H), 7.50 – 7.26 (m, 12H), 5.12 (t, *J* = 5.8 Hz, 1H), 5.07 (t, *J* = 9.6 Hz, 1H), 4.83 (d, *J* = 10.9 Hz, 1H), 4.77 (d, *J* = 11.0 Hz, 1H), 4.57 (d, *J* = 10.0 Hz, 1H), 4.53 – 4.38 (m, 2H), 4.24 (t, *J* = 6.8 Hz, 1H), 3.96 (t, *J* = 8.8 Hz, 1H), 3.66 (dd, *J* = 11.4, 5.5 Hz, 1H), 3.56 – 3.44 (m, 2H), 3.39 (t, *J* = 8.9 Hz, 1H), 2.70 (qt, *J* = 7.5, 3.7 Hz, 2H), 2.60 (s, 1H), 1.25 (t, *J* = 7.4 Hz, 3H). ^{13}C NMR (101 MHz, $CDCl_3$) δ 166.34, 156.50, 144.02, 141.48, 137.90, 133.63, 130.12, 129.54, 128.78, 128.62, 128.30, 127.85, 127.23, 125.15, 120.17, 83.44, 78.68, 77.93, 77.33, 75.24, 73.31, 66.87, 47.38, 41.81, 24.46, 15.15.

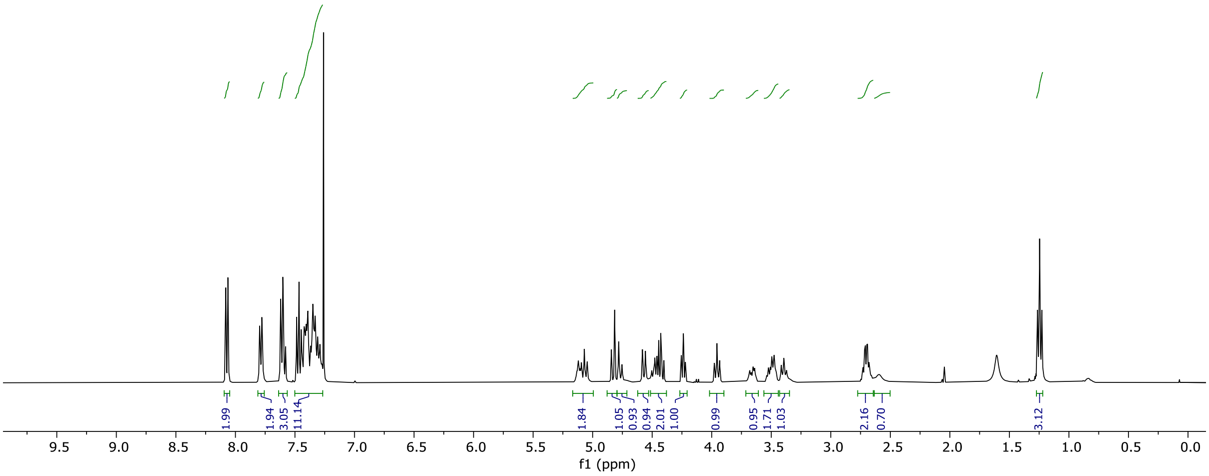

Figure S9. ^{1}H NMR (400 MHz, $CDCl_3$) spectrum of **5d**.

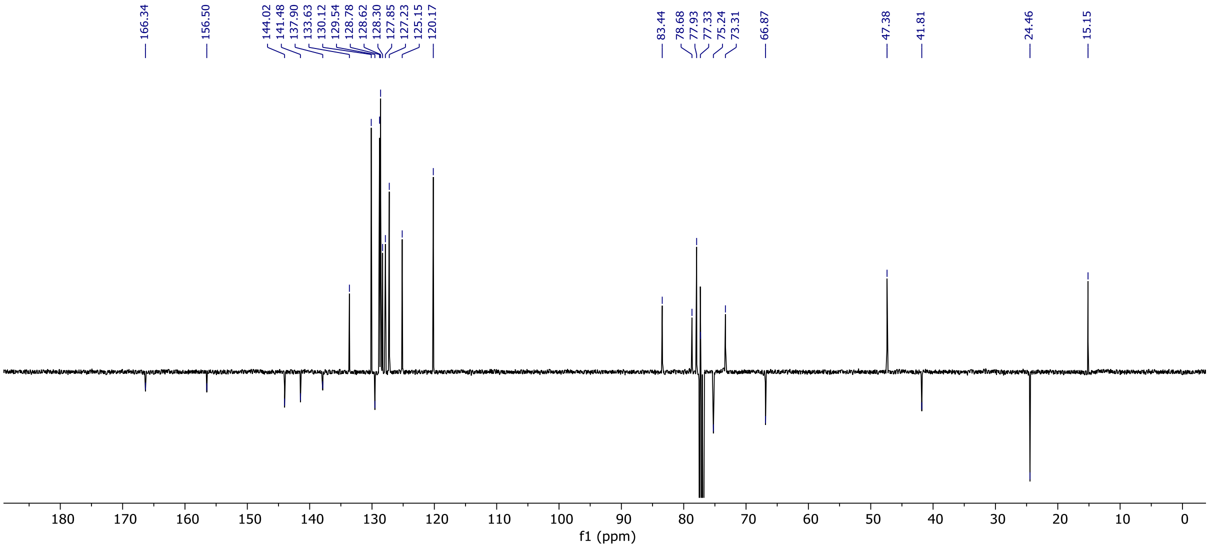

Figure S10. ^{13}C APT NMR (101 MHz, $CDCl_3$) spectrum of **5d**.

3.6. Ethyl 2-*O*-benzoyl-3-*O*-(9-fluorenylmethoxycarbonyl)-4-*O*-benzyl-6-deoxy-6-*N*-(9-fluorenylmethoxycarbamido)-1-thio-β-D-glucopyranoside (BB 5)

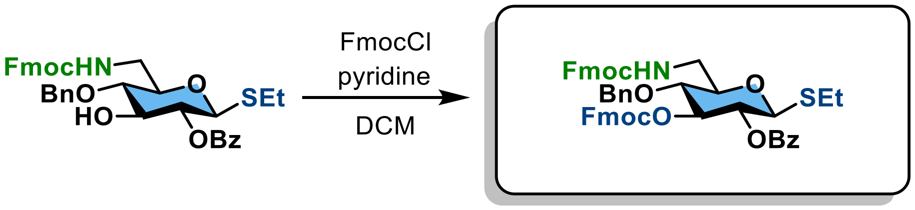

Compound **5d** (2.98 g, 4.66 mmol, 1.0 equiv.) was dissolved in anhydrous DCM (100 mL), pyridine (1.1 mL, 14 mmol, 3.0 equiv.) and FmocCl (1.81 g, 6.99 mmol, 1.5 equiv.) were added and the reaction mixture was stirred for three hours at r.t. TLC indicated completion after this time. The reaction mixture was then washed with brine (100 mL), it was dried with sodium

sulfate, filtered and concentrated *in vacuo*. The crude was purified by column chromatography (2 to 10% EtOAc in hex/DCM = 1/1) affording the product **5** (3.73 g, 93% yield) as a white solid. R*f* (hex/DCM/EA = 2/2/1) = 0.83. HR-MS *m/z* = 884.2917 $[M+Na]^+$, calculated for $C_{52}H_{47}NNaO_9S^+$: 884.2869. ^1H NMR (400 MHz, $CDCl_3$) δ 8.03 – 7.96 (m, 2H), 7.84 – 7.76 (m, 2H), 7.69 (ddt, *J* = 7.6, 4.2, 1.0 Hz, 2H), 7.62 (d, *J* = 7.4 Hz, 2H), 7.52 – 7.27 (m, 14H), 7.25 – 7.10 (m, 4H), 5.34 –f 5.22 (m, 2H), 5.10 – 5.02 (m, 1H), 4.69 – 4.58 (m, 2H), 4.54 (dd, *J* = 10.7, 6.8 Hz, 1H), 4.45 (dd, *J* = 10.7, 6.5 Hz, 1H), 4.25 (q, *J* = 4.9 Hz, 2H), 4.17 – 4.07 (m, 1H), 3.95 (t, *J* = 7.4 Hz, 1H), 3.57 (s, 4H), 2.71 (q, *J* = 7.4 Hz, 2H), 1.24 (t, *J* = 7.5 Hz, 3H). ^{13}C NMR (101 MHz, $CDCl_3$) δ 165.42, 156.48, 154.71, 144.04, 143.92, 143.45, 142.97, 141.51, 141.30, 141.19, 137.32, 133.53, 130.13, 129.21, 128.61, 128.51, 128.45, 128.19, 128.00, 127.93, 127.87, 127.26, 125.30, 125.12, 125.02, 120.19, 120.07, 120.05, 83.68, 80.71, 77.98, 76.16, 75.12, 70.77, 70.38, 66.80, 47.45, 46.55, 41.45, 24.59, 15.05.

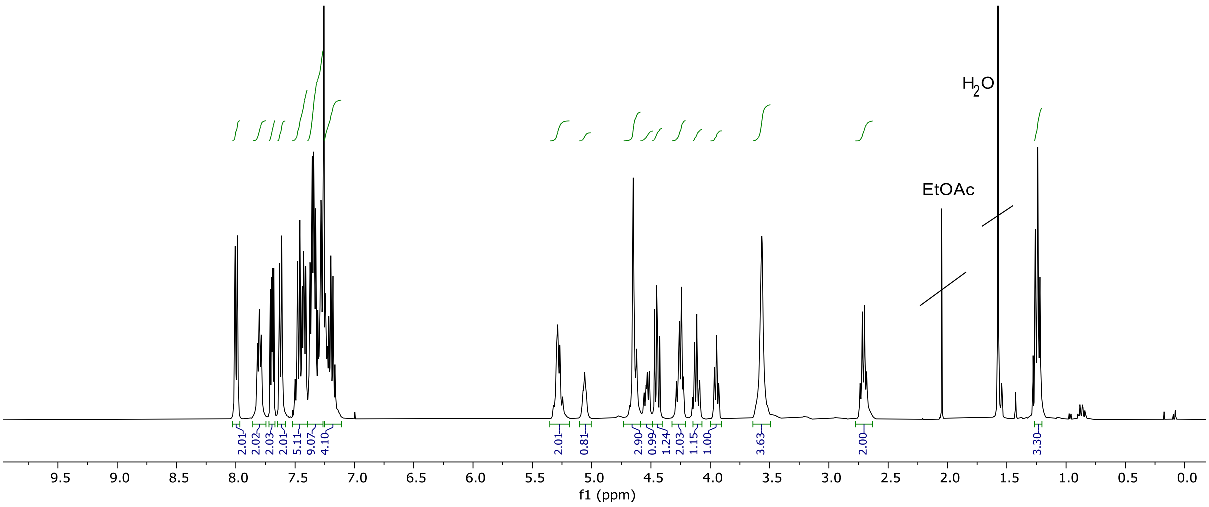

Figure S11. ^1H NMR (400 MHz, $CDCl_3$) spectrum of **BB 5**.

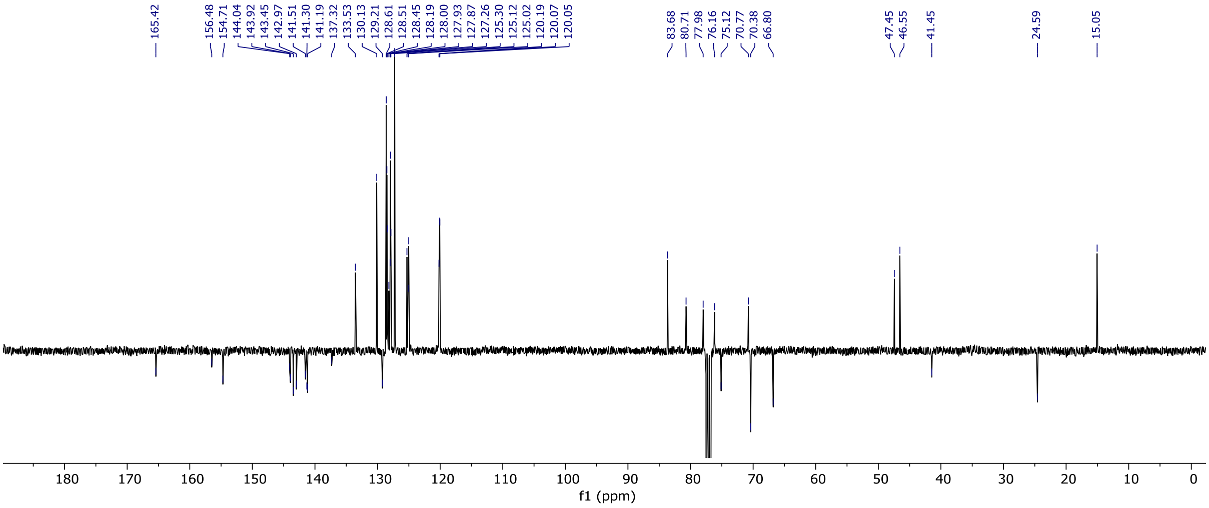

Figure S12. ^{13}C APT NMR (101 MHz, $CDCl_3$) spectrum of **BB 5**.

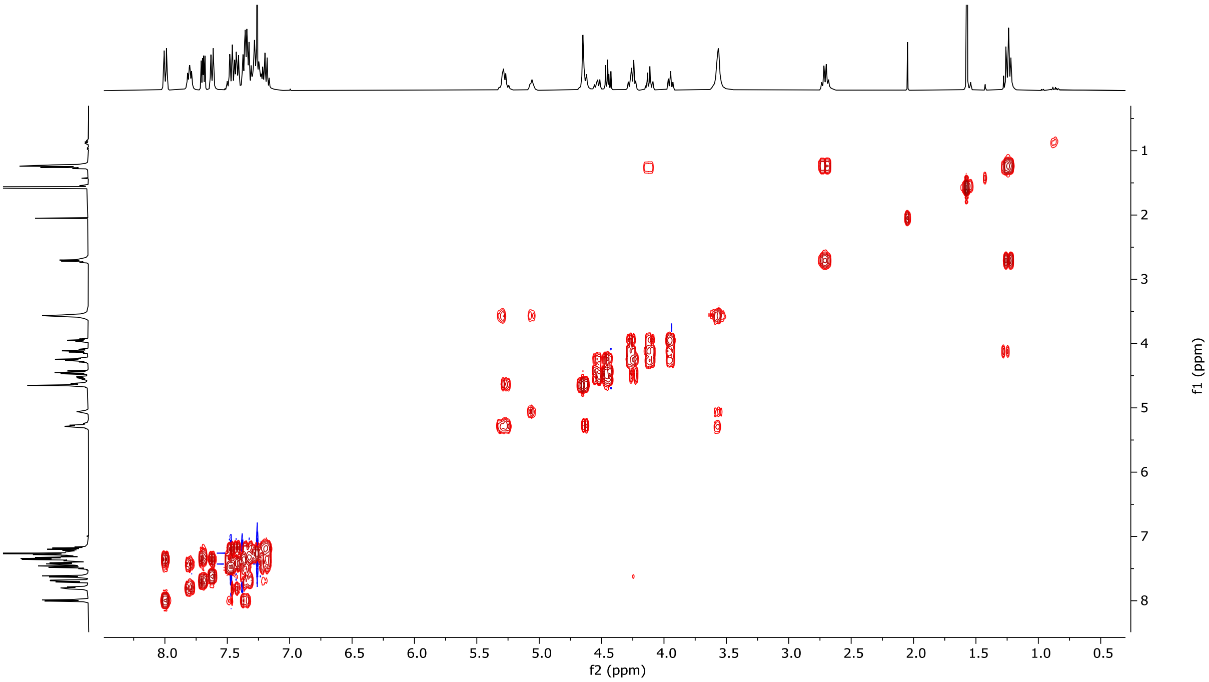

Figure S13. COSY NMR (400 MHz, $CDCl_3$) spectrum of **BB 5**.

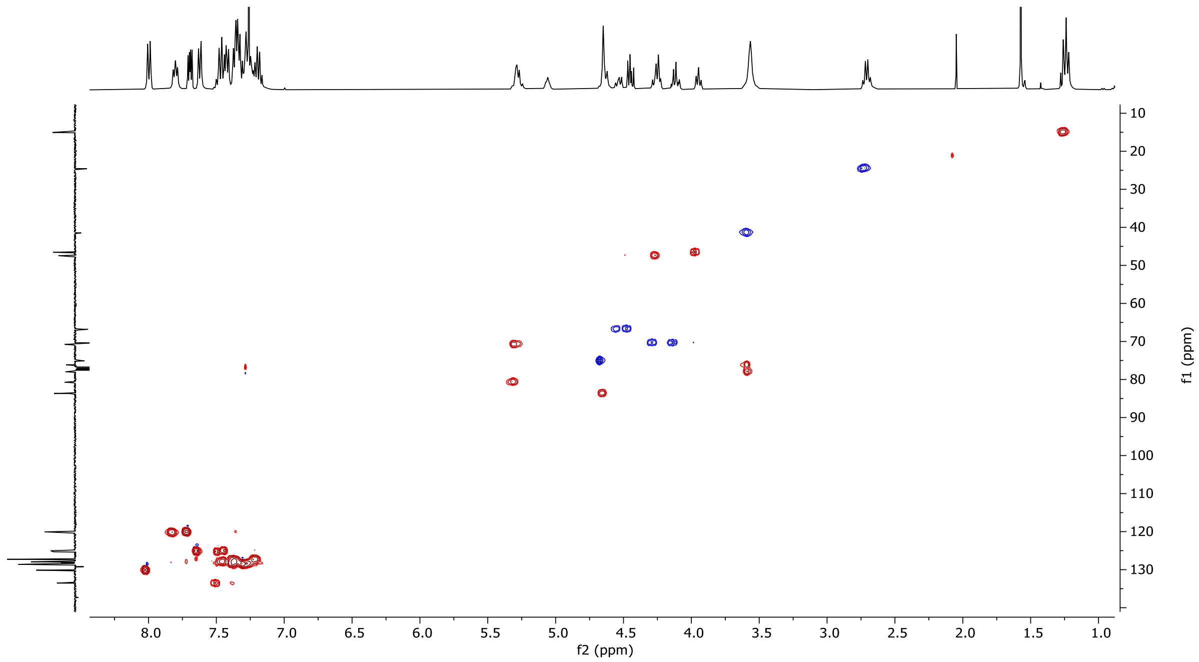

Figure S14. HSQC NMR (400MHz, CDCl3) spectrum of **BB 5**.

3.7. Ethyl 2-*O*-benzoyl-3-*O*-(2-naphthyl)methyl-4-*O*-benzyl-6-*O*-allylcarboxymethyl-1-thio-β-D-glucopyranoside (BB 3)

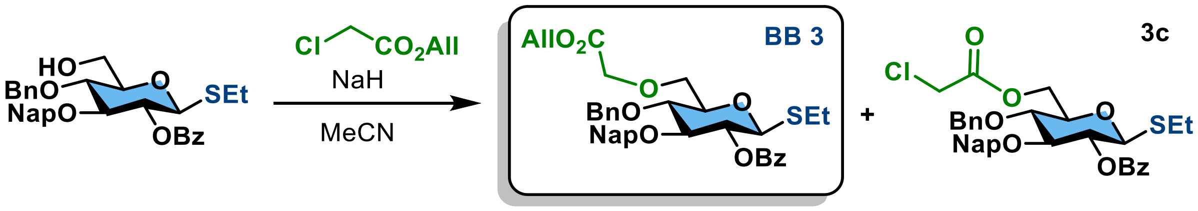

Sodium hydride (60% dispersion, 107 mg, 2.69 mmol, 1.5 equiv.) was washed three times with pentane and dried with stream of argon. **3b** (1.00 g, 1.79 mmol, 1.0 equiv.) was dissolved in anhydrous acetonitrile (20 mL) and added to the sodium hydride. This mixture was stirred for five minutes at r.t. followed by addition of allyl chloroacetate (623 µL, 5.37 mmol, 3.0 equiv.). The reaction mixture was stirred for one hour at r.t. and was then quenched with 10% aqueous citric acid. The mixture was diluted with DCM, the organic phase was separated, dried with sodium sulfate, filtered and concentrated *in vacuo*. The crude was purified by column chromatography (0 to 3% EtOAc in toluene to elute the product and then to 8% EtOAc to elute SM). This afforded the product **3** (302 mg, 36% based on recovered SM) as an off-white solidified oil along with unreacted **3b** (290 mg) and 6-*O*-chloroacetate side-product **3c** (192 mg, 24% yield based on recovered SM). Analytical data for **3**:

R*f* (10% EA in toluene) = 0.44. HR-MS *m/z* = 679.2365 [M+Na]$^+$, calculated for $C_{38}H_{40}NaO_8S^+$: 679.2336. ^{1}H NMR (400 MHz, $CDCl_3$) δ 7.98 – 7.89 (m, 2H), 7.72 – 7.63 (m, 1H), 7.62 – 7.56 (m, 1H), 7.55 – 7.49 (m, 3H), 7.43 – 7.27 (m, 9H), 7.23 (dd, *J* = 8.4, 1.7 Hz, 1H), 5.92 (ddt, *J* = 17.3, 10.4, 5.8 Hz, 1H), 5.39 – 5.21 (m, 3H), 4.91 (dd, *J* = 11.1, 8.9 Hz, 2H), 4.80 (dd, *J* = 13.2, 11.1 Hz, 2H), 4.66 (dt, *J* = 5.8, 1.4 Hz, 2H), 4.51 (d, *J* = 10.0 Hz, 1H), 4.32 – 4.17 (m, 2H), 3.95 – 3.83 (m, 3H), 3.79 (t, *J* = 9.3 Hz, 1H), 3.59 (ddd, *J* = 9.7, 4.1, 2.5 Hz, 1H), 2.78 – 2.62 (m, 2H), 1.22 (t, *J* = 7.4 Hz, 3H). ^{13}C NMR (101 MHz, $CDCl_3$) δ 170.22, 165.41, 138.11, 135.38, 133.25, 133.20, 133.00, 131.84, 129.87, 128.61, 128.44, 128.23, 128.02, 127.97, 127.75, 126.92, 126.15, 126.04, 125.89, 118.99, 84.20, 83.64, 79.76, 77.84, 75.44, 75.23, 72.30, 70.57, 69.31, 65.57, 23.99, 14.98.

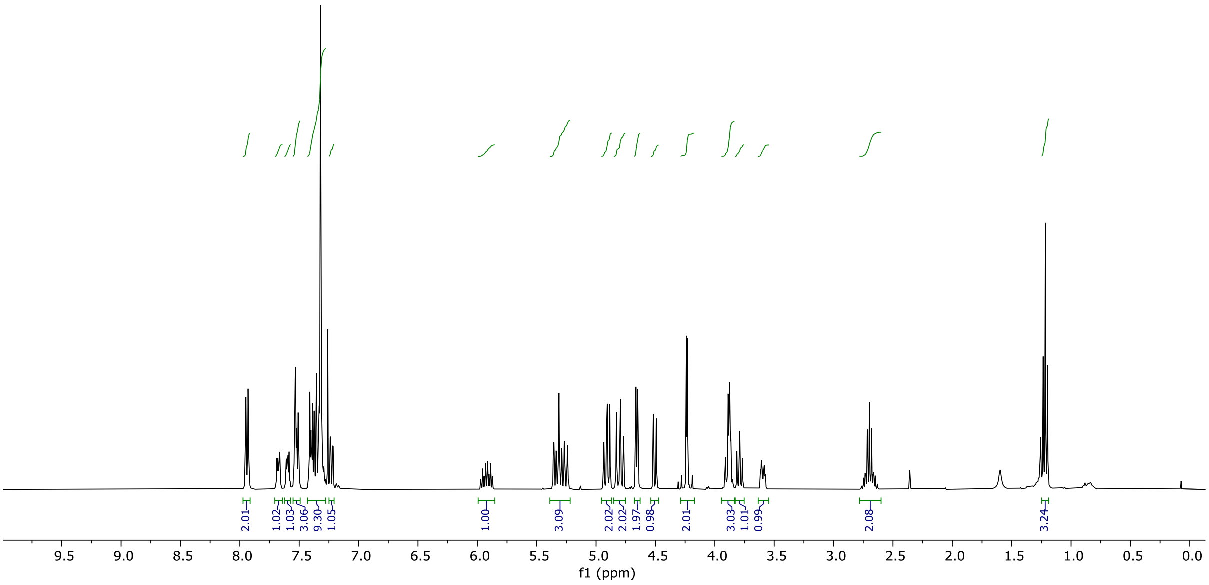

Figure S15. ^{1}H NMR (400 MHz, $CDCl_3$) spectrum of **3**.

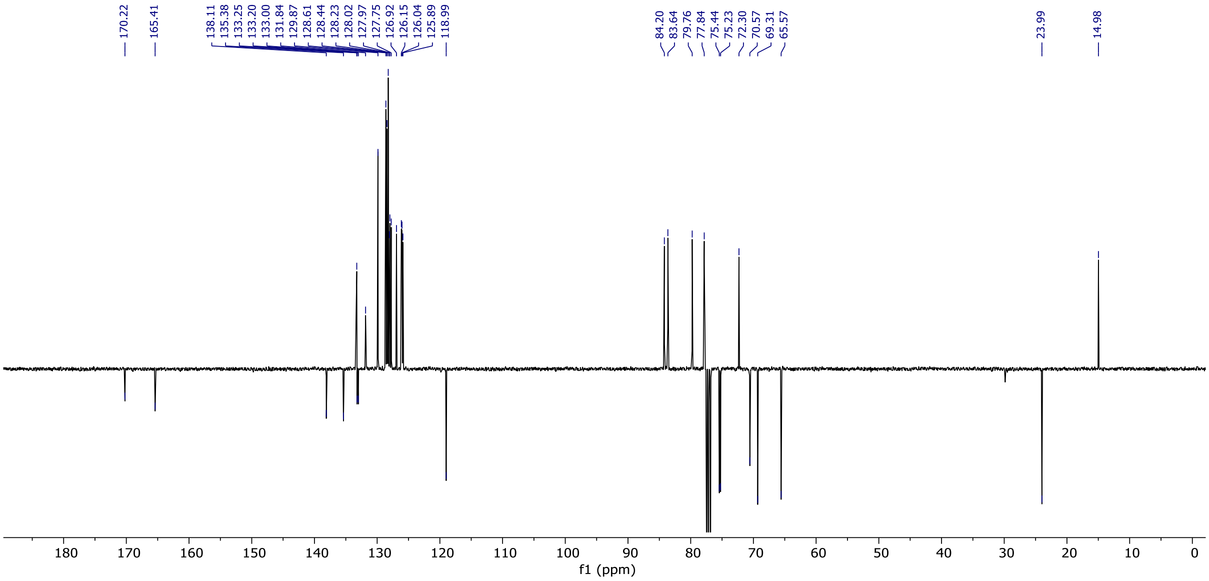

Figure S16. ^{13}C APT NMR (101 MHz, $CDCl_3$) spectrum of **3**.

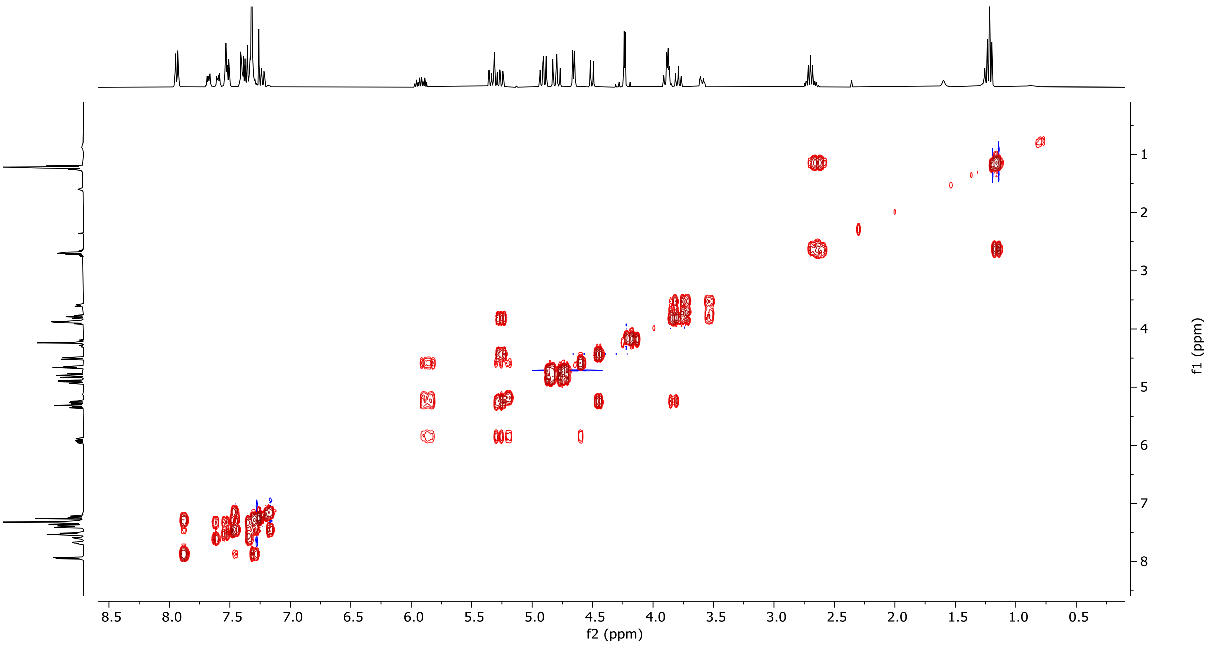

Figure S17. COSY NMR (400 MHz, $CDCl_3$) spectrum of **3**.

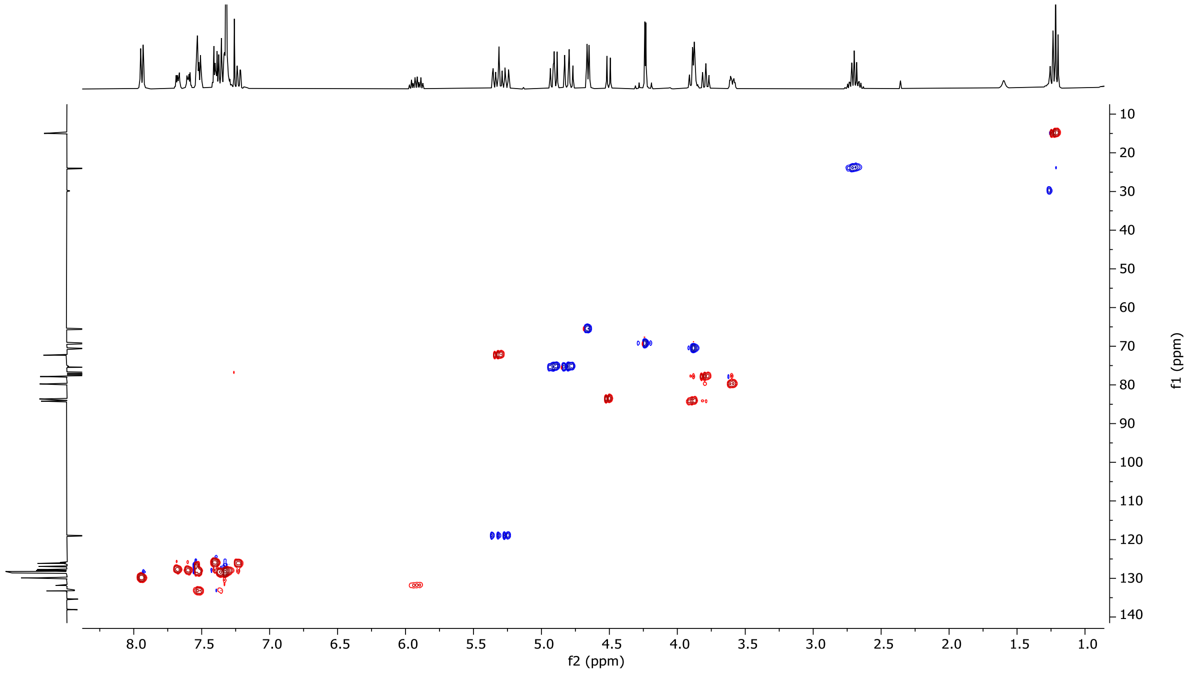

Figure S18. HSQC NMR (400MHz, $CDCl_3$) spectrum of **3**.

Analytical data for **3c**:

Rf = 0.54 (10% EtOAc in toluene). HR-MS *m/z* = 657.1645 $[M+Na]^+$, calculated for $C_{35}H_{35}ClNaO_7S^+$: 657.1690. ^{1}H NMR (400 MHz, $CDCl_3$) δ 8.01 – 7.91 (m, 2H), 7.74 – 7.66 (m, 1H), 7.65 – 7.60 (m, 1H), 7.59 – 7.50 (m, 3H), 7.45 – 7.22 (m, 10H), 5.33 (dd, *J* = 10.0, 9.1 Hz, 1H), 4.93 (dd, *J* = 11.1, 5.0 Hz, 2H), 4.84 (d, *J* = 11.2 Hz, 1H), 4.66 (d, *J* = 11.0 Hz, 1H), 4.55 (d, *J* = 10.0 Hz, 1H), 4.50 (dd, *J* = 11.9, 2.1 Hz, 1H), 4.29 (dd, *J* = 11.9, 4.7 Hz, 1H),

4.02 (dd, J = 15.0, 7.8 Hz, 2H), 3.93 (t, J = 8.9 Hz, 1H), 3.72 (dd, J = 9.9, 8.5 Hz, 1H), 3.65 (ddd, J = 9.8, 4.7, 2.1 Hz, 1H), 2.78 – 2.59 (m, 2H), 1.22 (t, J = 7.5 Hz, 3H). ^{13}C NMR (101 MHz, $CDCl_3$) δ 167.14, 165.39, 137.49, 135.07, 133.38, 133.19, 133.04, 129.88, 129.71, 128.75, 128.50, 128.42, 128.36, 128.32, 127.97, 127.78, 127.03, 126.15, 126.11, 126.02, 84.42, 83.80, 77.09, 76.98, 75.63, 75.22, 72.25, 64.63, 40.83, 24.23, 15.01.

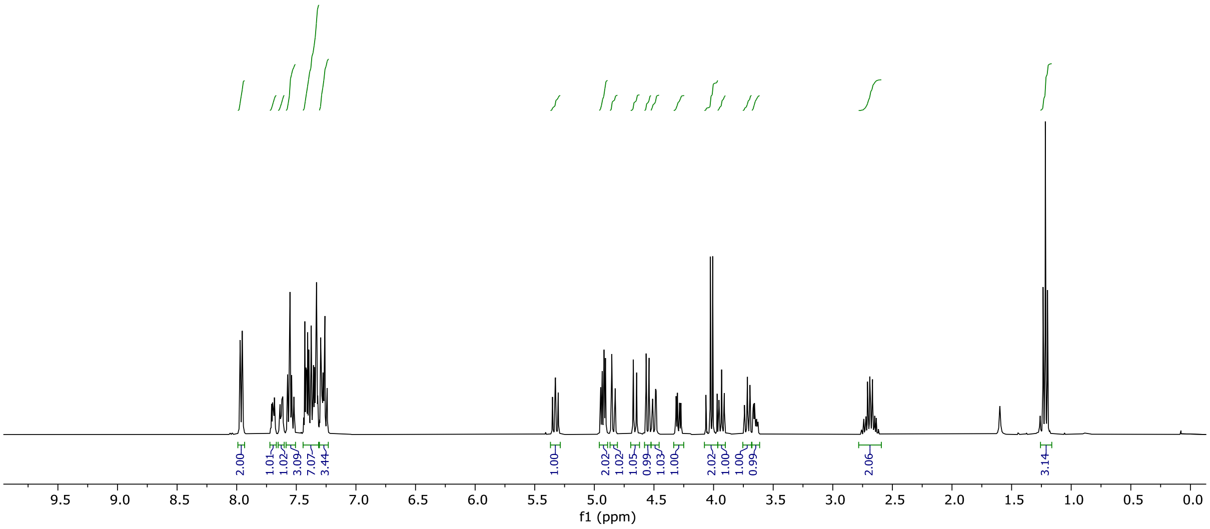

Figure S19. ^{1}H NMR (400 MHz, $CDCl_3$) spectrum of **3c**.

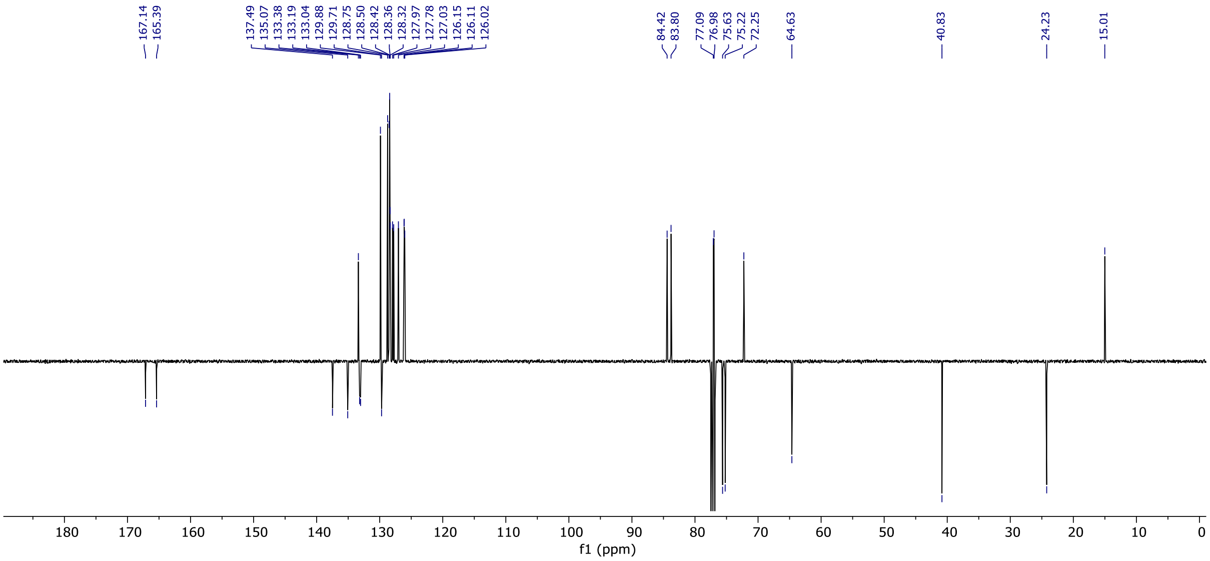

Figure S20. ^{13}C APT NMR (101 MHz, $CDCl_3$) spectrum of **3c**.

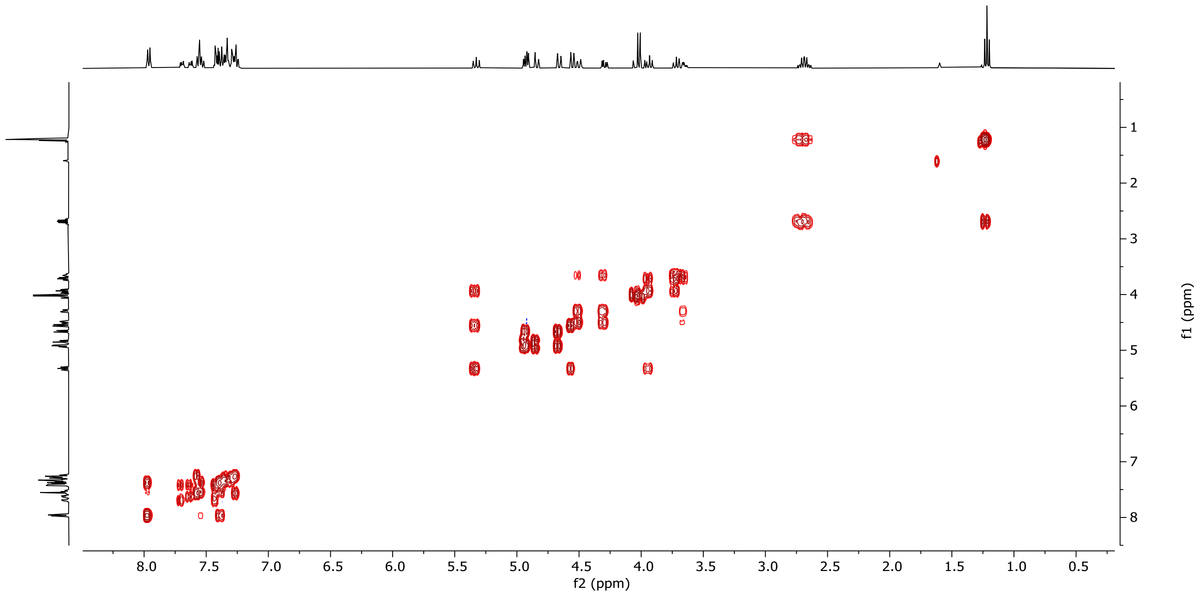

Figure S21. COSY NMR (400 MHz, $CDCl_3$) spectrum of **3c**.

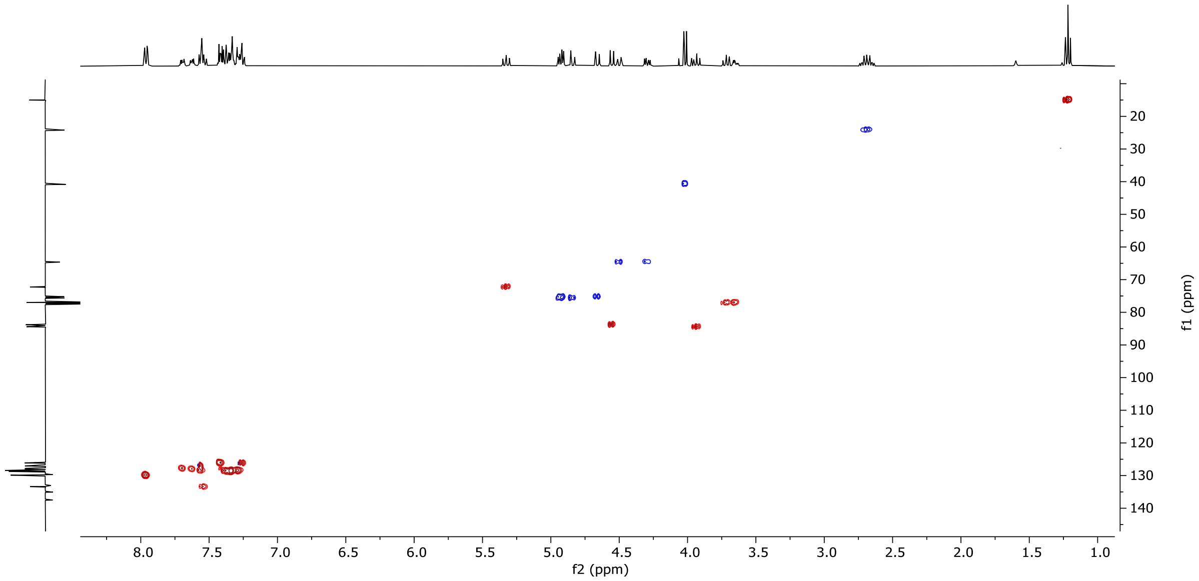

Figure S22. HSQC NMR (400MHz, $CDCl_3$) spectrum of **3c**.

3.8. Ethyl 2-*O*-benzoyl-3-*O*-(2-naphthyl)methyl-4,6-di-*O*-benzyl-1-thio-β-D-glucopyranoside (BB 8)

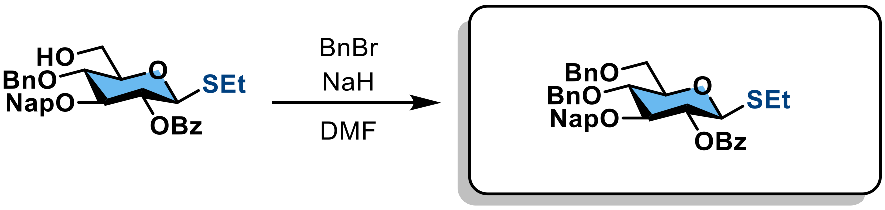

Compound **3b** (10.0 g, 17.9 mmol, 1.0 equiv.) was dissolved in anhydrous DMF (100 mL), cooled to 0 °C and sodium hydride (60% dispersion, 860 mg, 21.5 mmol, 1.2 equiv.) and benzyl bromide (freshly passed through basic alumina, 6.42 mL, 53.7 mmol, 3.0 equiv.) were added. After 2.5 hour at 0 °C, TLC indicated complete conversion of SM. The reaction mixture was then quenched with 10% aqueous citric acid, diluted with DCM (200 mL), washed with brine (3×150 mL) and dried with sodium sulfate. The drying agent was filtered off and the filtrate was concentrated *in vacuo*. The crude was purified by column chromatography (0.5 to 4% EtOAc in hex/DCM = 1/1) giving product **8** (10.84 g, 93% yield) as a white solid. R*f* (3% EtOAc in hex/DCM = 1/1) = 0.33. HR-MS *m/z* = 671.2464 $[M+Na]^+$, calculated for $C_{40}H_{40}NaO_6S^+$: 671.2438. ^{1}H NMR (400 MHz, $CDCl_3$) δ 7.99 – 7.91 (m, 2H), 7.72 – 7.63 (m, 1H), 7.62 – 7.57 (m, 1H), 7.56 – 7.48 (m, 3H), 7.42 – 7.27 (m, 12H), 7.25 – 7.17 (m, 3H), 5.34 (dd, *J* = 10.0, 8.9 Hz, 1H), 4.92 (d, *J* = 11.4 Hz, 1H), 4.84 (dd, *J* = 18.3, 10.8 Hz, 2H), 4.68 – 4.56 (m, 3H), 4.52 (d, *J* = 10.1 Hz, 1H), 3.89 (t, *J* = 9.0 Hz, 1H), 3.84 – 3.73 (m, 3H), 3.59 (ddd, *J* = 9.7, 4.4, 2.1 Hz, 1H), 2.73 (qq, *J* = 12.4, 7.4 Hz, 2H), 1.24 (t, *J* = 7.5 Hz, 3H). ^{13}C NMR (101 MHz, $CDCl_3$) δ 165.43, 138.26, 138.04, 135.39, 133.24, 133.19, 132.99, 129.88, 128.59, 128.52, 128.44, 128.25, 128.16, 128.00, 127.97, 127.88, 127.77, 127.75, 84.28, 83.61, 79.67, 78.14, 75.45, 75.28, 73.62, 72.40, 69.00, 23.99, 15.05.

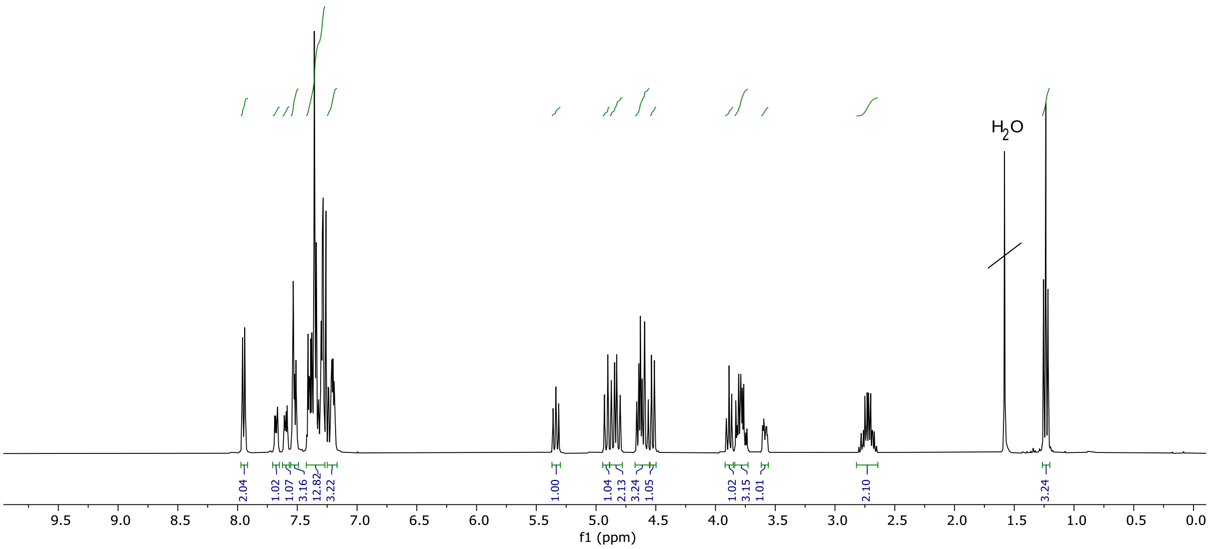

Figure S23. ^{1}H NMR (400 MHz, $CDCl_3$) spectrum of **BB 8**.

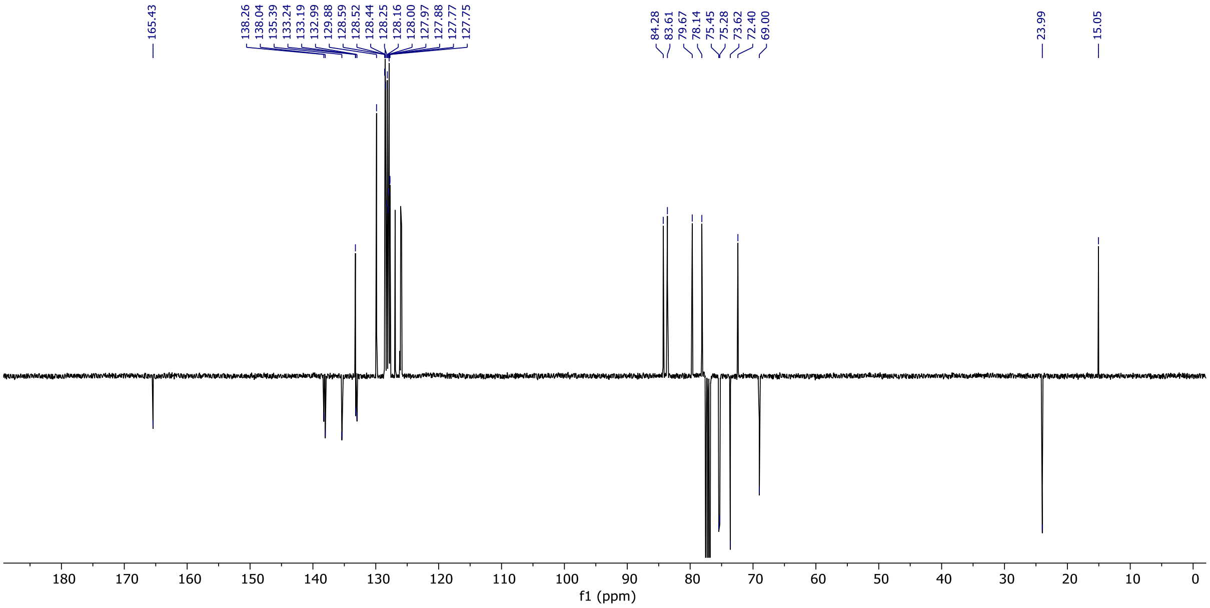

Figure S24. ^{13}C APT NMR (101 MHz, $CDCl_3$) spectrum of **BB 8**.

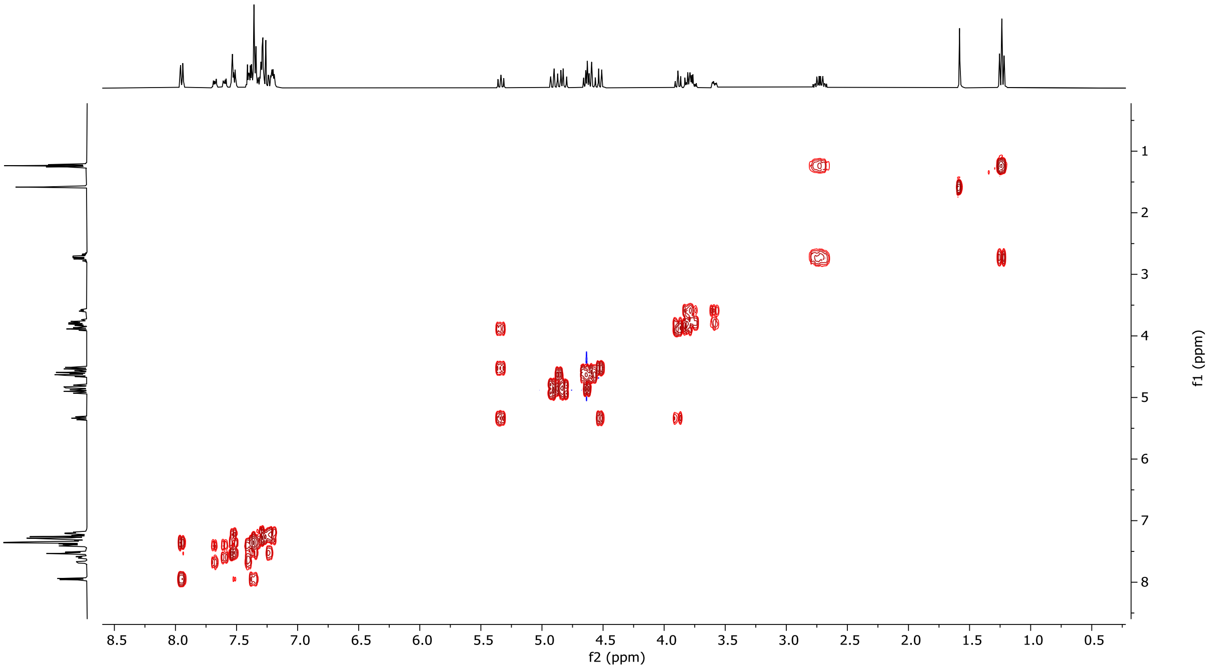

Figure S25. COSY NMR (400 MHz, $CDCl_3$) spectrum of **BB 8**.

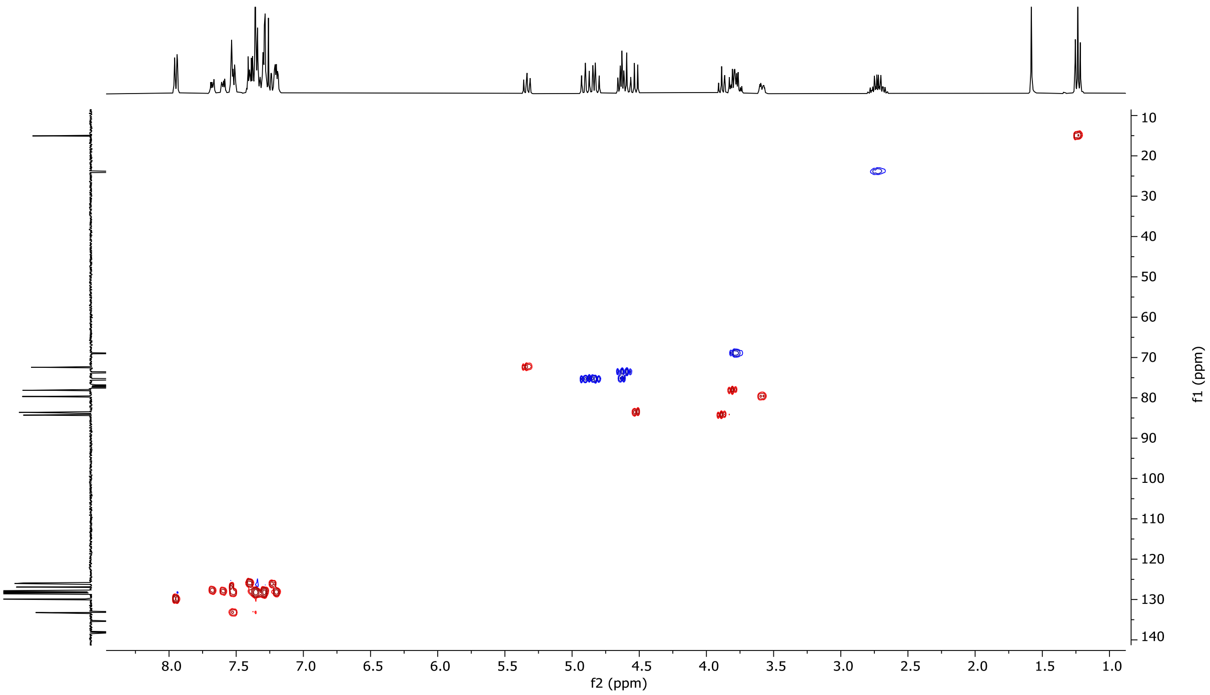

Figure S26. HSQC NMR (400MHz, $CDCl_3$) spectrum of **BB 8**.

3.9. Ethyl 2-*O*-benzoyl-4,6-di-*O*-benzyl-1-thio-β-D-glucopyranoside

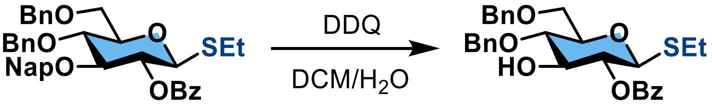

Building block **8** (9.76 g, 15.0 mmol, 1.0 equiv.) was dissolved in DCM (120 mL) and water (12 mL) was added. Upon addition of DDQ (6.83 g, 30.1 mmol, 2.0 equiv.), the mixture was stirred for one hour when TLC indicated complete conversion. The reaction mixture was then quenched with sat. aqueous $Na_2S_2O_3$ (150 mL) and sat. $NaHCO_3$ (150 mL). The organic layer was separated and the aqueous layer was extracted once more with DCM (100 mL). The combined DCM extracts were washed with brine (150 mL), dried with sodium sulfate, filtered and concentrated *in vacuo*. The crude was purified by column chromatography (2 to 10% EA in hex/DCM = 1/1) giving the product **4a** (6.40 g, 84% yield) as white solid. R*f* (10% EtOAc in hex/DCM = 1/1) = 0.35. ^{1}H NMR (400 MHz, $CDCl_3$) δ 8.11 – 8.01 (m, 2H), 7.58 (tt, *J* = 7.4, 1.8 Hz, 1H), 7.51 – 7.41 (m, 2H), 7.39 – 7.23 (m, 10H), 5.12 (dd, *J* = 10.0, 9.1 Hz, 1H), 4.80 (d, *J* = 11.2 Hz, 1H), 4.70 – 4.62 (m, 2H), 4.62 – 4.53 (m, 2H), 3.93 (td, *J* = 9.0, 3.3 Hz, 1H), 3.82 (dd, *J* = 11.0, 2.0 Hz, 1H), 3.76 (dd, *J* = 11.0, 4.3 Hz, 1H), 3.67 (dd, *J* = 9.8, 8.8 Hz, 1H), 3.55 (ddd, *J* = 9.8, 4.3, 2.0 Hz, 1H), 2.83 – 2.64 (m, 2H), 2.56 (d, *J* = 3.8 Hz, 1H), 1.26 (t, *J* = 7.4 Hz, 3H). ^{13}C NMR (101 MHz, $CDCl_3$) δ 166.36, 138.22, 138.15, 133.49, 130.12,

129.71, 128.71, 128.56, 128.54, 128.24, 128.15, 127.95, 127.82, 83.22, 79.36, 78.16, 77.28, 75.03, 73.66, 73.41, 69.03, 24.08, 15.13.

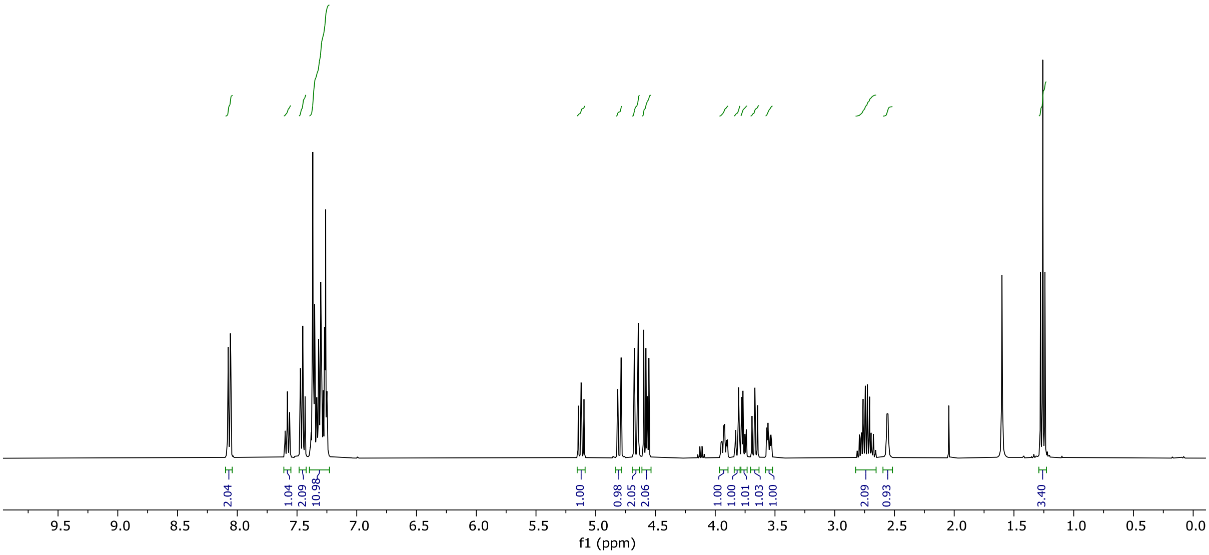

Figure S27. ^{1}H NMR (400 MHz, $CDCl_3$) spectrum of **4a**.

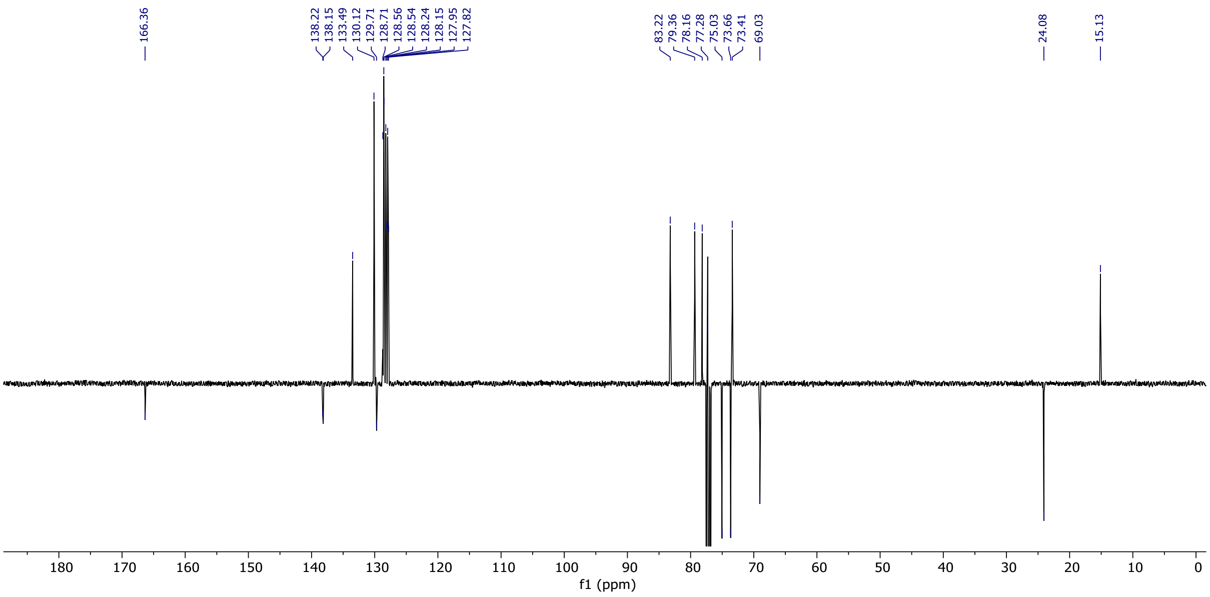

Figure S28. ^{13}C APT NMR (101 MHz, $CDCl_3$) spectrum of **4a**.

3.10. Ethyl 2-*O*-benzoyl-3-*O*-(9-fluorenylmethoxycarbonyl)-4,6-di-*O*-benzyl-1-thio-β-D-glucopyranoside (BB 4)

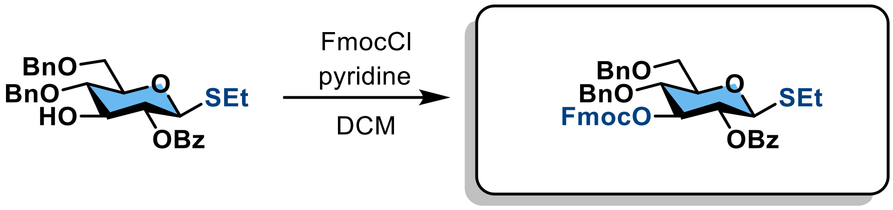

Thioglycoside **4a** (4.30 g, 8.46 mmol, 1.0 equiv.) was dissolved in anhydrous DCM (100 mL) and pyridine (2.05 mL, 25.4 mmol, 3.0 equiv.) and FmocCl (3.28 g, 12.7 mmol, 1.5 equiv.) were added. The reaction mixture was stirred for four hours at r.t. upon which time TLC indicated complete conversion of SM. The reaction mixture was washed with 10% aqueous citric acid (100 mL), brine (100 mL), dried with sodium sulfate, filtered and concentrated *in vacuo*. The crude was purified by column chromatography (0 to 4% EtOAc in DCM/hex = 1/1) to afford product **4** (5.72 g, 93% yield) as a white fluffy solid. R*f* (10% EtOAc in hex/DCM = 1/1) = 0.63. HR-MS *m/z* = 753.2529 $[M+Na]^+$, calculated for $C_{44}H_{42}NaO_8S^+$: 753.2493. ^{1}H NMR (400 MHz, $CDCl_3$) δ 8.04 – 7.96 (m, 2H), 7.69 (dd, *J* = 7.6, 3.6 Hz, 2H), 7.51 – 7.28 (m, 12H), 7.25 – 7.12 (m, 7H), 5.32 (dt, *J* = 23.7, 9.8 Hz, 2H), 4.70 – 4.61 (m, 3H), 4.57 (dd, *J* = 11.6, 4.5 Hz, 2H), 4.25 (dd, *J* = 10.4, 7.1 Hz, 1H), 4.07 (dd, *J* = 10.4, 8.0 Hz, 1H), 3.99 – 3.88 (m, 2H), 3.87 – 3.72 (m, 2H), 3.64 (ddd, *J* = 9.8, 3.9, 2.1 Hz, 1H), 2.85 – 2.66 (m, 2H), 1.26 (t, *J* = 7.4 Hz, 3H). ^{13}C NMR (101 MHz, $CDCl_3$) δ 165.41, 154.79, 143.47, 143.03, 141.25, 141.15, 138.13, 137.62, 133.42, 130.11, 129.34, 128.55, 128.49, 128.46, 128.00, 127.97, 127.92, 127.88, 127.85, 127.24, 120.02, 120.00, 83.51, 80.84, 79.43, 75.87, 75.05, 73.65, 70.88, 70.34, 68.66, 46.52, 24.24, 15.02.

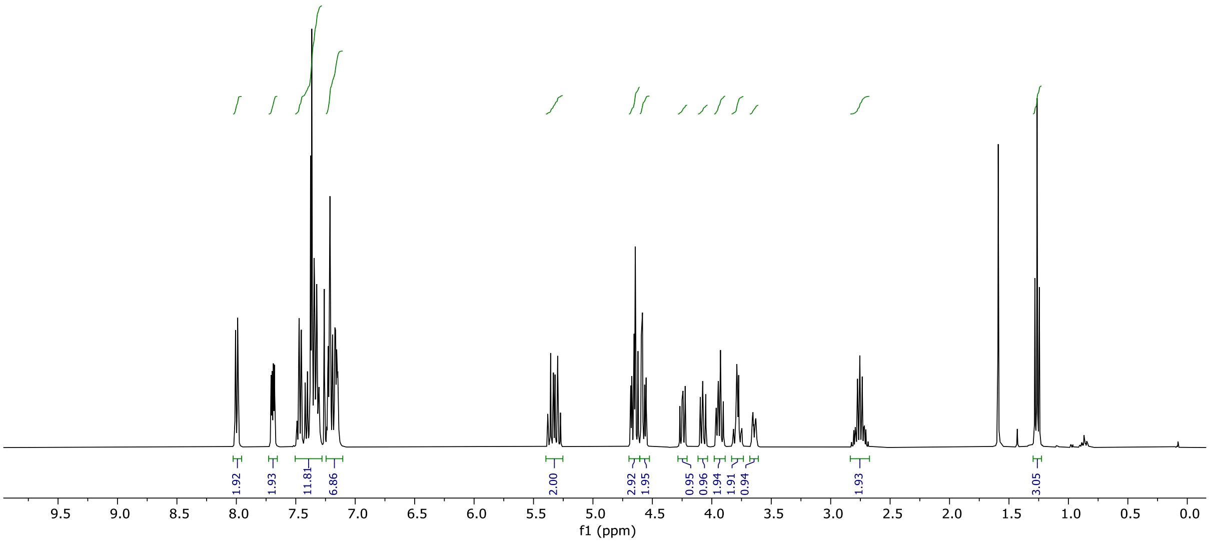

Figure S29. ^{1}H NMR (400 MHz, $CDCl_3$) spectrum of **BB 4**.

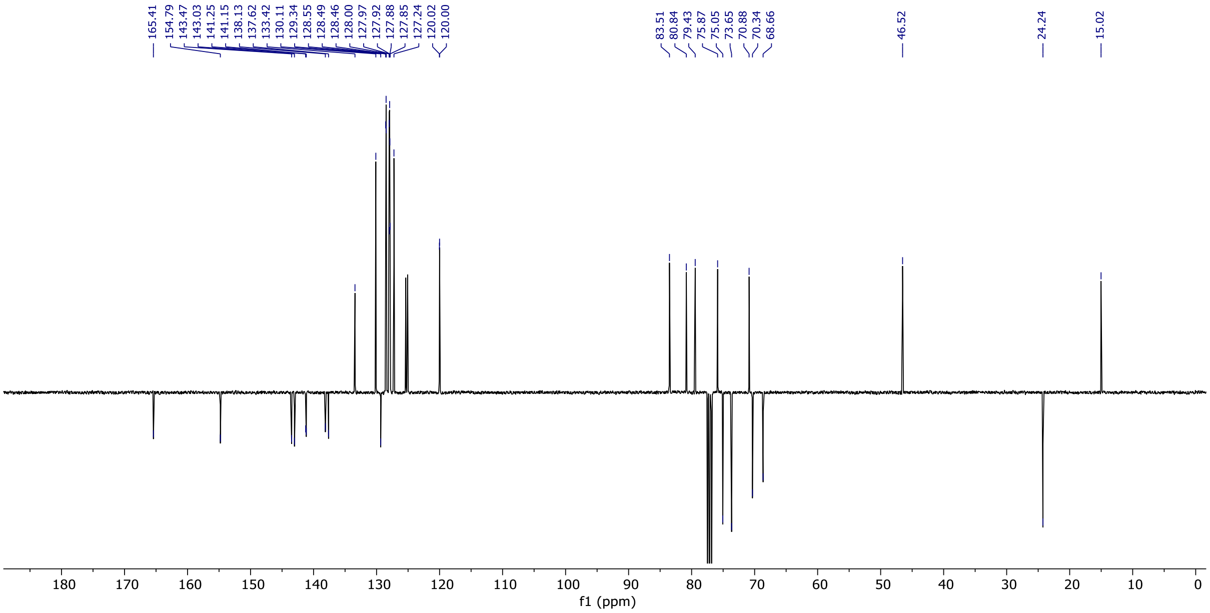

Figure S30. ^{13}C APT NMR (101 MHz, $CDCl_3$) spectrum of **BB 4**.

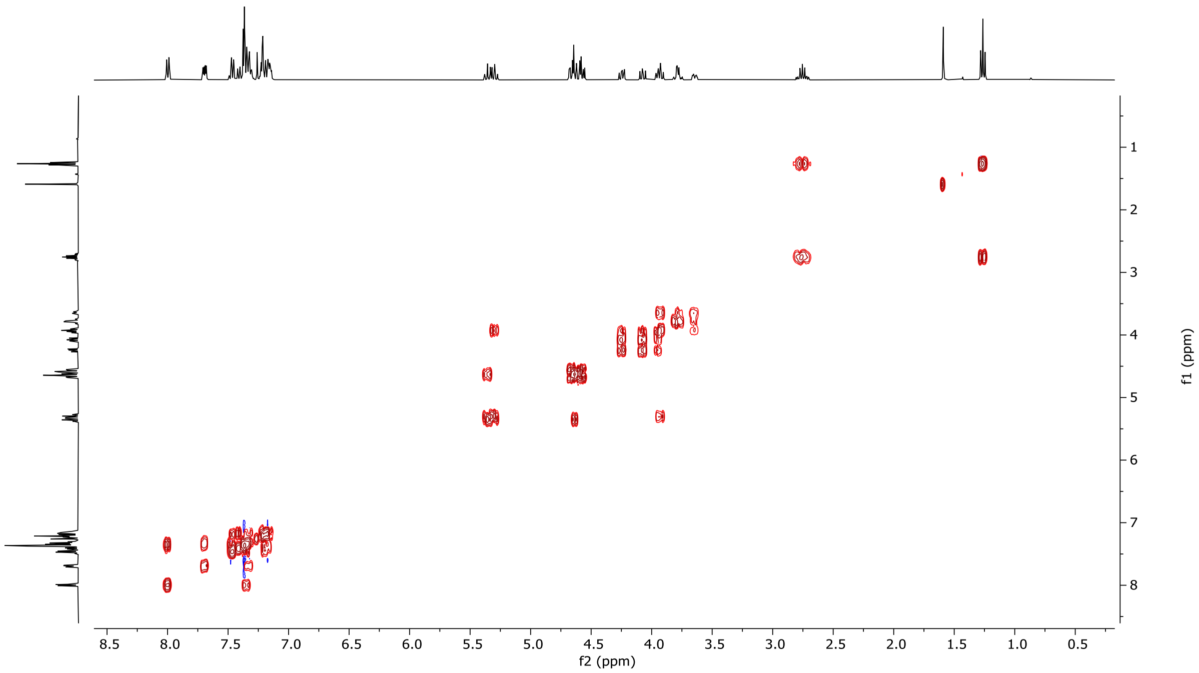

Figure S31. COSY NMR (400 MHz, $CDCl_3$) spectrum of **BB 4**.

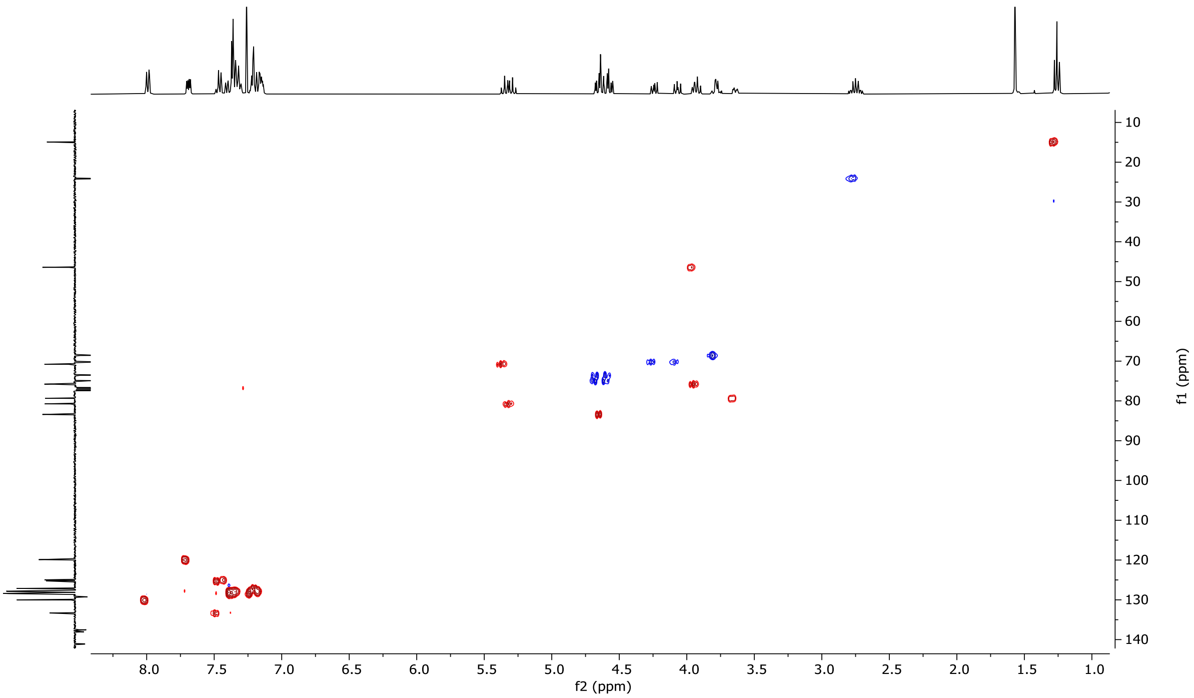

Figure S32. HSQC NMR (400MHz, $CDCl_3$) spectrum of **BB 4**.

3.11. 17-((Allyloxycarbonyl)amino)heptadecanoic acid (AA 20)

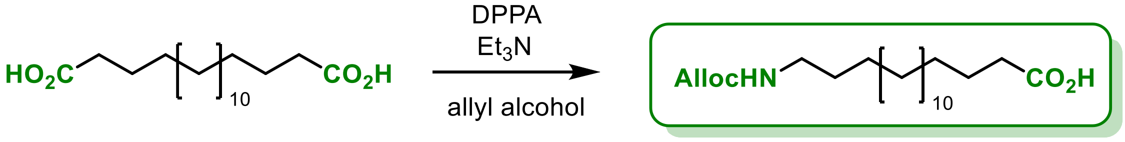

Octadecanedioic acid (2.50 g, 7.95 mmol, 1.0 equiv.) was dissolved in allyl alcohol (50 mL), Et_3N (1.66 mL, 11.9 mmol, 1.5 equiv.) and DPPA (2.57 mL, 11.9 mmol, 1.5 equiv.) were added and the mixture was heated to 100 °C for 16 hours. The reaction mixture was then concentrated *in vacuo* and partitioned between DCM (100 mL) and 10% aqueous citric acid (100 mL). The organic layer was separated, dried with sodium sulfate and concentrated *in vacuo*. The crude was purified by flash chromatography (0 to 15% EA in hex/DCM = 1/1 with 0.2% AcOH) giving product **20** (395 mg, 13% yield, unoptimized) as a white powder. R*f* (10% MeOH/DCM with 0.2% AcOH) = 0.71. HR-MS *m/z* = 392.2768 $[M+Na]^+$, calculated for $C_{21}H_{39}NNaO_4^+$: 392.2771. ^{1}H NMR (400 MHz, $CDCl_3$) δ 5.92 (ddt, *J* = 16.4, 10.9, 5.7 Hz, 1H), 5.30 (dd, *J* = 17.3, 1.7 Hz, 1H), 5.21 (dd, *J* = 10.5, 1.8 Hz, 1H), 4.77 – 4.51 (m, 3H), 3.17 (q, *J* = 6.6 Hz, 2H), 2.34 (t, *J* = 7.5 Hz, 2H), 1.63 (p, *J* = 7.4 Hz, 2H), 1.49 (p, *J* = 7.1 Hz, 2H), 1.38 – 1.17 (m, 24H). ^{13}C NMR (101 MHz, $CDCl_3$) δ 178.92, 156.42, 133.14, 117.76, 65.58, 41.22, 34.01, 30.10, 29.73, 29.67, 29.53, 29.41, 29.36, 29.18, 26.87, 24.83.

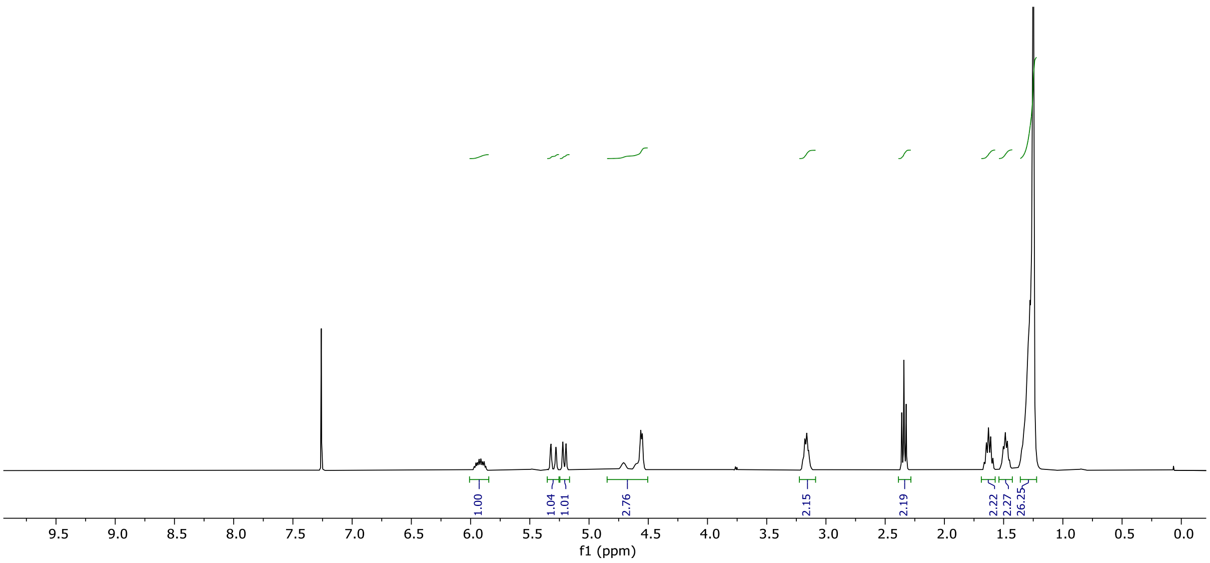

Figure S33. ^{1}H NMR (400 MHz, $CDCl_3$) spectrum of **AA 20**.

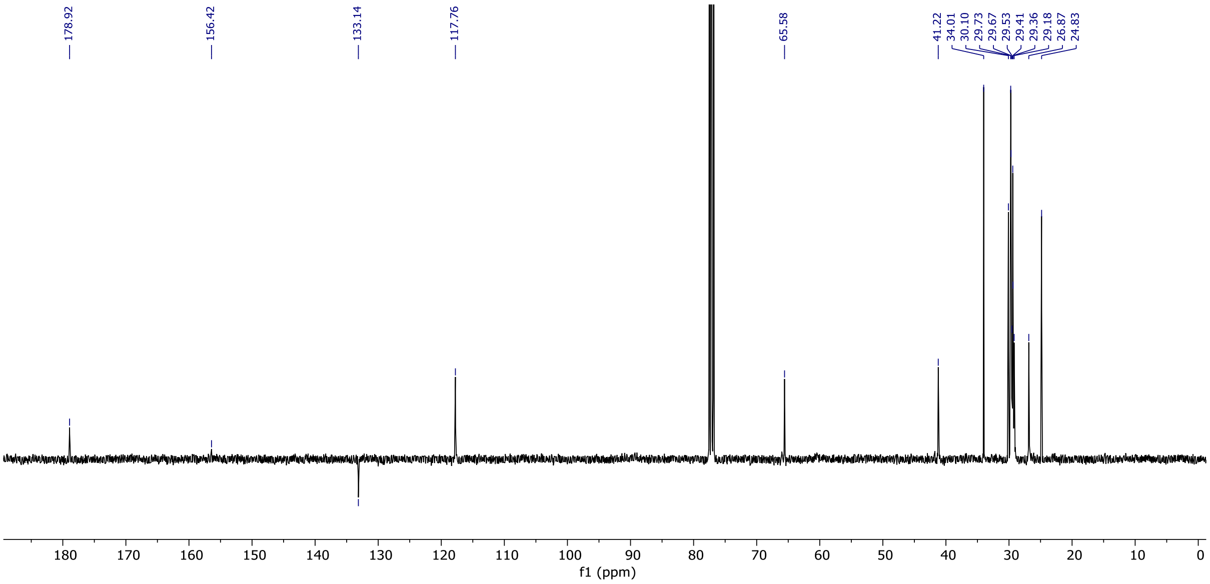

Figure S34. ^{13}C APT NMR (101 MHz, $CDCl_3$) spectrum of **AA 20**.

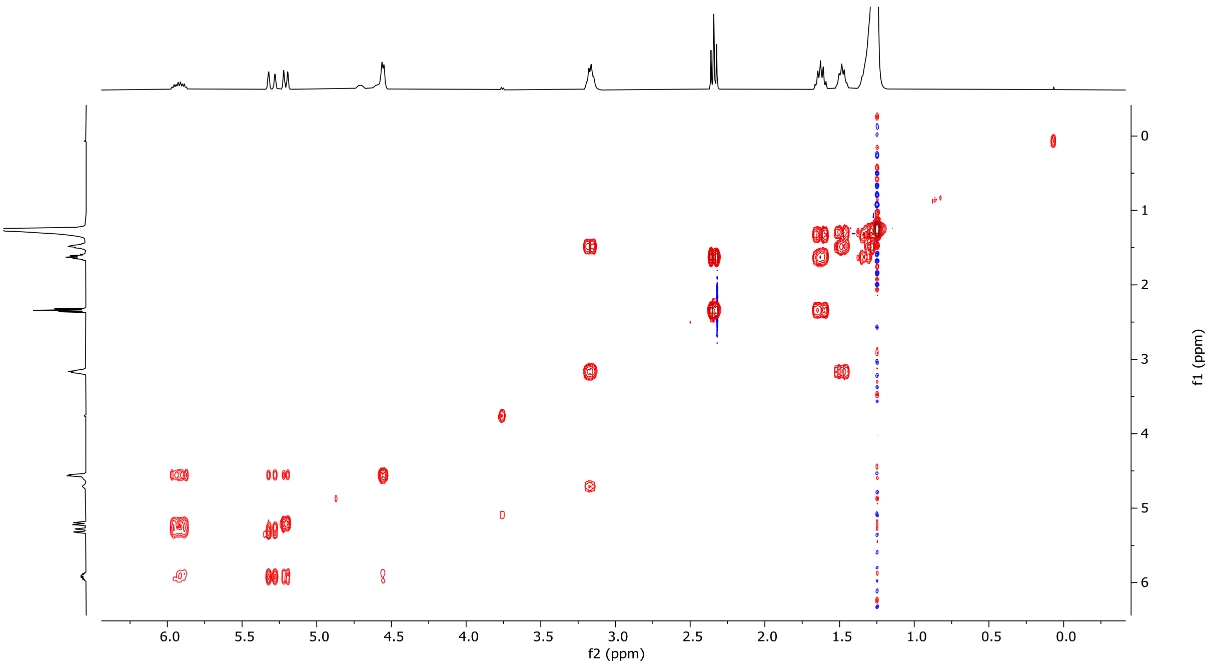

Figure S35. COSY NMR (400 MHz, $CDCl_3$) spectrum of **AA 20**.

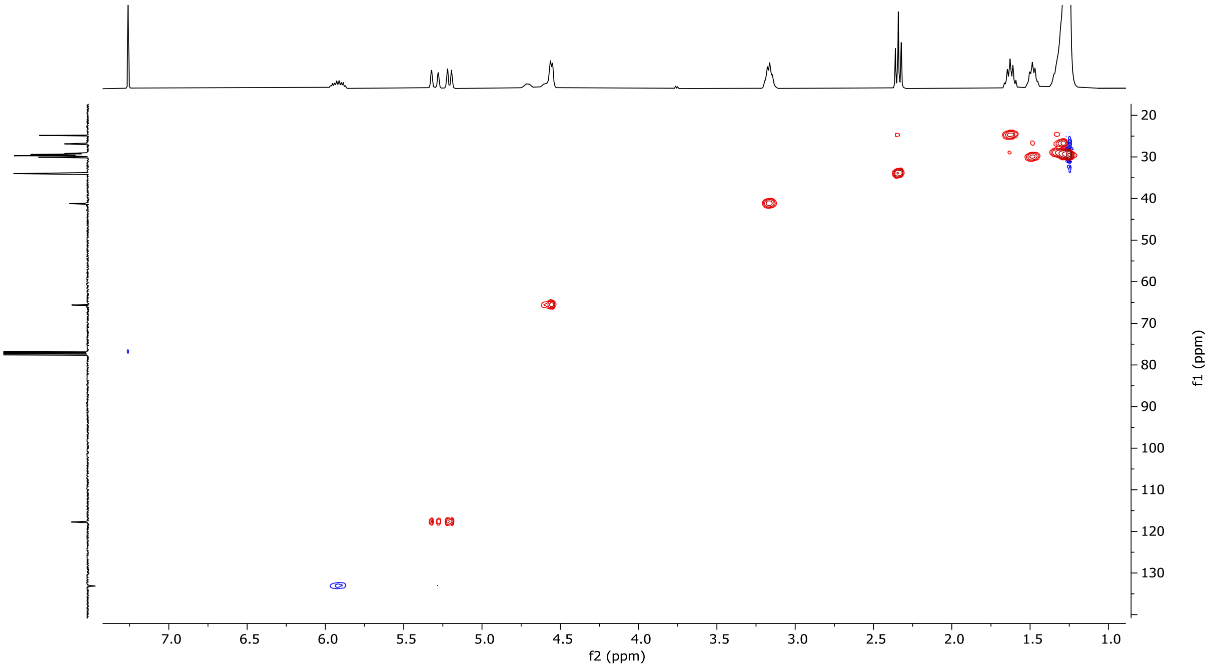

Figure S36. HSQC NMR (400MHz, $CDCl_3$) spectrum of **AA 20**.

4. Resin functionalization

Functionalized Merrifield resin with decreased loading was synthesized by limiting the linker reactant in an established procedure.[2]

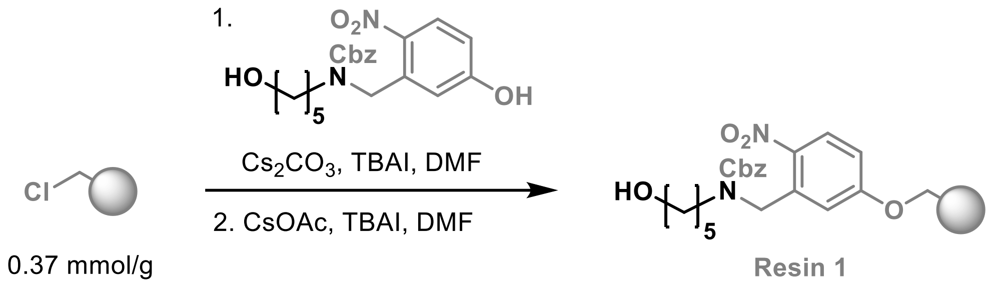

As an example, Merrifield resin (2 g, initial loading 0.37 mmol/g, 0.74 mmol) was washed three times with DCM and three times with DMF. The resin was then suspended in anhydrous DMF (40 mL) and linker (186 mg, 0.48 mmol, two equivalents corresponding to the desired loading) was added followed by addition of freshly dried cesium carbonate (195 mg, 0.60 mmol, 2.5 equiv. corresponding to the desired loading) and TBAI (44 mg, 0.12 mmol, 0.5 equiv. corresponding to the desired loading). The mixture was spun, protected from light, on a rotavap at 60 °C and 600 mbar for 20 hours. Freshly dried cesium acetate (710 mg, 3.7 mmol, 5 equiv.) together with TBAI (273 mg, 0.74 mmol, 1 equiv.) were added to cap unreacted chloromethyl groups and the mixture was spun, protected from light, on a rotavap at 60 °C and 600 mbar for an additional 20 hours. The mixture was then filtered, and the resin was washed with DMF, THF, THF/H_2O (2/1), H_2O, THF, MeOH, DMF and DCM. The resin was then dried *in vacuo* and stored protected from light. Loading of 0.12 mmol/g was determined by glycosylation and Fmoc quantification.[3]

Using the same procedure, but different amounts of linker (233 mg, 0.60 mmol), cesium carbonate (244 mg, 0.75 mmol) and TBAI (55 mg, 0.15 mmol) in the first step gave a resin with a loading of 0.14 mmol/g. Similarly, using linker (280 mg, 0.72 mmol), cesium carbonate (293 mg, 0.9 mmol) and TBAI (66 mg, 0.18 mmol) gave a resin with a loading of 0.16 mmol/g.

---

[2] Hoang K. L. M. *et. al.* Traceless Photolabile Linker Expedites the Chemical Synthesis of Complex Oligosaccharides by Automated Glycan Assembly, *J. Am. Chem. Soc.* **2019**, 141 (22), 9079-9086

[3] Gude, M.; Ryf, J.; White, P. D. An Accurate Method for the Quantitation of Fmoc-Derivatized Solid Phase Supports. *Lett. Pept. Sci.* **2002**, 9, 203-206

5. Automated Glycan Assembly

Solvents used for the preparation of building block solutions, activator, acid wash (TMSOTf), and capping solutions were either dried with freshly activated 4Å molecular sieves or taken from an anhydrous solvent system (JC Meyer-solvent systems). Other solvents used were HPLC grade. The building blocks were co-evaporated three times with toluene and dried overnight under high vacuum before use. All solutions were freshly prepared and kept under argon during the automation run. Final yields were calculated based on resin loading, which was determined by performing one glycosylation (Module C) followed by DBU-promoted Fmoc cleavage and determination of dibenzofulvene formation by measuring its UV absorbance.[4]

5.1. Preparation of reagent solutions

- **Building block solution**: Building block (0.10 mmol) was dissolved in DCM (1 mL).
- **NIS/TfOH activator solution**: Recrystallized NIS (1.57 g, 7.0 mmol) was dissolved in 45 mL of a 2:1 v/v mixture of anhydrous DCM and anhydrous dioxane. Then, triflic acid (64 μL, 0.7 mmol) was added. The solution is kept at 0°C for the duration of the automation run.
- **Fmoc deprotection solution A**: A solution of 20% piperidine in DMF (v/v) was prepared.
- **Fmoc deprotection solution B**: A solution of 20% triethylamine in DMF (v/v) was prepared.
- **TMSOTf solution**: TMSOTf (0.45 mL, 2.49 mmol) was dissolved in DCM (40 mL).

5.2. Modules for Automated Solid-Phase Synthesis

Module A: Resin preparation for synthesis

All automated syntheses were performed on a 0.015 mmol scale. The given resin (0.015 mmol of free sites) was placed in the reaction vessel and was swollen in DCM for 20 min at room temperature before synthesis. During this time, all reagent lines needed for the synthesis were washed and primed. After swelling, the resin was washed with DMF, THF, and DCM (three times each with 2 mL for 25 s).

[4] Kröck, L. *et al.* Streamlined access to conjugation-ready glycans by automated synthesis. *Chem. Sci.* **2012**, *3*, 1617−1622.

Module B: Acidic wash (TMSOTf solution)

The resin was swollen in DCM (2 mL) and the temperature of the reaction vessel was adjusted to -20 °C. Upon reaching this temperature, TMSOTf solution (1 mL, 0.06 mmol) was added dropwise to the reaction vessel. After bubbling for three minutes, the acidic solution was drained and the resin was washed with DCM (2 mL) for 25 s.

Action	Cycles	Solution	Amount	T (°C)	Incubation time
Cooling	-	-	-	-20	-
Deliver	1	DCM	2 mL	-20	-
Deliver	1	TMSOTf solution	1 mL	-20	3 min
Wash	1	DCM	1 mL	-20	25 s

Module C1: Thioglycoside glycosylation × 1 cycle

The standard thioglycoside-based glycosylation conditions are used for 5-AMP resins above loading of 0.15 mmol/g.[5] The building block solution (0.10 mmol of BB in 1 mL of DCM per glycosylation) was delivered to the reaction vessel. After the set temperature was reached, the reaction was started by dropwise addition of the activator solution (1.0 mL, 0.15 mmol). After completion of the reaction, the solution is drained and the resin was washed with DCM, DCM/dioxane (1:2, *V/V*, 3 mL for 20 s), and DCM (twice, each with 2 mL for 25 s). The temperature of the reaction vessel is increased to 25°C for the next module.

Action	Cycles	Solution	Amount	T (°C)	Incubation time
Cooling	-	-	-	-20	~15 min
Deliver	1	BB solution	1 mL	-20	-
Deliver	1	Activator solution A	1 mL	-20	3 min
Reaction time 1	1	-	-	-20	10 min
Reaction time 2	1	-	-	0	30 min
Wash	1	DCM	2 mL	0	5 s
Wash	1	DCM / Dioxane 2:1	2 mL	0	20 s
Heating	-	-	-	25	-
Wash	2	DCM	2 mL	>0	25 s

[5] Joseph, A. A.; Pardo-Vargas, A.; Seeberger, P.H. *J. Am. Chem. Soc.* **2020**, *142*, 19, 8561–8564.

Module C2: Thioglycoside glycosylation × 2 cycles

These glycosylation conditions are used for resin **1** with loading not higher than 0.15 mmol/g and for resin **2**. The building block solution (0.10 mmol of BB in 1 mL of DCM per glycosylation) was delivered to the reaction vessel. After the set temperature was reached, the reaction was started by dropwise addition of the activator solution (1.0 mL, 0.15 mmol). After completion of the reaction, the solution is drained and the resin was washed with DCM, DCM/dioxane (1:2, *V*/*V*, 3 mL for 20 s), and DCM (twice, each with 2 mL for 25 s). The temperature of the reaction vessel is increased to 25°C for the next module.

Action	Cycles	Solution	Amount	T (°C)	Incubation time
Cooling	-	-	-	-20	-
Deliver	1	BB solution	1 mL	-20	-
Deliver	1	Activator solution B	1 mL	-20	3 min
Reaction time 1	1	-	-	-20	10 min
Reaction time 2	1	-	-	0	30 min
Wash	1	DCM	2 mL	0	5 s
Wash	1	DCM / Dioxane 2:1	2 mL	0	20 s
Cooling	-	-	-	-20	15 min
Deliver	1	BB solution	1 mL	-20	-
Deliver	1	Activator solution B	1 mL	-20	3 min
Reaction time 1	1	-	-	-20	10 min
Reaction time 2	1	-	-	0	30 min
Wash	1	DCM	2 mL	0	5 s
Wash	1	DCM / Dioxane 2:1	2 mL	0	20 s
Heating	-	-	-	25	-
Wash	2	DCM	2 mL	>0	25 s

Module D1: Fmoc deprotection with piperidine

The resin was washed with DMF (three times with 2 mL for 25 s) and the temperature of the reaction vessel was adjusted to 25 °C. 2 mL of Fmoc deprotection solution was delivered to the reaction vessel and kept under Ar bubbling. After five minutes, the reaction solution was drained and the resin was washed with DMF (three times with two mL for 25 s) and DCM (five times each with 2 mL for 25 s). The temperature of the reaction vessel was decreased to -20 °C for the next module.

Action	Cycles	Solution	Amount	T (°C)	Incubation time
Wash	3	DMF	2 mL	25	25 s
Deliver	1	Fmoc depr. A	2 mL	25	5 min
Wash	3	DMF	2 mL	25	25 s
Wash	5	DCM	2 mL	25	25 s
Cooling	1	-	-	-20	-

Module D2: Fmoc deprotection with triethylamine

The resin was washed with DMF (three times with 2 mL for 25 s) and the temperature of the reaction vessel was adjusted to 25 °C. Fmoc deprotection solution (2 mL) was delivered to the reaction vessel and kept under Ar bubbling. After five minutes, the reaction solution was drained and the resin was washed with DMF (three times with 2 mL for 25 s) and DCM (five times each with 2 mL for 25 s). The temperature of the reaction vessel was decreased to -20 °C for the next module.

Action	Cycles	Solution	Amount	T (°C)	Incubation time
Wash	3	DMF	2 mL	25	25 s
Deliver	3	Fmoc depr. B	2 mL	25	5 min
Wash	3	DMF	2 mL	25	25 s
Wash	5	DCM	2 mL	25	25 s
Cooling	1	-	-	-20	-

5.3. Post-AGA manipulations

Module E: *N*-Fmoc deprotection

The resin was stirred using a mechanical stirrer in a filter-equipped syringe (5 mL). It was washed with DCM and then DMF (both three times with 3 mL for 10 s). The resin was then treated with 20% piperidine in DMF (three times with 3 mL for 5 min), followed by washing with DMF and DCM (both three times with 3 mL for 10 s).

Action	Cycles	Solution	Amount	T (°C)	Incubation time
Wash	3	DCM	3 mL	25	10 s
Wash	3	DMF	3 mL	25	10 s
Deliver	3	Fmoc depr. A	3 mL	25	5 min

Wash	3	DMF	3 mL	25	10 s
Wash	3	DCM	3 mL	25	10 s

Module F: Amide coupling

The resin was stirred by a mechanical stirrer in a filter-equipped syringe (5 mL). It was washed with DCM and then DMF (both three times with 3 mL for 10 s). The given carboxylic acid (0.22 mmol) was dissolved in anhydrous DMF (1.5 mL), *N*-methylmorpholine (50 µL, 0.44 mmol) was added, followed by addition of HATU (76 mg, 0.2 mmol). This solution was left to pre-activate for four minutes before adding it to the washed resin and stirring for 2 hours. The resin was then washed with DMF and DCM (both three times with 3 mL for 10 s).

Action	Cycles	Solution	Amount	T (°C)	Incubation time
Wash	3	DCM	3 mL	25	10 s
Wash	3	DMF	3 mL	25	10 s
Deliver	1	Preact. acid soln.	1.5 mL	25	2 h
Wash	3	DMF	3 mL	25	10 s
Wash	3	DCM	3 mL	25	10 s

Module G: Acetylation

The resin was stirred using a mechanical stirrer in a filter-equipped syringe (5 mL). It was washed with DCM and then DMF (both three times with 3 mL for 10 s). An acetylation mixture of Ac_2O (100 µL) and DIPEA (200 µL) in DMF (1 mL) was added and the mixture was stirred for two hours. The resin was then washed with DMF and DCM (both three times with 3 mL for 10 s).

Action	Cycles	Solution	Amount	T (°C)	Incubation time
Wash	3	DCM	3 mL	25	10 s
Wash	3	DMF	3 mL	25	10 s
Deliver	1	Acetylation soln.	1.3 mL	25	2 h
Wash	3	DMF	3 mL	25	10 s
Wash	3	DCM	3 mL	25	10 s

Module H: *N*-MMT deprotection

The resin was stirred by a mechanical stirrer in a filter-equipped syringe (5 mL). It was washed with DMF and then DCM (both three times with 3 mL for 10 s). The resin was then treated

with solution of 2% TFA and 5% TIPS (*i*-Pr_3SiH) in DCM (three times with 3 mL for 10 min), followed by washing with DCM (three times with 3 mL for 10 s).

Action	Cycles	Solution	Amount	T (°C)	Incubation time
Wash	3	DMF	3 mL	25	10 s
Wash	3	DCM	3 mL	25	10 s
Deliver	3	TFA and TIPS soln.	3 mL	25	10 min
Wash	3	DCM	3 mL	25	10 s

Module I: Allyl deprotection

The resin was stirred by bubbling a weak stream of argon through the solution in a filter-equipped syringe (5 mL). It was washed with DCM (three times with 3 mL for 10 s). Fresh DCM (3 mL) was added and left to degas by bubbling argon through the solution for three minutes. Phenylsilane (37 µL, 0.3 mmol) was added followed by addition of $Pd(PPh_3)_4$ (1 mg). The mixture was then left to react for 10 minutes while still bubbling argon through it. The reaction was repeated two more times and the resin was then washed with DCM (three times with 3 mL for 10 s).

Action	Cycles	Solution	Amount	T (°C)	Incubation time
Wash	3	DMF	3 mL	25	10 s
Wash	3	DCM	3 mL	25	10 s
Deliver	3	$PhSiH_3$, $Pd(PPh_3)_4$	3 mL	25	10 min
Wash	3	DCM	3 mL	25	10 s

Module J: Peptide stapling

The resin in a filter-equipped syringe (5 mL) was washed with DCM, DMF (both three times with 3 mL for 10 s), DCM, Et_2O, DCM, Et_2O, DCM, Et_2O, DCM (always with 3 mL for 10 s) and dried overnight under high vacuum. A solution of *N*-methylmorpholine (10 µL, 0.09 mmol) in anhydrous DMF (1.5 mL) was sucked in the syringe with the resin and the syringe was shaken. A solution of PyAOP (23 mg, 0.045 mmol) in anhydrous DMF (1.5 mL) was also sucked in the syringe, the syringe was caped and shaken overnight.

Module K1: On-resin methanolysis

The resin was swollen in THF (3.0 mL) and a solution of NaOMe in MeOH (0.5 M, 0.5 mL) was added and the mixture was shaken in a filter-equipped syringe (5 mL) at room temperature

for 1.5 h. The solution was then drained and the resin was washed three times with THF/MeOH mixture (6/1, *V*/*V*).

Module L: On-resin hydrazinolysis

The resin was shaken in a filter-equipped syringe (5 mL) with 1M hydrazine in THF (2.5 mL) at room temperature overnight. The solution was then drained and the resin was washed three times with THF.

5.4. Post-solid-phase manipulations

Module M: Cleavage from the solid support

The oligosaccharides were cleaved from the solid support using a continuous-flow photoreactor as described previously.[6] For fully-protected oligosaccharides, DCM was used as a solvent. For semi-deprotected oligosaccharides, 10% MeOH/DCM (*V*/*V*) mixture was used as a solvent. The photo-cleavage was executed three times to ensure full cleavage.

Module K2: In-solution methanolysis

The protected oligosaccharide was dissolved in THF (3.0 mL, fresh from anhydrous solvent system). A solution of NaOMe in MeOH (0.5 M, 0.5 mL) was added and the mixture was stirred at room temperature for 1.5 h. The reaction was neutralized with Amberlite IR-120(H), the Amberlite was washed with THF and MeOH, and the filtrate was concentrated *in vacuo*. The crude compound was used for the hydrogenolysis reaction without further purification.

Module N1: Hydrogenolysis

The crude compound was dissolved in 3 mL of EA: *t*-BuOH: H_2O (2:2:1). Pd-C (10%, 0.1 g) was added and the reaction was stirred under H_2 atmosphere for 16 h. The reaction was filtered through cellite and washed with MeCN and H_2O. The filtrates were concentrated *in vacuum*, and dissolved in 3.0 mL of water for RP-HPLC purification.

Module N2: Hydrogenolysis

The crude compound was dissolved in THF (2.5 mL, fresh from anhydrous solvent system). Deionized water (1 mL) and AcOH (0.1 mL) were added followed by Pd/C (5%, 60 mg, Nobelyst®) was added and the reaction was stirred at 800 RPM under H_2 atmosphere for 16 h.

---

[6] Hurevich, M.; Kandasamy, J.; Ponnappa, B. M.; Collot, M.; Kopetzki, D.; McQuade, D. T.; Seeberger, P. H. Continuous Photochemical Cleavage of Linkers for Solid-Phase Synthesis. *Org. Lett.* **2014**, *16*, 1794–1797.

The reaction was filtered through cellite and washed with H_2O and THF. The filtrates were concentrated *in vacuum*, and dissolved in 3.0 mL of water, or 3.0 mL of 40% MeOH (for structures with aliphatic side-chains) for RP-HPLC purification.

Module O: HPLC analysis and purification

Analytical traces of crude and pure compounds were collected using an analytic RP-HPLC Agilent 1200 Series (**Methods 1, 2 and 3**). Purification of the crudes was conducted using a preparative RP-HPLC Agilent 1200 Series (**Methods 4, 5 and 6**).

- **Method 1:** (Hypercarb column, 150 x 4.6 mm, 3 µm) flow rate of 0.7 mL/min with ACN/H_2O (0.1% formic acid) as eluents [isocratic 100 % H_2O (0.1% formic acid) (5 min), linear gradient to 70% ACN (30 min)].
- **Method 2:** (Synergi C18 column, 250 x 4.6 mm, 4 µm), flow rate of 1 mL/min with ACN/H_2O (0.1% formic acid) as eluents [isocratic 5% ACN (5 min), linear gradient to 100% ACN (35 min)].
- **Method 3:** (Phenomenex, luna C5 column, 250 x 4.6 mm, 5 µm), flow rate of 0.5 mL/min with ACN/H_2O (0.1% formic acid) as eluents [isocratic 5% ACN (5 min), linear gradient to 100% ACN (30 min)].
- **Method 4:** (Hypercarb column, 150 x 10 mm, 5 µm), flow rate of 3.5 mL/min with H_2O (0.1% formic acid) as eluents [isocratic 100 % H_2O (0.1% formic acid) (5 min), linear gradient to 70% ACN (30 min)].
- **Method 5:** (Synergi C18 column, 250 x 10 mm, 4 µm), flow rate of 4 mL/min with H_2O (0.1% formic acid) as eluents [isocratic 100 % H_2O (0.1% formic acid) (5 min), linear gradient to 100% ACN (35 min)].
- **Method 6:** (Phenomenex, Luna C5 column, 250 x 10 mm, 5 µm), flow rate of 4 mL/min with ACN/H_2O (0.1% formic acid) as eluents [isocratic 5% ACN (5 min), linear gradient to 100% ACN (30 min)].

Following final purification, all final products were lyophilized on a Christ Alpha 2-4 LD plus freeze-dryer before characterization.

5.5. Oligosaccharide synthesis

Synthesis and analytical data of 19

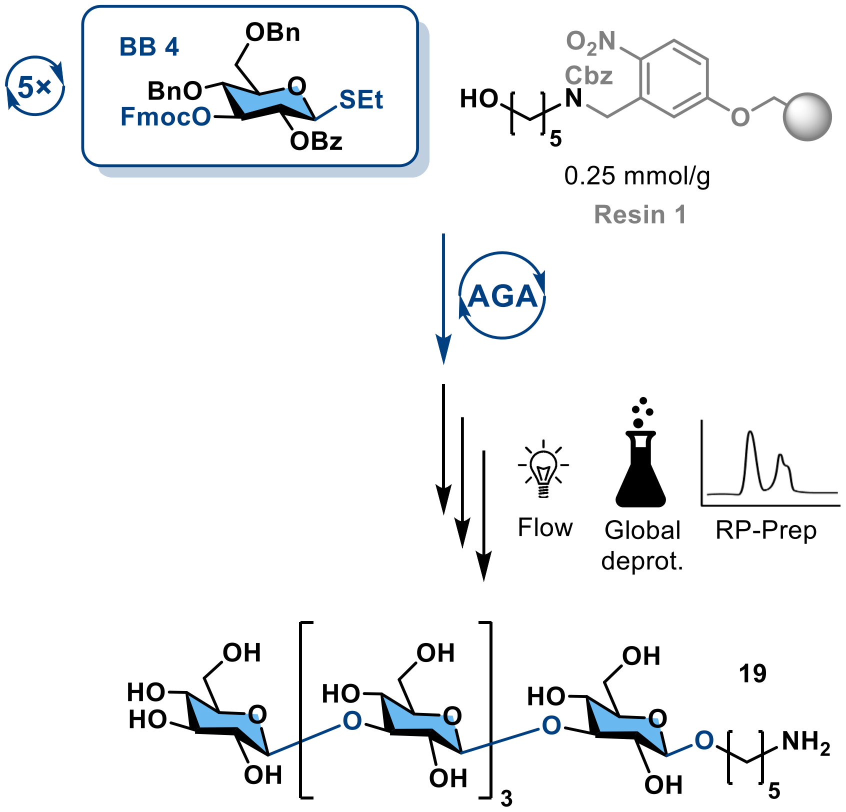

Step	Module	BB/reagent	Repeat	Notes
AGA	A	**1** (0.015 mmol)	1	0.25 mmol/g
	B, C1, D1	**4** (0.10 mmol)	4	-
	B, C1	**4** (0.10 mmol)	1	-
Post solid-phase	L	-	-	-
	M	-	3	-
	N1	-	-	-
	O	-	-	Methods 1 and 4

After automated glycan assembly, hydrazinolysis, photo-cleavage, hydrogenolysis, purification, and lyophilization **19** was obtained as a white solid (1.6 mg, 12%).

R_t (Method 1) = 23.7 min.

HRMS (ESI/Q-TOF) *m/z*: $[M + H]^+$ Calcd for $C_{35}H_{64}NO_{26}$ 914.3711; Found 914.3761.

^{1}H NMR (700 MHz, D_2O) δ 4.45 (d, *J* = 8.1 Hz, 1H), 3.93 – 3.86 (m, 7H), 3.77 – 3.62 (m, 11H), 3.54 – 3.39 (m, 15H), 3.36 (t, *J* = 9.5 Hz, 1H), 3.31 (t, *J* = 8.7 Hz, 1H), 2.96 (t, *J* = 7.6 Hz, 2H), 1.64 (q, *J* = 7.7 Hz, 5H), 1.42 (p, *J* = 8.0 Hz, 2H).

^{13}C NMR (176 MHz, D_2O) δ 102.73, 102.46, 102.44, 102.43, 101.84, 84.25, 84.05, 83.86, 75.92, 75.56, 75.54, 75.53, 75.48, 75.44, 73.34, 73.22, 73.17, 72.84, 70.02, 69.48, 68.06, 68.01, 67.97, 60.59, 60.57, 39.26, 28.07, 26.33, 22.00.

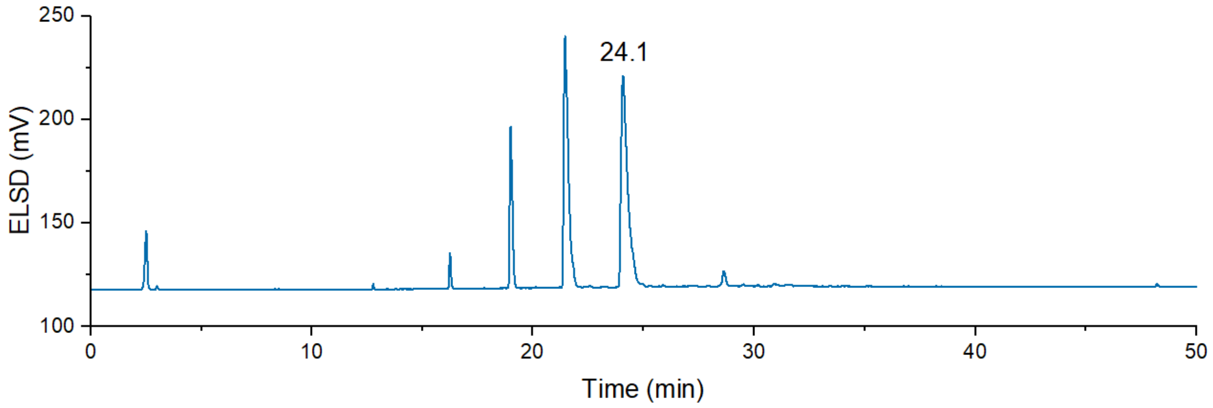

Figure S37. RP-HPLC trace of crude **19**.

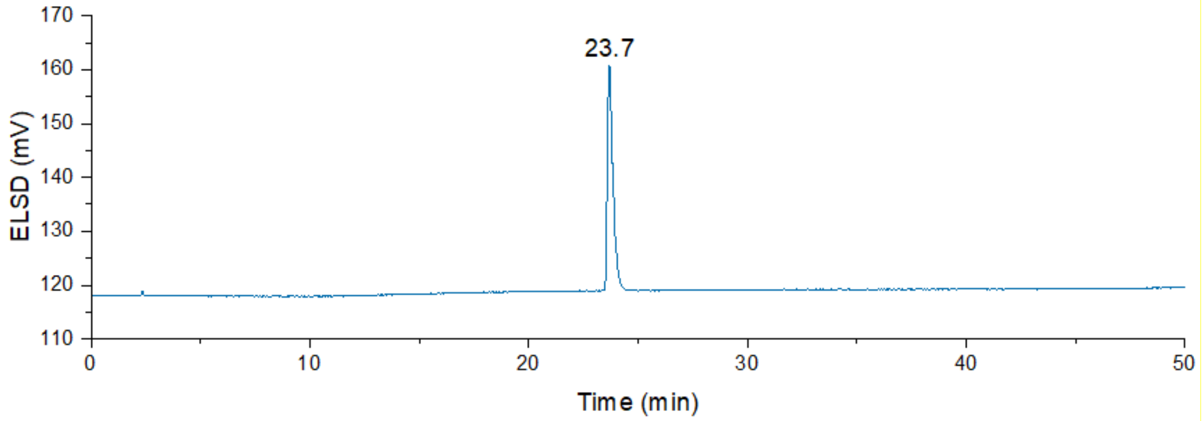

Figure S38. RP-HPLC trace of pure **19**.

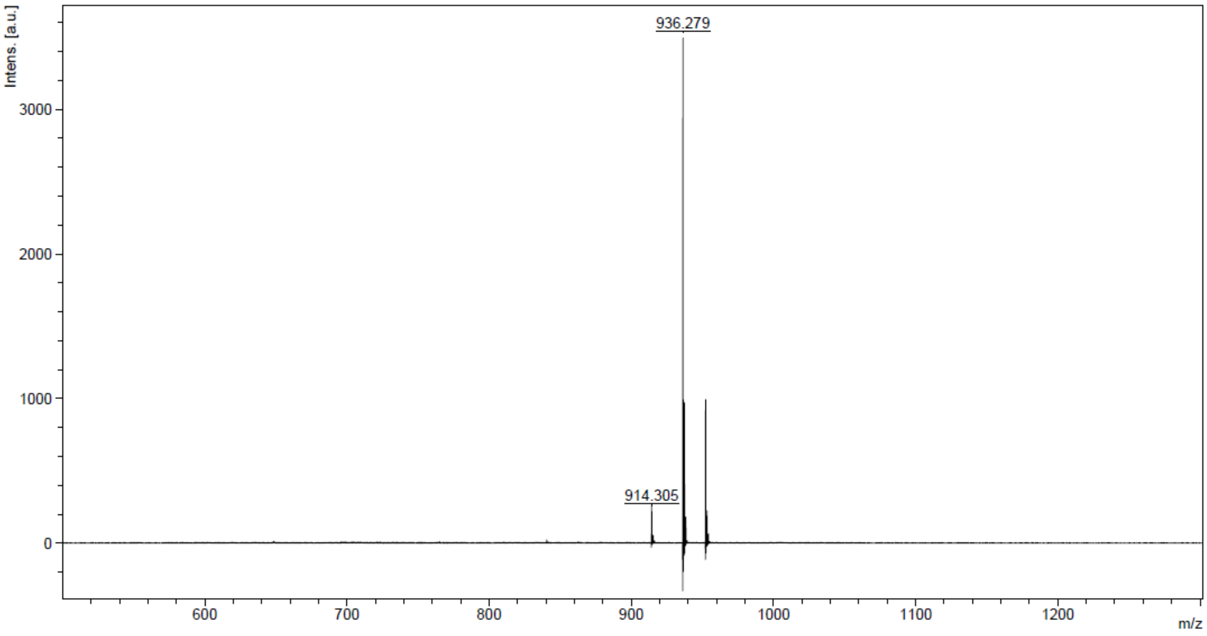

Figure S39. MALDI-TOF of **19**.

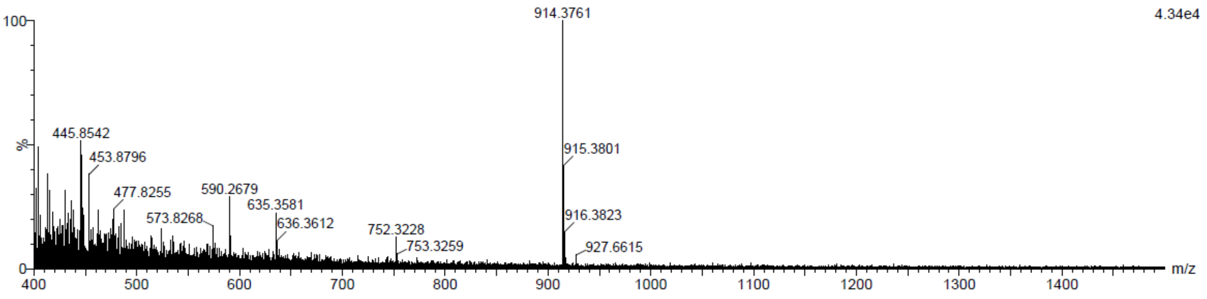

Figure S40. HR-MS of **19**.

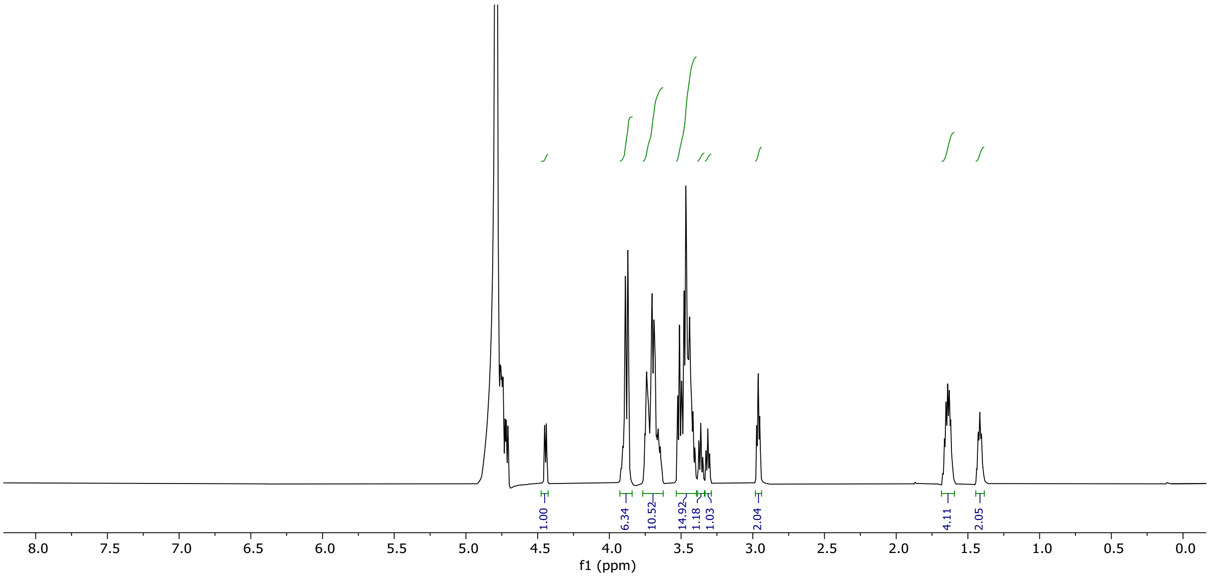

Figure S41. ^{1}H NMR (700MHz, D_2O) spectrum of **19**.

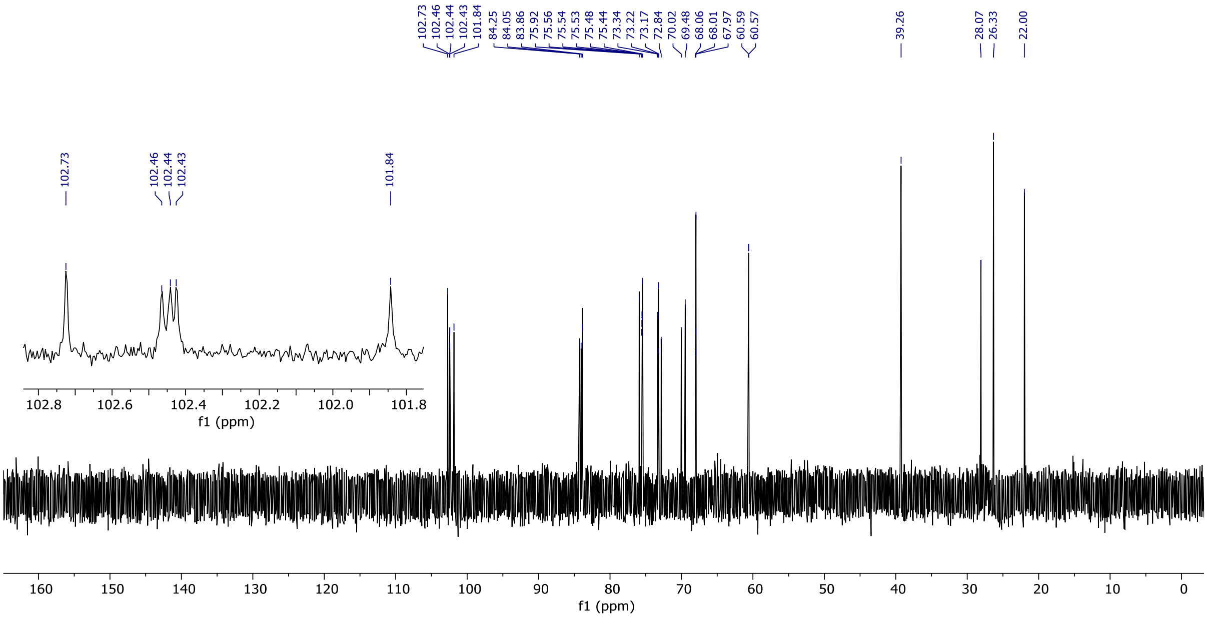

Figure S42. ^{13}C NMR (176MHz, D_2O) spectrum of **19**.

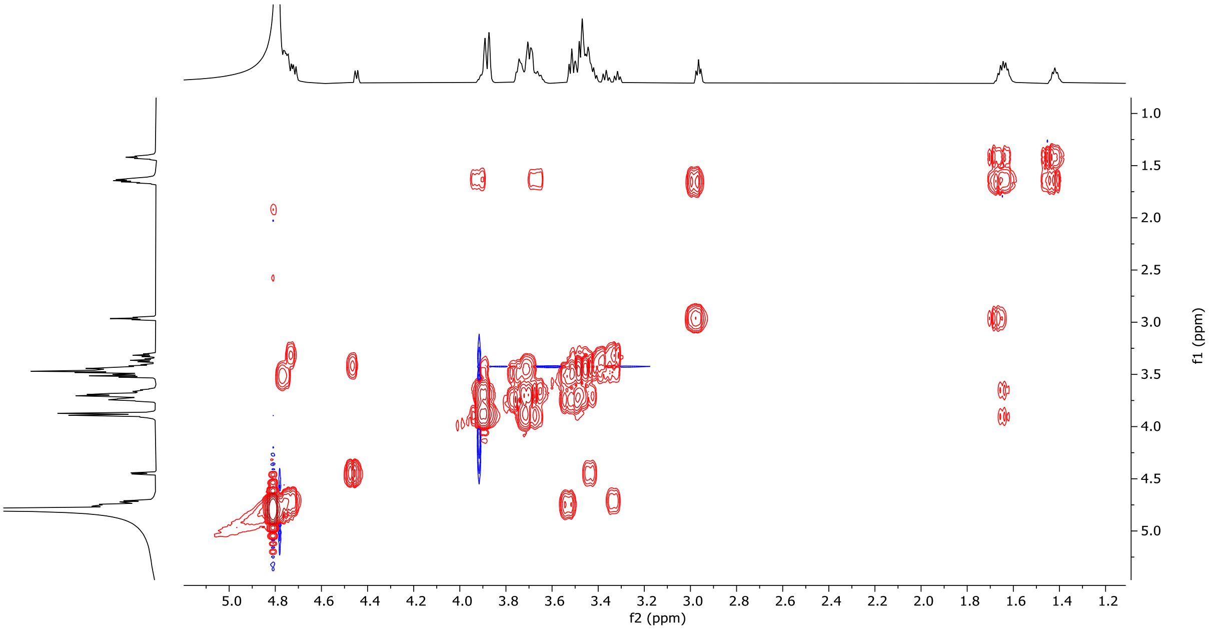

Figure S43. COSY NMR (700MHz, D_2O) spectrum of **19**.

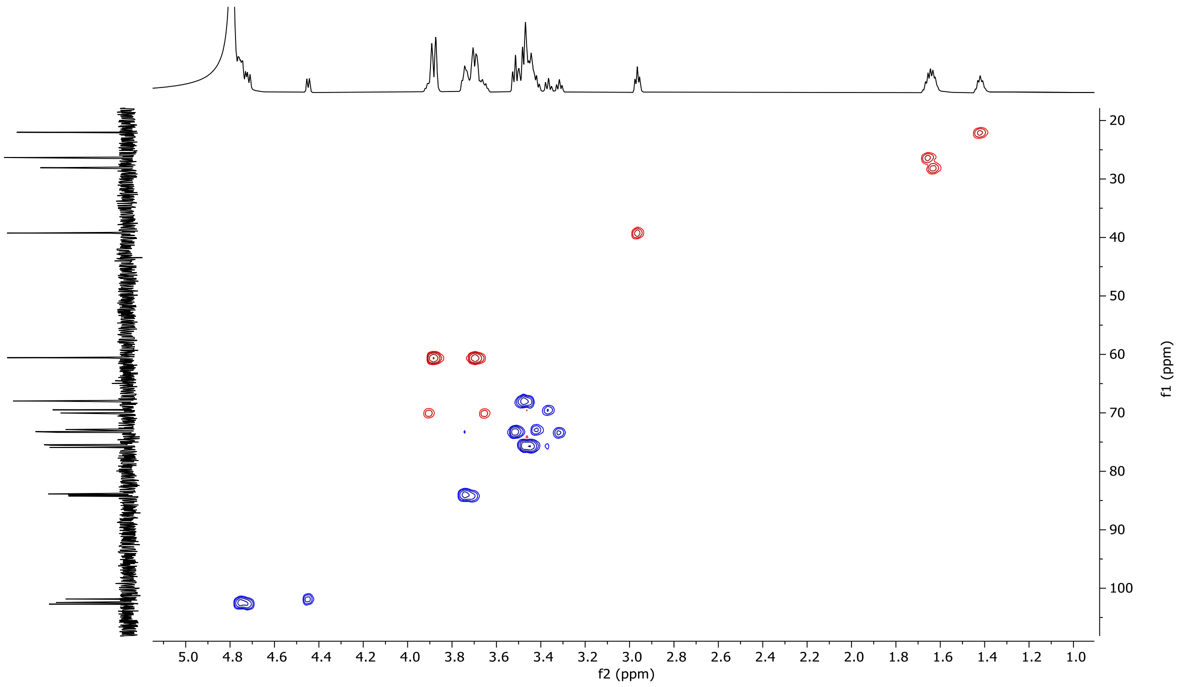

Figure S44. HSQC NMR (700MHz, D_2O) spectrum of **19**.

Synthesis and analytical data of 16

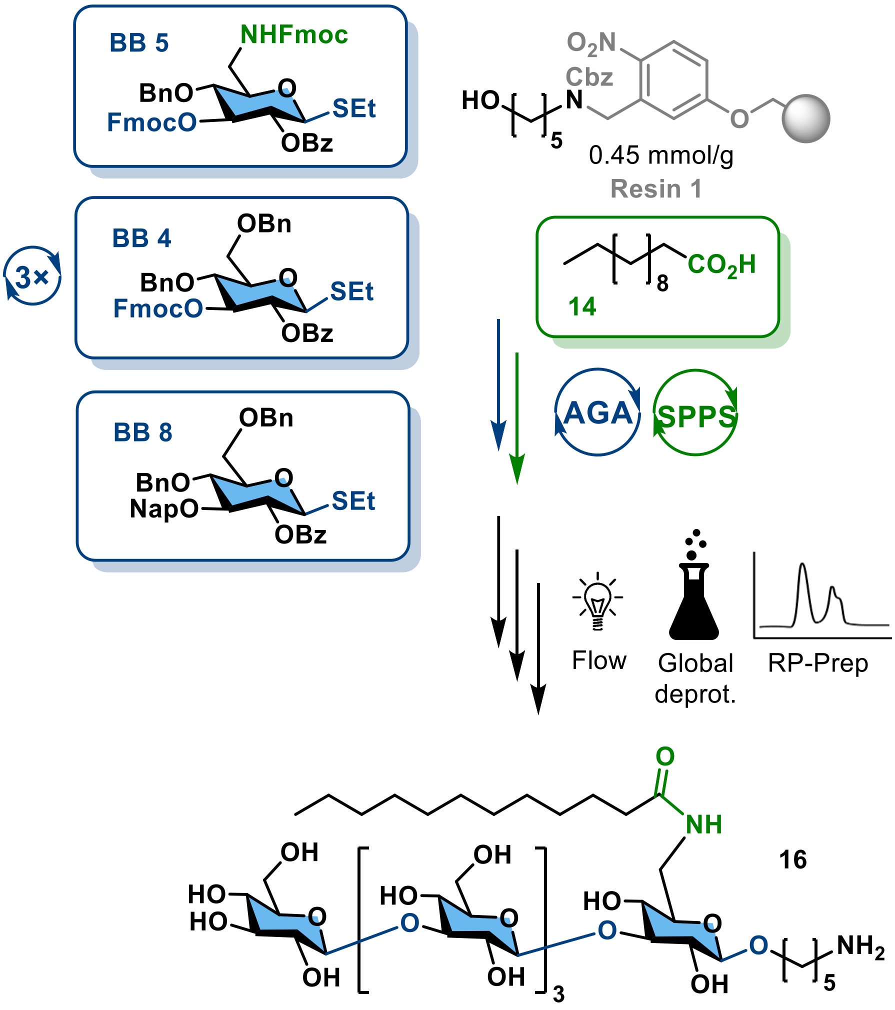

Step	Module	BB/reagent	Repeat	Notes
AGA	A	**1** (0.015 mmol)	1	0.45 mmol/g
	B, C1, D1	**5** (0.10 mmol)	1	-
	B, C1, D1	**4** (0.10 mmol)	3	-
	B, C1	**8** (0.10 mmol)	1	-
SPPS	E	-	3	-
	F	**14**	-	-
Post solid-phase	K1	-	-	-
	M	-	3	-
	N1	-	-	-
	O	-	-	Methods 3 and 6

After automated glycan assembly, side-chain attachment, methanolysis, photo-cleavage, hydrogenolysis, purification, and lyophilization **16** was obtained as a white solid (2.3 mg, 14%).

R_t (Method 3) = 20.0 min.

HRMS (ESI/Q-TOF) *m/z*: $[M + H]^+$ Calcd for $C_{47}H_{87}N_2O_{26}$ 1065.5542; Found 1095.5604.

^{1}H NMR (400 MHz, D_2O) δ 4.77 – 4.69 (m, 4H), 4.41 (d, *J* = 8.0 Hz, 1H), 3.90 (d, *J* = 12.4 Hz, 5H), 3.80 – 3.58 (m, 11H), 3.57 – 3.29 (m, 18H), 2.97 (t, *J* = 6.8 Hz, 2H), 2.24 (t, *J* = 5.7 Hz, 2H), 1.65 (dd, *J* = 15.6, 7.8 Hz, 4H), 1.60 – 1.51 (m, 2H), 1.42 (dt, *J* = 15.0, 7.6 Hz, 2H), 1.26 (s, 18H), 0.84 (t, *J* = 6.1 Hz, 3H).

^{13}C NMR (176 MHz, D_2O) δ 177.43, 102.75, 102.48, 102.45, 102.42, 101.81, 84.12, 84.01, 83.94, 75.95, 75.61, 75.56, 75.48, 73.76, 73.37, 73.24, 73.18, 72.90, 69.94, 69.76, 69.50, 68.03, 68.01, 60.62, 60.61, 40.02, 39.33, 35.73, 31.19, 28.75, 28.72, 28.57, 28.53, 28.35, 28.19, 28.03, 26.51, 25.40, 23.19, 22.18, 22.05, 13.42.

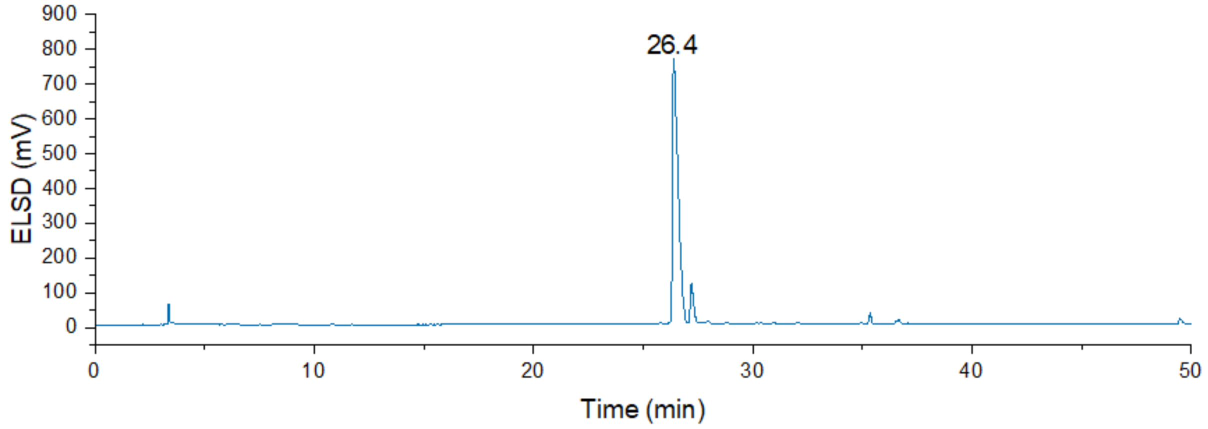

Figure S45. RP-HPLC trace of crude **16**.

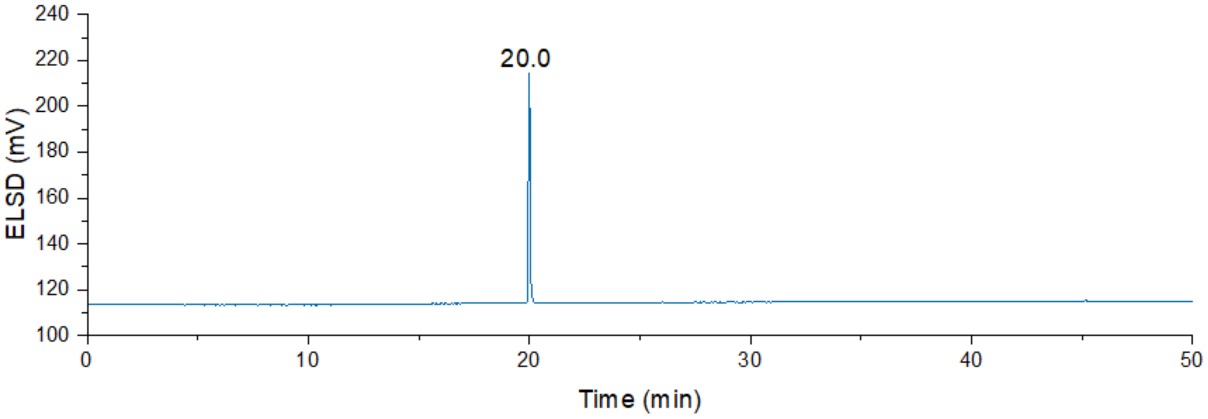

Figure S46. RP-HPLC trace of pure **16**.

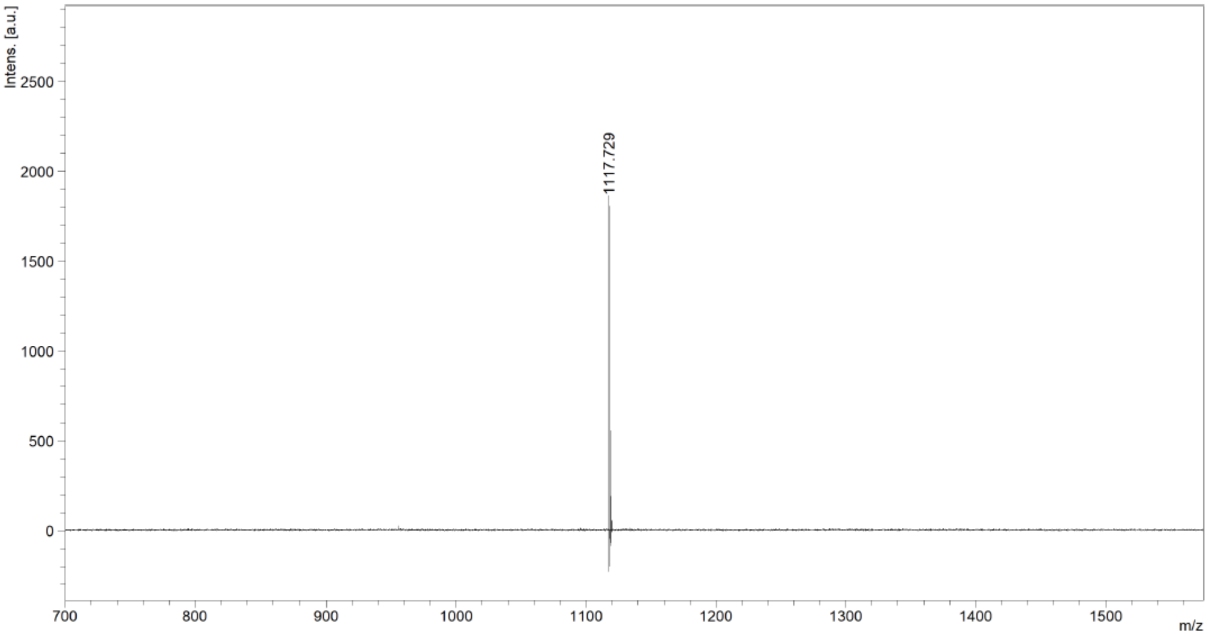

Figure S47. MALDI-TOF of **16**.

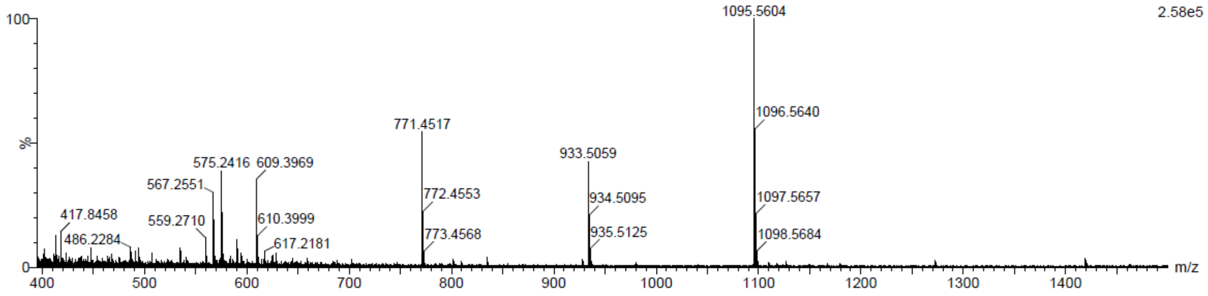

Figure S48. HR-MS of **16**.

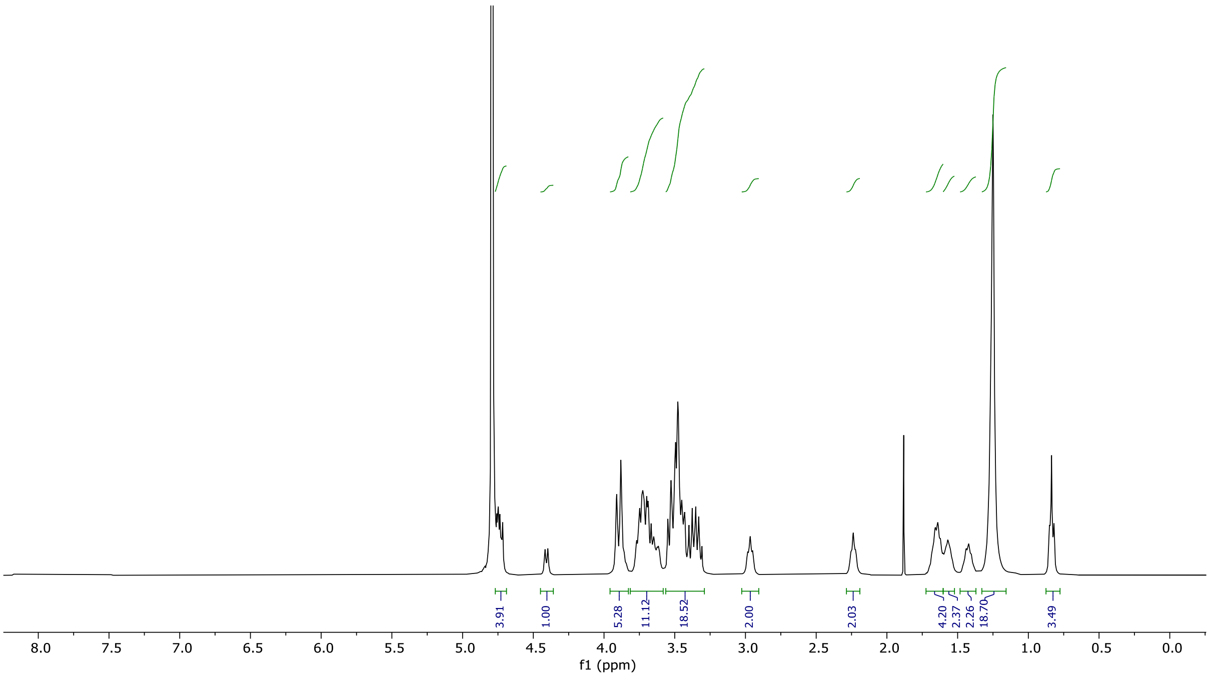

Figure S49. ^{1}H NMR (400MHz, D_2O) spectrum of **16**.

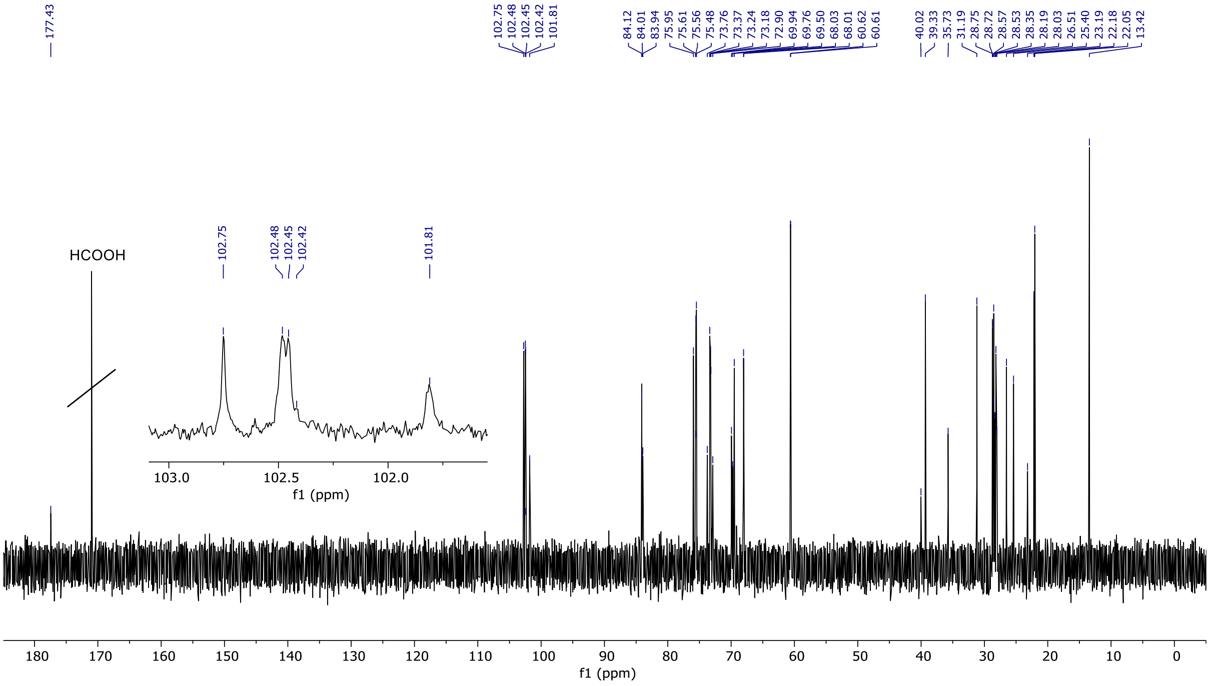

Figure S50. ^{13}C NMR (176MHz, D_2O) spectrum of **16**.

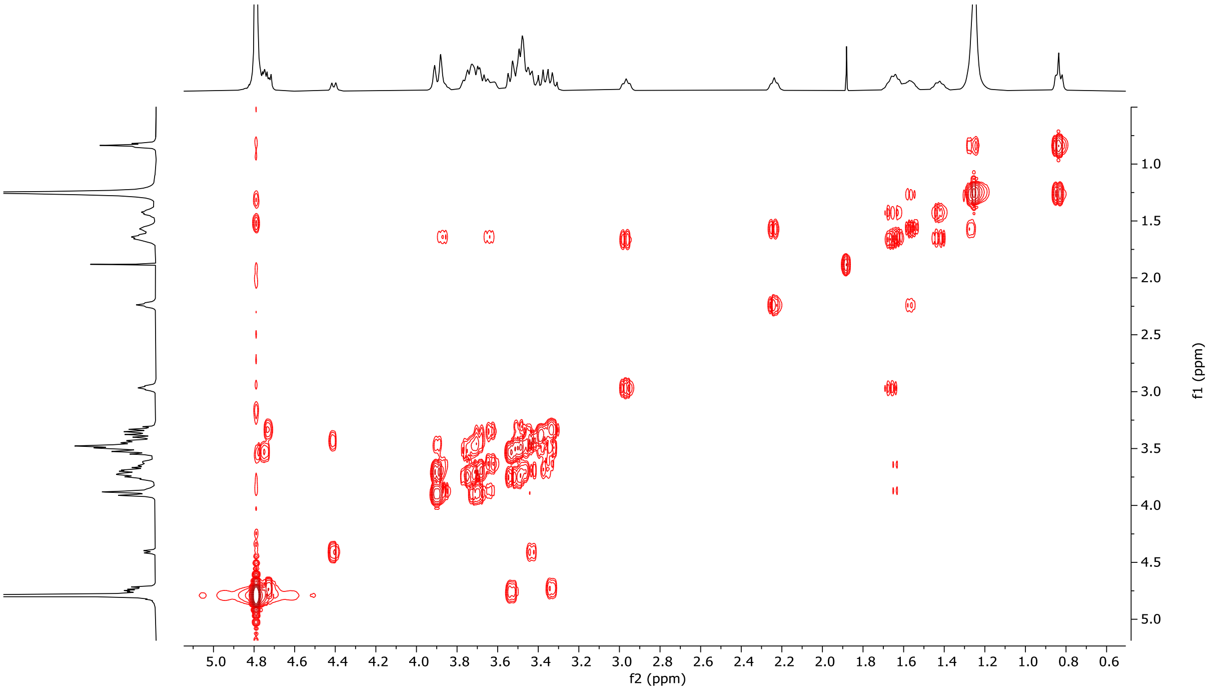

Figure S51. COSY NMR (700MHz, D_2O) spectrum of **16**.

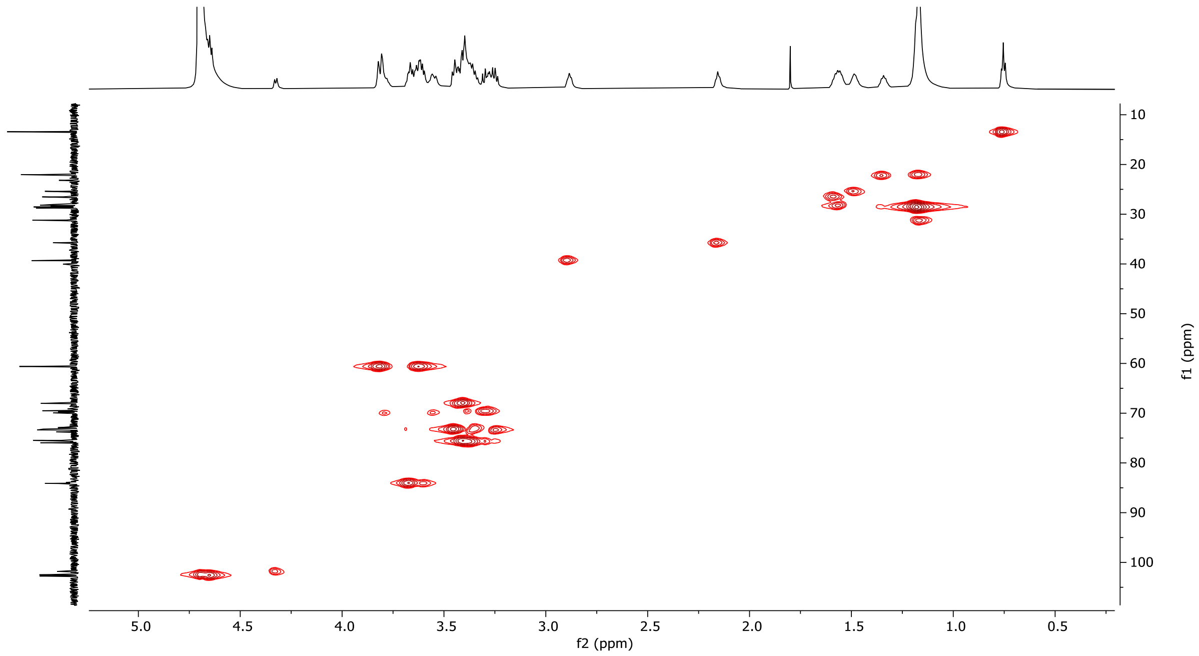

Figure S52. HSQC NMR (700MHz, D_2O) spectrum of **16**.

Synthesis and analytical data of 17

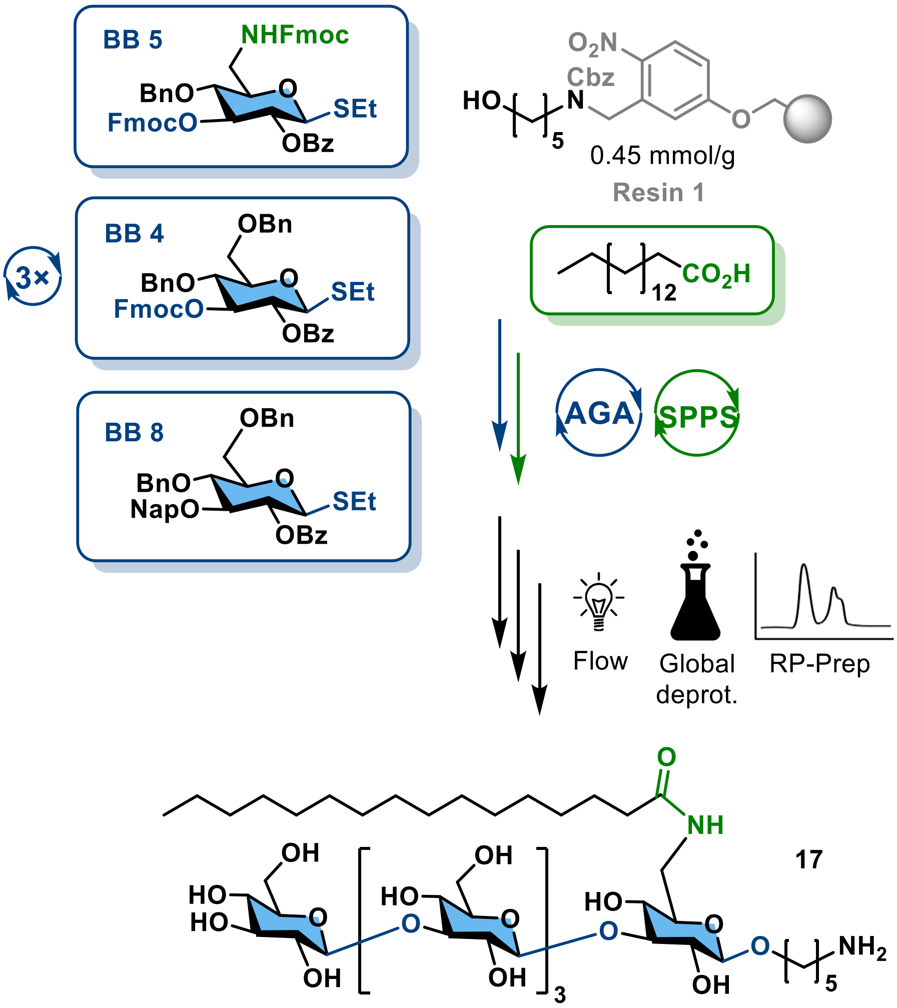

Step	Module	BB/reagent	Repeat	Notes
AGA	A	**1** (0.015 mmol)	1	0.45 mmol/g
	B, C1, D1	**5** (0.10 mmol)	1	-
	B, C1, D1	**4** (0.10 mmol)	3	-
	B, C1	**8** (0.10 mmol)	1	-
SPPS	E	-	3	-
	F	Palmitic acid	-	-
Post solid-phase	K1	-	-	-
	M	-	3	-
	N1	-	-	-
	O	-	-	Methods 3 and 6

After automated glycan assembly, side-chain attachment, methanolysis, photo-cleavage, hydrogenolysis, purification, and lyophilization **17** was obtained as a white solid (2.4 mg, 13%).

R_t (Method 3) = 23.0 min.

HRMS (ESI/Q-TOF) *m/z*: $[M + H]^+$ Calcd for $C_{51}H_{95}N_2O_{26}$ 1151.6168; Found 1151.6235.

^{1}H NMR (400 MHz, CD_3OD) δ 4.69 – 4.60 (m, 3H), 4.57 (d, *J* = 7.8 Hz, 1H), 4.28 (d, *J* = 7.9 Hz, 1H), 3.93 – 3.83 (m, 5H), 3.71 – 3.52 (m, 10H), 3.51 – 3.44 (m, 4H), 3.42 (t, *J* = 3.4 Hz, 1H), 3.41 – 3.33 (m, 11H), 3.29 – 3.22 (m, 3H), 2.87 (dd, *J* = 8.6, 6.4 Hz, 2H), 2.21 (t, *J* = 7.1 Hz, 2H), 1.74 – 1.55 (m, 8H), 1.55 – 1.40 (m, 3H), 1.30 (d, *J* = 10.0 Hz, 37H), 0.90 (t, *J* = 6.7 Hz, 3H).

^{13}C NMR (101 MHz, CD_3OD) δ 176.65, 105.19, 104.73, 104.71, 104.68, 103.78, 87.46, 87.29, 87.25, 87.14, 78.19, 77.88, 77.81, 75.67, 75.51, 75.08, 74.67, 71.63, 71.57, 70.00, 69.90, 62.64, 62.56, 41.60, 40.72, 37.14, 33.10, 30.83, 30.80, 30.69, 30.54, 30.51, 30.37, 30.35, 29.99, 28.14, 27.19, 23.99, 23.76, 14.47.

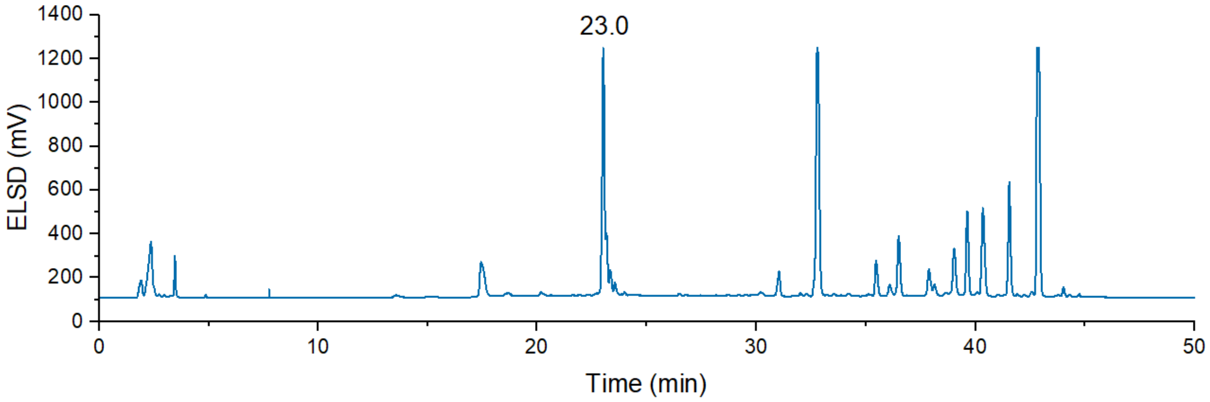

Figure S53. RP-HPLC trace of crude **17**.

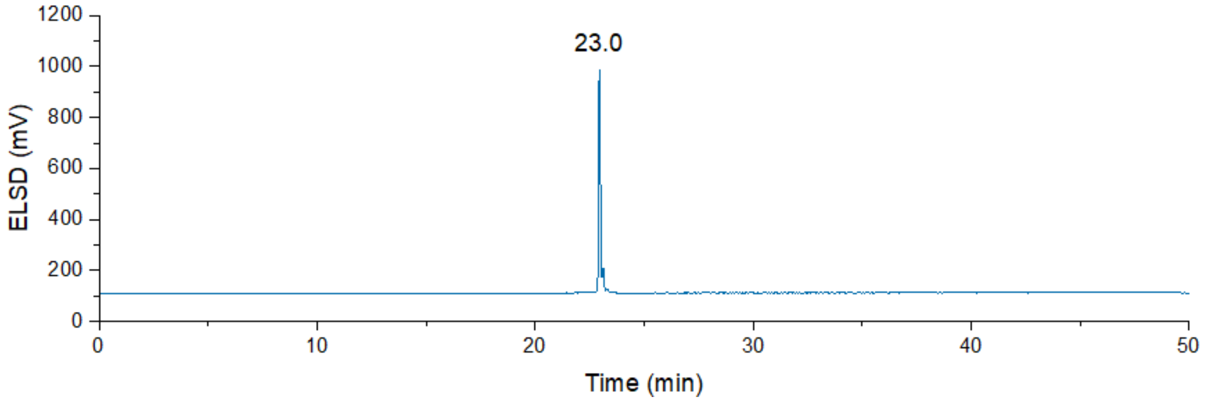

Figure S54. RP-HPLC trace of pure **17**.

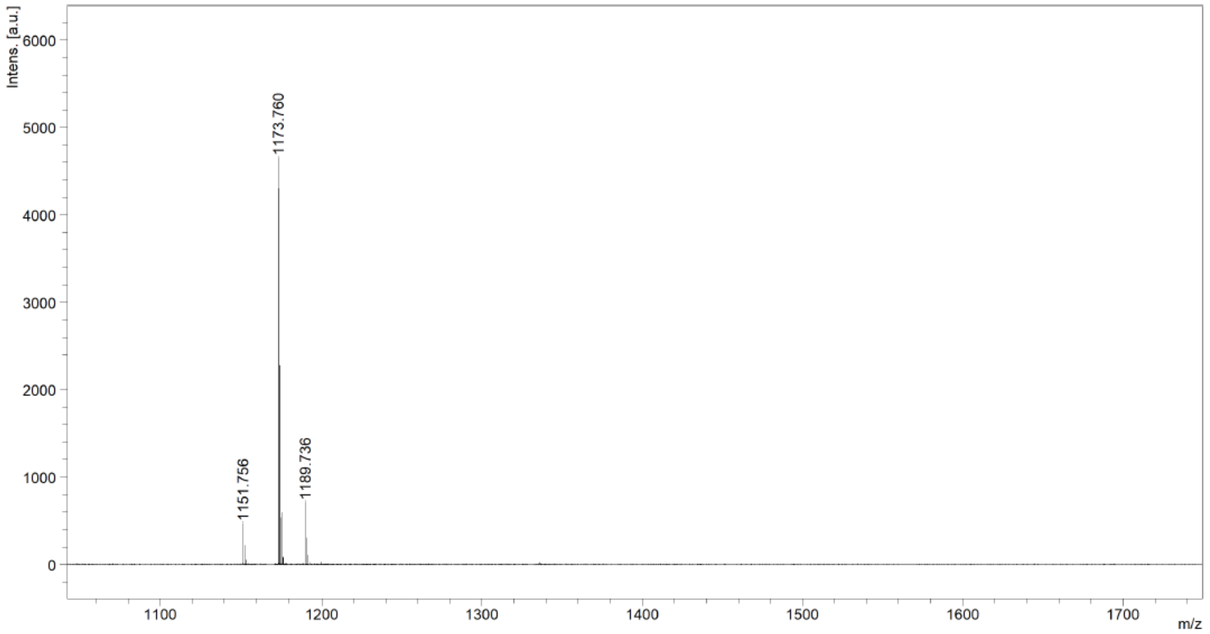

Figure S55. MALDI-TOF of **17**.

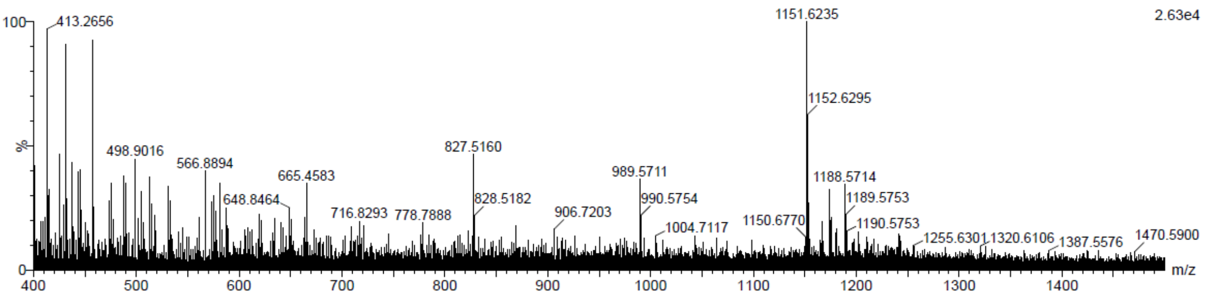

Figure S56. HR-MS of **17**.

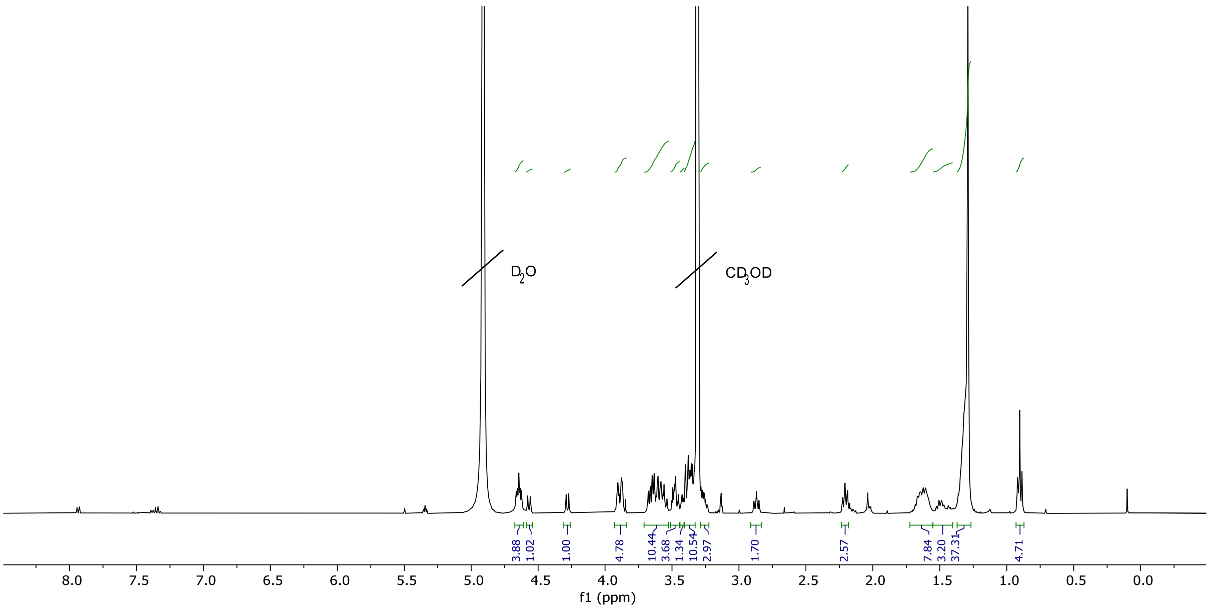

Figure S57. ^{1}H NMR (700MHz, CD_3OD) spectrum of **17**.

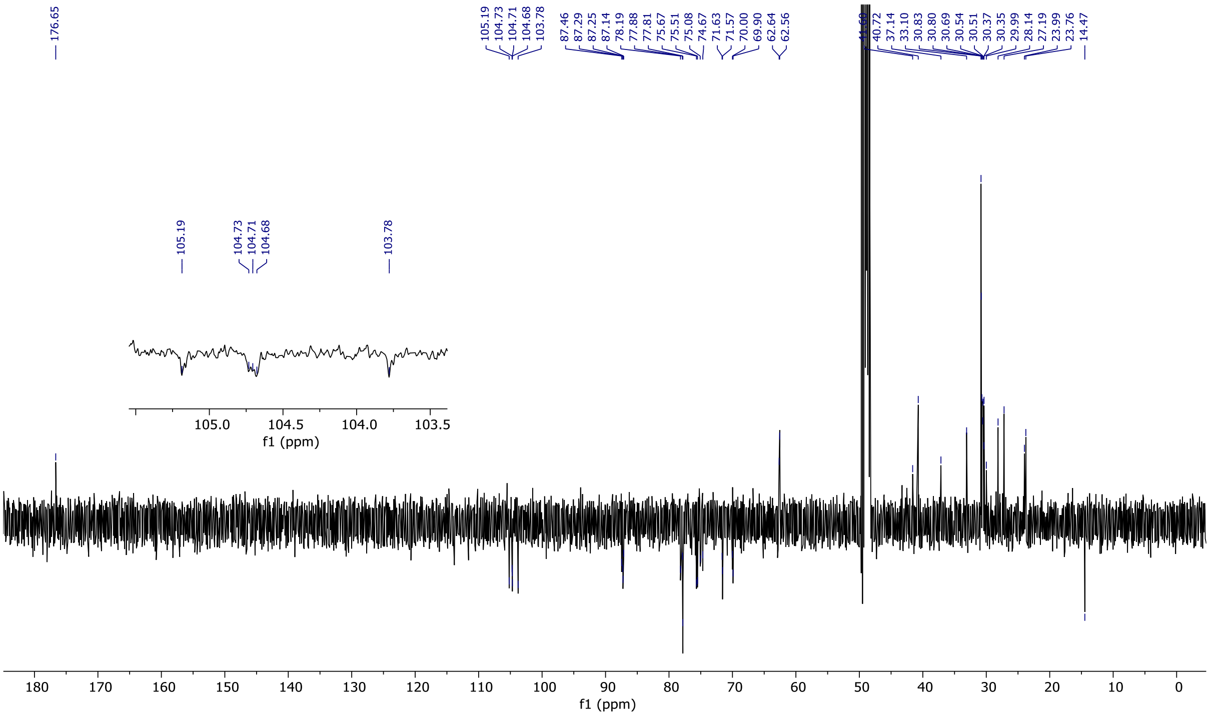

Figure S58. ^{13}C APT NMR (101MHz, CD_3OD) spectrum of **17**.

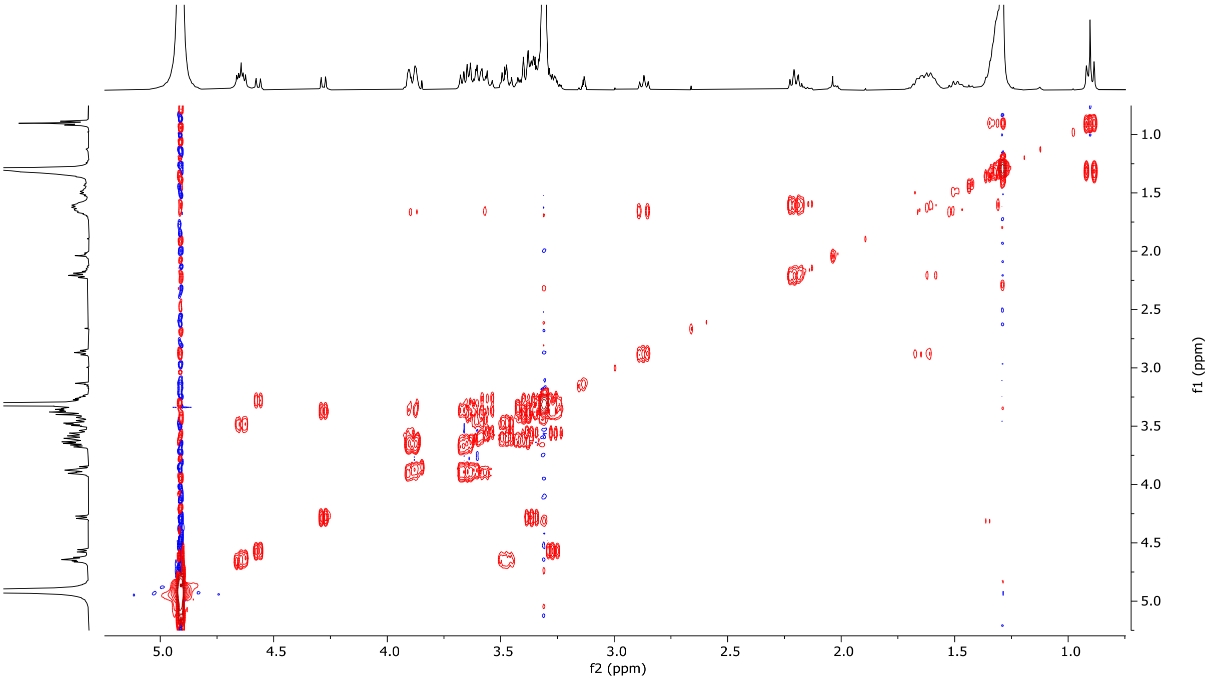

Figure S59. COSY NMR (400MHz, CD_3OD) spectrum of **17**.

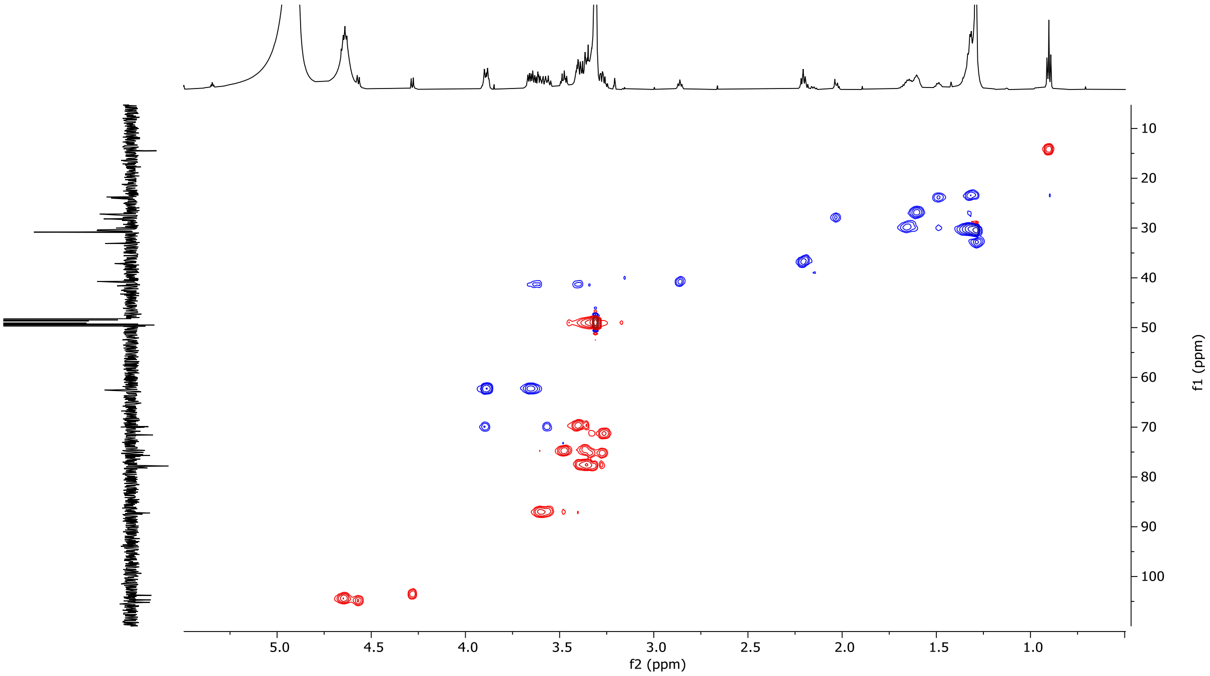

Figure S60. HSQC NMR (700MHz, CD_3OD) spectrum of **17**.

Synthesis and analytical data of 15

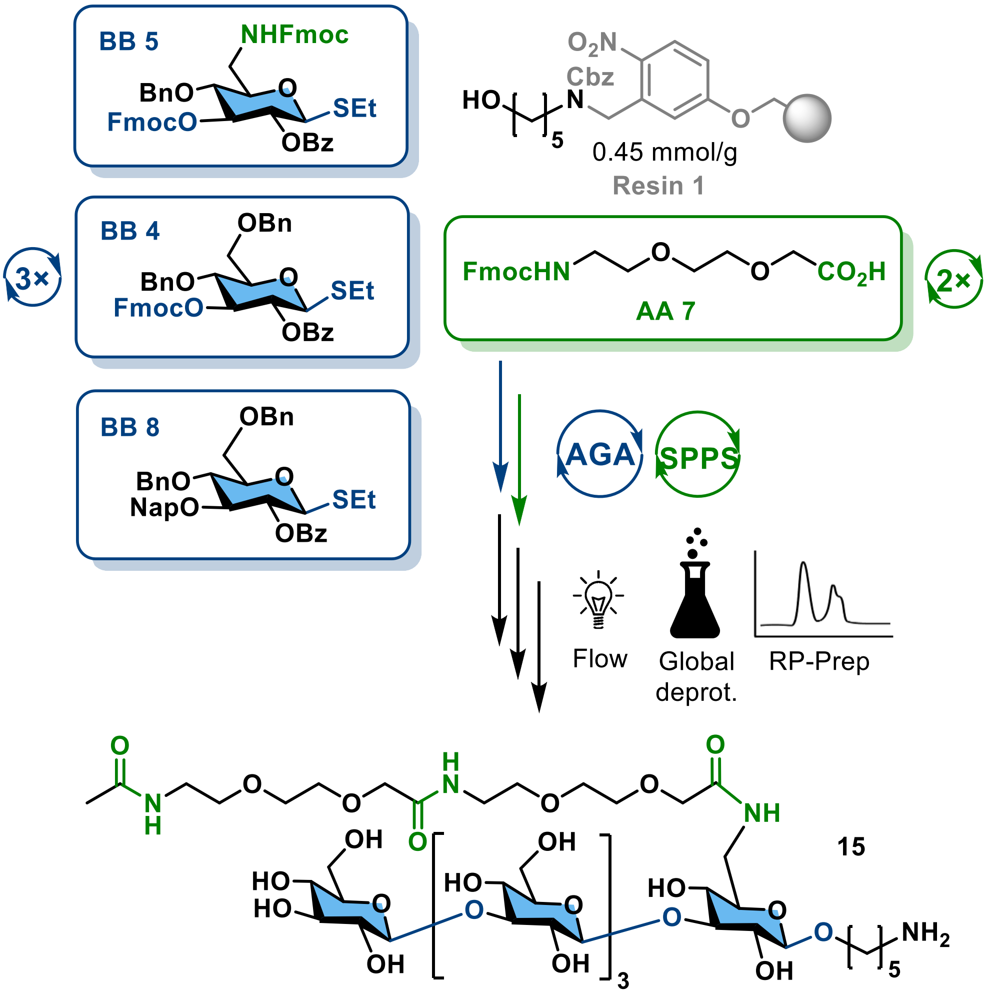

Step	Module	BB/reagent	Repeat	Notes
AGA	A	**1** (0.015 mmol)	1	0.45 mmol/g
	B, C1, D1	**5** (0.10 mmol)	1	-
	B, C1, D1	**4** (0.10 mmol)	3	-
	B, C1	**8** (0.10 mmol)	1	-
SPPS	E, F	**7**	2	-
	G	-	-	-
Post solid-phase	K1	-	-	-
	M	-	3	-
	N1	-	-	-
	O	-	-	Methods 1 and 3

After automated glycan assembly, side-chain construction, methanolysis, photo-cleavage, hydrogenolysis, purification, and lyophilization **15** was obtained as a white solid (3.2 mg, 19%).

R_t (Method 1) = 29.1 min.

HRMS (ESI/Q-TOF) *m/z*: $[M + H]^+$ Calcd for $C_{49}H_{89}N_4O_{32}$ 1245.5454; Found 1245.5516.

^1H NMR (700 MHz, D_2O) δ 4.41 (dd, *J* = 8.2, 2.0 Hz, 1H), 4.05 (dd, *J* = 5.3, 2.4 Hz, 4H), 3.91 – 3.85 (m, 5H), 3.83 (dt, *J* = 9.0, 6.5 Hz, 1H), 3.76 – 3.71 (m, 3H), 3.71 – 3.65 (m, 13H), 3.65 – 3.61 (m, 4H), 3.60 (t, *J* = 5.4 Hz, 2H), 3.53 – 3.39 (m, 16H), 3.36 (ddd, *J* = 16.0, 8.2, 3.6 Hz, 4H), 3.31 (ddd, *J* = 9.5, 8.3, 2.1 Hz, 1H), 2.95 (t, *J* = 7.6 Hz, 2H), 1.95 (s, 3H), 1.62 (dp, *J* = 21.9, 7.1 Hz, 5H), 1.40 (p, *J* = 7.4 Hz, 2H).

^{13}C NMR (176 MHz, D_2O) δ 174.19, 172.67, 172.59, 102.73, 102.47, 102.45, 102.43, 101.79, 84.06, 83.94, 83.90, 83.86, 75.92, 75.54, 75.45, 73.55, 73.35, 73.22, 73.17, 72.88, 70.37, 70.26, 69.90, 69.69, 69.48, 69.42, 69.29, 69.27, 68.73, 68.01, 60.60, 39.59, 39.27, 38.90, 38.35, 28.13, 26.35, 22.11, 21.75.

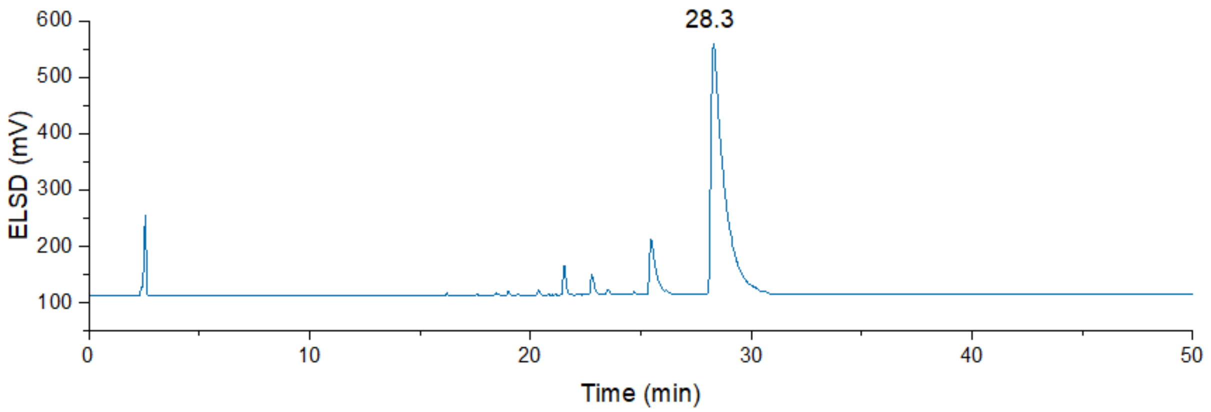

Figure S61. RP-HPLC trace of crude **15**.

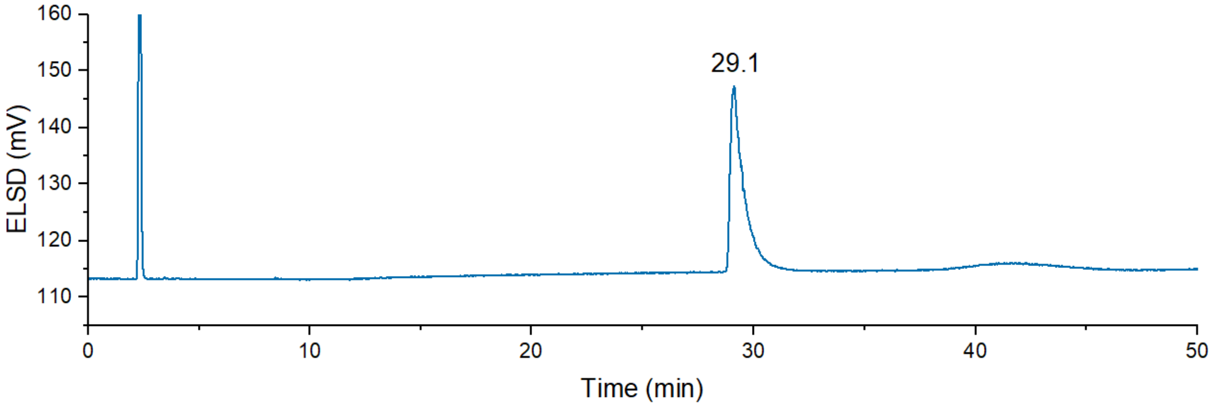

Figure S62. RP-HPLC trace of pure **15**.

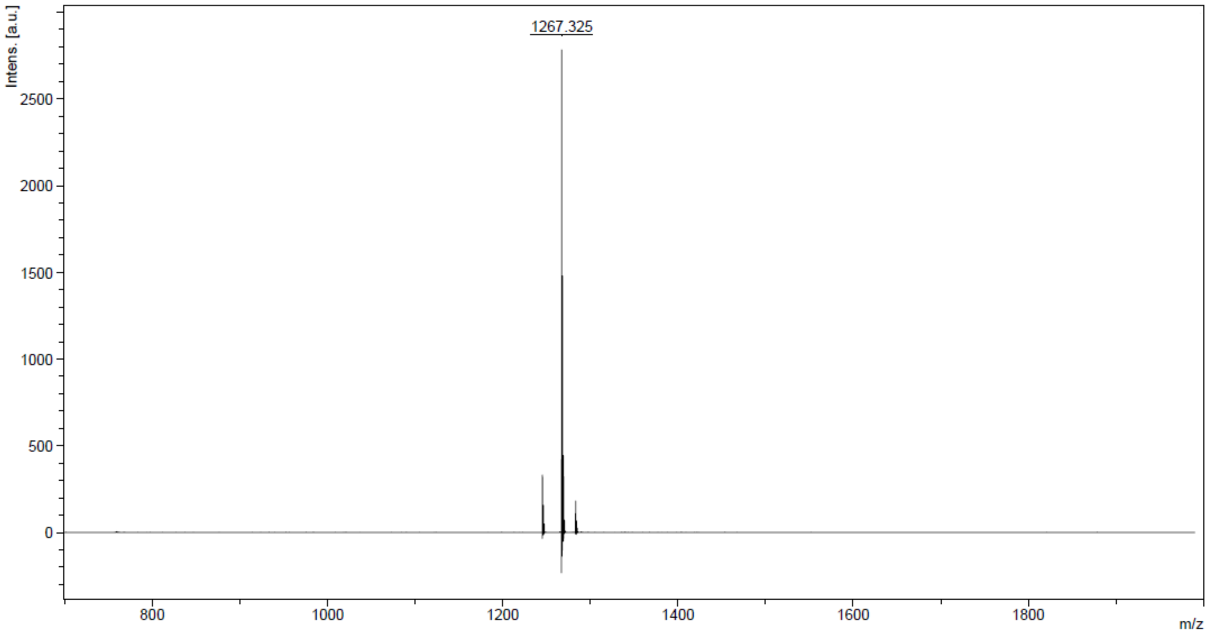

Figure S63. MALDI-TOF of **15**.

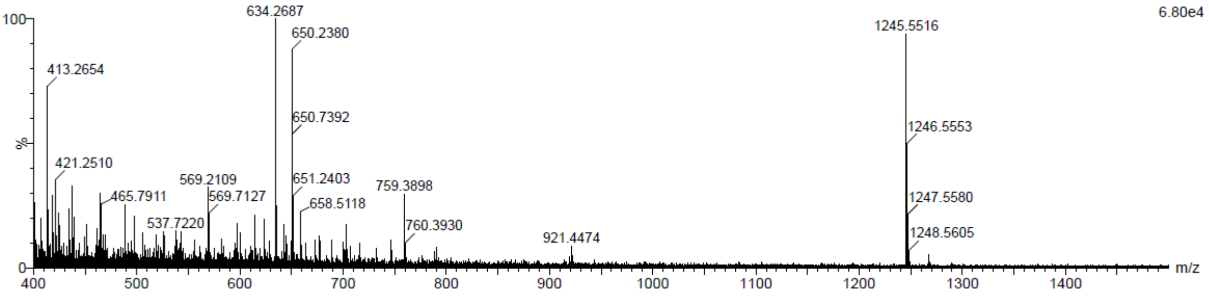

Figure S64. HR-MS of **15**.

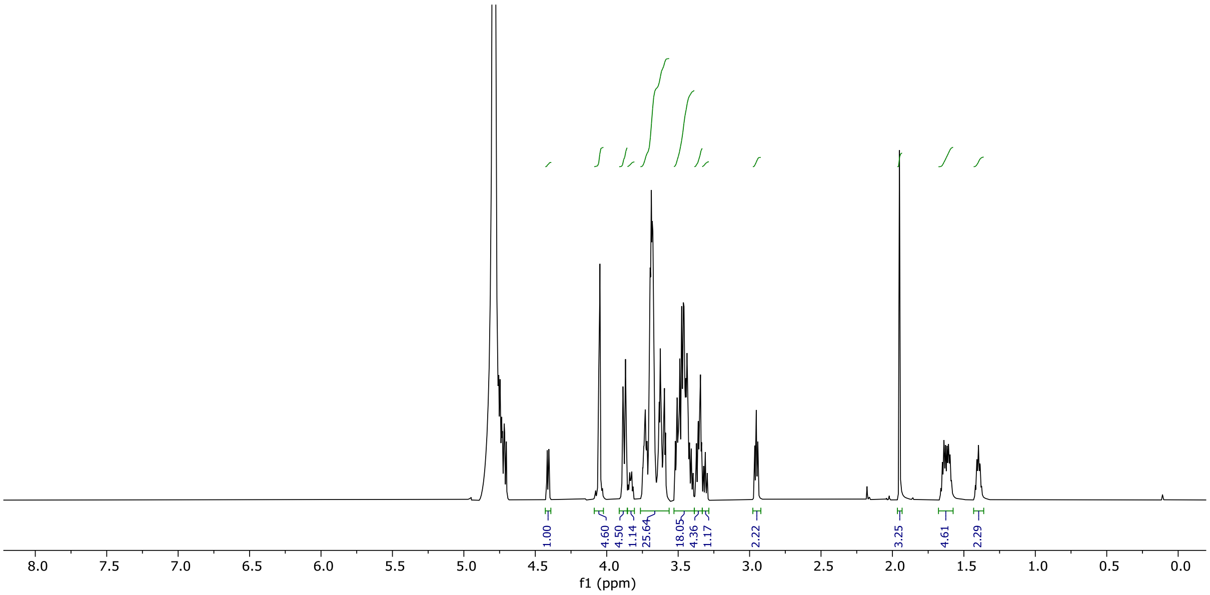

Figure S65. ^{1}H NMR (700MHz, D_2O) spectrum of **15**.

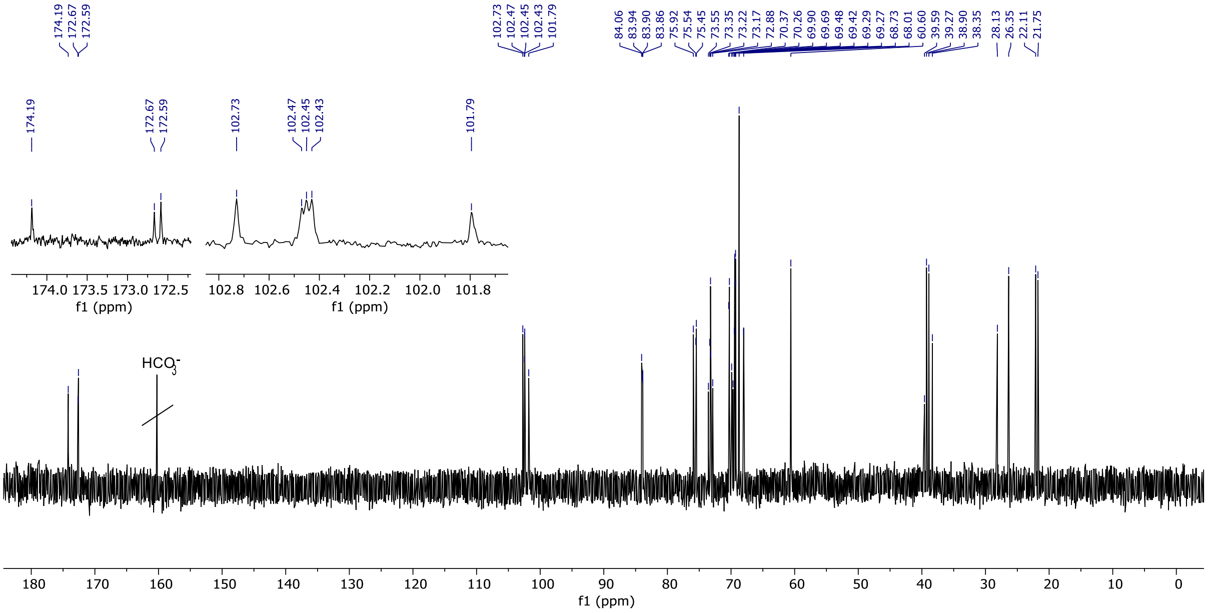

Figure S66. ^{13}C NMR (176MHz, D_2O) spectrum of **15**.

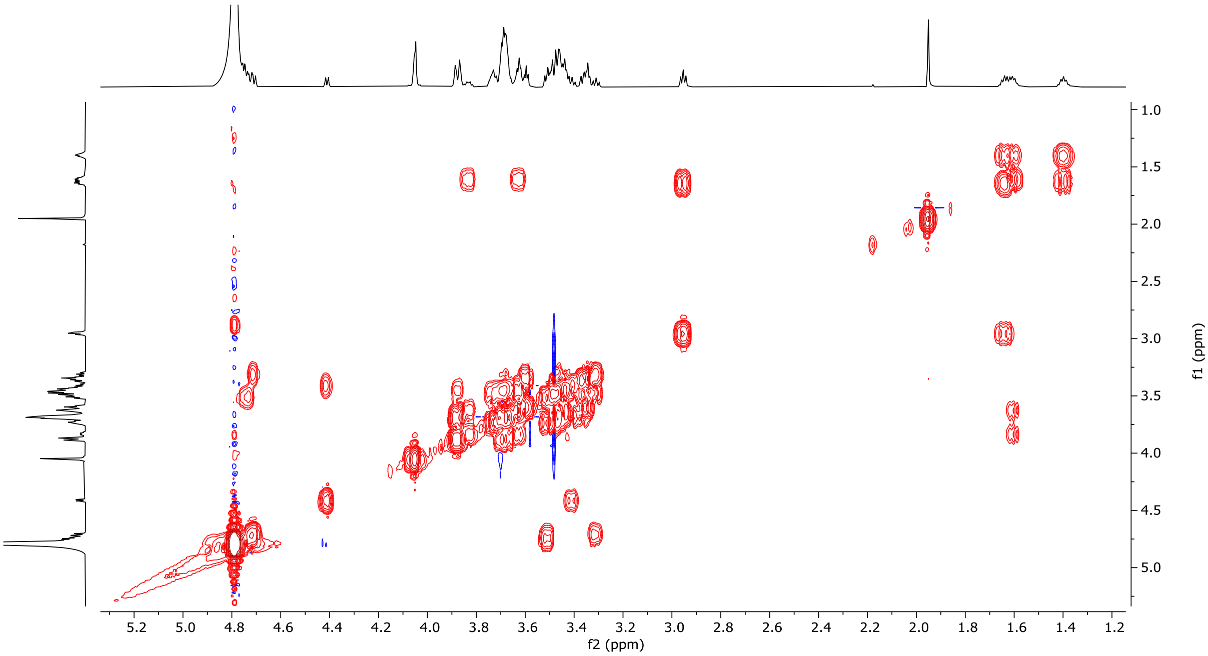

Figure S67. COSY NMR (700MHz, D_2O) spectrum of **15**.

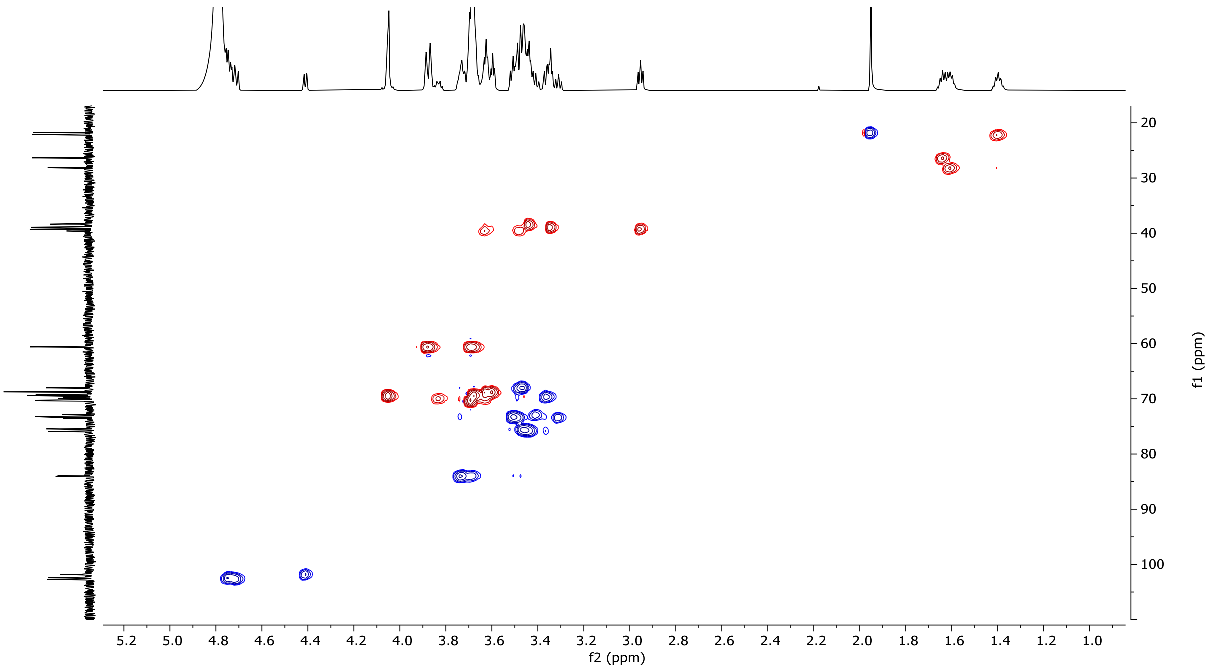

Figure S68. HSQC NMR (700MHz, D_2O) spectrum of **15**.

Synthesis and analytical data of 13

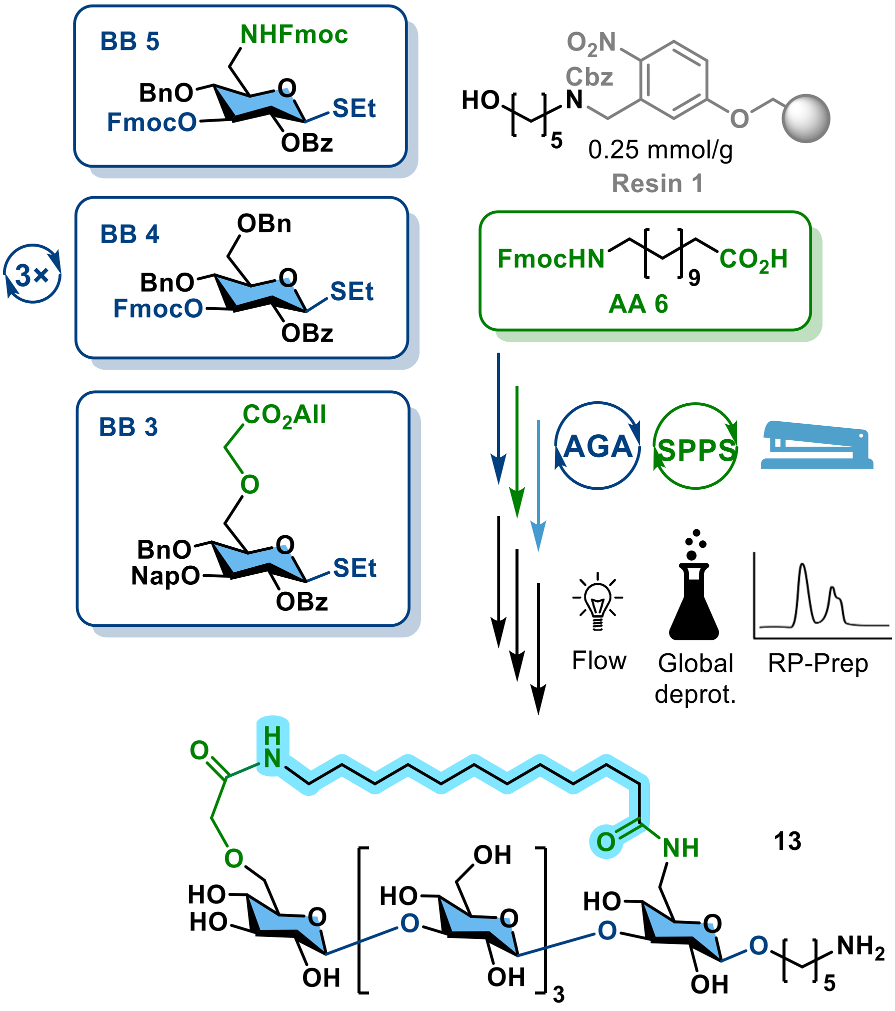

Step	Module	BB/reagent	Repeat	Notes
AGA	A	**1** (0.015 mmol)	1	0.25 mmol/g
	B, C1, D1	**5** (0.10 mmol)	1	-
	B, C1, D1	**4** (0.10 mmol)	3	-
	B, C1	**3** (0.10 mmol)	1	-
SPPS	E, F	**6**	1	-
	I, E	-	-	-
Stapling	J	-	-	-
Post solid-phase	M	-	3	-
	K2	-	-	-
	N2	-	-	-
	O	-	-	Methods 3 and 6

After automated glycan assembly, side-chain attachment, on-resin stapling, photo-cleavage, global deprotection, purification, and lyophilization **13** was obtained as a white solid (1.3 mg, 8%).

R_t (Method 3) = 18.7 min.

HRMS (ESI/Q-TOF) *m/z*: $[M + H]^+$ Calcd for $C_{49}H_{88}N_3O_{27}$ 1150.5600; Found 1150.5686.

^{1}H NMR (700 MHz, D_2O/CD_3CN = 3/2) δ 5.13 (d, *J* = 8.0 Hz, 1H), 5.08 – 5.02 (m, 3H), 4.35 (q, *J* = 15.2 Hz, 2H), 4.25 – 4.12 (m, 5H), 4.08 (t, *J* = 8.9 Hz, 1H), 4.04 – 3.85 (m, 9H), 3.83 – 3.76 (m, 3H), 3.76 – 3.71 (m, 6H), 3.68 (dd, *J* = 14.6, 3.1 Hz, 1H), 3.67 – 3.55 (m, 3H), 3.44 (dt, *J* = 14.0, 7.3 Hz, 1H), 3.25 (t, *J* = 7.6 Hz, 2H), 2.60 – 2.49 (m, 2H), 2.01 – 1.84 (m, 7H), 1.85 – 1.77 (m, 2H), 1.76 – 1.69 (m, 2H), 1.60 (s, 19H).

^{13}C NMR (176 MHz, D_2O/CD_3CN = 3/2) δ 177.15, 177.10, 103.27, 102.42, 102.25, 102.19, 101.97, 85.20, 83.87, 83.73, 83.11, 76.40, 76.33, 76.01, 75.52, 74.16, 73.98, 73.46, 73.27, 70.49, 70.40, 69.72, 68.54, 68.35, 68.27, 61.27, 39.68, 39.40, 38.93, 36.17, 29.86, 29.69, 29.54, 29.47, 28.86, 28.71, 26.98, 26.78, 26.16, 22.59.

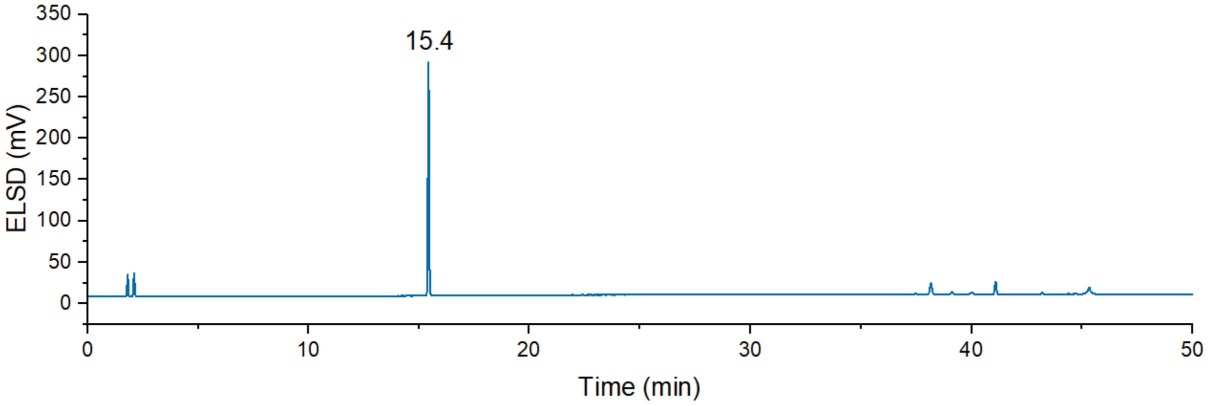

Figure S69. RP-HPLC trace of crude **13**.

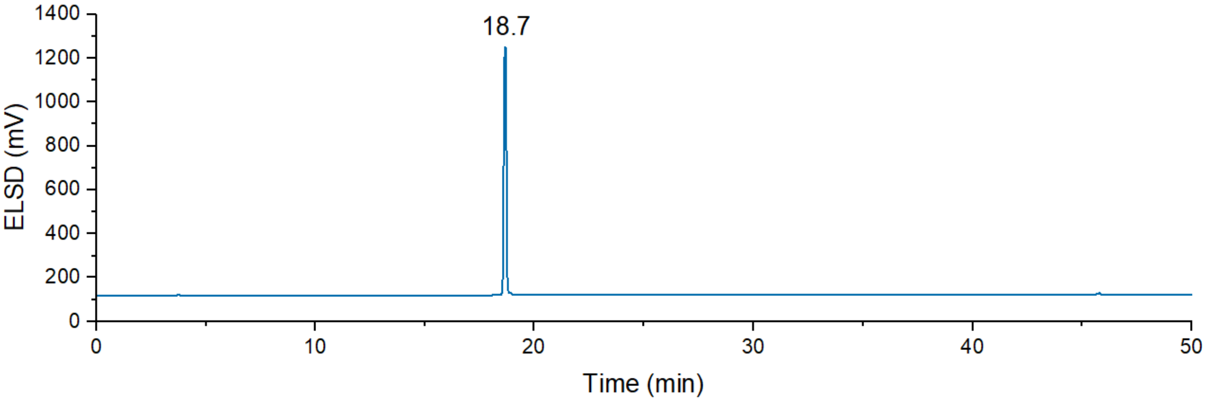

Figure S70. RP-HPLC trace of pure **13**.

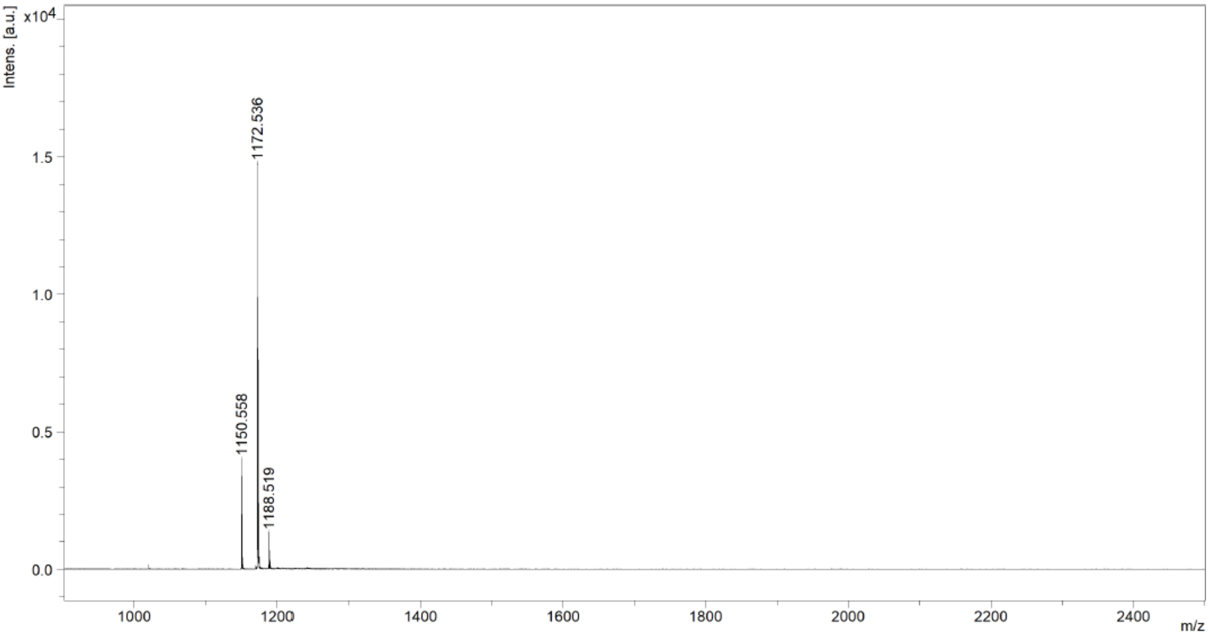

Figure S71. MALDI-TOF of **13**.

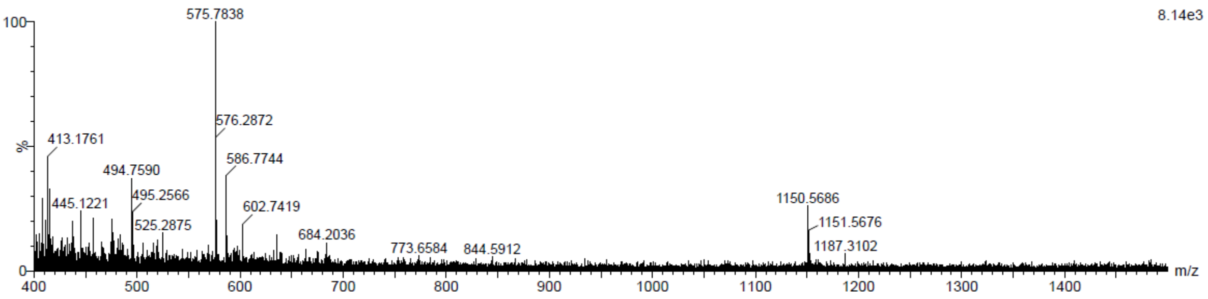

Figure S72. HR-MS of **13**.

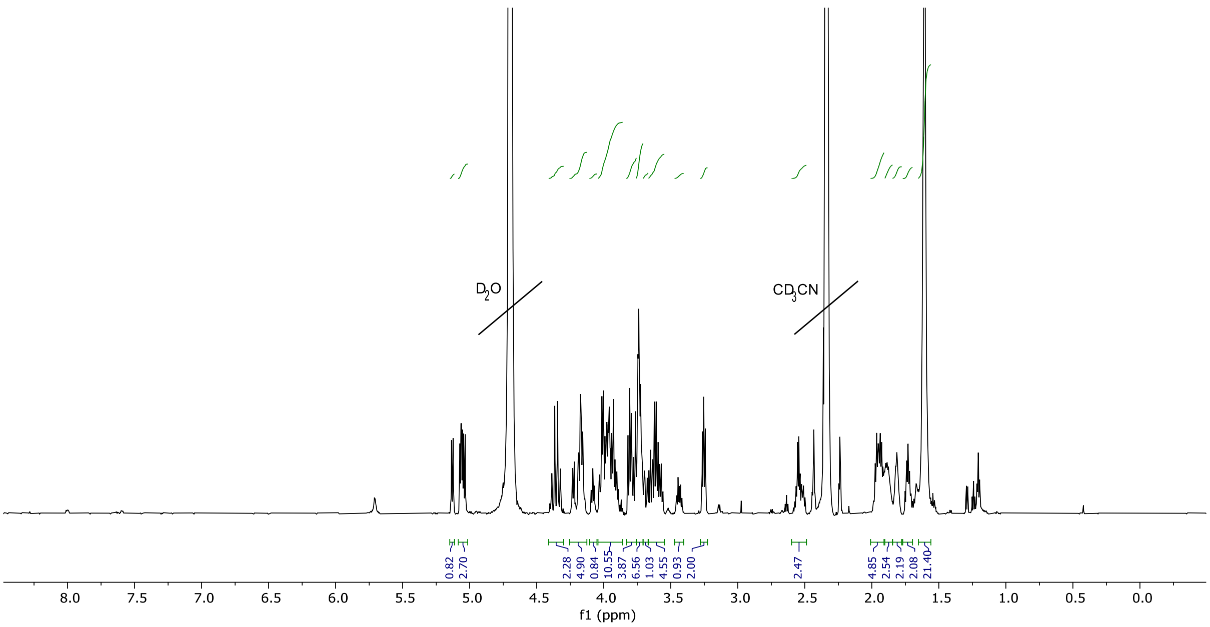

Figure S73. ^{1}H NMR (700MHz, D_2O/CD_3CN = 3/2) spectrum of **13**.

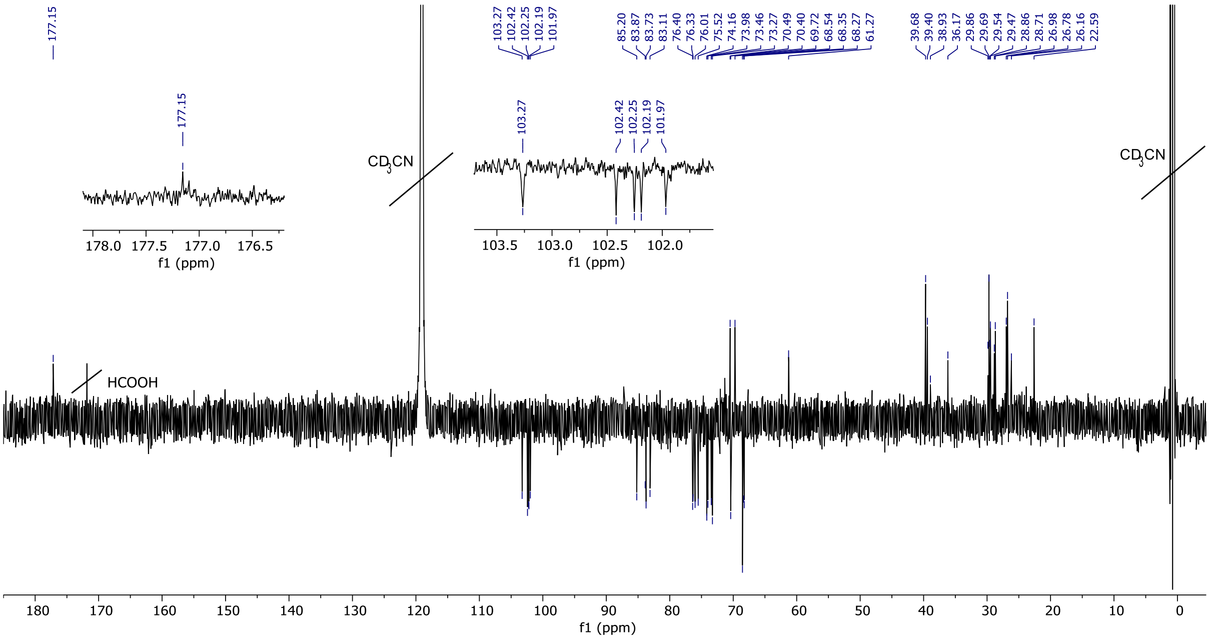

Figure S74. ^{13}C APT NMR (176MHz, D_2O/CD_3CN = 3/2) spectrum of **13**.

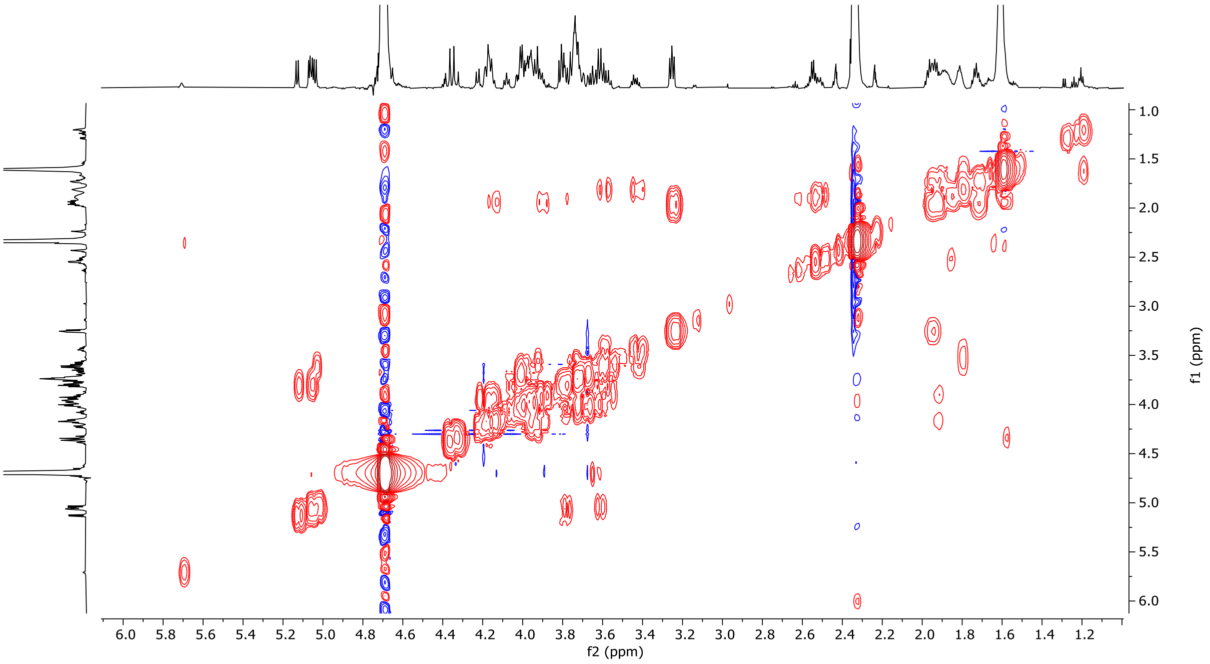

Figure S75. COSY NMR (700MHz, D_2O/CD_3CN = 3/2) spectrum of **13**.

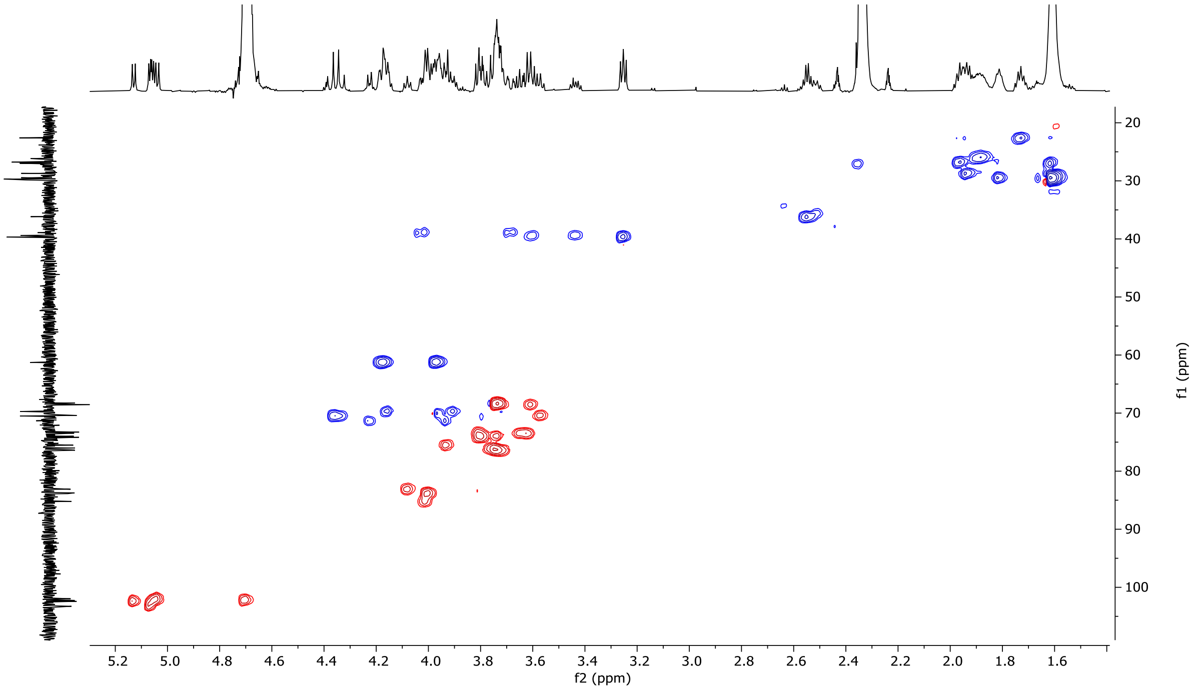

Figure S76. HSQC NMR (700MHz, D_2O/CD_3CN = 3/2) spectrum of **13**.

Synthesis and analytical data of 12

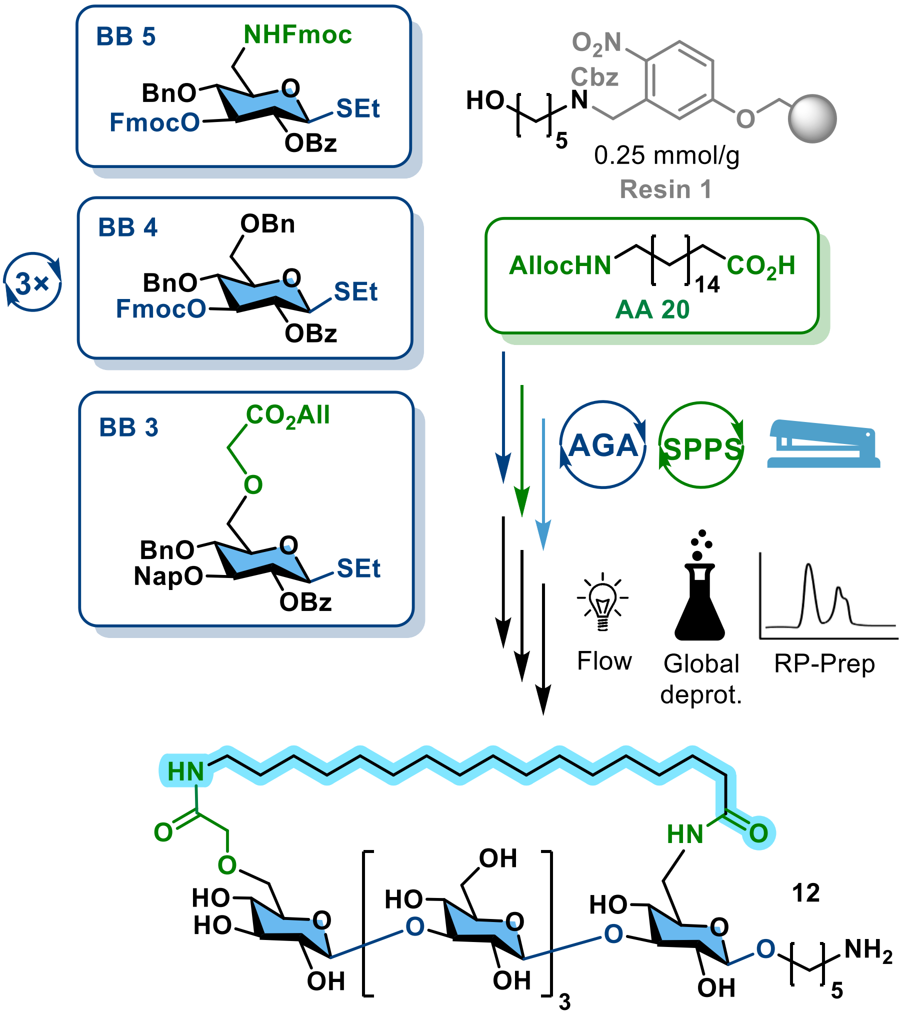

Step	Module	BB/reagent	Repeat	Notes
AGA	A	**1** (0.015 mmol)	1	0.25 mmol/g
	B, C1, D1	**5** (0.10 mmol)	1	-
	B, C1, D1	**4** (0.10 mmol)	3	-
	B, C1	**3** (0.10 mmol)	1	-
SPPS	E, F	**AA 20**	1	-
	I	-	-	-
Stapling	J	-	-	-
Post solid-phase	M	-	3	-
	K2	-	-	-
	N2	-	-	-
	O	-	-	Methods 3 and 6

After automated glycan assembly, side-chain attachment, on-resin stapling, photo-cleavage, global deprotection, purification, and lyophilization **12** was obtained as a white solid (1.5 mg, 8%).

R_t (Method 3) = 20.9 min.

HRMS (ESI/Q-TOF) *m/z*: $[M + 2H]^{2+}$ Calcd for $C_{54}H_{99}N_3O_{27}$ 610.8228; Found 610.8236.

^1H NMR (700 MHz, D_2O/CD_3CN = 3/2) δ 5.16 (d, *J* = 8.1 Hz, 1H), 5.10 (d, *J* = 7.9 Hz, 1H), 5.08 (d, *J* = 7.8 Hz, 1H), 5.05 (d, *J* = 7.9 Hz, 1H), 4.46 – 4.36 (m, 2H), 4.28 – 4.21 (m, 6H), 4.13 (t, *J* = 8.9 Hz, 1H), 4.10 – 3.95 (m, 9H), 3.92 – 3.76 (m, 11H), 3.74 (t, *J* = 8.7 Hz, 1H), 3.72 – 3.65 (m, 4H), 3.51 (dq, *J* = 13.7, 6.6 Hz, 1H), 3.35 – 3.30 (m, 2H), 2.61 (ddt, *J* = 20.0, 15.4, 7.4 Hz, 4H), 2.51 (p, *J* = 2.5 Hz, 1H), 2.31 (p, *J* = 2.5 Hz, 1H), 2.08 – 1.99 (m, 4H), 1.98 – 1.92 (m, 1H), 1.92 – 1.85 (m, 2H), 1.81 (dt, *J* = 14.2, 7.1 Hz, 2H), 1.68 (d, *J* = 6.8 Hz, 38H).

Poor solubility of the compound did not allow for sufficient sensitivity during 24-hour ^{13}C NMR acquisition (176 MHz).

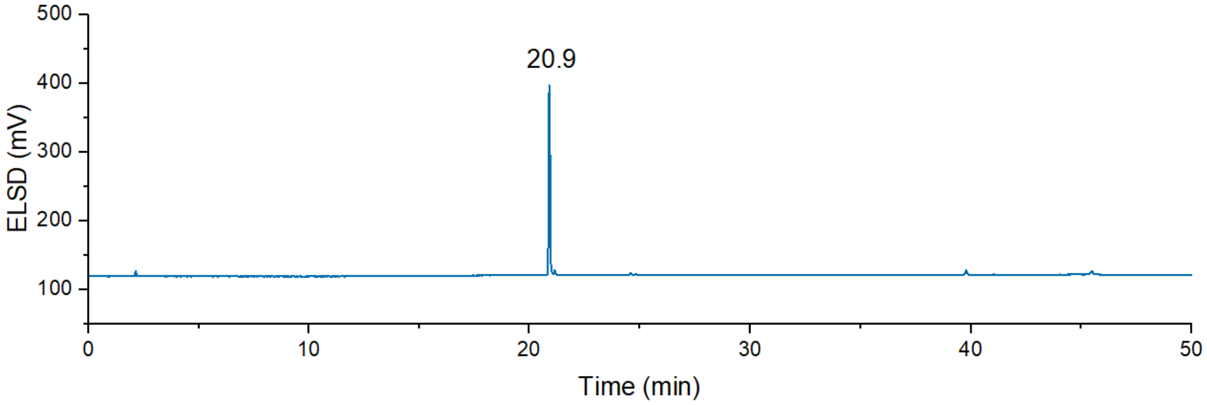

Figure S77. RP-HPLC trace of pure **12**.

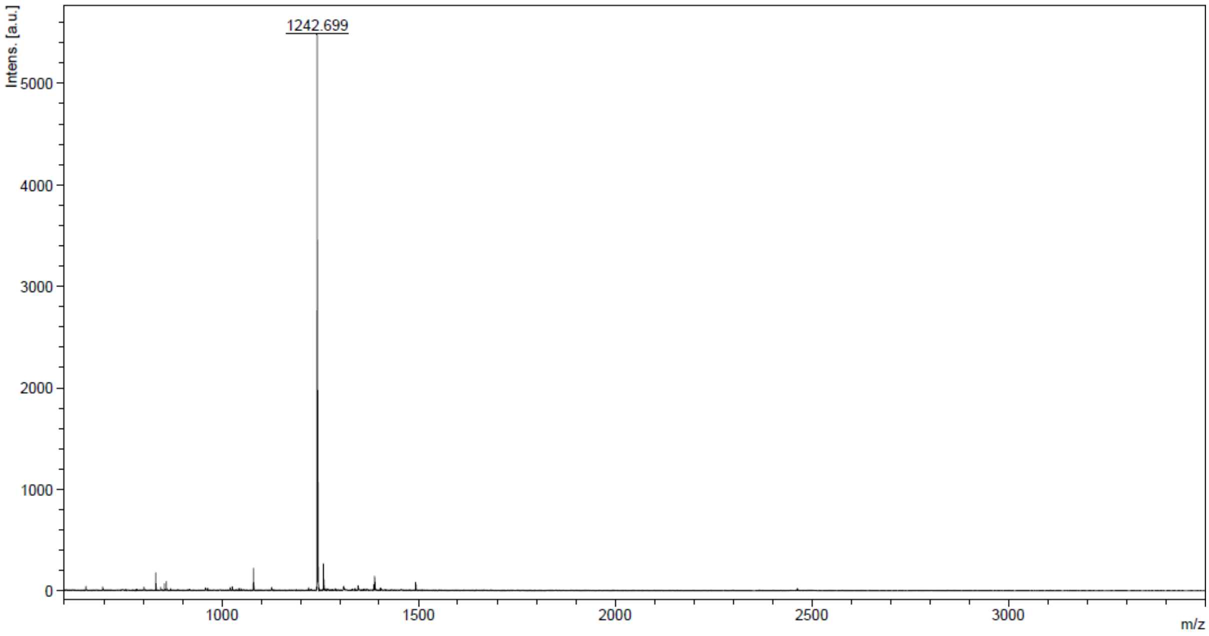

Figure S78. MALDI-TOF of **12**.

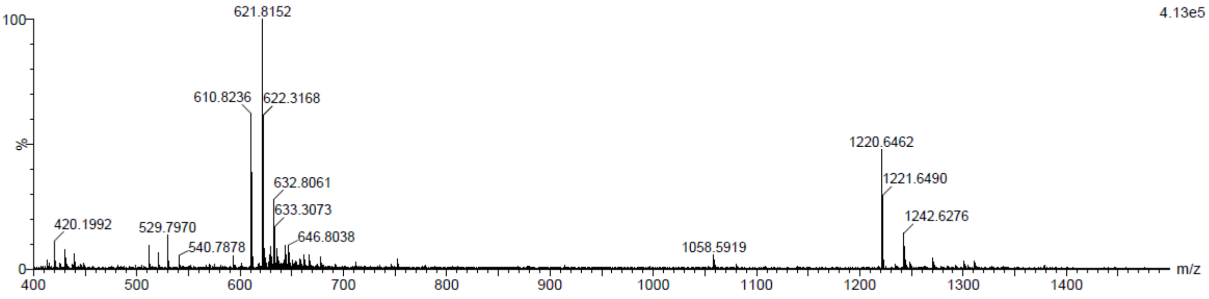

Figure S79. HR-MS of **12**.

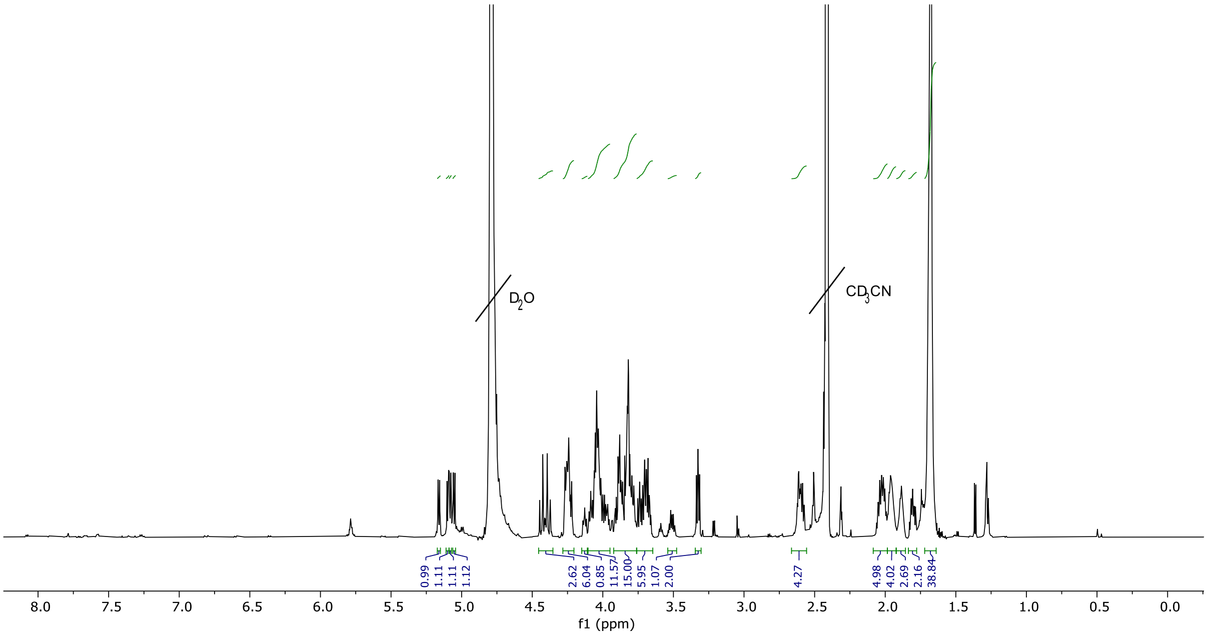

Figure S80. ^{1}H NMR (700MHz, D_2O/CD_3CN = 3/2) spectrum of **12**.

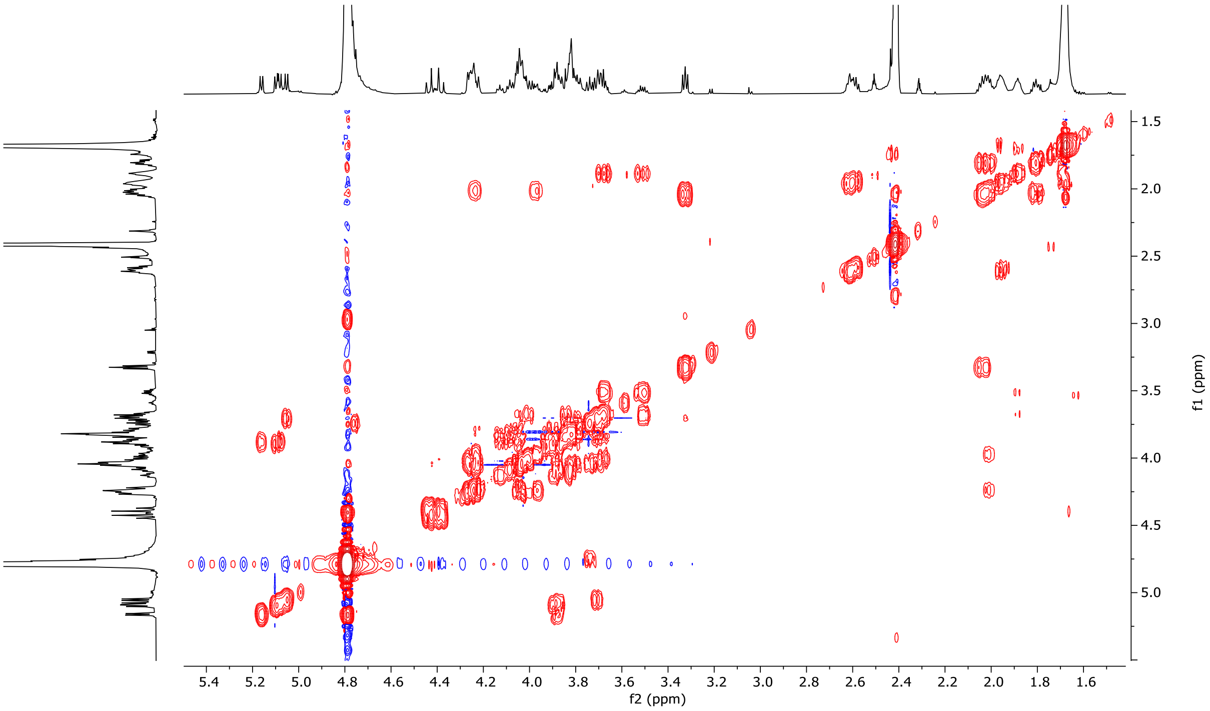

Figure S81. COSY NMR (700MHz, D_2O/CD_3CN = 3/2) spectrum of **12**.

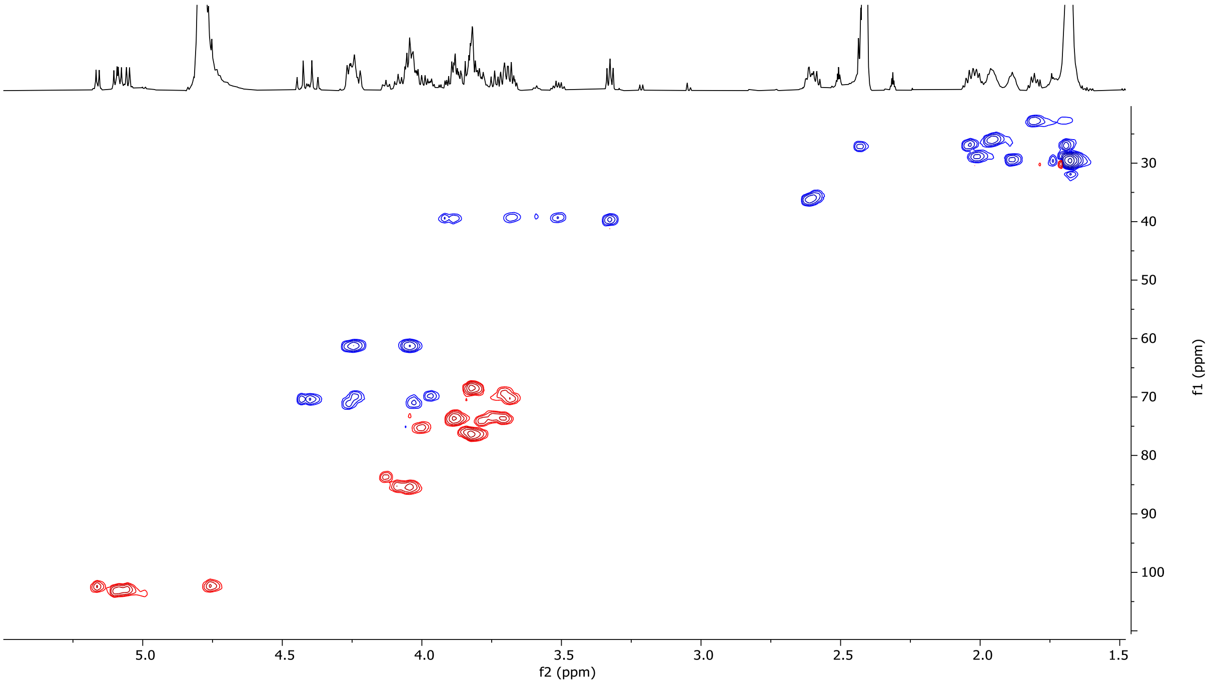

Figure S82. HSQC NMR (700MHz, D_2O/CD_3CN = 3/2) spectrum of **12**.

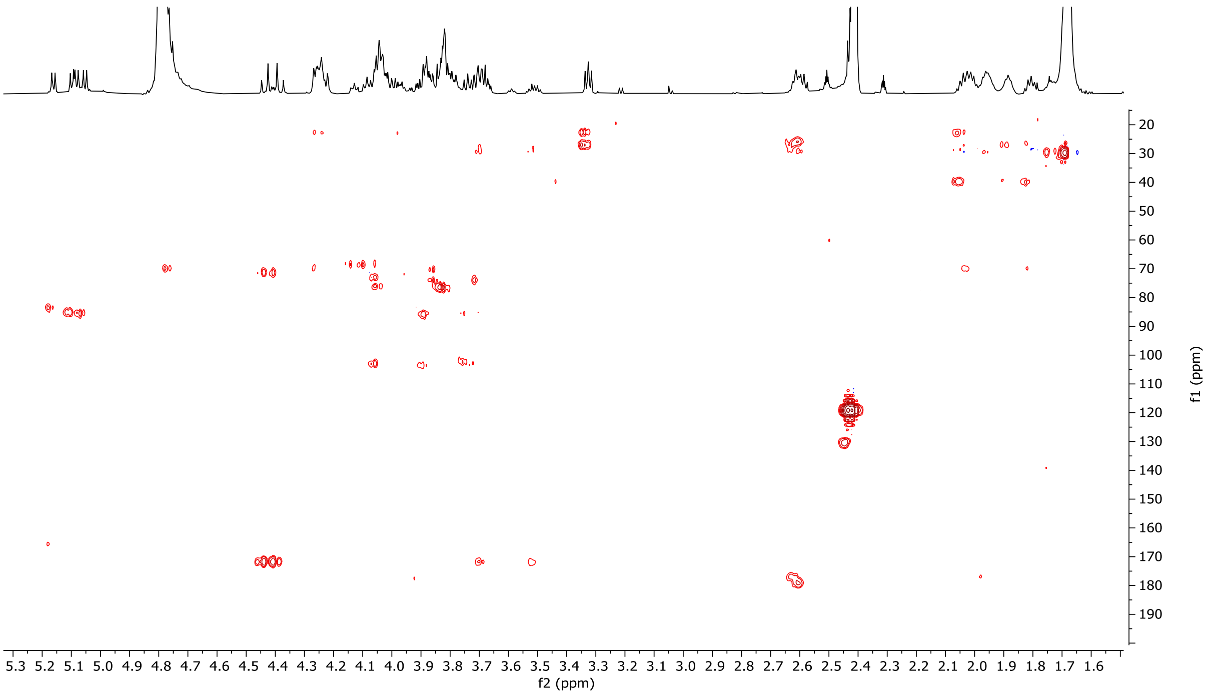

Figure S83. HMBC NMR (700MHz, D_2O/CD_3CN = 3/2) spectrum of **12**.

Synthesis and analytical data of 8

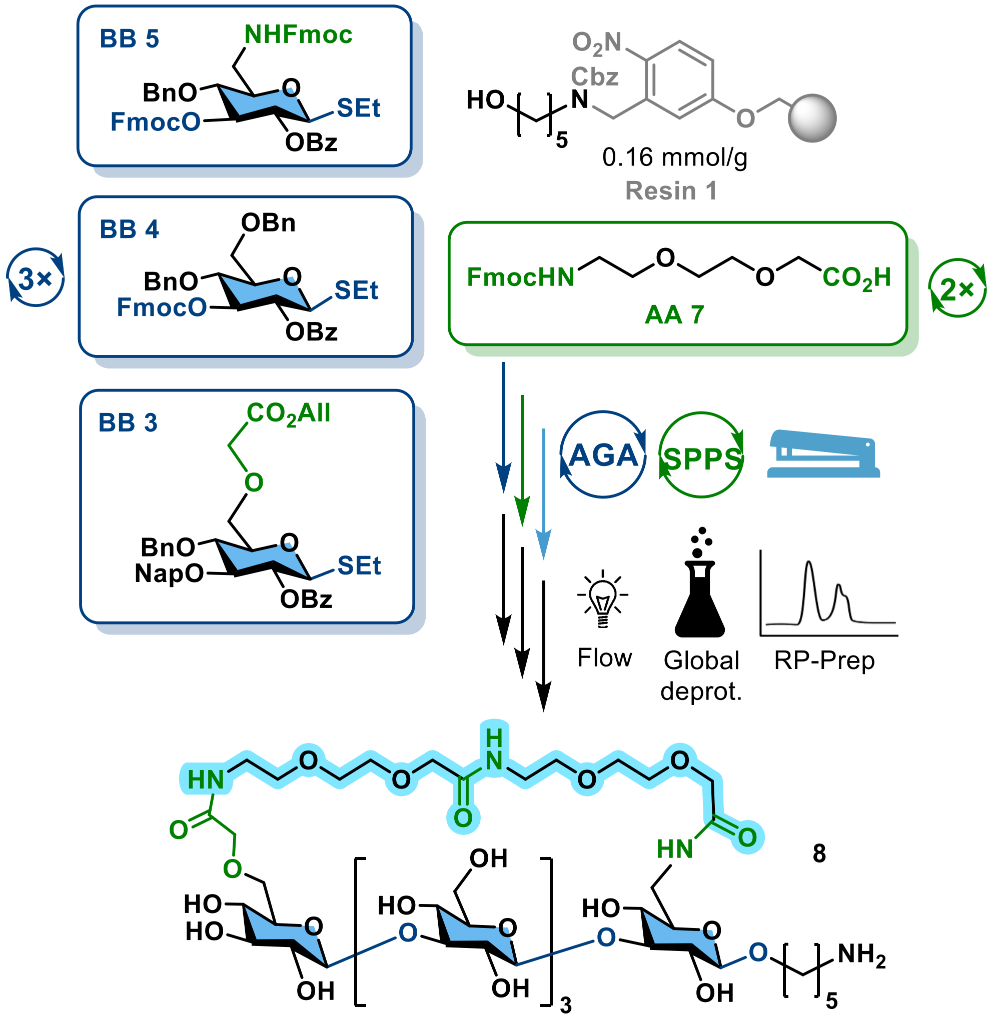

Step	Module	BB/reagent	Repeat	Notes
AGA	A	**1** (0.015 mmol)	1	0.16 mmol/g
	B, C2, D1	**5** (0.10 mmol)	1	-
	B, C2, D1	**4** (0.10 mmol)	3	-
	B, C2	**3** (0.10 mmol)	1	-
SPPS	E, F	**AA 7**	2	-
	I, E	-	-	-
Stapling	J	-	-	-
Post solid-phase	M	-	3	-
	K2	-	-	-
	N2	-	-	-
	O	-	-	Methods 1 and 4

After automated glycan assembly, side-chain construction, on-resin stapling, photo-cleavage, global deprotection, purification, and lyophilization **8** was obtained as a white solid (1.1 mg, 6%).

R_t (Method 1) = 30.2 min.

HRMS (ESI/Q-TOF) *m/z*: $[M + 2H]^{2+}$ Calcd for $C_{44}H_{88}N_4O_{32}$ 622.2686; Found 622.2688.

^{1}H NMR (700 MHz, D_2O) δ 4.46 (d, *J* = 8.1 Hz, 1H), 4.18 – 4.06 (m, 6H), 3.96 (d, *J* = 11.2 Hz, 1H), 3.89 (t, *J* = 13.9 Hz, 4H), 3.84 – 3.68 (m, 16H), 3.66 (q, *J* = 5.1 Hz, 6H), 3.61 – 3.39 (m, 19H), 3.33 (q, *J* = 9.4 Hz, 2H), 2.98 (t, *J* = 7.6 Hz, 2H), 1.65 (dp, *J* = 21.6, 7.4 Hz, 4H), 1.42 (p, *J* = 7.8 Hz, 2H).

^{13}C NMR (176 MHz, D_2O) δ 173.04, 172.74, 172.59, 102.80, 102.44, 101.86, 101.61, 101.56, 84.06, 82.67, 82.27, 82.01, 75.82, 75.61, 74.92, 73.80, 73.28, 73.13, 70.41, 70.27, 69.96, 69.46, 69.38, 69.26, 68.82, 68.15, 67.99, 67.82, 60.73, 39.29, 38.38, 28.15, 26.36, 22.06.

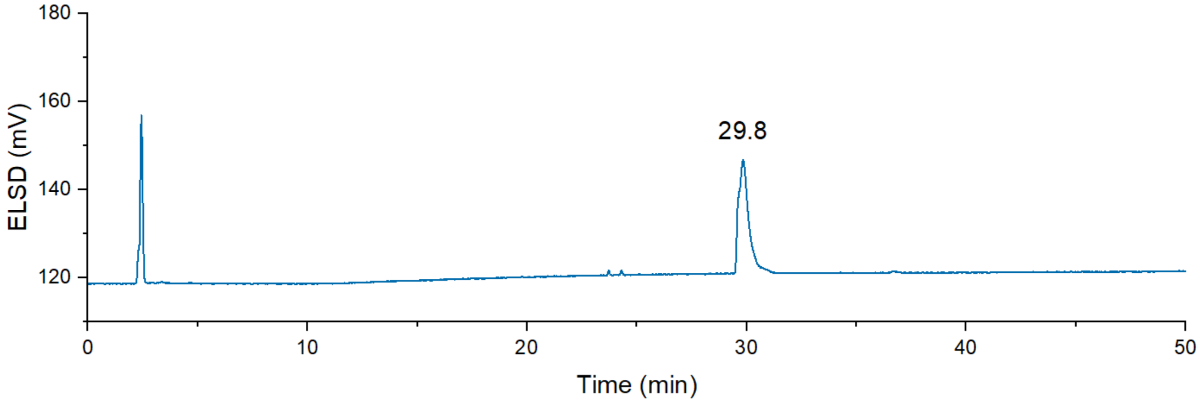

Figure S84. RP-HPLC trace of crude **8**.

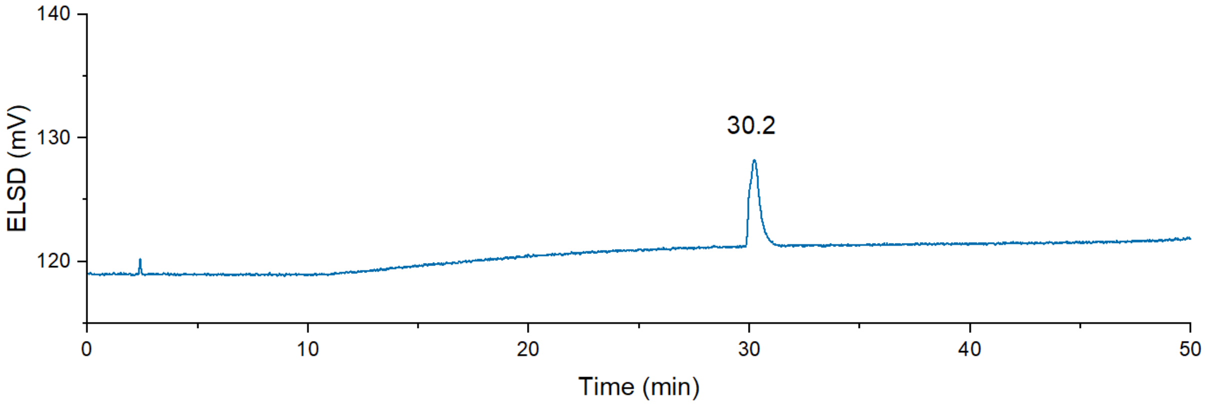

Figure S85. RP-HPLC trace of pure **8**.

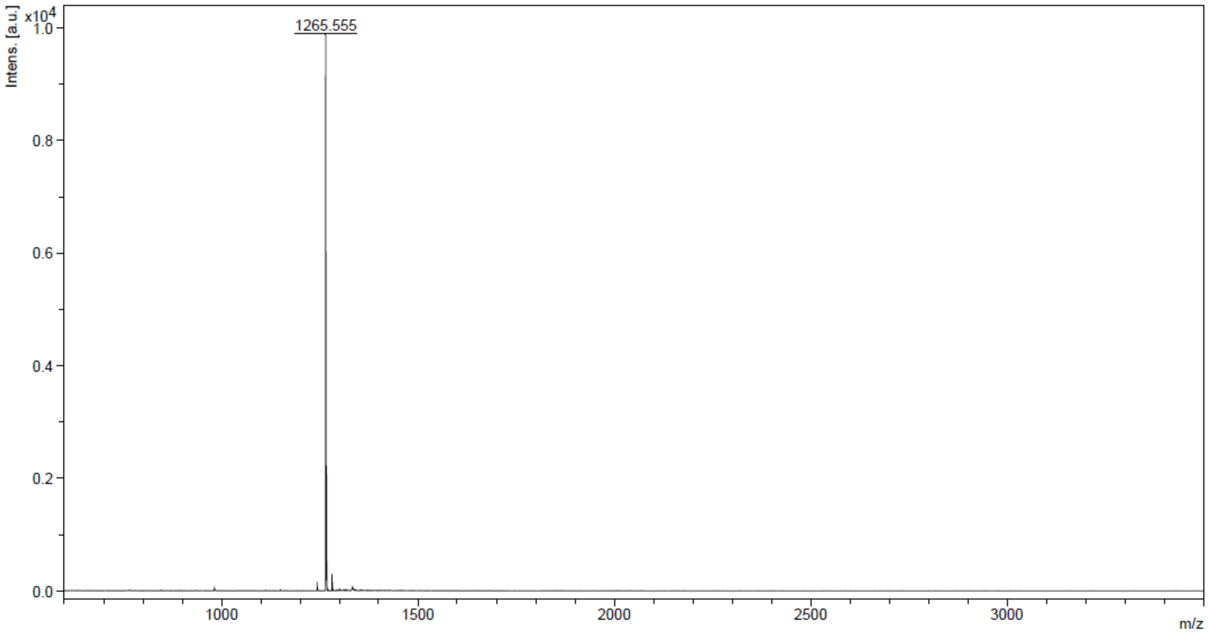

Figure S86. MALDI-TOF of **8**.

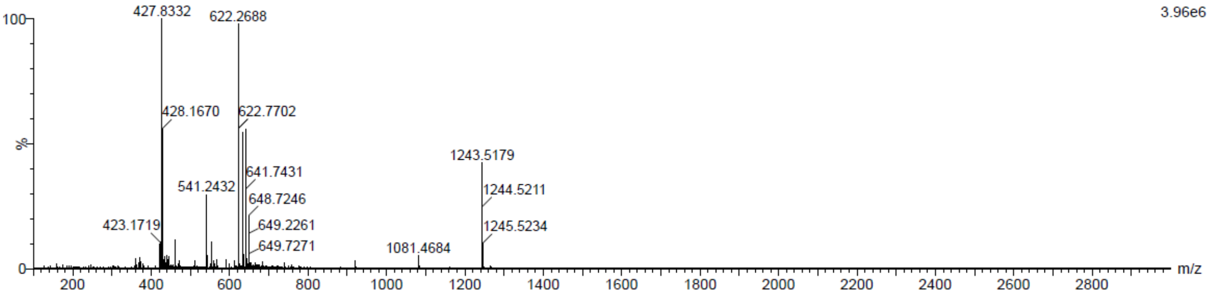

Figure S87. HR-MS of **8**.

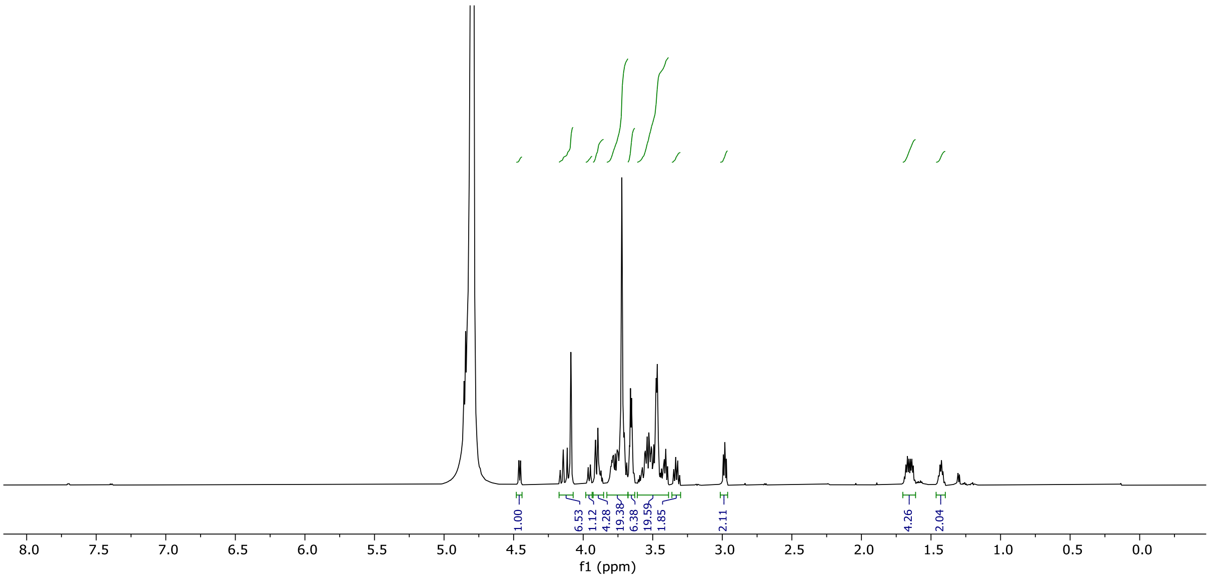

Figure S88. ^{1}H NMR (700MHz, D_2O) spectrum of **8**.

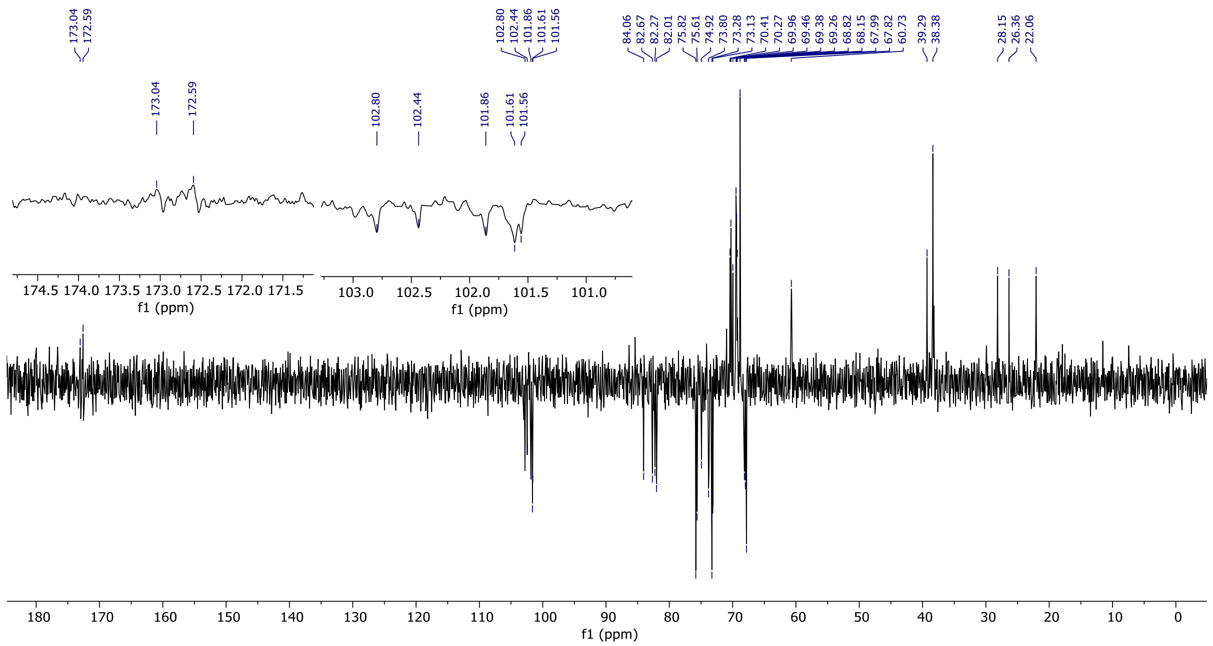

Figure S89. ^{13}C NMR APT (176MHz, D_2O) spectrum of **8**.

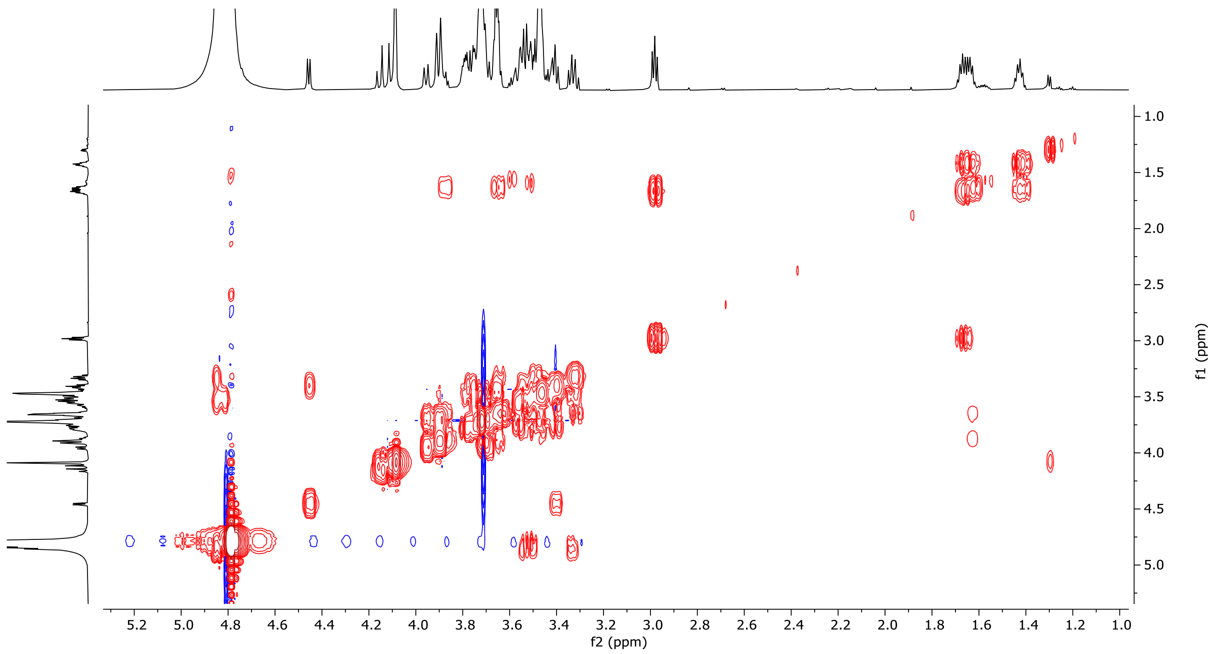

Figure S90. COSY NMR (700MHz, D_2O) spectrum of **8**.

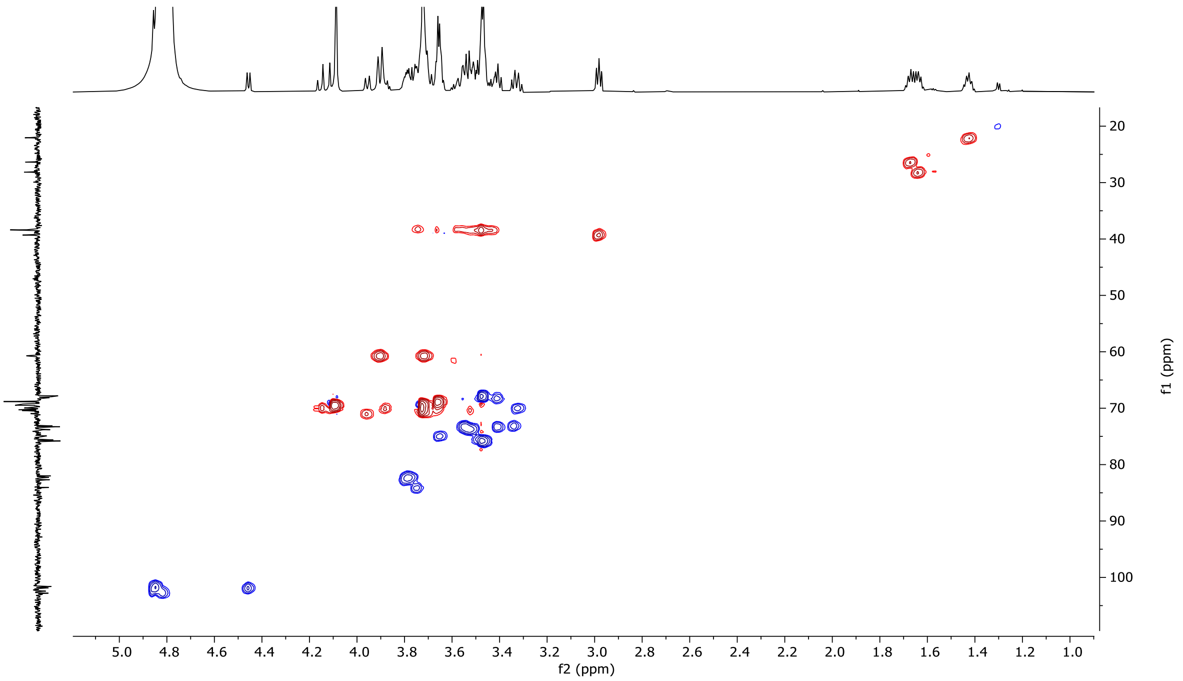

Figure S91. HSQC NMR (700MHz, D_2O) spectrum of **8**.

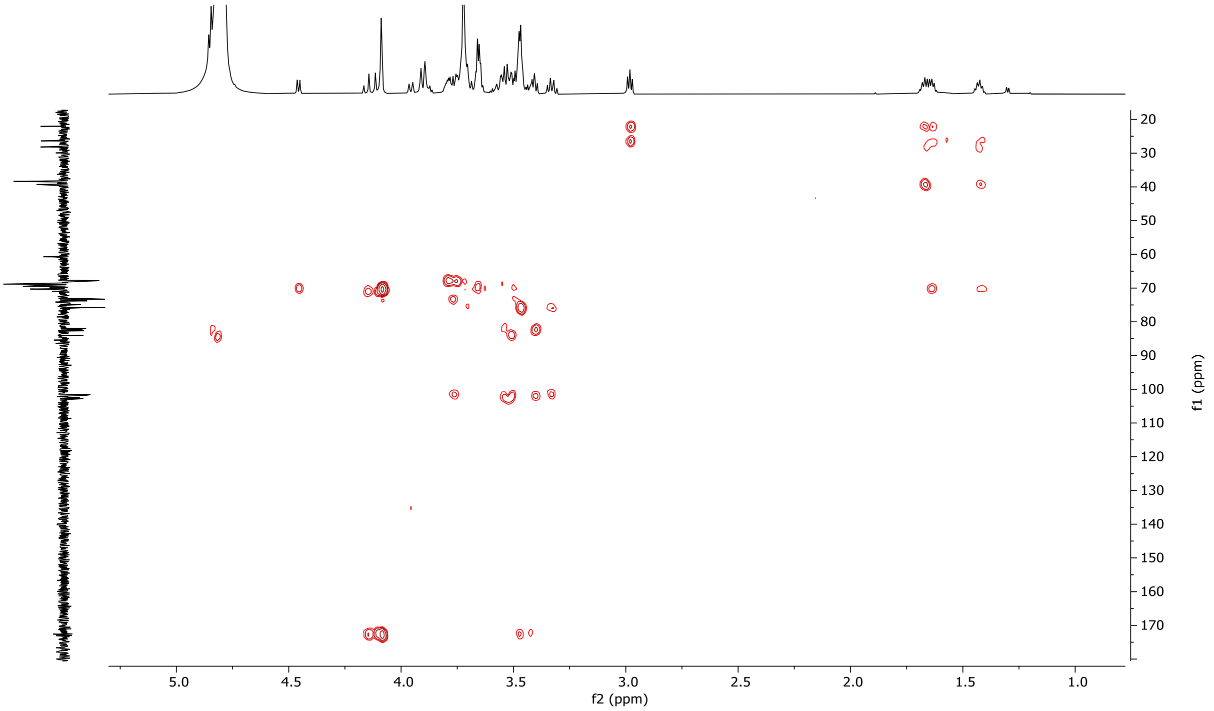

Figure S92. HMBC NMR (700MHz, D_2O) spectrum of **8**.

Synthesis and analytical data of 10

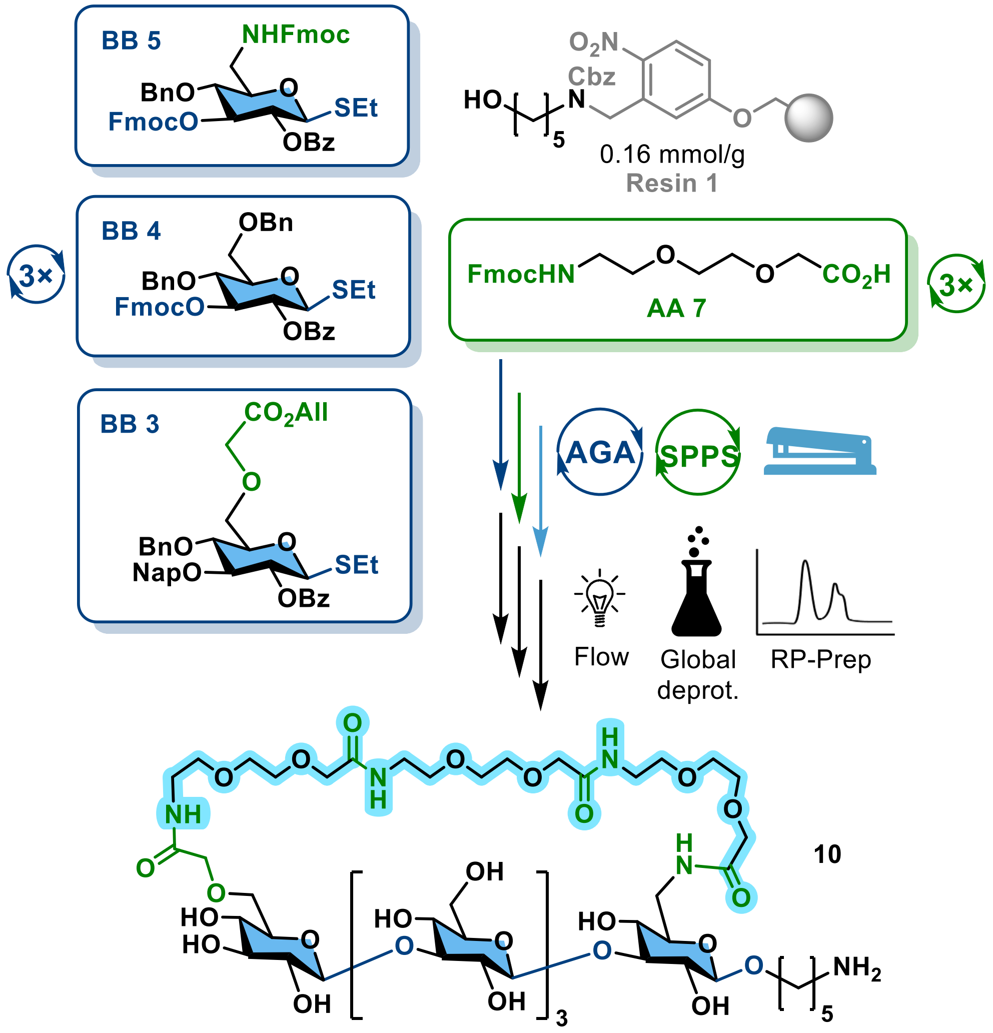

Step	Module	BB/reagent	Repeat	Notes
AGA	A	**1** (0.015 mmol)	1	0.16 mmol/g
	B, C2, D1	**5** (0.10 mmol)	1	-
	B, C2, D1	**4** (0.10 mmol)	3	-
	B, C2	**3** (0.10 mmol)	1	-
SPPS	E, F	**AA 7**	3	-
	I, E	-	-	-
Stapling	J	-	-	-
Post solid-phase	M	-	3	-
	K2	-	-	-
	N2	-	-	-
	O	-	-	Methods 2 and 5

After automated glycan assembly, side-chain construction, on-resin stapling, photo-cleavage, global deprotection, purification, and lyophilization **10** was obtained as a white solid (1.2 mg, 6%).

R_t (Method 2) = 25.4 min.

HRMS (ESI/Q-TOF) *m/z*: $[M + 2H]^{2+}$ Calcd for $C_{55}H_{99}N_5O_{35}$ 694.8055; Found 694.8066.

^{1}H NMR (700 MHz, D_2O) δ 4.45 (d, *J* = 8.1 Hz, 1H), 4.17 – 4.05 (m, 8H), 3.95 – 3.84 (m, 5H), 3.82 – 3.68 (m, 22H), 3.68 – 3.57 (zm, 10H), 3.52 (tdd, *J* = 15.6, 7.2, 3.1 Hz, 4H), 3.47 (q, *J* = 3.9 Hz, 10H), 3.45 – 3.38 (m, 2H), 3.38 – 3.32 (m, 2H), 2.98 (t, *J* = 7.6 Hz, 2H), 1.65 (dp, *J* = 21.6, 7.2 Hz, 5H), 1.42 (p, *J* = 7.7 Hz, 2H).

^{13}C NMR (176 MHz, D_2O) δ 172.93, 172.92, 172.58, 172.48, 102.69, 102.36, 101.99, 101.97, 101.86, 83.91, 83.03, 82.94, 82.89, 75.74, 75.62, 75.48, 74.77, 73.23, 73.16, 72.27, 70.76, 70.47, 70.31, 70.30, 69.92, 69.83, 69.48, 69.35, 69.27, 68.81, 68.74, 68.64, 68.02, 67.89, 67.84, 60.70, 60.65, 39.29, 38.41, 28.16, 26.37, 22.08.

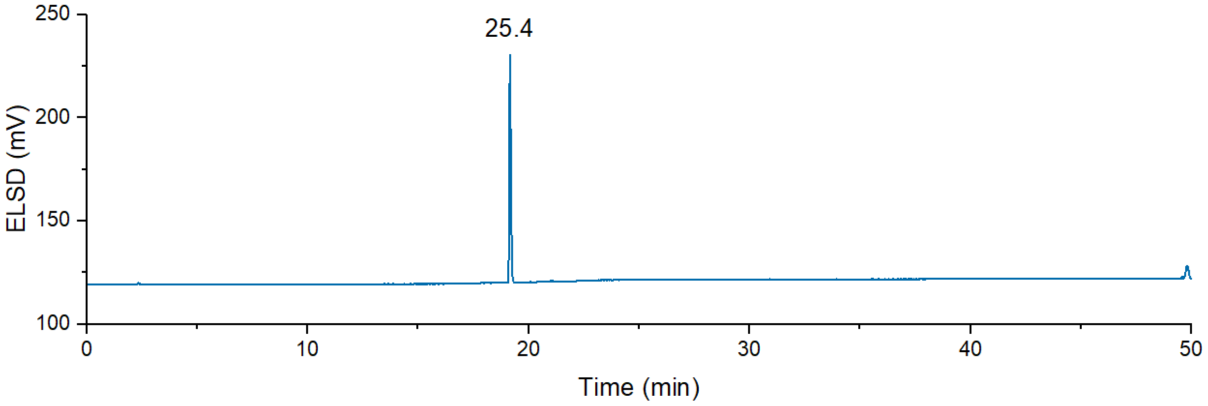

Figure S93. RP-HPLC trace of pure **10**.

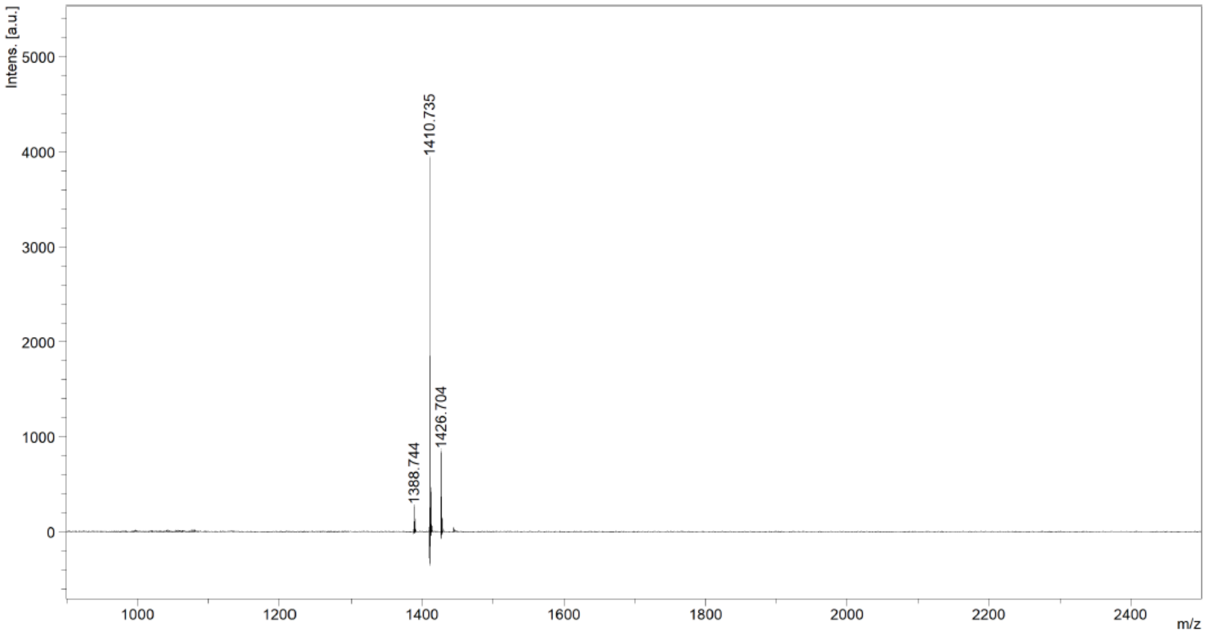

Figure S94. MALDI-TOF of **10**.

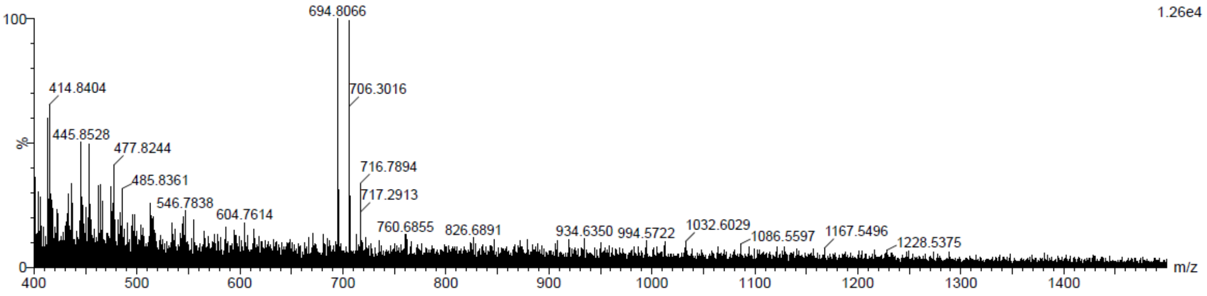

Figure S95. HR-MS of **10**.

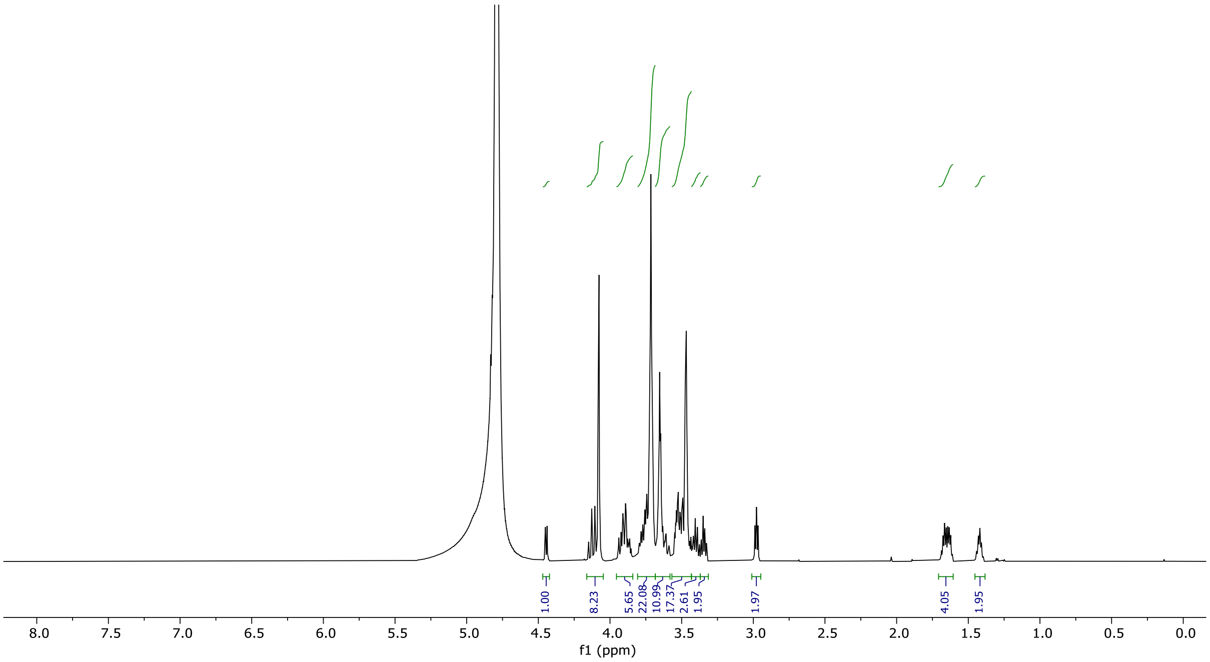

Figure S96. ^{1}H NMR (700MHz, D_2O) spectrum of **10**.

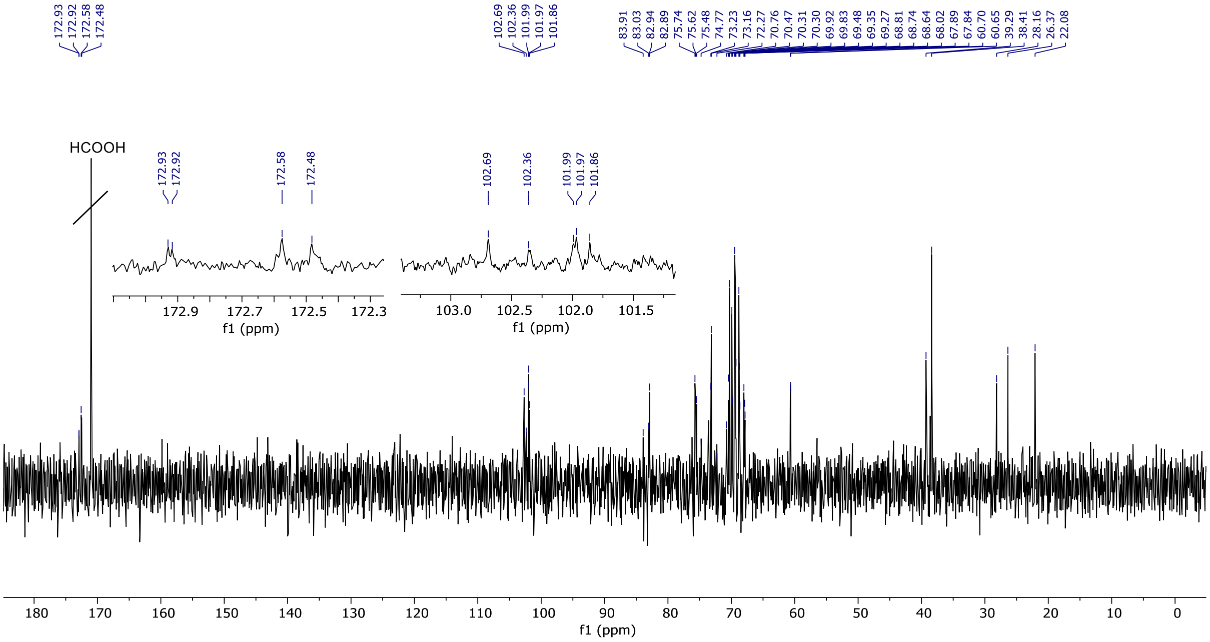

Figure S97. ^{13}C NMR (176MHz, D_2O) spectrum of **10**.

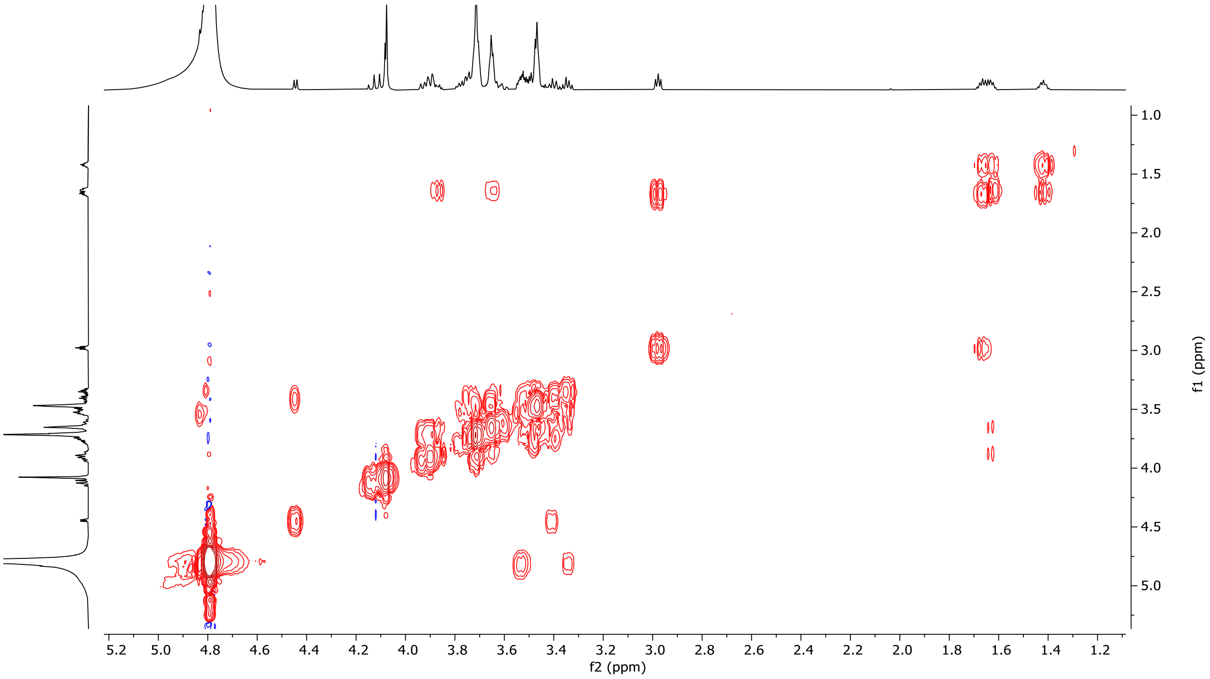

Figure S98. COSY NMR (700MHz, D_2O) spectrum of **10**.

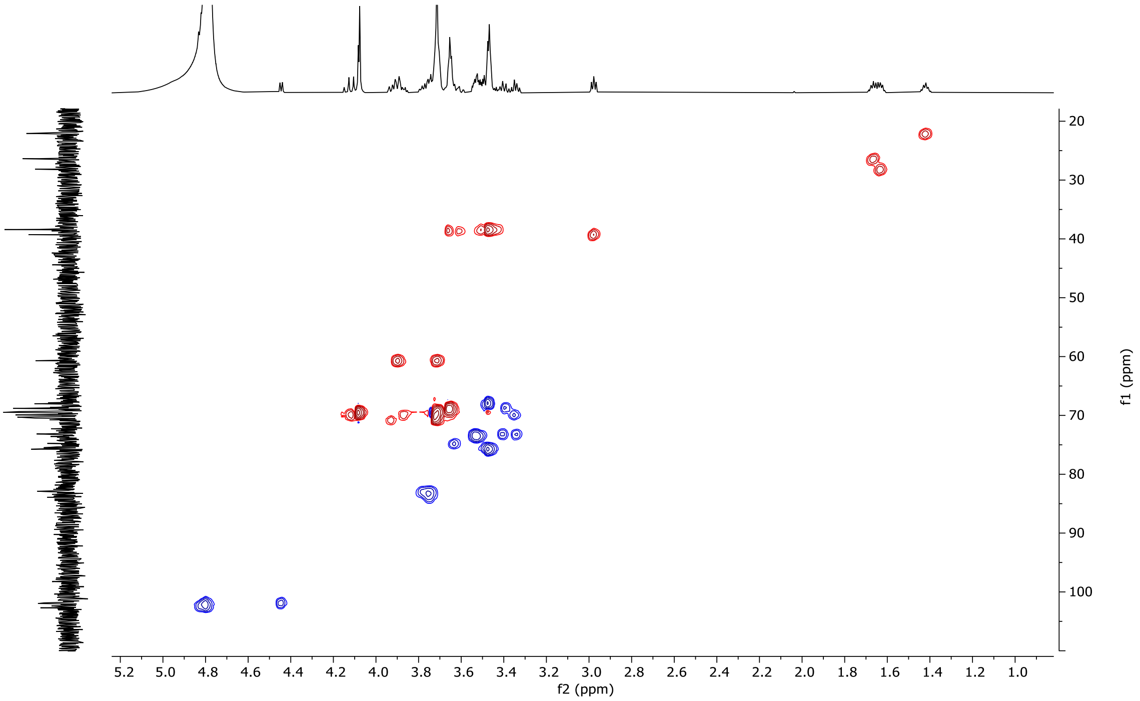

Figure S99. HSQC NMR (700MHz, D_2O) spectrum of **10**.

Synthesis and analytical data of 9

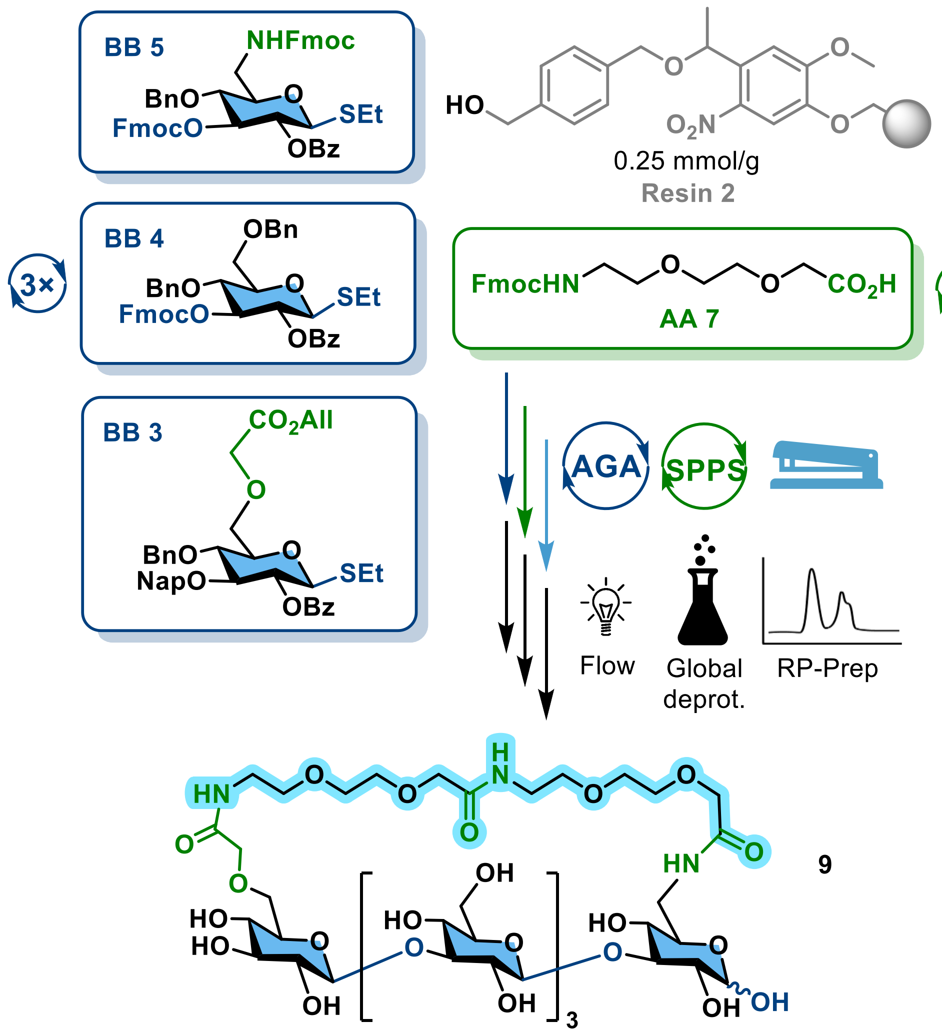

9

Step	Module	BB/reagent	Repeat	Notes
AGA	A	**2** (0.015 mmol)	1	0.25 mmol/g
	B, C2, D1	**5** (0.10 mmol)	1	-
	B, C2, D1	**4** (0.10 mmol)	3	-
	B, C2	**3** (0.10 mmol)	1	-
SPPS	E, F	**AA 7**	2	-
	I, E	-	-	-
Stapling	J	-	-	-
Post solid-phase	M	-	3	10% MeOH in DCM
	K2	-	-	-
	N2	-	2	-
	O	-	-	Methods 2 and 5

After automated glycan assembly, side-chain construction, on-resin stapling, photo-cleavage, global deprotection, purification, and lyophilization **9** was obtained as a white solid (2.5 mg, 15%).

R_t (Method 2) = 13.8 and 14.0 min (mixture of α- and β-anomers).

HRMS (ESI/Q-TOF) *m/z*: $[M + H]^+$ Calcd for $C_{44}H_{76}N_3O_{32}$ 1158.4406; Found 1158.4304.

^{1}H NMR (700 MHz, D_2O) δ 5.17 (d, *J* = 3.8 Hz, 1H, α-anomer), 4.65 (d, *J* = 8.1 Hz, 1H, β-anomer), 4.18 – 4.05 (m, 6H), 3.99 – 3.85 (m, 5H), 3.84 – 3.68 (m, 18H), 3.68 – 3.63 (m, 5H), 3.63 – 3.29 (m, 21H).

^{13}C NMR (176 MHz, D_2O) δ 173.02, 172.60, 172.53, 102.79, 102.43, 101.82, 101.58, 95.63 (β-anomer), 92.14 (α-anomer), 84.07, 82.68, 82.22, 82.02, 80.24, 75.80, 75.54, 74.89, 74.24, 73.82, 73.28, 73.11, 71.49, 70.93, 70.37, 70.26, 69.93, 69.45, 69.36, 69.22, 68.81, 68.17, 68.00, 67.81, 60.62, 38.39.

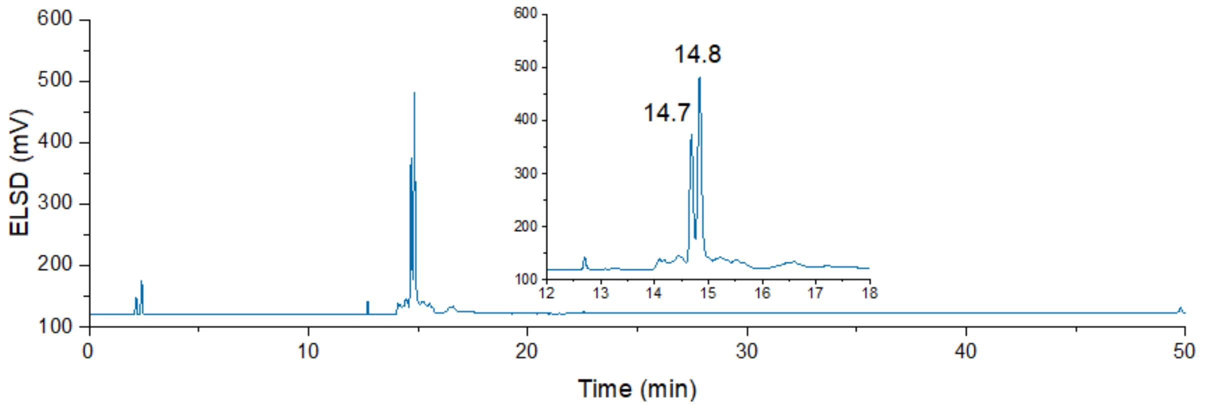

Figure S100. RP-HPLC trace of crude **9**.

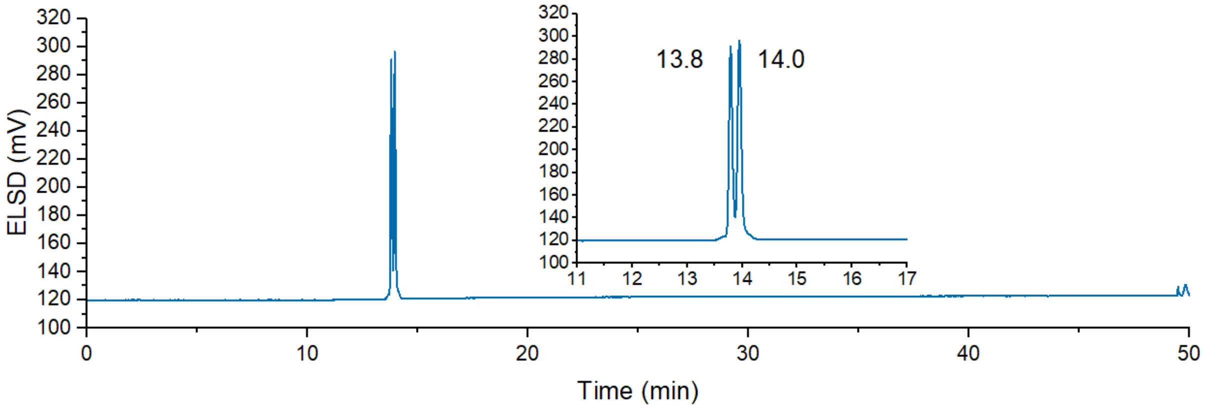

Figure S101. RP-HPLC trace of pure **9**.

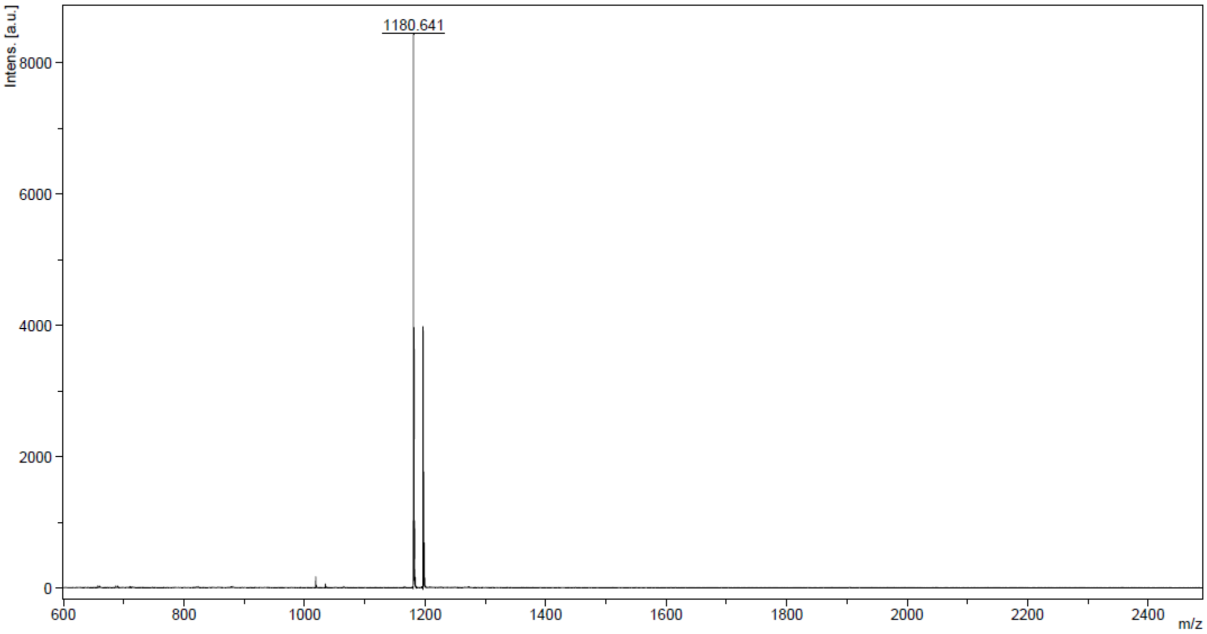

Figure S102. MALDI-TOF of **9**.

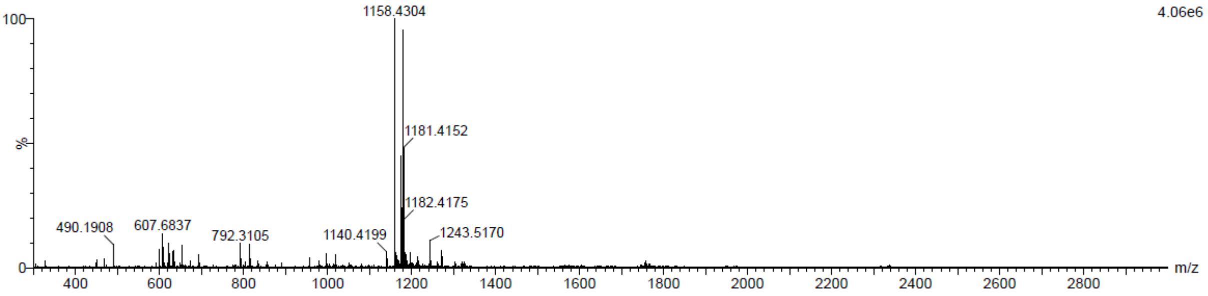

Figure S103. HR-MS of **9**.

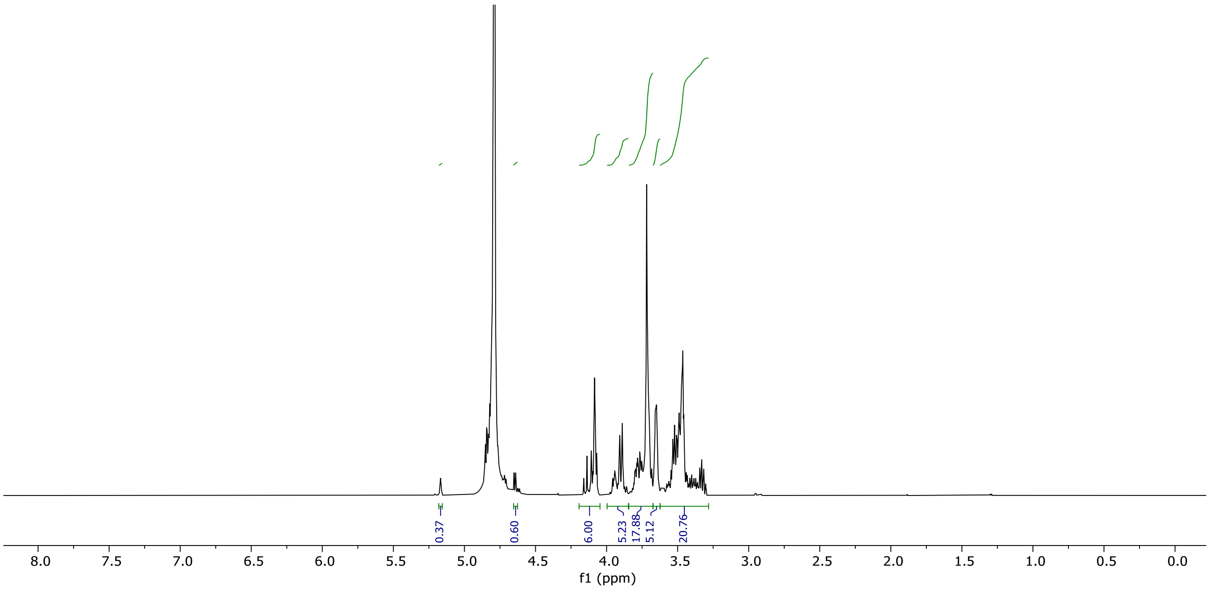

Figure S104. ^{1}H NMR (700MHz, D_2O) spectrum of **9**.

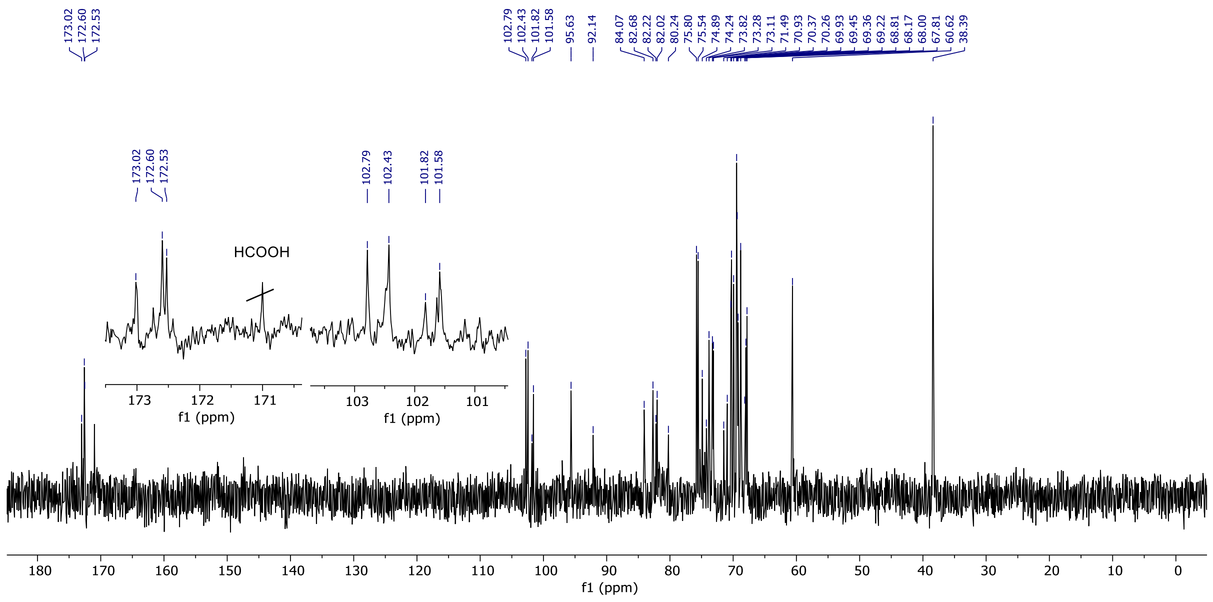

Figure S105. ^{13}C NMR (176MHz, D_2O) spectrum of **9**.

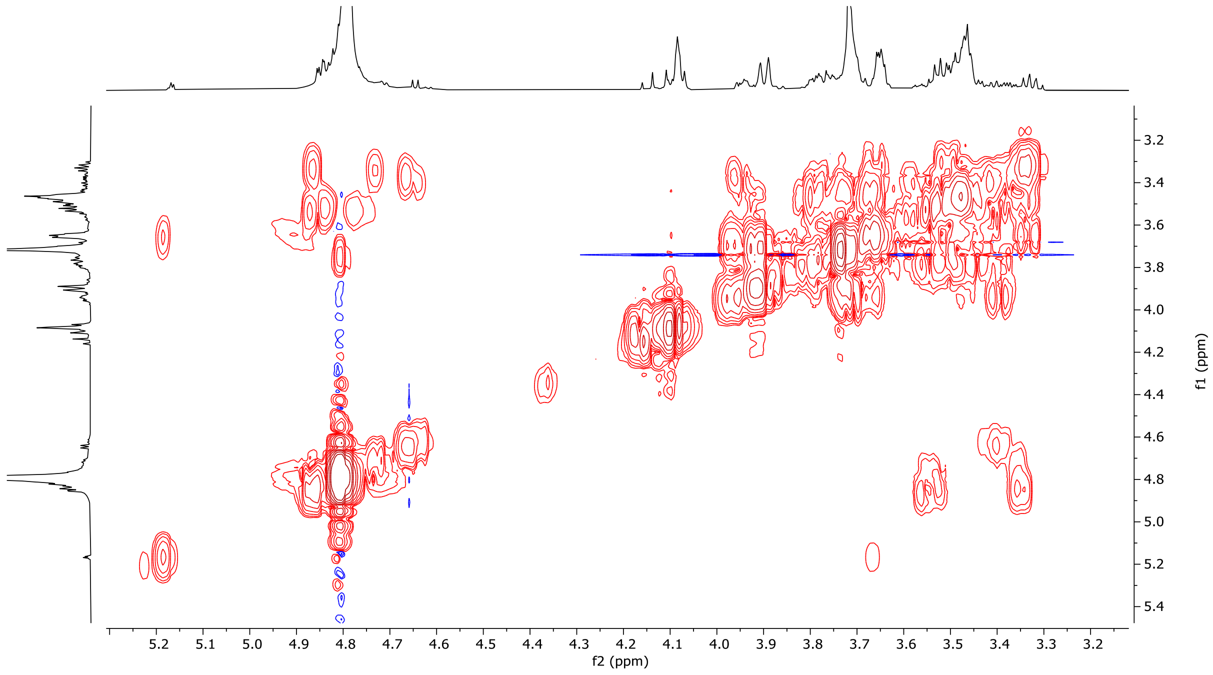

Figure S106. COSY NMR (700MHz, D_2O) spectrum of **9**.

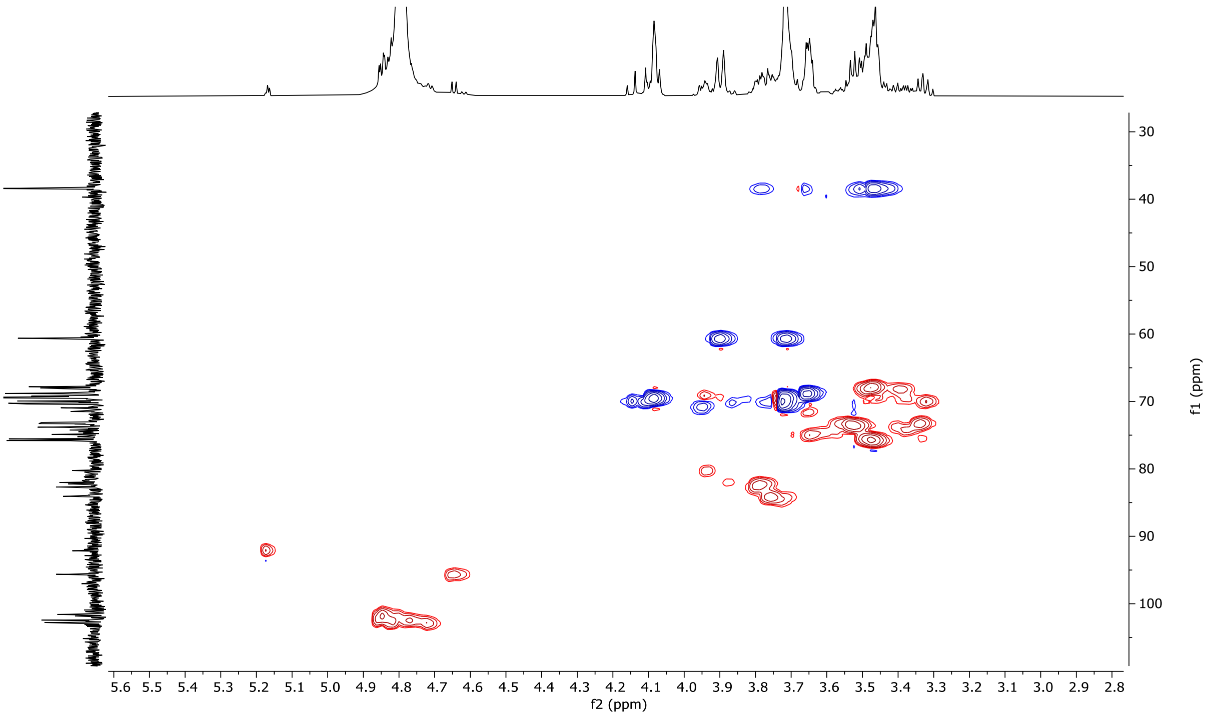

Figure S107. HSQC NMR (700MHz, D_2O) spectrum of **9.**

Synthesis and analytical data of 18

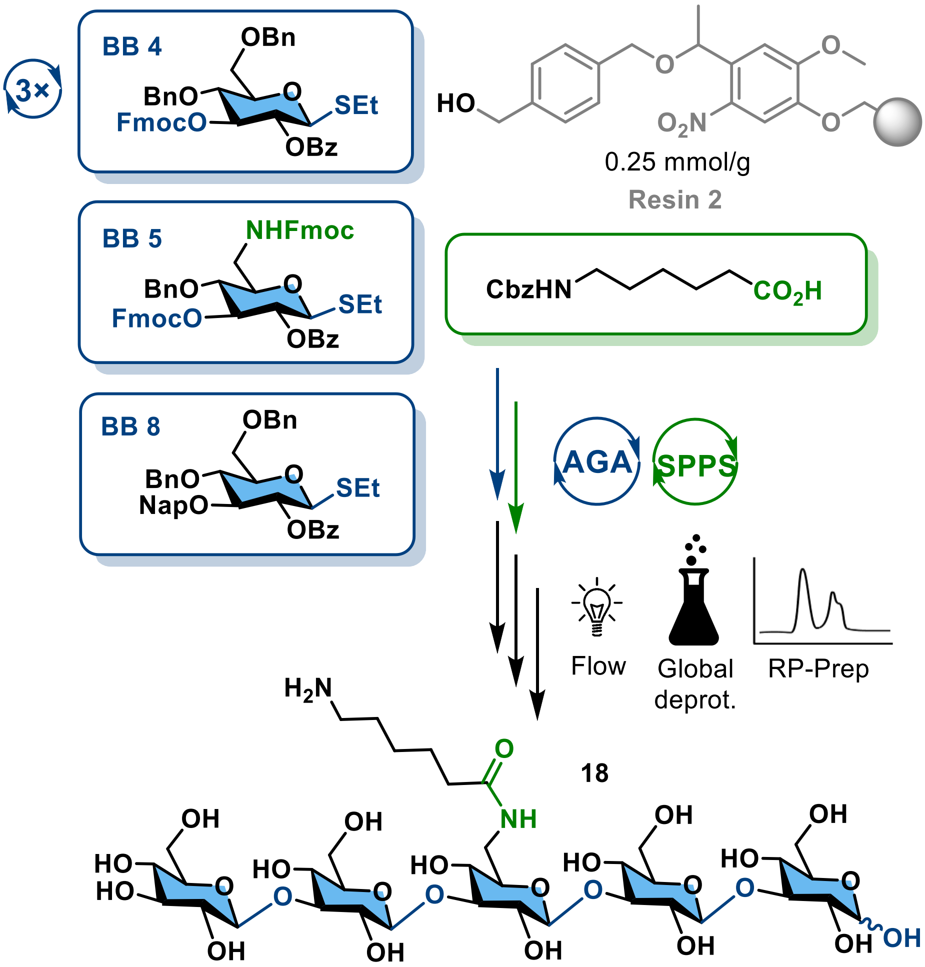

Step	Module	BB/reagent	Repeat	Notes
AGA	A	**2** (0.015 mmol)	1	0.25 mmol/g
	B, C2, D1	**4** (0.10 mmol)	2	-
	B, C2, D1	**5** (0.10 mmol)	1	-
	B, C2, D1	**4** (0.10 mmol)	1	-
	B, C2	**8** (0.10 mmol)	1	-
SPPS	E, F	CbzHN-C_5H_{10}-COOH	1	-
Post solid-phase	L	-	1	-
	M	-	3	-
	N2	-	2	-
	O	-	-	Methods 1 and 4

After automated glycan assembly, side-chain attachment, hydrazinolysis, photo-cleavage, global deprotection, purification, and lyophilization **18** was obtained as a white solid (8.3 mg, 59%).

R_t (Method 1) = 25.4 and 25.7 min (mixture of α- and β-anomers).

HRMS (ESI/Q-TOF) *m/z*: $[M + H]^+$ Calcd for $C_{36}H_{65}N_2O_{26}$ 941.3820; Found 941.3882.

^{1}H NMR (700 MHz, D_2O) δ 5.21 (d, *J* = 3.7 Hz, 1H, α-anomer), 4.77 (d, *J* = 8.1 Hz, 1H), 4.73 (ddd, *J* = 7.9, 4.2, 1.7 Hz, 2H), 4.71 (d, *J* = 8.0 Hz, 1H), 4.65 (d, *J* = 8.0 Hz, 1H, β-anomer), 3.93 – 3.79 (m, 5H), 3.79 – 3.63 (m, 9H), 3.56 – 3.43 (m, 11H), 3.43 – 3.36 (m, 3H), 3.33 (dd, *J* = 9.5, 8.0 Hz, 1H), 2.97 (t, *J* = 7.6 Hz, 2H), 2.28 (t, *J* = 7.4 Hz, 2H), 1.69 – 1.58 (m, 4H), 1.36 (p, *J* = 7.4 Hz, 2H).

^{13}C NMR (176 MHz, D_2O) δ 177.11, 102.75, 102.71, 102.56, 102.44, 102.36, 95.62 (β-anomer), 91.96 (α-anomer), 84.32, 84.15, 84.07, 83.55, 83.53, 82.17, 75.95, 75.60, 75.57, 75.52, 75.48, 73.85, 73.83, 73.82, 73.38, 73.29, 73.21, 73.14, 71.15, 71.02, 69.51, 69.32, 69.28, 68.06, 68.04, 67.99, 67.90, 60.61, 60.43, 39.74, 39.21, 35.34, 26.44, 26.42, 25.10, 25.08, 24.78, 24.75.

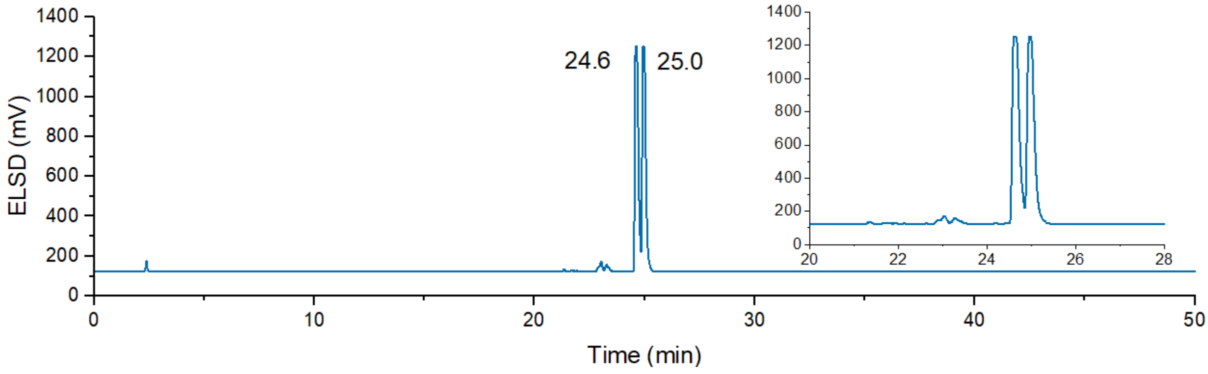

Figure S108. RP-HPLC trace of crude **18**.

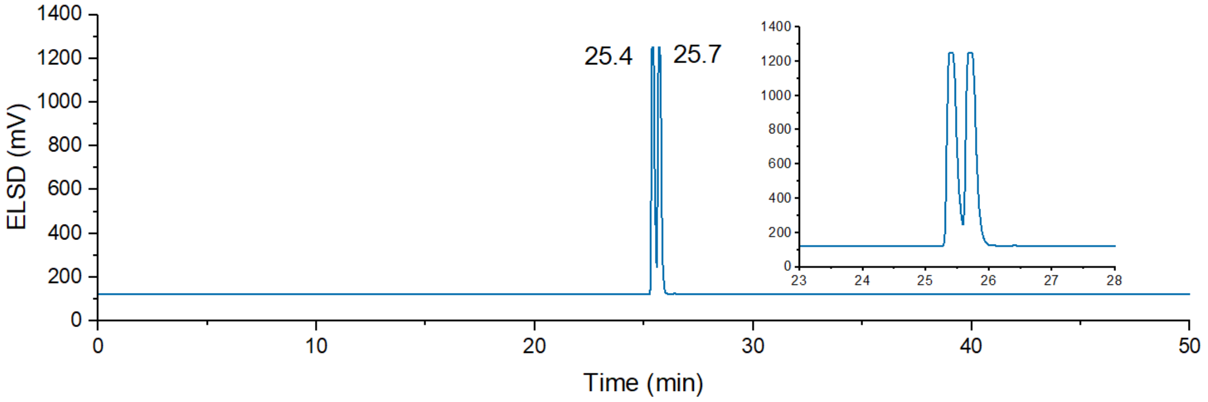

Figure S109. RP-HPLC trace of pure **18**.

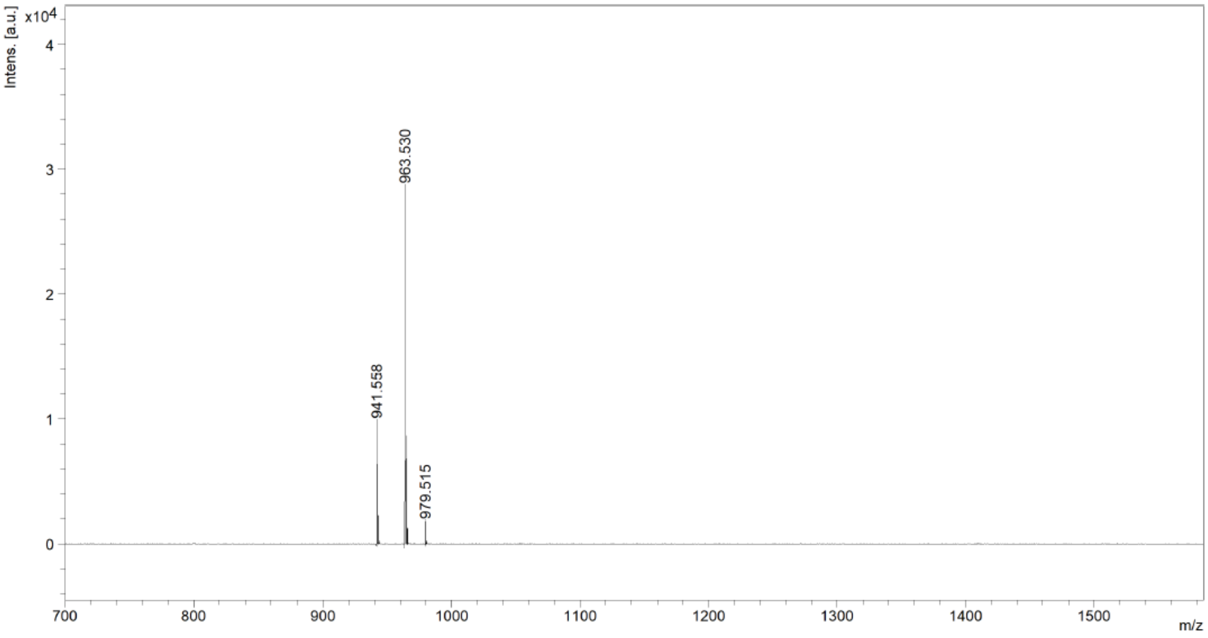

Figure S110. MALDI-TOF of **18**.

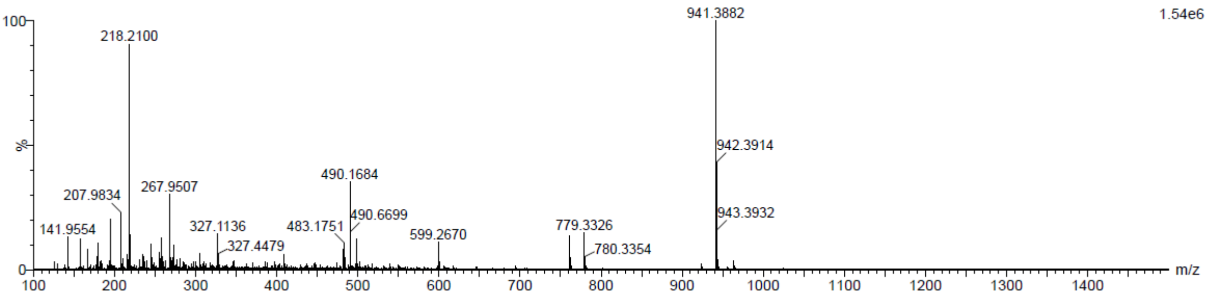

Figure S111. HR-MS of **18**.

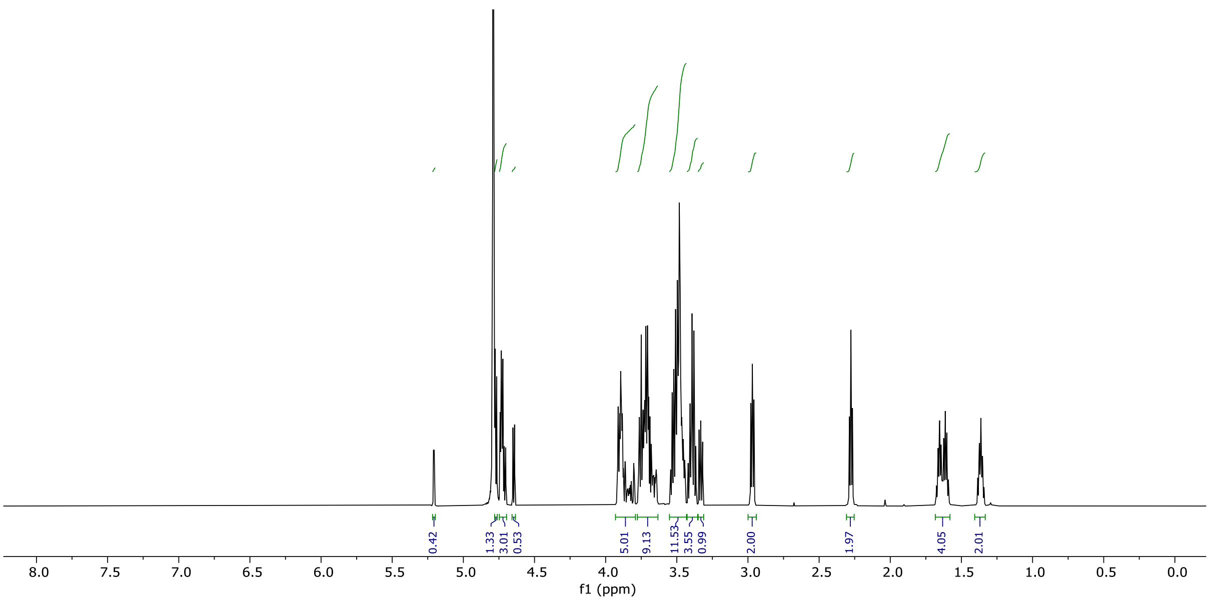

Figure S112. ^{1}H NMR (700MHz, D_2O) spectrum of **18**.

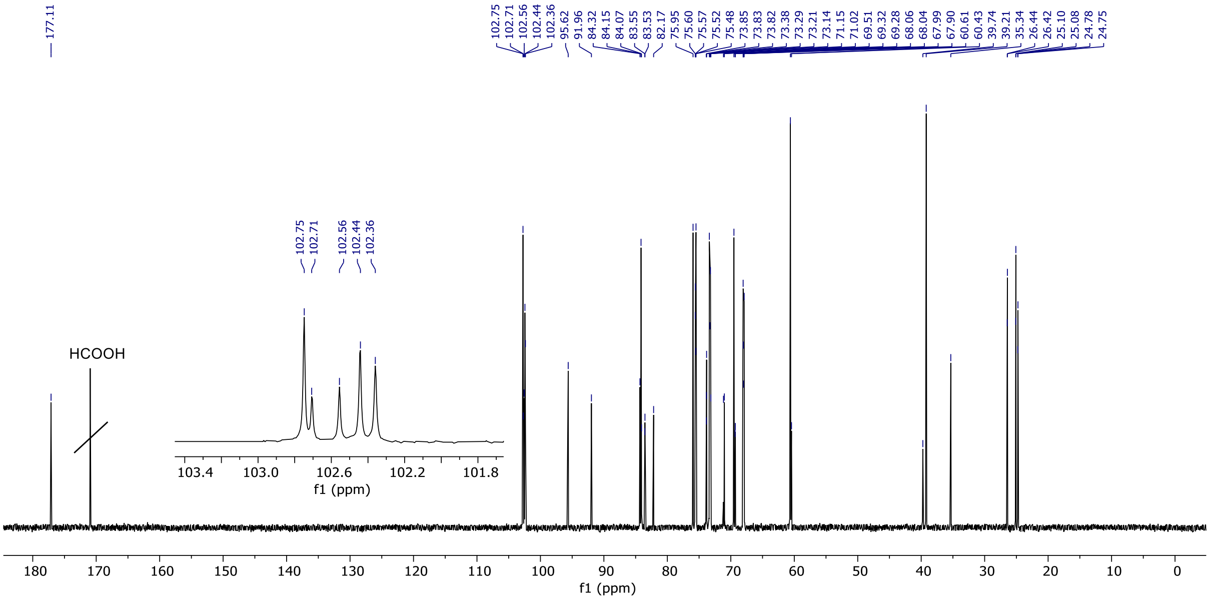

Figure S113. ^{13}C NMR (176MHz, D_2O) spectrum of **18**.

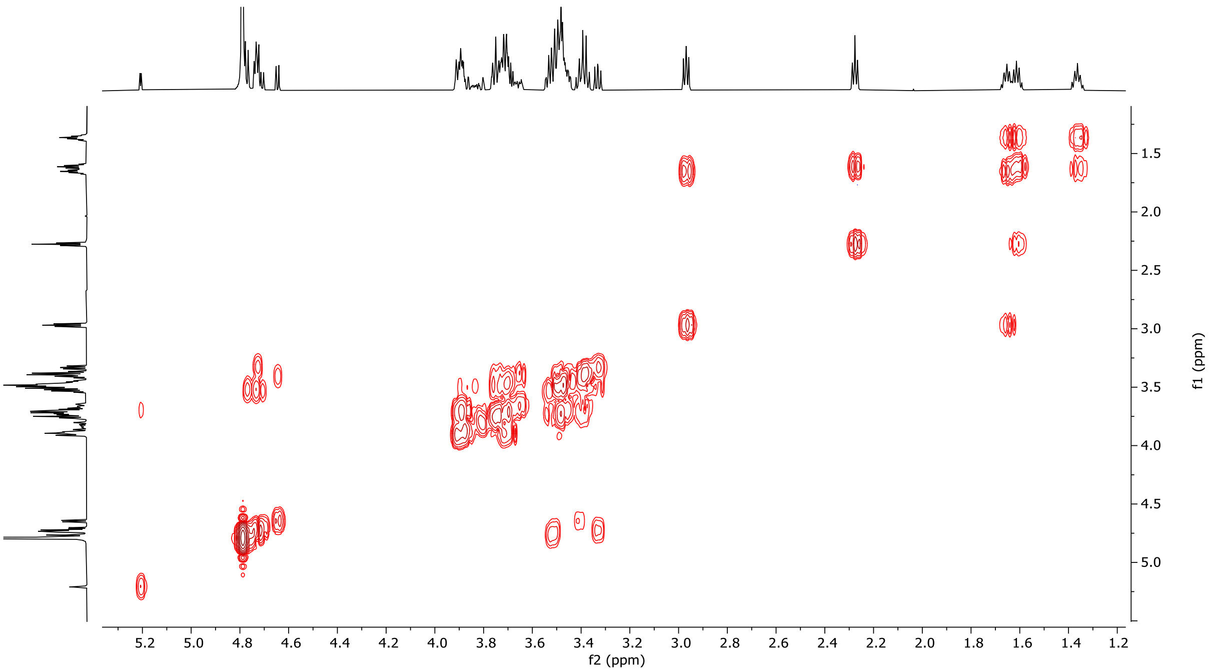

Figure S114. COSY NMR (700MHz, D_2O) spectrum of **18**.

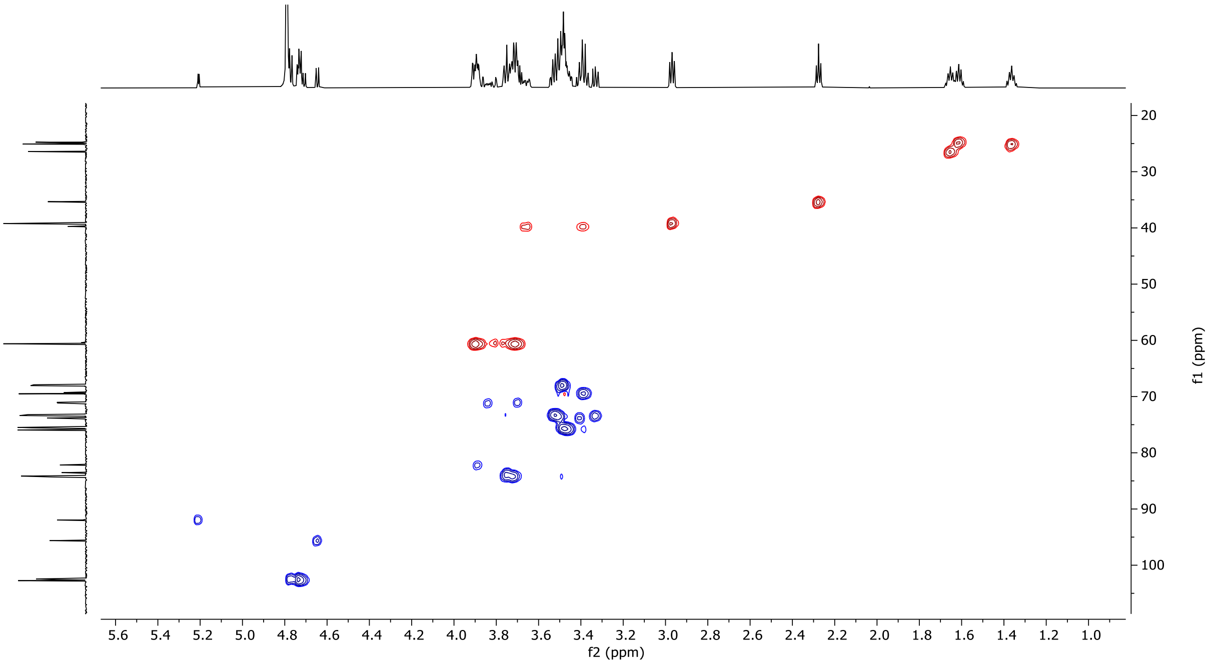

Figure S115. HSQC NMR (700MHz, D_2O) spectrum of **18.**

Synthesis and analytical data of 11

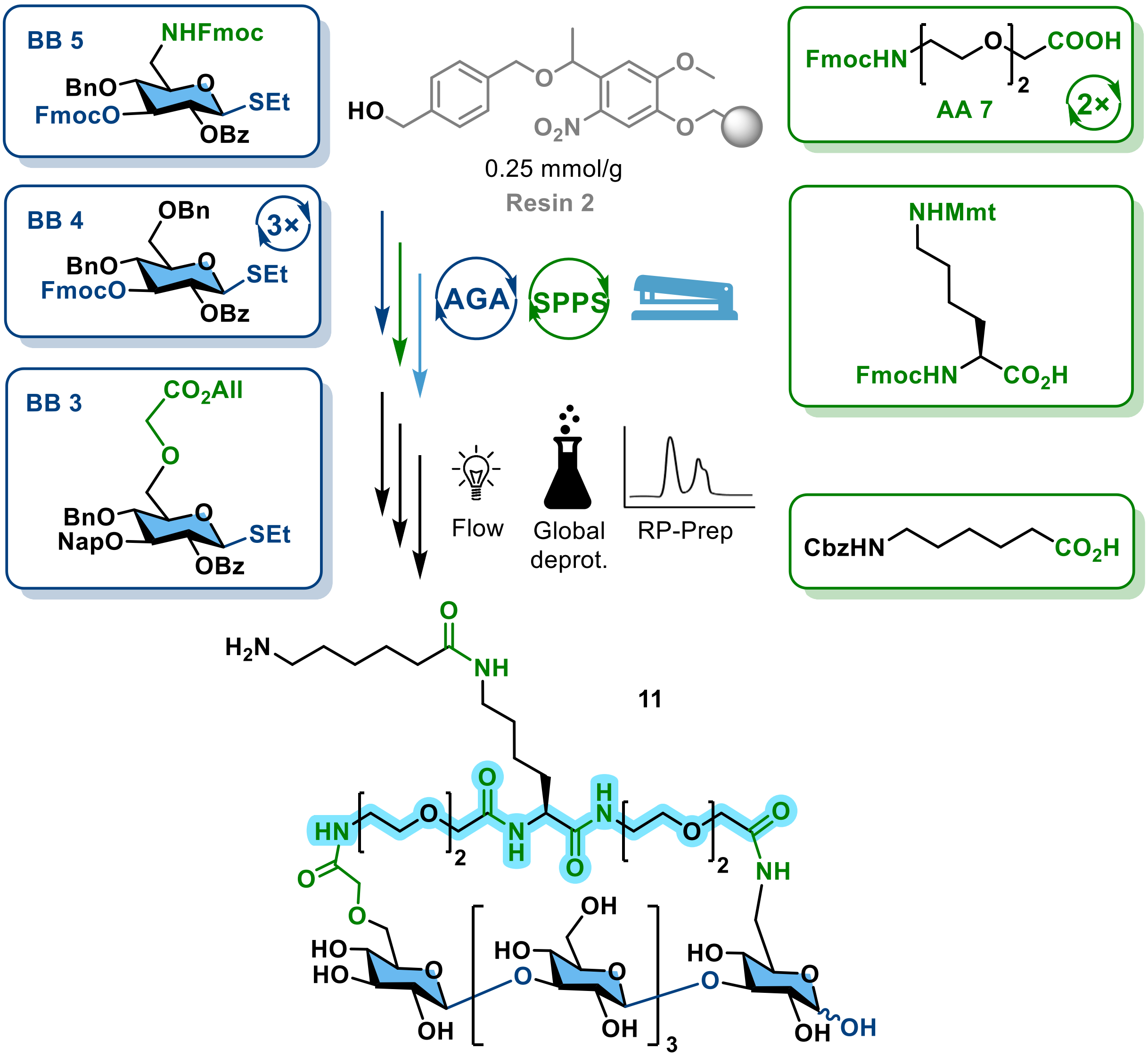

Step	Module	BB/reagent	Repeat	Notes
AGA	A	**2** (0.015 mmol)	1	0.25 mmol/g
	B, C2, D1	**5** (0.10 mmol)	1	-
	B, C2, D1	**4** (0.10 mmol)	3	-
	B, C2	**3** (0.10 mmol)	1	-
SPPS	E, F	**AA 7**	-	-
	E, F	Fmoc-Lys(Mtt)-OH	-	-
	E, F	**AA 7**	-	-
	H, F	CbzHN-C_5H_{10}-COOH	-	-
	I, E	-	-	-
Stapling	J	-	-	-

**Post solid-phase**	K1	-	-	-
	M	-	3	-
	N2	-	2	-
	O	-	-	Methods 2 and 5

After automated glycan assembly, side-chain construction, on-resin stapling, methanolysis, photo-cleavage, hydrogenolysis, purification, and lyophilization **11** was obtained as a white solid (2.4 mg, 12%).

R_t (Method 2) = 18.2 and 18.5 min (mixture of α- and β-anomers).

HRMS (ESI/Q-TOF) *m/z*: $[M + H + Na]^{2+}$ Calcd for $C_{56}H_{99}N_6NaO_{34}$ 711.3047; Found 711.3071.

^{1}H NMR (700 MHz, D_2O) δ 5.16 (d, *J* = 3.7 Hz, 1H, α-anomer), 4.65 (d, *J* = 8.0 Hz, 1H, β-anomer), 4.30 (t, *J* = 7.2 Hz, 2H), 4.17 – 4.04 (m, 13H), 3.96 – 3.83 (m, 11H), 3.81 – 3.68 (m, 36H), 3.64 (dtd, *J* = 18.2, 6.6, 3.3 Hz, 13H), 3.57 – 3.41 (m, 33H), 3.41 – 3.31 (m, 9H), 3.15 (td, *J* = 7.0, 4.7 Hz, 4H), 2.96 (t, *J* = 7.7 Hz, 4H), 2.22 (t, *J* = 7.3 Hz, 4H), 1.79 (h, *J* = 6.5 Hz, 2H), 1.72 (ddd, *J* = 20.8, 13.6, 8.0 Hz, 2H), 1.65 (p, *J* = 7.7 Hz, 4H), 1.59 (p, *J* = 7.6 Hz, 4H), 1.50 (p, *J* = 6.9 Hz, 4H), 1.35 (dh, *J* = 15.6, 7.7 Hz, 8H).

^{13}C NMR (176 MHz, D_2O) δ 176.42, 173.82, 173.81, 172.96, 172.92, 172.69, 172.49, 172.43, 172.40, 172.37, 171.32, 102.80, 102.46, 102.37, 102.13, 101.95, 101.79, 101.77, 95.65 (β-anomer), 92.08 (α-anomer), 84.18, 84.11, 82.83, 82.77, 82.64, 82.61, 80.86, 79.17, 79.15, 75.77, 75.60, 75.58, 75.51, 75.33, 74.85, 74.49, 74.17, 73.75, 73.72, 73.68, 73.25, 73.22, 73.14, 71.41, 70.84, 70.48, 70.41, 70.32, 69.94, 69.87, 69.40, 69.31, 69.26, 68.84, 68.76, 68.74, 68.52, 68.42, 68.01, 67.82, 67.80, 60.70, 60.65, 60.63, 53.47, 53.43, 39.20, 38.93, 38.63, 38.39, 35.46, 30.83, 30.81, 27.89, 26.39, 25.07, 24.85, 22.38.

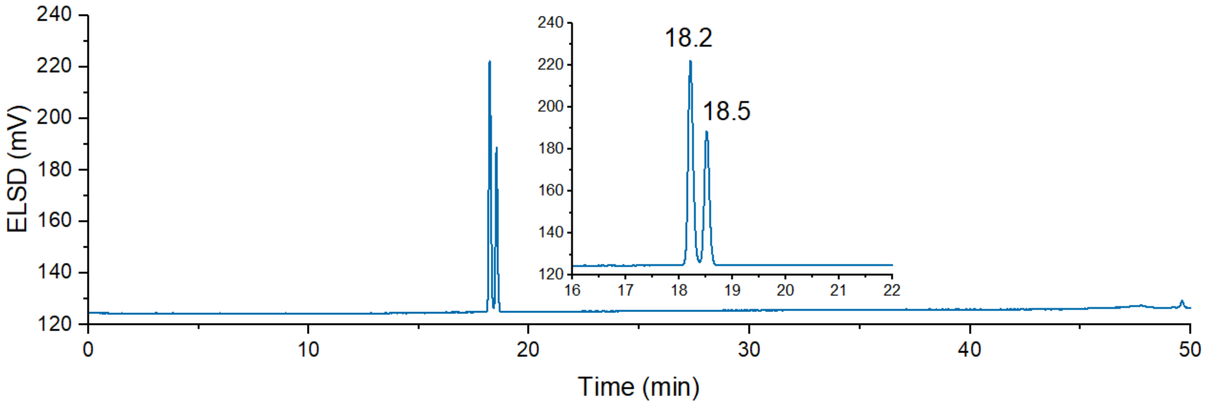

Figure S116. RP-HPLC trace of pure **11**.

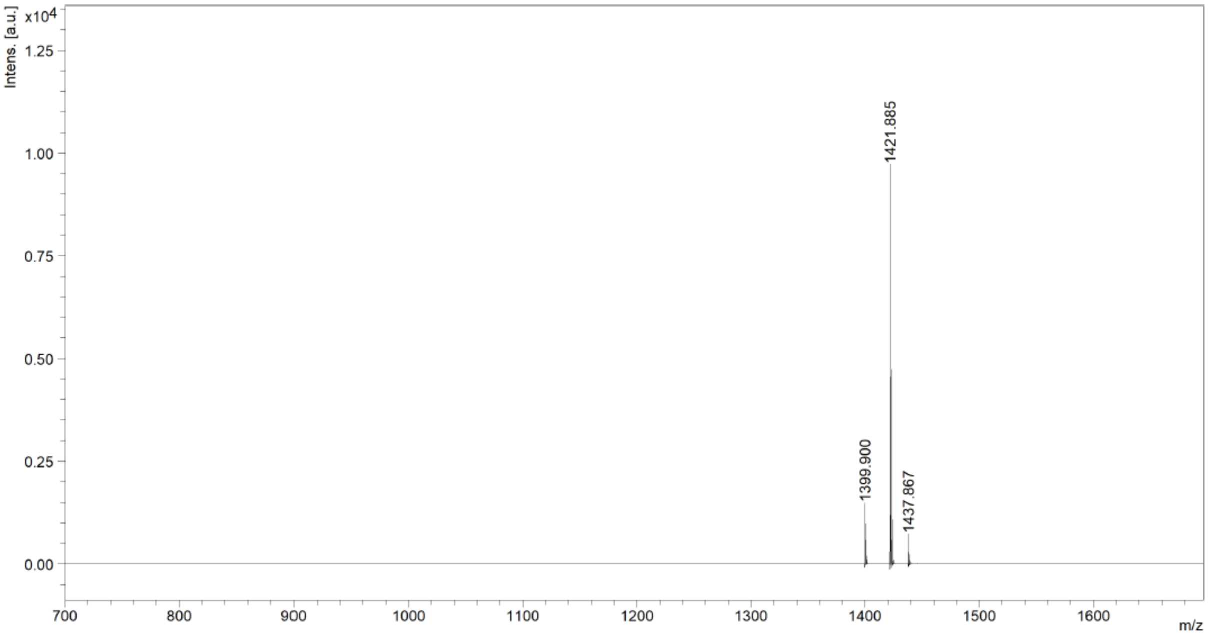

Figure S117. MALDI-TOF of **11**.

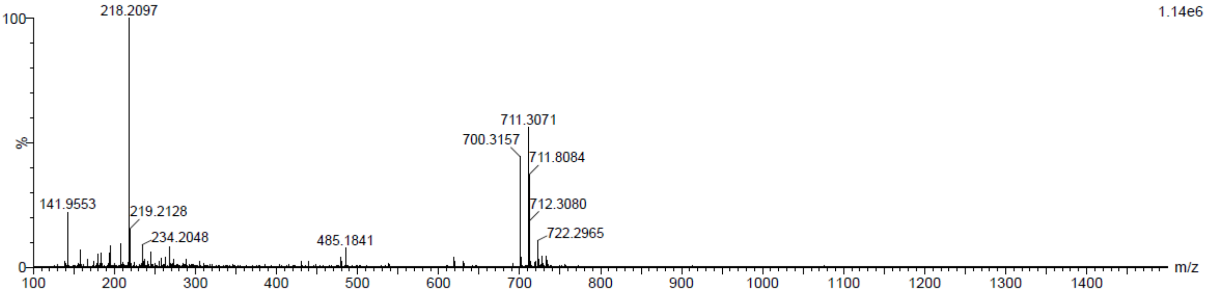

Figure S118. HR-MS of **11**.

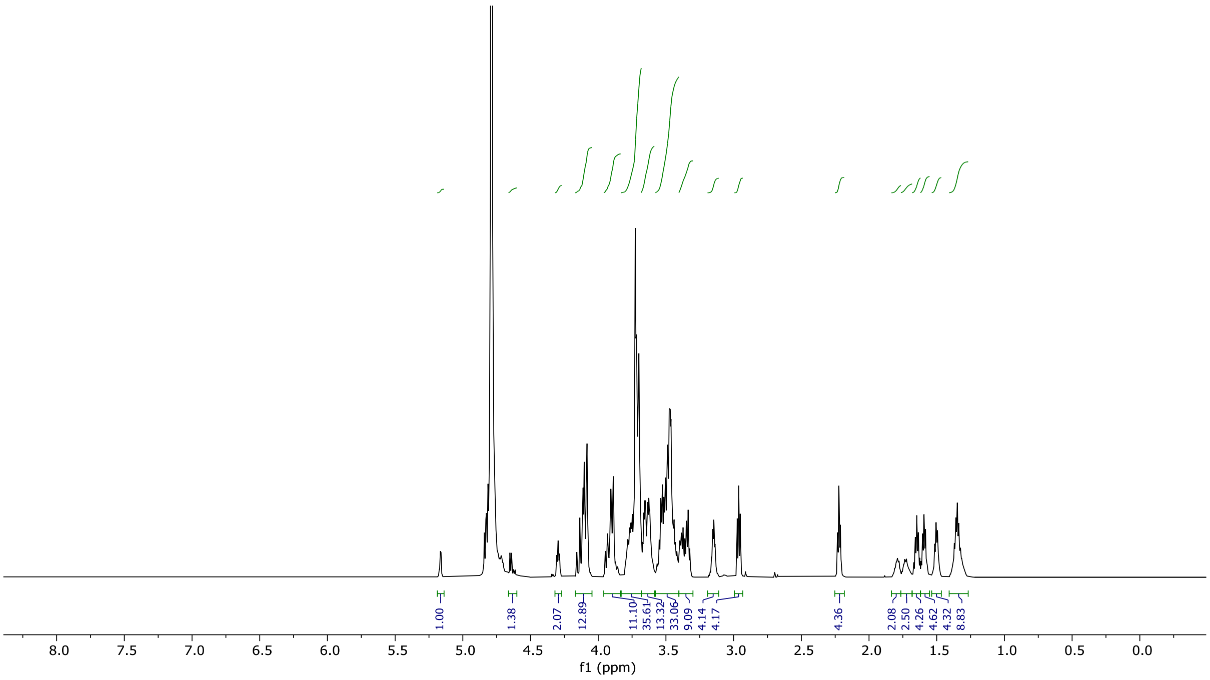

Figure S119. ^{1}H NMR (700MHz, D_2O) spectrum of **11**.

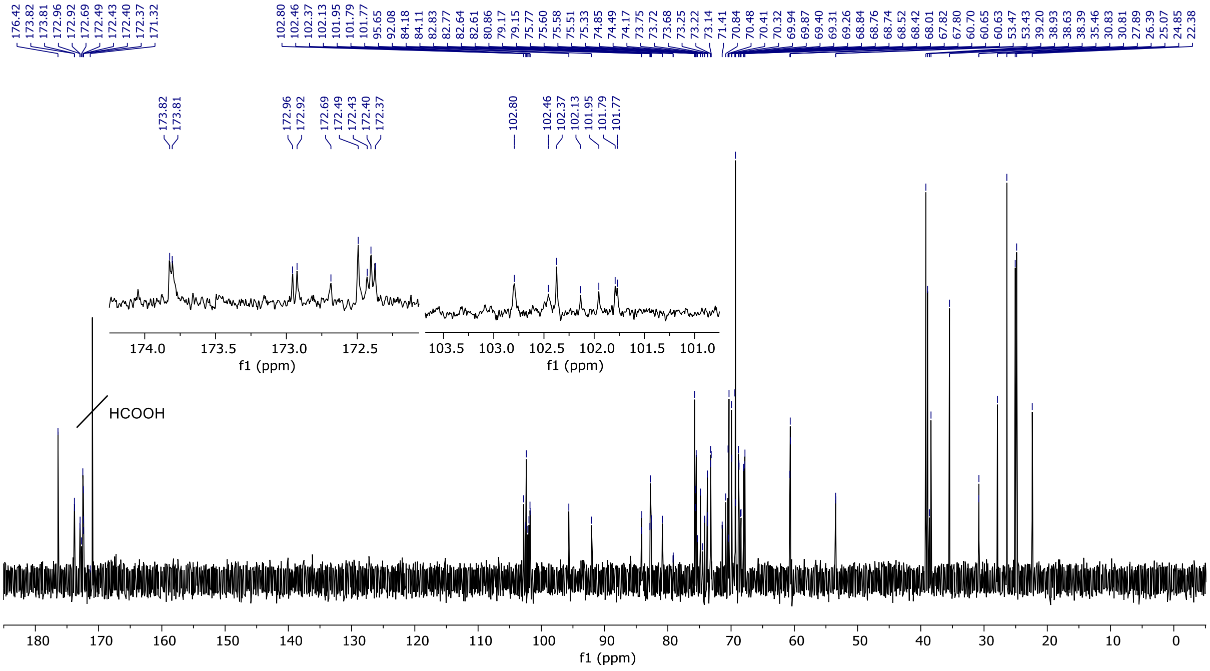

Figure S120. ^{13}C NMR (176MHz, D_2O) spectrum of **11**.

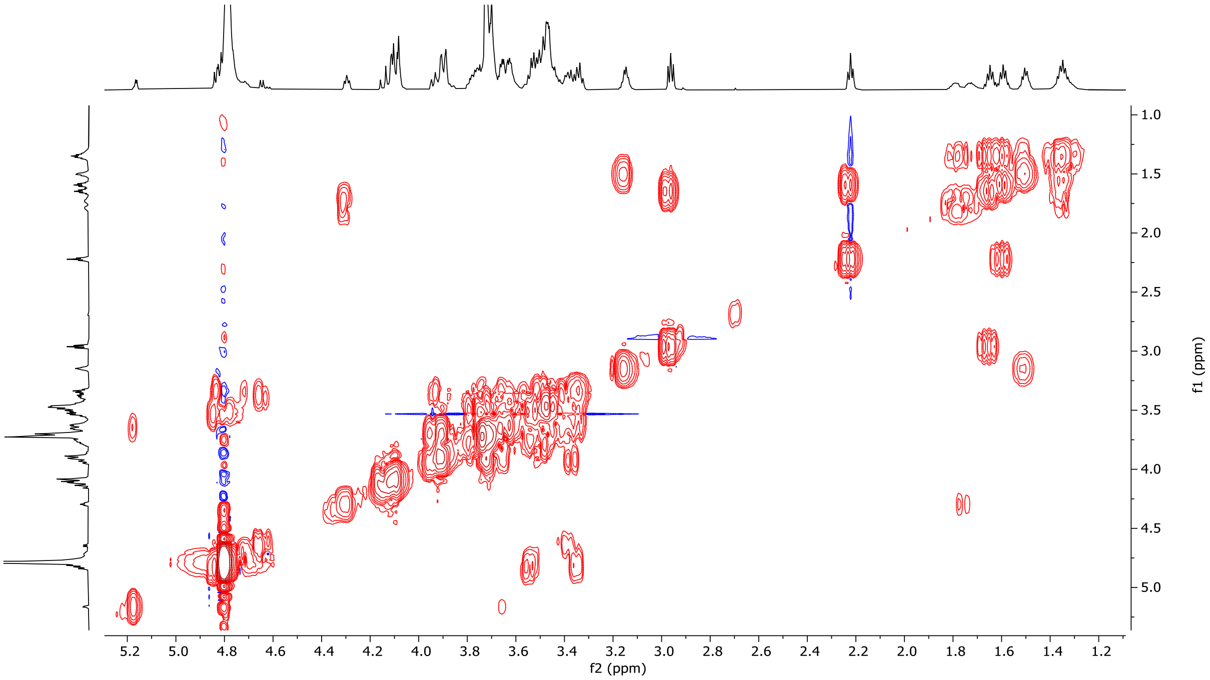

Figure S121. COSY NMR (700MHz, D_2O) spectrum of **11**.

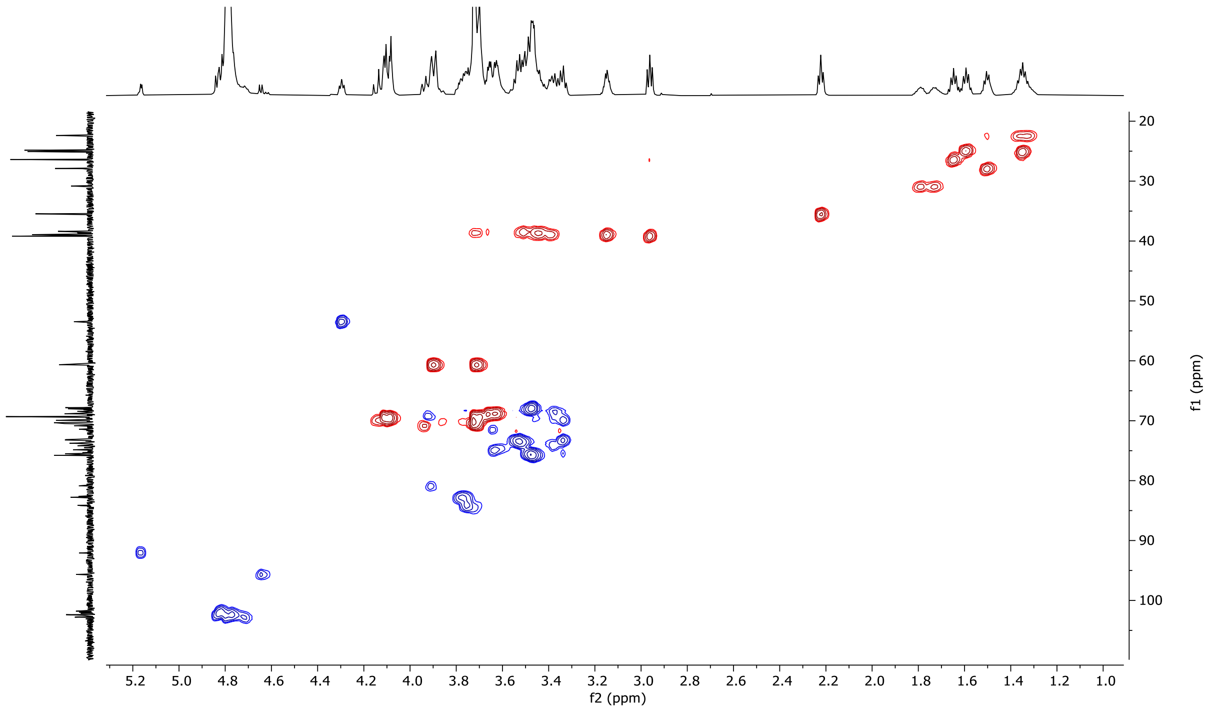

Figure S122. HSQC NMR (700MHz, D_2O) spectrum of **11**.

6. Molecular dynamics simulations

6.1. System setup

To obtain initial coordinates of the molecules for parametrizations and simulations, we generated PDB files of the glucan structures with the GLYCAM webserver[7] and PDB files of the linker structures with the CHARMM-GUI webserver[8]. These PDB files were aligned and combined with the software PyMOL. To generate force field parameters for the linkers with the AMBER software "antechamber" [9], we followed standard AMBER protocols for building non-standard residues. The PEG-2 and PEG-3 linkers were parametrized as residues that include the last glucose unit at the reducing end of the glucan, because the linkers are bound to this reducing end via an N-linkage for which the GLYCAM force field for glucans does not provide standard parameters. In this antechamber parametrization, force field parameters were generated with the general AMBER force field (GAFF) using the AM1-BCC charge model, and residue files for connecting to the remaining four glucan residues were created with the command "prepgen". For these remaining glucan residues, we used the force field $GLYCAM06_{OSMOr14}$, which was optimized for use with the TIP5P water to correct an overestimation of attractive glucan interactions in the GLYCAM06 force field and other standard force fields[10]. The C12 and C16 linkers were parametrized with antechamber as two residues containing 6 and 8 C atoms, respectively. For connecting these linkers to the reducing end of the glucan, the glucose unit at this end was changed to xylose. All systems were finally hydrated in octahedral TIP5P simulation boxes.

6.2. Generation and analysis of simulation data

After standard minimization and relaxation procedures, we generated 5 independent simulation trajectories with a length of 1 microsecond and 4 additional independent simulation trajectories with a length of 5 microseconds for each system. The total simulation time thus was 25 microseconds for each system. In these MD simulations, the temperature was kept at 300 K using a Langevin thermostat with a collision frequency of 1 ps^{-1}, the pressure was maintained

---

[7] Grant, O. C.; Wentworth, D.; Holmes, S. G.; Kandel, R.; Sehnal, D.; Wang, X.; Xiao, Y.; Sheppard, P.; Grelsson, T.; Coulter, A.; et al. Generating 3D Models of Carbohydrates with GLYCAM-Web. *bioRxiv* **2025**, 2025.2005.2008.652828.

[8] Jo, S., Kim, T., Iyer, V.G. and Im, W. (2008), CHARMM-GUI: A web-based graphical user interface for CHARMM. J. Comput. Chem., 29: 1859-1865.

[9] Wang, J.; Wang, W.; Kollman, P. A.; Case, D. A. Automatic atom type and bond type perception in molecular mechanical calculations. *Journal of Molecular Graphics and Modelling* **2006**, *25* (2), 247-260.

[10] Sauter, J.; Grafmüller, A. Predicting the Chemical Potential and Osmotic Pressure of Polysaccharide Solutions by Molecular Simulations. *J. Chem. Theory Comput.* **2016**, *12* (9), 4375-4384.

at 1 bar with a Berendsen barostat, a cutoff length of 10 Å was used for non-bonded interactions, and long-range electrostatic interactions were treated with the Particle Mesh Ewald (PME) method. In addition, hydrogen mass repartitioning[11] was employed, which allowed for a simulation time step of 4 femtoseconds. All simulations were performed with the Amber20 software[12] on graphics processing units (GPUs).

For the analysis of equilibrium properties, the first 200 ns of each trajectory were discarded as additional relaxation time. The glucan end-to-end distributions in Figure … was calculated between the centers of the terminal glucan rings, which were determined as the center of mass of the C1 and C4 atoms of the rings. Statistical errors were determined based on five independent sets of trajectories with a total length of 5 microseconds. In one of these sets, the data from the five simulation trajectories with length of 1 microsecond were combined. The statistical errors of the distribution in Figure 5 represent the standard error of the mean of distributions determined for these five datasets.

7. Glycan array analysis

The glycans were dissolved at 0.1 mM in 50 mM sodium phosphate buffer pH 8.5 and immobilized in 56 identical fields on *N*-hydroxysuccinimide (NHS) ester-activated hydrogel glass slides (NEXTERION® 3-D Hydrogel coated glass slides, Schott) using a non-contact sciFLEXARRAYER S12 microarray spotter (Scienion, Berlin, Germany). After incubation overnight in a humidified box, the remaining NHS groups of the slides were quenched with ethanolamine. The slides were blocked with 1% (w/v) bovine serum albumin (BSA) in phosphate buffered saline (PBS) and a 64 well incubation gasket (FlexWell Grid, Grace Bio Labs) was attached. The slides were incubated with 10 µg/mL mouse monoclonal antibody 2G8 (abcam, Cat. ab233743) or rabbit monoclonal antibody 8201 (ThermoFisher Scientific, Cat. MA5-33305) in 1% BSA-PBS for 1 h at 37° C. After three washes with PBS containing 0.1% (v/v) Tween-20 (PBS-T) the slides were incubated with AlexaFluor™ 488 goat anti-rabbit IgG (H+L)

---

[11] Hopkins, C. W.; Le Grand, S.; Walker, R. C.; Roitberg, A. E. Long-Time-Step Molecular Dynamics through Hydrogen Mass Repartitioning. *J. Chem. Theory Comput.* **2015**, *11* (4), 1864-1874.

[12] D.A. Case, K. Belfon, I.Y. Ben-Shalom, S.R. Brozell, D.S. Cerutti, T.E. Cheatham, III, V.W.D. Cruzeiro, T.A. Darden, R.E. Duke, G. Giambasu, M.K. Gilson, H. Gohlke, A.W. Goetz, R. Harris, S. Izadi, S.A. Iz-mailov, K. Kasavajhala, A. Kovalenko, R. Krasny, T. Kurtzman, T.S. Lee, S. LeGrand, P. Li, C. Lin, J. Liu, T. Luchko, R. Luo, V. Man, K.M. Merz, Y. Miao, O. Mikhailovskii, G. Monard, H. Nguyen, A. Onufriev, F. Pan, S. Pantano, R. Qi, D.R. Roe, A. Roitberg, C. Sagui, S. Schott-Verdugo, J. Shen, C.L. Simmerling, N.R. Skrynnikov, J. Smith, J. Swails, R.C. Walker, J. Wang, L. Wilson, R.M. Wolf, X. Wu, Y. Xiong, Y. Xue, D.M. York and P.A. Kollman (2020), AMBER 2020, University of California, San Francisco.

(Invitrogen, Cat. A11008) in case of mAb 8201 or AlexaFluor™ 635 goat anti-mouse IgG (H+L) (Invitrogen, Cat. A31574) in the case of mAb 2G8 diluted 1:400 for 1 h at 37°C. The slides were washed twice with PBS-T. After removing the gasket, the slides were washed once with PBS and once with water. The dried slides were scanned with an InnoScan 1100 Fluorescence Scanner (Innopsys). Intensities were evaluated with Mapix 9.1.0 (Innopsys). The statistical analysis was performed with the software GraphPad Prism 10.4.0 (GraphPad Software, Inc.).
